# Slow slip modulates low-frequency seismicity on the Parkfield segment of the San Andreas Fault

**DOI:** 10.1038/s41467-026-74095-9

**Published:** 2026-06-09

**Authors:** Zahra Zali, Patricia Martínez-Garzón, David Mencin, Gregory C. Beroza

**Affiliations:** 1 GFZ Helmholtz Centre for Geosciences, Potsdam, Germany; 2 RWTH Aachen University, Aachen, Germany; 3 EarthScope Consortium, Washington, DC USA; 4 Department of Geophysics, Stanford University, Stanford, CA USA

**Keywords:** Seismology, Natural hazards, Geophysics

## Abstract

Understanding how slow slip events (SSEs) influence fault behavior is essential for characterizing the fault slip spectrum and its role in earthquake generation. Here, we show that deep learning applied to strainmeter data can detect short-duration SSEs on the San Andreas Fault near Parkfield, enabling an SSE catalog. SSEs are coherently observed across instruments, with evidence from nearby creepmeters. Location analysis indicates shallow depths and slip consistent with right-lateral motion. They follow a cubic moment–duration scaling law, similar to earthquakes and consistent with both subduction zone observations, and linear scaling as an upper bound. Low-frequency earthquakes increase following SSEs, suggesting that slow aseismic slip modulates seismicity. Detecting these SSEs fills an observational gap in slow earthquake studies and highlights their broader relevance. These findings support a continuum between aseismic and seismic slip, where transient deformation in creeping segments perturbs stress in adjacent locked areas, potentially promoting seismic activity.

## Abstract (web summary)

Using deep learning and strainmeter data, the authors detect short-duration slow slip events on the San Andreas Fault at Parkfield and show that these events are linked to increased seismic activity and have scaling laws similar to earthquakes.

## Introduction

Faults release tectonic stress through both fast (seismic) and slow (aseismic) slip^1,2^. Aseismic slip includes steady fault creep and transient episodes such as afterslip and slow slip events (SSEs), which can last from minutes to months. SSEs release accumulated strain along faults and contribute to long-term moment release^3,4^, potentially reducing the elastic energy available for large earthquakes^5,6^. They can influence seismic hazard by occurring before^7,8^, during^9^, or after earthquakes^10–13^, and in some cases may trigger seismic events^8,14–18^. The physical mechanisms that control the occurrence of SSEs remain incompletely understood. Whether a fault slips seismically or aseismically depends on several factors, including physical properties (temperature and pressure), fault zone structure, material properties^19,20^, mineralogical composition of the fault gouge^21^, and the presence of geometrical heterogeneities^22^. SSEs tend to occur in specific depth ranges, typically either in the shallow upper crust, such as along the selected segments of the San Andreas Fault in California, where steady creep is common^23^, or at greater depths within subduction zones, for example, the Cascadia^24^ and Nankai margins^25^, where slow slip occurs in the transition zone between locked and creeping segments. These regions may promote slow slip due to a combination of elevated pore fluid pressures, which reduce effective normal stress^26,27^, and velocity-strengthening frictional behavior that favors stable sliding over seismic rupture^20,28^. Such conditions are often inferred near the base of the seismogenic zone or within weak fault materials. In transform fault settings such as the San Andreas Fault, SSEs have also been reported, though their small magnitude and short duration are challenging to detect using either geodetic or strain-based observations^29–31^.

Borehole strainmeters (BSMs) are highly sensitive to strain changes in the surrounding Earth, enabling them to capture subtle deformation that may be missed by high-precision GPS, thereby bridging the measurement gap between seismometers and GPS^32^. Their ability to capture deformation across timescales ranging from seconds to weeks makes them valuable for detecting transient aseismic phenomena, including short-duration SSEs. Despite their potential, BSM data are often dominated by environmental and instrumental noise, which can obscure subtle tectonic signals^33^. As a result, confirmed SSE detections using strainmeter data are rare and typically rely on visual inspection or simple threshold-based criteria^13,34^, which are time-consuming, prone to subjectivity, and often ineffective due to noise levels in the data^31,35^. The identified SSEs in tectonic fault settings have been mostly observed on individual strainmeter stations near the San Andreas Fault^36^, the Marmara region in Turkey^13,34^, and the Alto Tiberina Fault in Italy^37^, highlighting both the potential of these instruments for studying aseismic slip and the necessity to develop methodologies that enable their systematic detection in noisy environments.

In this study, we present a deep learning-based algorithm to detect SSEs embedded in continuous strainmeter data. We employ a multi-step workflow that includes wavelet-based signal representation, dimensionality reduction via a neural autoencoder, and unsupervised clustering. This approach allowed us to detect SSEs on up to three independent strainmeter stations in the Parkfield section of the San Andreas Fault, with the associated slip also observed on a nearby creepmeter. The spatial coherence of these events enabled source modeling, revealing that the SSEs are shallow, exhibit slip consistent with the right-lateral motion of the San Andreas Fault, and follow a cubic moment–duration scaling law similar to regular earthquakes. Previous studies either could not detect SSEs in strainmeter data due to their small amplitudes and high noise levels, or identified them only on individual stations. The SSEs observed across multiple strainmeter stations provide robust evidence of the spatial coherence of these signals and enable analysis of both the signal characteristics and the spatial extent of the events that cause them. This allows us to explore the moment–duration scaling analysis of short-term SSEs in a transform fault setting, extending earthquake-like scaling observations to shallow aseismic slip.

**Fig. 1:**
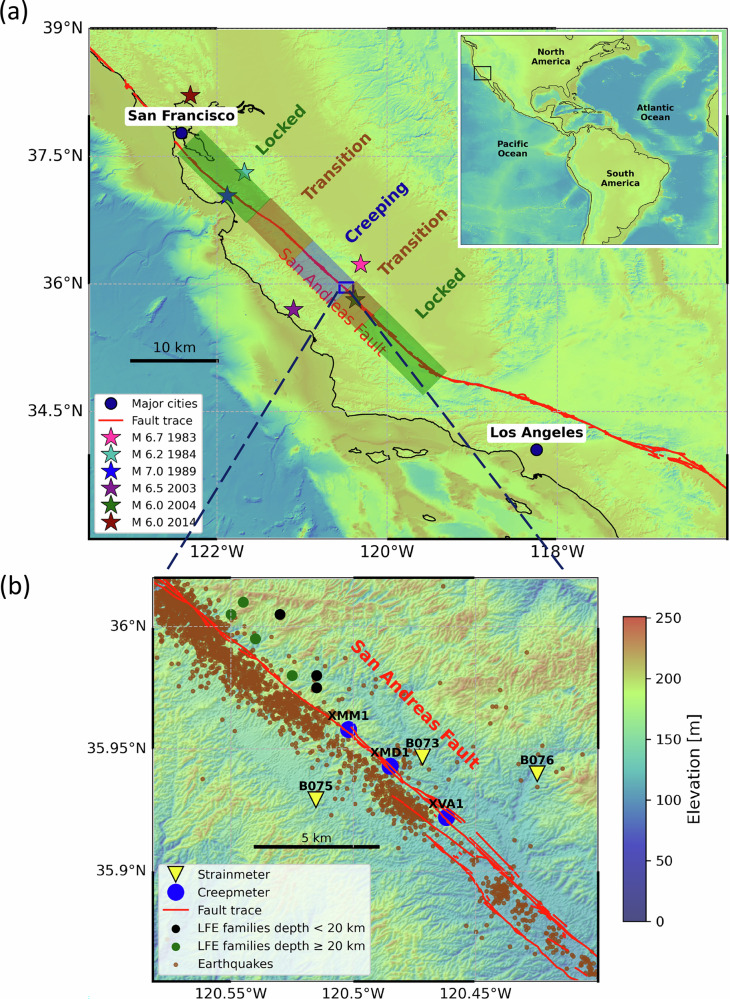
Location of the study area and instruments on the creeping section of the San Andreas Fault, near Parkfield, California. **a** Map showing the San Andreas Fault trace, earthquakes with M > 6 since 1983, and major nearby cities. The creeping, transition, and locked zones are based on previous studies^86^. **b** Detailed view of the study area highlighting the strainmeters and creepmeters used in this study. Black and green circles represent the low-frequency earthquake catalog^70^. Brown dots show earthquakes from the Northern California Earthquake Data Center. Red lines represent mapped surface fault traces from the United States Geological Survey (USGS) Quaternary Fault and Fold Database (Qfault), including the San Andreas Fault.

SSEs have been studied on several strike-slip faults in California^38,39^. Seismic manifestations of ongoing slow processes such as SSE commonly include tremor, very-low-frequency earthquakes, earthquake swarms, and low-frequency earthquakes (LFEs)^40–42^, all of which may occur in response to aseismic stress transients^41^. In many regions, small seismic events, including LFEs, are viewed as indirect indicators of slow aseismic slip, because stress changes associated with gradual fault slip can promote brittle failure on small asperities embedded within the slipping region^43^. This spatial and temporal association is often interpreted as reflecting related slow-slip processes acting within the same fault zone. The temporal correlation between LFEs and SSEs may arise from localized brittle failure within a broader region undergoing slow aseismic slip, as LFEs are generally interpreted to originate from small patches embedded within a larger, slowly slipping fault area^44–47^. The coupling between SSEs and LFEs has been observed mainly in subduction zones^40,47–51^, and similar relationships between aseismic slip transients and seismic events have also been documented on several strike-slip faults in California^52–54^. Understanding the relationship between SSEs and LFEs is critical for understanding both aseismic and seismic fault behavior and may offer insight into the mechanisms governing earthquake occurrence and recurrence^14,18^. In our study, we show that low-frequency earthquakes (LFEs) near Parkfield (Fig. 1) tend to follow the detected SSEs, suggesting a potential causal relationship between aseismic slip and subsequent LFE activity. This behavior aligns with earlier observations in both subduction and strike-slip tectonic regimes, where variations in LFE activity have been shown to track the evolution of slow slip transients. These observations place our study within a broader context of aseismic–seismic coupling on strike-slip faults.

## Results

### A machine learning approach to detect slow slip events in strainmeter data

We present a machine learning-based approach for automatically detecting Slow Slip Events (SSEs) embedded in strainmeter records. Our approach uses deep learning techniques to overcome the challenges of traditional methods and identify SSEs from large datasets, enhancing the potential for systematic analysis of strainmeter records.

**Fig. 2:**
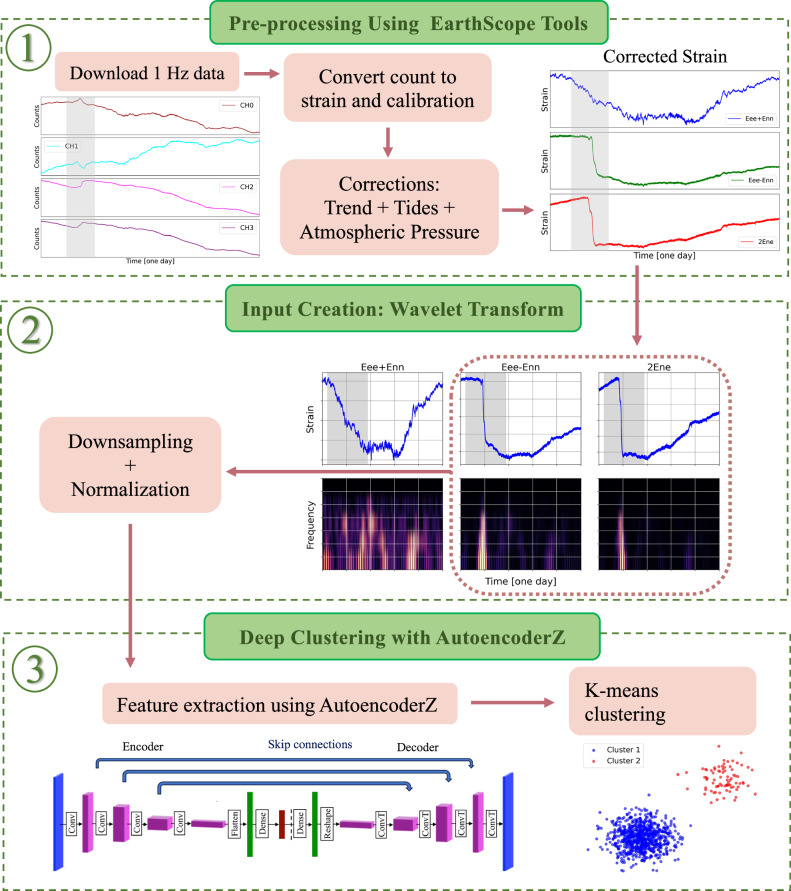
Flowchart of our slow slip event (SSE) detection algorithm for strainmeter data. The workflow includes three main steps: (1) preprocessing of strainmeter data, (2) wavelet transform for signal representation, and (3) deep learning involving feature extraction with AutoencoderZ followed by K-means clustering. The AutoencoderZ architecture shown in panel 3 is adapted from our previous studies^55,56^, with modifications.

Our workflow includes three main steps (Fig. 2): (I) Pre-preprocessing: Raw strain data is corrected for long-term trends, tidal effects, and atmospheric pressure using EarthScopeStrainTools^33^. We then convert gauge strain to regional strain (areal, differential, engineering) via a calibration matrix. (II) Signal representation: We use the continuous wavelet transform (Morlet wavelet) to enhance transient signals such as SSEs. We then compute daily wavelet spectra, downsampled for efficiency, and normalized for input into the machine learning model. The wavelet transform (WT) provides a representation in which short-duration step-like strain transients produce a characteristic broadband, high-amplitude pattern that is visually distinct from background noise (Fig. 2) and is generally distinguishable from short-lived signatures of local earthquakes. This representation, therefore, serves as the basis for distinguishing transient deformation from other sources of variability in the strain records. III) Feature extraction and event detection via clustering: To cluster the daily wavelet representations, the dimensionality of the data must be reduced because each day of data contains 86,400 samples and clustering performance degrades in high-dimensional spaces. We therefore use AutoencoderZ, a deep autoencoder model developed in our previous studies^55,56^, to learn a compact set of features that summarizes each day’s WT. The encoder compresses each WT into a latent vector of 24 features, which preserves key characteristics of the signals. In this low-dimensional feature space, K-means clustering groups days with similar WT patterns. We use only differential and engineering strains as input to the model, as they are most sensitive to tectonic deformation^32^. The method is applied separately to each component, and detections from the differential and shear components are then combined to account for variability in signal amplitude across the two components. The first clustering stage separates days that contain a transient strain change from those that show only background variability. The second stage operates only on the transient days, isolating short-duration SSEs (which share a characteristic broadband WT signature) from other transient sources, such as local earthquakes or instrumental noise. Because the approach is fully unsupervised, the clusters arise directly from differences in the learned signal features, allowing efficient identification of SSEs and offering potential for near-real-time tectonic monitoring using strainmeter data. After clustering and automatic detection, we visually inspect the detected events to remove any remaining non-tectonic or unwanted signals.

The method was developed and tested with data from the Parkfield section of the San Andreas Fault (Fig. 1a), where a long-term network of strainmeters exists, and a manual SSE catalog from visual inspection of the continuous recordings is available for validation^57^. We use three strainmeters: B073 (reference strainmeter; closest to the fault and consistently recording the highest-amplitude signals), B075, and B076, which were selected based on their proximity to the fault and each other (Fig. 1b). The dataset includes the four gauges of each strainmeter, spans the years 2009–2016, and has a sampling frequency of 1 Hz to resolve short-duration events.

### SSEs recorded on three strainmeters at Parkfield

We used our algorithm to detect SSEs across multiple strainmeter stations. We first applied it to station B073, which is the reference strainmeter for the manual SSE catalog and is located 1.1 km from the San Andreas Fault. The manual catalog covered the years 2009 to 2016 and included 71 events that, in most cases, were also observed on a nearby creepmeter. By comparing the automatic detections with the manual catalog, we found that our unsupervised algorithm automatically detected 90% of the SSEs listed in the manual catalog over the 8-year interval. The durations of our short-duration SSEs range from 25 to 100 minutes, with an average duration of approximately 50 minutes. Hence, this fills an observational gap of slow slip events observed using seismic and geodetic instrumentation.

To assess whether a detected transient represents an SSE, we evaluated the waveform characteristics following the criteria used in ref. ^57^. We visually inspected whether the signal showed a step-like strain change evolving over tens of minutes, with the largest amplitudes appearing in the differential and engineering strain components compared to the areal component. Where needed, we also examined the individual gauge records, since tectonic deformation affects multiple gauges consistently, whereas instrumental artifacts often appear in only a single gauge. When available, supporting offsets on nearby creepmeters were also taken into account. We also considered the overall transient shape together with the strain patterns produced by shallow dislocation models (e.g., Fig. 6 in ref. ^57^), which provides an additional qualitative check that the signal is consistent with aseismic fault slip. The 7 missed events exhibit amplitude changes of around 0.01 microstrain on the strainmeter. While some detected SSEs show similarly small amplitudes, they typically occur over shorter durations (often less than half an hour) and generate sharp transients in the daily wavelet transform images. In contrast, the missed events evolve over longer timescales, from about half an hour to 100 min, producing smoother transients that are more difficult to distinguish from background signal variations. The instrumental dynamic range of GTSM borehole strainmeters is large (digitizer resolution ~10⁻¹⁰ strain and observed signals up to ~10⁻⁵ strain^58^). However, the effective detection threshold for longer-duration deformation is controlled by noise. Spectral analyses in previous studies^58^ show a pronounced rise in noise for periods longer than ~1000 s, which overlaps with the durations of the SSEs in our catalog. As a result, small-amplitude events (~0.01 µstrain) lie near the practical noise level at these periods, explaining why some low-amplitude transients were missed by the automated method. The missed events were excluded during the second clustering step, where the algorithm prioritizes reducing false positives. In addition to the 90% detection rate, we identified 3 signals with sharp features in the wavelet transform, but based on waveform shape and amplitude distribution across the three components, we do not interpret them as SSEs (Figs. S1–S3). We also observe 16 false detections linked to short-duration (~16 min) step-like jumps that occur only during a specific period of the record (primarily in 2016), likely due to instrumental noise (see Fig. S4 for an example).

**Fig. 3:**
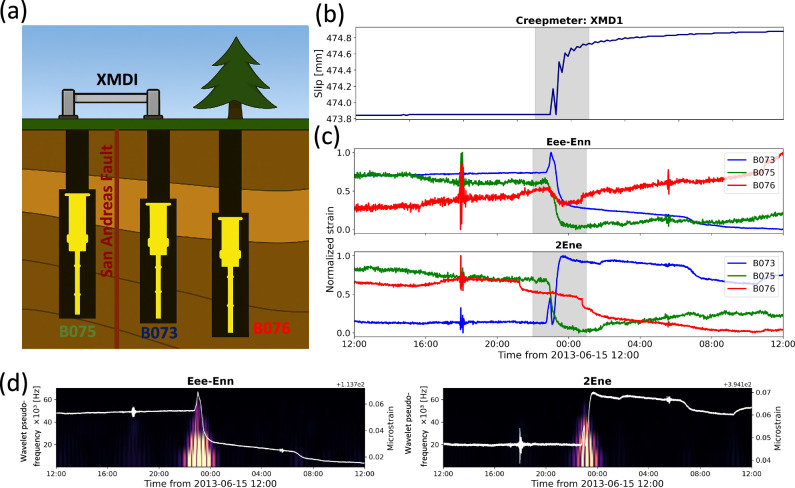
Example of a newly detected slow slip event (SSE) identified by our algorithm. **a** Schematic illustration of the instrument configuration, showing the surface creepmeter XMD1 and three borehole strainmeters (B075, B073, B076) installed at ~150 m depth around the San Andreas Fault. **b** Creepmeter signal indicating surface slip associated with the SSE. **c** Normalized strainmeter time series from the three strainmeters. The largest strain signal is observed at B073, followed by B075, with the smallest at B076, consistent with their relative positions to the fault. **d** Daily wavelet transform (WT) of the two strainmeter components. The corresponding strainmeter signals are overlaid in white. The y-axis shows wavelet pseudo-frequencies obtained from the scale–frequency mapping of the continuous wavelet transform.

Our algorithm identified 21 SSEs (representing 30% of the total) that are not present in the manual catalog (three examples of newly detected SSEs are shown in Figs. S5–S7). Out of these new detections, 14 of them were further confirmed by their visibility in nearby creepmeters (see Figs. 3, S8). Some of the SSEs did not show slip on the creepmeters, which may suggest a deeper source for these events, but a visual inspection of the strainmeter data confirmed their occurrence. The new detections highlight the ability of our method to uncover previously unknown patterns. We also investigated the possible existence of longer-duration SSEs in the Parkfield strainmeter data using extended wavelet scales, but did not identify any longer-duration events at station B073. This analysis, together with illustrative examples and the corrected strainmeter time series for the full 8-year period, is described in detail in Supplementary Note 1 and the figures therein. All 92 SSEs introduced in this study are provided in Supplementary Data 1, while the corresponding strainmeter waveforms, wavelet transform plots, and the traces of the three nearby creepmeters are presented in Supplementary Data 2.

To our knowledge, no automated SSE detection algorithm has been previously developed solely on strainmeter data. A region-specific matched-filter method combining strain and tilt data was used to detect short-term SSEs in southwest Japan by modeling slip on the plate interface and using the Akaike Information Criterion (AIC) for event identification^59^. Their method detects many SSEs from a GNSS-based catalog but misses small events and produces some false detections. Compared to other methods, our detection rate also exceeds SSEDetector^60^, a supervised approach trained on synthetic GNSS data, which achieved an 87.5% detection rate.

We then applied the algorithm to the neighboring stations B075 and B076, which are located farther from the fault than B073, at distances of 2.9 km and 3.4 km, respectively (Fig. 1b). The increased distance from the fault results in a decrease in the SNR of the SSEs, making the detection of these events more challenging. Still, in strainmeter B075, our automatic algorithm was able to detect 60% of them. A visual check of the signals revealed that 20 of the 26 missed events could still be observed, albeit with very small amplitudes (<2 nstrain). Approximately half of the data from the B076 from our study period is corrupted. From the remaining data, we were able to visually detect 42% of the SSEs, though with very small amplitude changes compared to the two previous stations. Automatic detection in this station is particularly challenging due to the presence of numerous other transient signals (most likely non-tectonic) that interfere with the identification of smaller changes. As a future improvement, one could incorporate external earthquake catalogs to mask known seismic arrivals, which would reduce false positives and improve clustering quality by filtering out unrelated transients. Additionally, signal separation or transient removal techniques^61,62^ could help suppress non-tectonic signals and enhance the sensitivity of the detection algorithm. However, this would require careful assessment to avoid excluding strain transients that may be temporally correlated with SSEs and to define appropriate magnitude and distance thresholds.

### Similar scaling laws for earthquakes and short-term SSEs in Parkfield

**Fig. 4:**
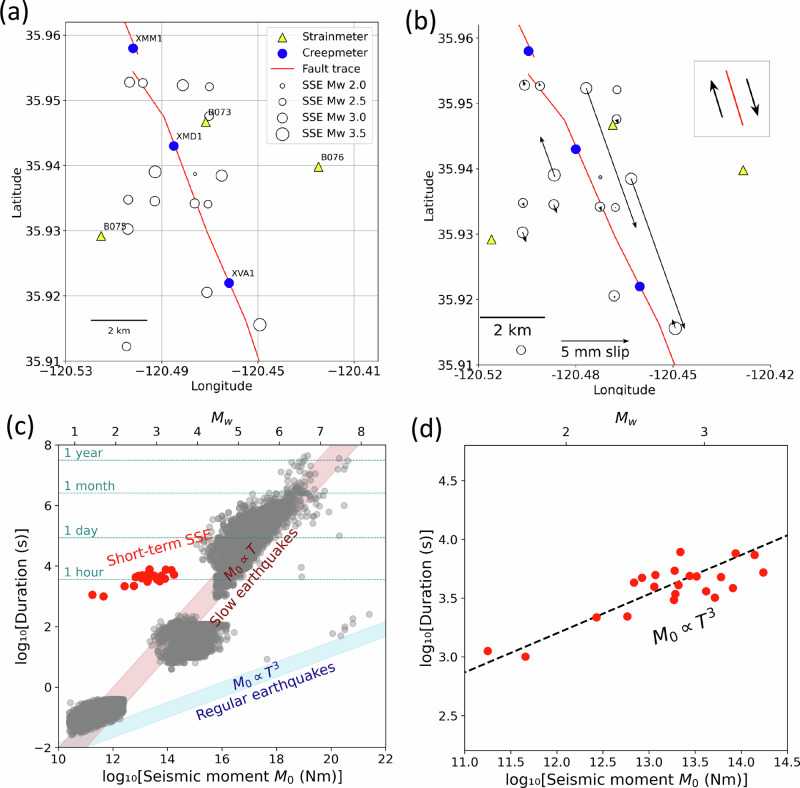
Source analysis and moment–duration scaling of short-term slow slip events (SSEs). **a** Estimated source locations and depths of 22 SSEs detected across all three strainmeters. Events are color-coded by depth, and symbol sizes scale with moment magnitude. **b** Slip directions inferred from forward modeling. The red line indicates the trace of the San Andreas Fault. Slip vectors are consistent with right-lateral motion along the fault, and their lengths are proportional to the modeled slip magnitude. The inset illustrates the expected right-lateral motion across the fault for reference. **c** Moment–duration relationship of different studies from Ide and Beroza (2023)^69^, shown as gray dots. The blue and red lines show the seismic moment and duration scaling for slow and regular earthquakes, respectively^45^. SSEs from this study are plotted as red dots. **d** Moment–duration relationship of the Parkfield SSEs, showing a cubic scaling trend (dashed line) consistent with that observed for regular earthquakes.

To investigate the physical characteristics of the short-term SSEs, we analyze 22 events coherently observed across all three strainmeters B073, B075, and B076 (see Fig. S9 for three example SSEs observed at the three stations). For the location analysis, we include all SSEs from the manual catalog, regardless of whether they were detected by our machine learning method, together with newly detected SSEs, and retain only those that are coherently observable at all three stations; events observable at only one or two stations are excluded, although all manual and newly detected SSEs have comparable durations in the 25–100 min range. We estimate each SSE’s location, slip, and seismic moment using forward modeling based on the Okada dislocation solution for a rectangular fault patch^63^. The predicted strain at each station is computed for unit strike-slip, forming the Green’s function kernel in the observation equation *ε*^*obs*^ = **G**.*s*. Here, *ε*^*obs*^ is the observed strain, **G** is the Green’s function matrix describing the strain response to unit slip, and *s* is the fault slip. Solving this in a least-squares sense yields the best-fitting scalar slip, *s*, for each candidate source (see method section). Constraining the geometry to the San Andreas Fault and assuming a pure strike-slip mechanism^64^, we find that these SSEs are shallow (depth <4 km), with slip directions consistent with right-lateral motion along the fault (Fig. 4a, b). Quantitatively, ~90% of the events are consistent with right-lateral motion. The strike-slip assumption constrains motion to be parallel to the fault plane, while the sense of slip (right- or left-lateral) is inferred from the inversion rather than imposed a priori. The majority of events, particularly those with larger slip magnitudes, exhibit right-lateral behavior, whereas some smaller-magnitude events show opposite slip directions. For smaller events, the sense of slip is weakly constrained due to limited station coverage and low signal amplitudes, and some inconsistencies with the expected right-lateral behavior likely reflect the limited observational coverage provided by only three stations, a limitation also noted by Gladwin et al. (1994) in the context of sparse spatial sampling. Misfit analyses (Figs. S10–S12) show that source depth and horizontal location are only weakly resolved with the three-station strainmeter geometry. Misfit curves typically exhibit broad low-misfit regions rather than sharply defined minima, indicating that multiple nearby models produce similar strain predictions. Accordingly, the inferred shallow depths should be interpreted as preferred solutions within the explored domain rather than uniquely constrained estimates. Independent surface creep observations further support the shallow character of the inferred slip. Slip amplitude is determined from the least-squares fit to the observed strain for each tested geometry. Although seismic moment depends on both slip and fault area, the moment-duration scaling does not rely on a precise determination of source location or depth. All results of the Okada dislocation model are provided in Supplementary Data 1.

We find that the short-term SSEs follow a cubic moment–duration scaling law (Fig. 4c, d), consistent with the scaling observed for regular earthquakes^65^. This contrasts with earlier studies that reported a linear moment–duration relationship for SSEs across diverse tectonic settings, particularly for longer-duration or deeper events^43,45,66^. Our results are consistent with recent studies suggesting earthquake-like scaling for SSEs^67,68^, although the interpretation of these results has been re-evaluated in light of limits in detectability^69^. Previous work^69^ argued that the linear scaling reflects a bound on moment rate, governed by the diffusional nature of slow earthquake propagation, and that the cubic scaling may apply in some cases, but that the linear scaling reflects a boundary of behavior. Our findings support this interpretation because they represent deformation transients that are significantly slower relative to their moment than previously observed (Fig. 4c, d).

The limited three-station strainmeter geometry strongly constrains the resolving power of the source inversion. Depth and horizontal location are weakly constrained and should be interpreted as approximate, preferred solutions rather than uniquely resolved parameters. This limitation also affects the estimation of source dimensions and, therefore, can introduce uncertainty in the interpretation of scaling relationships. Future work combining denser geodetic networks, additional independent observations (e.g., GPS or creepmeters), and improved constraints on fault geometry will be necessary to better resolve source properties and to more robustly assess the scaling behavior of short-term slow slip events.

### Low-frequency earthquakes increase following SSEs

**Fig. 5:**
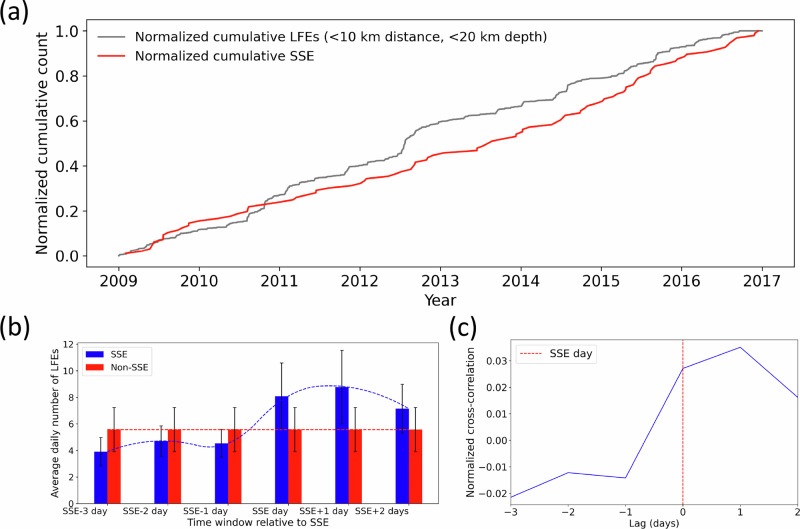
Temporal relationship between slow slip events (SSEs) and low-frequency earthquakes (LFEs). **a** Normalized cumulative counts of LFEs (within 10 km of station B073 and depth <20 km) and SSEs. **b** Mean daily LFE counts over the 6-day interval [−3, −2, −1, 0, +1, +2] relative to the SSE day (day 0), comparing periods with SSEs (blue) to randomly selected non-SSE periods (red). This comparison is used to assess whether LFE activity increases near SSEs. Error bars show the standard error of the mean daily LFE rate at each lag, indicating that the post-SSE increase is not driven by a few isolated high-count days but reflects a consistent ensemble trend. **c** Normalized correlation between daily LFE activity and SSE timing. Both time series were z-score (subtract the mean and divide by the standard deviation) normalized.

We observe a temporal correlation between short-duration slow slip events (SSEs) and increased low-frequency earthquakes (LFEs). Using the LFE catalog along the central San Andreas Fault^70^, we analyze eight years of catalog data from 2009 to 2016. As an initial step, we compare the cumulative number of LFEs with the cumulative number of SSEs (Fig. S13a). However, this comparison is challenging due to the significant difference in event frequency, approximately 500,000 LFEs (one million LFEs over 2001–2016 and ~500,000 LFEs within our study period)versus only 92 SSEs. Given that SSEs are short in duration and low in amplitude, we reduce the spatial extent of the LFE catalog to focus on potential local interactions and restrict the analysis to LFEs occurring within 10 km of our reference strainmeter station (Fig. S13b). This threshold aligns with the estimated spatial uncertainty of LFE family locations and the typical lateral extent of their detection footprints in the Parkfield area^70^, ensuring that only spatially coherent LFE-strainmeter interactions are considered (see Fig. 1b for LFE family locations and Fig. S13a for the cumulative plot). We also investigate whether the correlation changes when applying a depth constraint to the LFEs. LFE depths range between 16 and 28 km^70^. We plot the cumulative number of LFEs and SSEs separately for LFEs shallower and deeper than 20 km (Figs. 5a, S13c). The comparison shows a stronger temporal correlation between SSEs and LFEs shallower than 20 km. This range aligns with the lower bound of LFE depths in the catalog (~16 km) and is consistent with the ~16 km depth of SSEs previously identified using the GPS network in Parkfield^31^. We note, however, that the 20 SSEs reported in the previous study^31^ were not detected as individual events, but inferred indirectly by stacking GPS time series using the timing of LFE bursts from Shelly (2017). That study did not publish a catalog of individual SSE times, and the timing information is only available in graphical form as approximate central times of LFE bursts. The authors also explicitly reported that corresponding strainmeter signals could not be resolved due to noise at multi-day periods. As a result, our study and that of Rousset et al. (2019)^31^ address different observational expressions of slow slip and are not directly comparable on an event-by-event basis.

Better correlation with shallower LFEs could indicate a possible connection between the shallow upper few km where our SSEs are happening, and the base of the seismogenic zone, where the shallow LFEs are happening. The normalized cumulative counts of LFEs within 10 km of station B073 and shallower than 20 km (a total of approximately 15,000 LFEs in this range), along with the normalized cumulative counts of SSEs, are shown in Fig. 5a. We also compared the cumulative number of SSEs with that of earthquakes (Figure S14) and tremors (Fig. S15) at different distances from the reference strainmeter in the central San Andreas Fault region. No correlation is observed between SSEs and tremors, while a possible weak correlation appears for earthquakes, though it is much less clear than the correlation with LFEs.

The temporal correlation between SSEs and LFEs is apparent in the cumulative plot. To quantify this relationship, we computed the normalized correlation between daily LFE counts and SSE occurrences (Fig. 5c). For a 6-day window covering 3 days before and 2 days after SSE, we observe a distinct peak in correlation on the day following the SSE (SSE + 1). Because the SSE series contains only 92 non-zero days over the 8-year period, while LFE daily counts are much larger and highly variable, reaching up to 157 events/day, the normalized cross-correlation is expected to have small absolute amplitudes. In this context, the interpretation relies on the timing of the peak rather than the coefficient magnitude, and the maximum near SSE day indicates enhanced LFE activity immediately following SSE onset. To evaluate whether LFE activity is elevated around SSEs, we compute the mean daily LFE counts over a 6-day window centered on each SSE (from 3 days before to 2 days after) and compare them with similar windows during randomly selected non-SSE periods. Non-SSE reference windows were randomly selected from periods without SSEs and were required to lie outside the −3 to +2 day neighborhood of any SSE to avoid overlap with pre- and post-SSE intervals. This allows us to identify short-term temporal correlations between LFEs and SSEs. (Fig. 5b). LFE rates are higher during an SSE, on the day of the event, and the two subsequent days, implying that SSEs may influence the timing and occurrence of nearby LFEs, potentially by modifying local stress. Previous studies have also reported a temporal relationship between SSEs and tremor^40^, which led to the definition of Episodic Tremor and Slip (ETS) in subduction zones. Tremor has been shown to consist of sequences of low-frequency earthquakes (LFEs) in some regions, and several studies have further established the correspondence between SSEs and LFEs^44,51,71^, suggesting a complex interaction between aseismic and seismic slip. To assess the robustness of the increased LFE rates after SSEs, we repeated the analysis first using only days on which LFEs occurred (excluding days with zero LFEs) and second, using median daily LFE counts, which are less affected by outlier days. Figure S16a shows that restricting the analysis to LFE-active days yields the same qualitative pattern as Fig. 5b, with elevated LFE activity after SSEs. Figure S16b presents the median-based results, confirming that the observed post-SSE increase is not driven by a small number of extreme LFE days. Additionally, Supplementary Table 1 reports variability measures (mean, standard deviation, and interquartile range) of LFE/day rates, all of which support the conclusion that LFE activity systematically increases following SSEs.

Many previous studies examining the relationship between SSEs and LFEs have relied on inferred SSE timing from LFE activity^41,72^ since the timing of LFE can provide constraints on the dynamics of SSE,^45,50,68^ rather than direct detection of individual SSEs. In contrast, our study uses a systematically constructed catalog of SSEs, allowing for a direct and time-resolved comparison with the existing LFE catalog. Our approach provides more robust evidence for a temporal and potentially causal link between SSEs and LFE activity in fault zones.

### Stress transfer between the creeping section and the transition zone

Understanding the slip dynamics and transient deformation within the creeping section can inform how aseismic slip can modulate stress accumulation and release. We observe a temporal modulation of low-frequency seismicity associated with SSEs, implying a transient transfer of stress from the creeping section to adjacent fault zones, particularly in the transition zone between the creeping and locked segments.

Our analysis shows that the SSEs are shallow and kinematically consistent with the right-lateral motion of the San Andreas Fault, and with their surface manifestation known as creep events. Aseismic fault creep was first documented on the San Andreas Fault at Cienega Winery^73^. In addition to steady aseismic slip, accelerated episodes of surface fault creep, referred to as creep events, were subsequently discovered on the San Andreas Fault^74,75^. Although measured at the surface, creep events were subsequently recorded on borehole strain meters on the San Andreas Fault near San Juan Bautista and found to extend to depths of up to several km, with surface slip found to lag slip at depth^29^. With the discovery of slow slip events in subduction zones, which also represent aseismic shear slip on faults, creep events might better be characterized as short-term slow slip events.

Their moment–duration scaling follows a cubic law, similar to regular earthquakes. We note that in dynamic earthquake models, it is the elastodynamic stress concentration ahead of the rupture front that sustains rupture. In those models, the rupture velocity is comparable to the seismic wave speed. Ide and Beroza (2023)^69^ suggest that elastodynamic rupture leads to scaling where both the duration and the length of rupture grow linearly with time, which leads to moment scaling with the cube of time if the stress drop is constant. For slow slip events, the cubic scaling with duration must have another origin; however, given the limited resolution of source dimensions, particularly depth and fault area, this scaling should be interpreted with caution. In particular, uncertainties in the size of the slipping area may affect the inferred relationship. Nevertheless, these quantifications provide a physical bridge between slow and fast fault slip recorded in seismic and geodetic data, reinforcing the idea that fault deformation occurs across a continuum of transient behaviors.

In this work, we show that deep learning applied to continuous strainmeter data detects short-duration slow slip events on the creeping section of the San Andreas Fault near Parkfield. We identify coherent SSE signals across multiple instruments, with supporting observations from creepmeters, and find that these events are associated with increased low-frequency earthquake activity following slip. Source analysis indicates shallow slip consistent with right-lateral motion on the San Andreas Fault, although source geometry remains only weakly constrained by the available three stations. The detected events follow an approximate cubic moment–duration scaling relationship, consistent with that of earthquakes. Our observations underscore the importance of including slow slip dynamics, particularly in creeping fault segments, when modeling stress transfer and fault interaction. Such processes may influence stress loading in adjacent locked zones and therefore have implications for seismic hazard assessment.

## Methods

To obtain high-resolution data and capture short-duration slow slip events, we download strainmeter data sampled at 1 Hz (the highest available sampling rate) from IRIS/EarthScope. We consider data spanning 8 years, from 2009 to 2016, consistent with the time period covered by a manual catalog of shallow aseismic slip events^57^. This catalog was constructed by visually inspecting the differential and engineering strain channels of B073, located 1.1 km from the San Andreas Fault (Fig. 1), for their characteristic short-duration, step-like transients using 4-hour analysis windows. When available, coincident offsets on nearby creepmeters were used as supporting evidence for the shallow aseismic origin of a candidate event. These criteria define the set of short-duration SSEs that we use to validate the automatic detections from our machine-learning workflow. In addition to this reference station, we analyzed two other stations: B075, located on the opposite (west) side of the fault at a distance of 2.9 km, and B076, located on the same side as B073 at a distance of 3.4 km. Both stations are approximately 4 km from B073. The nearest station to B073 beyond these is located 17 km away, which is probably too distant to detect the small, slow slip events observed at B073.

Our approach consists of four main stages: (1) preprocessing borehole strainmeter data, (2) transforming the data into a representation that highlights slow slip events (SSEs) using wavelet analysis, (3) feature extraction and dimensionality reduction using AutoencoderZ, and (4) clustering for event detection. We describe these steps in the following.

### Preprocessing of Strainmeter data

Strain records often contain unwanted signals that can obscure the detection of SSEs. These include long-term borehole curing trends, tidal effects, barometric pressure variations, rainfall-induced changes, and abrupt offsets that occasionally appear on individual gauges without a known cause. We use EarthScopeStrainTools^33^ for all preprocessing steps (Fig. S17). In the following, we describe the preprocessing steps:

First, we download the raw data and convert it into strain units (see Fig. S17). We then detrended the data by evaluating the trend over the relevant timeframe, and remove a simple linear trend from the long-term borehole relaxation signal. Next, we correct the data for tidal effects. BAYTAP08^33,76,77^ is a Bayesian modeling procedure that determines the tidal amplitudes and phases for several of the largest tidal constituents (M2, O1, P1, K1, N2, S2). These constituents are then used in SPOTL to generate a forward model time series of tides for each gauge, which can then be subtracted from the data. We also calculated an atmospheric pressure correction coefficient for each gauge in BAYTAP08, similar to the tidal constituents. This coefficient is used with the colocated surface barometric pressure sensor data to create a pressure correction time series, and the data were corrected accordingly for the effects of atmospheric pressure variations. The EarthScope documentation recommends applying an offset correction after the previous three corrections. Offsets are automatically detected using a simple first-differencing algorithm. After comparing the data with and without offset correction for SSE detection, we found that applying the offset correction over the entire analysis period sometimes altered the signal shape during the SSE (Fig. S18), preventing its detection. Therefore, we decided not to apply the offset correction for SSE detection.

The strain recorded by each gauge reflects the combined response of the instrument, grout, and bedrock to strain; however, we are concerned with the strain of the rock formation itself. To obtain this, a calibration (or orientation) matrix is applied to the four gauge strains, transforming the recorded data into areal (*ε*_*xx*_ + *ε*_*γγ*_), differential shear (*ε*_*xx*_ − *ε*_*γγ*_), and engineering shear strains (2*ε*_*xγ*_) in the East/North reference system, where εᵢ_ⱼ_ denotes the components of the horizontal strain tensor (with i, j = x or y). After correcting the gauge strain and converting it to regional strain via calibration, the data are ready for analysis.

### Wavelet transform for event representation

SSEs produce a step-like jump in the strainmeter data. To develop an algorithm for their automatic detection, we require a data representation that enhances the visibility of SSEs. First, we tested the Short-Time Fourier Transform (STFT), but it did not reveal distinct patterns at the time of the SSE (Fig S19). We found that the wavelet transform (WT), using the Morlet wavelet basis, provides an effective representation of the event because it offers a good balance between time and frequency localization, making it suitable for detecting transient signals such as SSEs. The duration of the signals captured depends on the wavelet scale and the sampling rate. Since our data is sampled at 1 Hz, the duration of the signal captured at each wavelet scale approximately corresponds to the scale itself. For a Morlet wavelet with central frequency *ω*_0_ = 6 the relationship between the scale *s* and the approximate period *T* is given by:

1 $$T\approx 1.25\times {{\rm{s}}}$$

The factor 1.25 comes from the inverse of the Morlet wavelet’s central frequency^78^ and $s$ denotes the wavelet scale. Because our strainmeter data are sampled at 1 Hz, the maximum period captured by our largest scale $s=2048\ {is}:$

2 $${T}_{\max }\approx 1.25\times 2048\approx 2560\,{{\rm{s}}}\approx 43\,\min$$

Thus, with the five scales used in this study (evenly spaced between 1 and 2048), the WT is intrinsically sensitive to signal durations ranging from approximately 1 second to ~45 minutes. SSEs are primarily captured at high wavelet scales (i.e., lower frequencies and longer durations), while smaller-scale coefficients capture high-frequency strainmeter variations. With this setting, SSEs appear as localized, high-energy signals, making them clearly distinguishable from other parts of the signal.

Although the WT’s formal maximum resolvable period is ~40 minutes, events with rise times up to ~120–150 minutes still produce detectable energy within our wavelet scales. A step-like transient with a multi-hour rise is not a pure low-frequency signal: its onset contains finite slope and curvature, and these local gradients project energy onto shorter periods (approximately 10–40 minutes), which lie within the sensitive band of the Morlet Continuous WT. As the rise time increases, the transient becomes progressively smoother, reducing its curvature and thereby suppressing the higher-frequency components that the WT can detect. For very slow rises on the order of 3–4 hours or longer, the signal’s spectral energy shifts almost entirely to periods beyond the WT’s maximum scale, rendering such transients indistinguishable from background low-frequency variability under our chosen settings. Supplementary Note 2 illustrates this behavior using synthetic step-like transients, showing how their visibility in the WT progressively diminishes with increasing event duration when using our specific WT configuration (Morlet wavelet, scales 1–2048, equivalent to periods of ~1 *s* to ~40 min and sensitive to transients from 1 s to 150 min).

For the input to the machine learning model, we create daily WTs, with each day consisting of 86,400 samples. To reduce the input size for efficient training, we downsample the WT by a factor of 10. This does not affect the resolution of our detection, as the WT is created from the 1 Hz data, and the downsampling is applied to the already-transformed data, preserving the necessary details. In the final step, we normalize the data using its mean and standard deviation. This ensures that all input features have a similar scale, improving the stability and performance of the machine learning model. The data is now ready for use as the model input.

### Feature extraction and dimensionality reduction with AutoencoderZ

To detect SSEs automatically in strainmeter data, we employ a clustering approach that aims to separate them into a distinct cluster. Clustering is an unsupervised learning approach that groups similar data points without relying on predefined labels or examples. Unsupervised frameworks have been applied successfully in seismology to detect and cluster seismic signals^79^. However, as data dimensionality increases, the performance of conventional clustering methods decreases. To address this, we apply feature extraction and dimensionality reduction to transform the input data into a lower-dimensional space before clustering. This is achieved using AutoencoderZ, a deep learning-based autoencoder model developed in our previous studies^55,56^. This model has been proven to perform exceptionally well across various data types^80^ and durations^56^. The lower-dimensional representation serves as the input for the clustering step, where SSEs are grouped separately from other signals. We train the model separately for each station and each component. The autoencoder was trained in an unsupervised manner using 80% of the data for training and 20% for validation. During training, the AutoencoderZ model minimizes a reconstruction loss that quantifies the difference between the input and its reconstruction. We monitor both training and validation losses to ensure the model generalizes well and does not overfit. Figure S20 shows the reconstruction loss curves for the station B073 differential component as an example, illustrating the training and validation behavior.

Among the three strains (areal, differential, and engineering), tectonic movements are primarily reflected in differential and engineering strains, while changes in the areal strain are more sensitive to vertical strain changes, such as those from barometric pressure variations and anthropogenic noise. For this reason, we use only differential and engineering strains as the input for the model to detect SSEs.

### Clustering for event detection

We use the extracted features for the clustering task. During the training process, the autoencoder learns to reconstruct the output from the latent space to closely match the input. Once trained, the features extracted from the autoencoder’s bottleneck are used for the clustering task, which is performed using the K-means algorithm. K-means is a fast and commonly applied clustering technique. The algorithm partitions the latent space into clusters, each represented by a cluster centroid. This centroid, which marks the center of a cluster, is the mean of all data points within that specific cluster. K-means clustering was performed separately for each station and strain component to account for differences in signal characteristics, and multiple random initializations (n_init = 20) were used to ensure stable clustering results.

We use the Silhouette score to define the number of clusters. The Silhouette score measures how similar an object is to its own cluster compared to other clusters, with values ranging from −1 to 1. A higher score indicates better-defined and well-separated clusters. The optimal number of clusters is determined by maximizing the average Silhouette score, with the highest score indicating the best clustering configuration.

In this work, we use a two-step clustering approach. First, we apply clustering, and based on the Silhouette score, and find that the optimal number of clusters is two. Upon examining the data in the two clusters, we observe that one cluster contains data with a spike-like pattern in the Wavelet Transform (WT), while the other cluster includes samples (each representing one day) that show a smoother progression without sudden changes. The first cluster includes SSE events that exhibit spike-like patterns in the WT, but also contains other transient changes, such as earthquakes. To filter out non-SSE events from the first cluster, we perform a second clustering on the data within it. This step effectively separates many samples that do not contain SSEs but include other transient signals. Again, the optimal number of clusters in this step, based on the Silhouette score, is two. We designate one of these clusters as the SSE cluster, as it mainly contains SSE events. The other cluster, with less sharp spike patterns in the WT, is associated with other transient signals, which are not the focus of this study. The autoencoder was trained using standard training and validation splits to ensure stable feature extraction, while the subsequent clustering step is fully unsupervised and does not rely on labeled targets. The choice of two clusters and separate clustering for each station and strain component reflects the distinct signal characteristics of SSEs, which produce step-like transient strain changes that are clearly separable from background noise. Further validation on additional years and regions will be valuable to assess its broader applicability.

### Source location and scaling analysis

To estimate the source properties of short-duration SSEs, we implemented a forward modeling framework based on the Okada solution for rectangular fault dislocations in a homogeneous elastic half-space^63^. We performed a grid search over six fault parameters: horizontal location (x, y), depth (z), strike, dip, and fault length. We related the fault width from the length using an empirical scaling relation *W* = *C*_1_ *L*^*β*^ with *C*_*1*_ = 15 and *β* = 2/3, consistent with empirical constraints for width-limited strike-slip faults^81^.

We modeled slip as pure strike-slip, consistent with the kinematics of the San Andreas Fault^64^. For each trial configuration, we assumed a unit strike-slip displacement (1 m) and used the Okada solution to compute the predicted strain tensor at the three strainmeter stations. This defines the Green’s function matrix ***G*** (i.e., influence coefficients), which quantifies the elastic strain response per unit slip. We then estimated the scalar slip amplitude *s* that best fits the observed strain *ε*^*obs*^ using the linear observation equation:

3 $${\varepsilon }^{{obs}}={{\bf{G}}}\,s$$

The best-fitting slip was obtained using a least-squares solution:

4 $$s=\frac{{{{\bf{G}}}}^{T}\,{\varepsilon }^{{obs}}}{{{{\bf{G}}}}^{T}{{\bf{G}}}}$$

The forward strain predictions were then scaled by this slip value and compared to the observed strain. We defined the optimal model as the configuration that minimized the misfit, defined as the sum of squared differences between observed and predicted strain:

5 $${{||}{\varepsilon }^{{obs}}-s{{\bf{G}}}{||}}^{2}$$

The search domain for the grid search was constrained based on physical and geological considerations. The horizontal coordinates (x, y) were allowed to vary within ±3 km from the barycenter of the strainmeter network. Depth values ranged from 500 meters to 4 km. These ranges were chosen based on preliminary inversions indicating that misfit minima occur at shallow depths and near the network center, with little improvement in fit at larger distances or greater depths (Figs. S10–S12). Strike and dip were constrained to 310°–330° and 60°–80°, respectively, consistent with the local geometry of the San Andreas Fault. Fault length was varied between 0.5 and 4 km.

Because the inversion relies on static strain from only three stations, the misfit surfaces commonly exhibit broad low-misfit regions rather than sharply defined minima, indicating limited resolution of source depth and horizontal location. For the best-fitting model, we extracted the source location, geometry, and slip amplitude, and used these to compute the seismic moment as:

6 $${{{\rm{M}}}}_{0}=\mu {As}$$

Where *μ* is the shear modulus (assumed 30 GPa), *A* is the fault patch area (length × width), and *s* is the strike-slip displacement across the fault^82^. We then calculated the corresponding moment magnitude using the standard relation^83^:

7 $${{{\rm{M}}}}_{{{\rm{w}}}}=\frac{2}{3}({log }_{10}{{{\rm{M}}}}_{0}-9.1)$$

While fault length is explored during the grid search, the limited three-station geometry provides only weak constraints on source dimensions. Slip and seismic moment are better resolved than fault length or depth, and we therefore focus the scaling analysis on moment–duration relationships. To investigate the scaling behavior of SSEs, we used these moment estimates along with manually picked start and end times for the 22 selected events to calculate their durations. The resulting moment and duration values were used to construct a moment–duration scaling relationship (Fig. 4c, d).

To visualize the slip direction of the modeled SSEs, we assumed pure strike-slip motion and used the strike angle and sign of the slip from each best-fitting model. The slip azimuth was taken to be equal to the fault strike, while the sign of the modeled slip determined the polarity of motion along that direction. East–north vector components were calculated from the strike and normalized slip amplitude as *u = slip × cos(strike)* and *v = slip × sin(strike)*, and plotted as directional arrows at each event location. The inferred slip directions are mostly aligned with right-lateral motion along the San Andreas Fault, consistent with its known tectonic setting.

## Supplementary information

Supplementary Information

Description of Additional Supplementary File

Supplementary Data 1- SSE_Catalog_Parkfield

Supplementary_Data_2_SSE_Figures

Transparent Peer Review file

## Data Availability

All strainmeter data used in this study were accessed from the NSF NGF data archive operated by EarthScope Consortium (NSF award 2435260) and are publicly available at: https://www.unavco.org/data/strain-seismic/bsm-data/bsm-data.html Creep-meter data are publicly available through the USGS deformation monitoring database at: https://earthquake.usgs.gov/monitoring/deformation/data/download.php Supplementary Data 1, an Excel file, contains the complete catalog of 92 SSEs introduced in this study, with newly detected SSEs highlighted relative to those previously reported in the manual catalog. It also includes a subset of 22 SSEs observed at all three strainmeter stations and used for the Okada dislocation modeling, with information on amplitude changes at the three stations, the associated creepmeters where slip is observed, and the corresponding model results, including SSE location, depth, magnitude, and duration. In addition, Supplementary Data 2 provides the corrected strainmeter signals, wavelet transform figures, and the traces of the three nearby creepmeters for all SSEs in the catalog.

## Code availability

The code for preprocessing strainmeter data and preparing it for analysis, as well as the deep learning algorithm used for detecting slow slip events, is available at the following GitHub repositories: https://github.com/ZahraZali/DeepStrain^84^ and https://github.com/ZahraZali/AutoencoderZ^85^. The EarthScope tools used for strainmeter data preprocessing are publicly available from Mikegottlieb84 & Cehanagan (2024) at: https://github.com/EarthScope/earthscopestraintools/tree/V0.1.42-beta. For the Okada dislocation modeling in Python, we used the okada_wrapper available at: https://github.com/cutde-org/okada_wrapper/tree/master.

## References

[1] Beroza, G. C. & Ide, S. Slow earthquakes and nonvolcanic tremor. *Annu. Rev. Earth Planet. Sci.* **39**, 271–296 (2011).

[2] Jolivet, R. & Frank, W. B. The transient and intermittent nature of slow slip. *AGU Adv.* **1**, e2019AV000126 (2020).

[3] Dragert, H., Wang, K. & James, T. S. A silent slip event on the deeper cascadia subduction interface. *Science* **292**, 1525–1528 (2001). 11313500 10.1126/science.1060152

[4] Radiguet, M. et al. Slow slip events and strain accumulation in the Guerrero gap, Mexico. *J. Geophys. Res.* **117**, B04305 (2012).

[5] Chen, K. H. & Bürgmann, R. Creeping faults: Good news, bad news? *Rev. Geophys.* **55**, 282–286 (2017).

[6] Harris, R. A. Large earthquakes and creeping faults. *Rev. Geophys.* **55**, 169–198 (2017).

[7] Kato, A., Fukuda, J., Nakagawa, S. & Obara, K. Foreshock migration preceding the 2016 Mw 7.0 Kumamoto earthquake, Japan. *Geophys. Res. Lett.* **43**, 8945–8953 (2016).

[8] Uchida, N., Iinuma, T., Nadeau, R. M., Bürgmann, R. & Hino, R. Periodic slow slip triggers megathrust zone earthquakes in northeastern Japan. *Science* **351**, 488–492 (2016). 26823425 10.1126/science.aad3108

[9] Wei, S., Graves, R., Helmberger, D., Avouac, J.-P. & Jiang, J. Sources of shaking and flooding during the Tohoku-Oki earthquake: A mixture of rupture styles. *Earth Planet. Sci. Lett.* **333–334**, 91–100 (2012).

[10] Hsu, Y.-J., Segall, P., Yu, S.-B., Kuo, L.-C. & Williams, C. A. Temporal and spatial variations of post-seismic deformation following the 1999 Chi-Chi, Taiwan earthquake. *Geophys. J. Int.* **169**, 367–379 (2007).

[11] Ozawa, S. et al. Coseismic and postseismic slip of the 2011 magnitude-9 Tohoku-Oki earthquake. *Nature* **475**, 373–376 (2011). 21677648 10.1038/nature10227

[12] Alwahedi, M. A. & Hawthorne, J. C. Intermediate-magnitude postseismic slip follows intermediate-magnitude (M 4 to 5) earthquakes in California. *Geophys. Res. Lett.* **46**, 3676–3687 (2019).

[13] Martínez-Garzón, P. et al. Slow strain release along the eastern Marmara region offshore Istanbul in conjunction with enhanced local seismic moment release. *Earth Planet. Sci. Lett.* **510**, 209–218 (2019).

[14] Segall, P., Desmarais, E. K., Shelly, D., Miklius, A. & Cervelli, P. Earthquakes triggered by silent slip events on Kīlauea volcano, Hawaii. *Nature* **442**, 71–74 (2006). 16823451 10.1038/nature04938

[15] Kato, A. et al. Propagation of Slow Slip Leading Up to the 2011 Mw 9.0 Tohoku-Oki Earthquake. *Science* **335**, 705–708 (2012). 22267578 10.1126/science.1215141

[16] Khoshmanesh, M., Shirzaei, M. & Uchida, N. Deep slow-slip events promote seismicity in northeastern Japan megathrust. *Earth Planet. Sci. Lett.* **540**, 116261 (2020).

[17] Obara, K. & Kato, A. Connecting slow earthquakes to huge earthquakes. *Science* **353**, 253–257 (2016). 27418504 10.1126/science.aaf1512

[18] Bürgmann, R. The geophysics, geology and mechanics of slow fault slip. *Earth Planet. Sci. Lett.* **495**, 112–134 (2018).

[19] Rubin, A. M. & Ampuero, J.-P. Earthquake nucleation on (aging) rate and state faults. *J. Geophys. Res.* **110**, B11312 (2005).

[20] Rubin, A. M. Episodic slow slip events and rate-and-state friction. *J. Geophys. Res.* **113**, B11414 (2008).

[21] Kaduri, M., Gratier, J.-P., Lasserre, C., Çakir, Z. & Renard, F. Quantifying the partition between seismic and aseismic deformation along creeping and locked sections of the North Anatolian Fault, Turkey. *Pure Appl. Geophys.* **176**, 1293–1321 (2019).

[22] Romanet, P., Bhat, H. S., Jolivet, R. & Madariaga, R. Fast and slow slip events emerge due to fault geometrical complexity. *Geophys. Res. Lett.* **45**, 4809–4819 (2018).

[23] Murray, J. R. & Segall, P. Spatiotemporal evolution of a transient slip event on the San Andreas fault near Parkfield, California. *J. Geophys. Res. Solid Earth* **110**, B09407 (2005).

[24] Wech, A. G. & Creager, K. C. A continuum of stress, strength and slip in the Cascadia subduction zone. *Nat. Geosci.* **4**, 624–628 (2011).

[25] Obara, K. Characteristics and interactions between non-volcanic tremor and related slow earthquakes in the Nankai subduction zone, southwest Japan. *J. Geodyn.* **52**, 229–248 (2011).

[26] Shelly, D. R., Beroza, G. C., Ide, S. & Nakamula, S. Low-frequency earthquakes in Shikoku, Japan, and their relationship to episodic tremor and slip. *Nature* **442**, 188–191 (2006). 16838019 10.1038/nature04931

[27] Audet, P. & Bürgmann, R. Possible control of subduction zone slow-earthquake periodicity by silica enrichment. *Nature* **510**, 389–392 (2014). 24943955 10.1038/nature13391

[28] Scholz, C. H. Earthquakes and friction laws. *Nature* **391**, 37–42 (1998).

[29] Gladwin, M. T., Gwyther, R. L., Hart, R. H. G. & Breckenridge, K. S. Measurements of the strain field associated with episodic creep events on the San Andreas Fault at San Juan Bautista, California. *J. Geophys. Res. Solid Earth* **99**, 4559–4565 (1994).

[30] Linde, A. T., Gladwin, M. T., Johnston, M. J. S., Gwyther, R. L. & Bilham, R. G. A slow earthquake sequence on the San Andreas fault. *Nature* **383**, 65–68 (1996).

[31] Rousset, B., Bürgmann, R. & Campillo, M. Slow slip events in the roots of the San Andreas fault. *Sci. Adv.* **5**, eaav3274 (2019). 30788438 10.1126/sciadv.aav3274 PMC6374109

[32] Agnew, D. C. Strainmeters and tiltmeters. *Rev. Geophys.* **24**, 579–624 (1986).

[33] Hodgkinson, K., Langbein, J., Henderson, B., Mencin, D. & Borsa, A. Tidal calibration of plate boundary observatory borehole strainmeters. *J. Geophys. Res. Solid Earth* **118**, 447–458 (2013).

[34] Martínez-Garzón, P. et al. Near-fault monitoring reveals combined seismic and slow activation of a fault branch within the Istanbul–Marmara Seismic Gap in Northwest Turkey. *Seismol. Res. Lett.* **92**, 3743–3756 (2021).

[35] Smith, E. F. & Gomberg, J. A search in strainmeter data for slow slip associated with triggered and ambient tremor near Parkfield, California. *J. Geophys. Res. Solid Earth* **114**, B00A03 (2009).

[36] Gittins, D. B. & Hawthorne, J. C. Scattered M3–4 Slip Bursts Within Creep Events on the San Andreas Fault. *J. Geophys. Res. Solid Earth* **129**, e2023JB028187 (2024).

[37] Chiaraluce, L. et al. A strainmeter array as the fulcrum of novel observatory sites along the Alto Tiberina Near Fault Observatory. *Sci. Drill.* **33**, 173–190 (2024).

[38] Materna, K. et al. Shallow slow slip events in the Imperial Valley with along-strike propagation. *Geophys. Res. Lett.* **51**, e2023GL108089 (2024).

[39] Ross, Z. E., Cochran, E. S., Trugman, D. T. & Smith, J. D. 3D fault architecture controls the dynamism of earthquake swarms. *Science* **368**, 1357–1361 (2020). 32554593 10.1126/science.abb0779

[40] Rogers, G. & Dragert, H. Episodic Tremor and Slip on the Cascadia Subduction Zone: The Chatter of Silent Slip. *Science* **300**, 1942–1943 (2003). 12738870 10.1126/science.1084783

[41] Frank, W. B. et al. Uncovering the geodetic signature of silent slip through repeating earthquakes. *Geophys. Res. Lett.* **42**, 2774–2779 (2015).

[42] Shelly, D. R. & Hardebeck, J. L. Precise tremor source locations and amplitude variations along the lower-crustal central San Andreas Fault. *Geophys. Res. Lett*. **37**, L14301 (2010).

[43] Peng, Z. & Gomberg, J. An integrated perspective of the continuum between earthquakes and slow-slip phenomena. *Nat. Geosci.* **3**, 599–607 (2010).

[44] Shelly, D. R., Beroza, G. C. & Ide, S. Non-volcanic tremor and low-frequency earthquake swarms. *Nature* **446**, 305–307 (2007). 17361180 10.1038/nature05666

[45] Ide, S., Beroza, G. C., Shelly, D. R. & Uchide, T. A scaling law for slow earthquakes. *Nature* **447**, 76–79 (2007). 17476265 10.1038/nature05780

[46] Liu, C. et al. Rupture processes of the 2012 September 5 Mw 7.6 Nicoya, Costa Rica earthquake constrained by improved geodetic and seismological observations. *Geophys. J. Int.* **203**, 175–183 (2015).

[47] Aden-Antoniów, F. et al. Low-frequency earthquakes downdip of deep slow slip beneath the north island of New Zealand. *J. Geophys. Res. Solid Earth* **129**, e2023JB027971 (2024).

[48] Wech, A. G., Creager, K. C. & Melbourne, T. I. Seismic and geodetic constraints on Cascadia slow slip. *J. Geophys. Res. Solid Earth* **114**, B10316 (2009).

[49] Frank, W. B. et al. The evolving interaction of low-frequency earthquakes during transient slip. *Sci. Adv.* **2**, e1501616 (2016). 27152345 10.1126/sciadv.1501616 PMC4846440

[50] Lengliné, O., Frank, W. B., Marsan, D. & Ampuero, J.-P. Imbricated slip rate processes during slow slip transients imaged by low-frequency earthquakes. *Earth Planet. Sci. Lett.* **476**, 122–131 (2017).

[51] Nakano, M., Hori, T., Araki, E., Kodaira, S. & Ide, S. Shallow very-low-frequency earthquakes accompany slow slip events in the Nankai subduction zone. *Nat. Commun.* **9**, 984 (2018). 29540688 10.1038/s41467-018-03431-5 PMC5852141

[52] Lohman, R. B. & McGuire, J. J. Earthquake swarms driven by aseismic creep in the Salton Trough, California. *J. Geophys. Res. Solid Earth* **112**, B04405 (2007).

[53] Sirorattanakul, K. et al. The 2020 Westmorland, California earthquake swarm as aftershocks of a slow slip event sustained by fluid flow. *J. Geophys. Res. Solid Earth* **127**, e2022JB024693 (2022).

[54] Vavra, E. J. et al. Characteristic slow-slip events on the Superstition Hills fault, southern California. *Geophys. Res. Lett.* **51**, e2023GL107244 (2024).

[55] Zali, Z., Mousavi, S. M., Ohrnberger, M., Eibl, E. P. S. & Cotton, F. Tremor clustering reveals pre-eruptive signals and evolution of the 2021 Geldingadalir eruption of the Fagradalsfjall Fires, Iceland. *Commun. Earth Environ.* **5**, 1 (2024).

[56] Zali, Z. et al. Low-frequency tremor-like episodes before the 2023 MW 7.8 Türkiye earthquake linked to cement quarrying. *Sci. Rep.* **15**, 6354 (2025). 39984511 10.1038/s41598-025-88381-x PMC11845488

[57] Mencin, D. Periodic and Static Strain Investigations with Borehole Strainmeters and GPS. PhD thesis, University of Colorado Boulder https://scholar.colorado.edu/concern/graduate_thesis_or_dissertations/xd07gs87r (2018).

[58] Barbour, A. J. & Agnew, D. C. Noise levels on plate boundary observatory borehole strainmeters in southern California. *Bull. Seismol. Soc. Am.* **101**, 2453–2466 (2011).

[59] Yabe, S. et al. Eight-year catalog of deep short-term slow slip events at the Nankai trough based on objective detection algorithm using strain and tilt records. *Earth Planets Space* **75**, 13 (2023).

[60] Costantino, G. et al. Multi-station deep learning on geodetic time series detects slow slip events in Cascadia. *Commun. Earth Environ.* **4**, 435 (2023).

[61] Zali, Z., Ohrnberger, M., Scherbaum, F., Cotton, F. & Eibl, E. P. S. Volcanic tremor extraction and earthquake detection using music information retrieval algorithms. *Seismol. Res. Lett.* **92**, 3668–3681 (2021).

[62] Zali, Z., Rein, T., Krüger, F., Ohrnberger, M. & Scherbaum, F. Ocean bottom seismometer (OBS) noise reduction from horizontal and vertical components using harmonic–percussive separation algorithms. *Solid Earth* **14**, 181–195 (2023).

[63] Okada, Y. Surface deformation due to shear and tensile faults in a half-space. *Bull. Seismol. Soc. Am.* **75**, 1135–1154 (1985).

[64] Zoback, M. D. et al. New Evidence on the State of Stress of the San Andreas Fault System. *Science* **238**, 1105–1111 (1987). 17839366 10.1126/science.238.4830.1105

[65] Kanamori, H. & Anderson, D. L. Amplitude of the Earth’s free oscillations and long-period characteristics of the earthquake source. *J. Geophys. Res.* **80**, 1075–1078 (1975).

[66] Gao, H., Schmidt, D. A. & Weldon, R. J. II Scaling relationships of source parameters for slow slip events. *Bull. Seismol. Soc. Am.* **102**, 352–360 (2012).

[67] Michel, S., Gualandi, A. & Avouac, J.-P. Similar scaling laws for earthquakes and Cascadia slow-slip events. *Nature* **574**, 522–526 (2019). 31645722 10.1038/s41586-019-1673-6

[68] Frank, W. B. & Brodsky, E. E. Daily measurement of slow slip from low-frequency earthquakes is consistent with ordinary earthquake scaling. *Sci. Adv.* **5**, eaaw9386 (2019). 31616786 10.1126/sciadv.aaw9386 PMC6774729

[69] Ide, S. & Beroza, G. C. Slow earthquake scaling reconsidered as a boundary between distinct modes of rupture propagation. *Proc. Natl. Acad. Sci.* **120**, e2222102120 (2023). 37523541 10.1073/pnas.2222102120 PMC10410734

[70] Shelly, D. R. A 15 year catalog of more than 1 million low-frequency earthquakes: Tracking tremor and slip along the deep San Andreas Fault. *J. Geophys. Res. Solid Earth* **122**, 3739–3753 (2017).

[71] Bletery, Q. & Nocquet, J.-M. Slip bursts during coalescence of slow slip events in Cascadia. *Nat. Commun.* **11**, 2159 (2020). 32358488 10.1038/s41467-020-15494-4 PMC7195424

[72] Mouchon, C., Frank, W. B., Radiguet, M., Poli, P. & Cotte, N. Subdaily slow fault slip dynamics captured by low-frequency earthquakes. *AGU Adv.* **4**, e2022AV000848 (2023).

[73] Tocher, D. The Alaska earthquake of July 10, 1958: Movement on the Fairweather fault and field investigation of southern epicentral region. *Bull. Seismol. Soc. Am.* **50**, 267–292 (1960).

[74] Yamashita, P. A. & Burford, R. D. *Catalog of Preliminary Results from an 18-Station Creepmeter Network along the San Andreas Fault System in Central California for the Time Interval June 1969 to June 1973*. *USGS Report* 9 https://ui.adsabs.harvard.edu/abs/1973usgs.rept….9Y (1973) 10.3133/ofr73366.

[75] Schulz, S. S., Burford, R. O. & Nason, R. D. *Catalog of Creepmeter Measurements in Central California from 1973 through 1975*. *Open-File Report*10.3133/ofr7731 (1976).

[76] Tamura, Y., Sato, T., Ooe, M. & Ishiguro, M. A procedure for tidal analysis with a Bayesian information criterion. *Geophys. J. Int.* **104**, 507–516 (1991).

[77] Tamura, Y. & Agnew, D. C. Baytap08 User’s Manual. (2008).

[78] Torrence, C. & Compo, G. P. A practical guide to wavelet analysis. *Bull. Am. Meteorol. Soc.* **79**, 61–78 (1998).

[79] Seydoux, L. et al. Clustering earthquake signals and background noises in continuous seismic data with unsupervised deep learning. *Nat. Commun.* **11**, 3972 (2020). 32769972 10.1038/s41467-020-17841-x PMC7414231

[80] Abed, W. et al. Hidden patterns in volcanic seismicity: deep learning insights from Mt. Etna’s 2020–2021 activity. *Sci. Rep.* (2026). 10.1038/s41598-026-36677-x PMC12905119 41580511

[81] Leonard, M. Earthquake fault scaling: self-consistent relating of rupture length, width, average displacement, and moment release. *Bull. Seismol. Soc. Am.* **100**, 1971–1988 (2010).

[82] Wells, D. L. & Coppersmith, K. J. New empirical relationships among magnitude, rupture length, rupture width, rupture area, and surface displacement. *Bull. Seismol. Soc. Am.* **84**, 974–1002 (1994).

[83] Hanks, T. C. & Kanamori, H. A moment magnitude scale. *J. Geophys. Res. Solid Earth* **84**, 2348–2350 (1979).

[84] Zali, Z. ZahraZali/DeepStrain: Initial release of DeepStrain. *Zenodo*10.5281/zenodo.16911303 (2025).

[85] Zali, Z. AutoencoderZ. Zenodo https://zenodo.org/records/14284460 (2024).

[86] Liu, Y.-K., Ross, Z. E., Cochran, E. S. & Lapusta, N. A unified perspective of seismicity and fault coupling along the San Andreas Fault. *Sci. Adv.* **8**, eabk1167 (2022). 35196076 10.1126/sciadv.abk1167 PMC8865773

