# Supplementary material for: Slow slip modulates low-frequency seismicity on the Parkfield segment of the San Andreas Fault
Source: Nat Commun. 2026 Jun 9;17:5137. doi: 10.1038/s41467-026-74095-9 (PMC13249816; doi:10.1038/s41467-026-74095-9)
Supplement: Supplementary file 1 — Supplementary Information [file 41467_2026_74095_MOESM1_ESM.pdf]

Supplementary Information

Slow slip modulates low-frequency seismicity on the San Andreas Fault

Zahra Zali[1], Patricia Martínez-Garzón[1,2], David Mencin[3], Gregory C. Beroza[4]

[1] GFZ Helmholtz Centre for Geosciences, Potsdam, Germany

[2] RWTH Aachen University, Aachen, Germany

[3] EarthScope Consortium, Washington DC, U.S.A.

[4] Department of Geophysics, Stanford University, Stanford, California, USA

Correspondence to: Zahra Zali (zali@gfz.de)

Description: This Supplementary Information file contains Supplementary Notes 1 and 2, together with the associated figures. It also includes Supplementary Figures S1–S20 and Supplementary Table S1, which provide additional methodological details, analyses, and supporting results referred to in the main text.

Supplementary Note 1: Investigation of potential longer-duration slow slip events in the Parkfield strainmeter data

Although the primary focus of this study is on short-duration slow slip events (SSEs), we also investigated the possible existence of longer-duration SSEs in the Parkfield strainmeter data to assess whether such events were present but not captured by our main detection framework.

To this end, in addition to the main wavelet transform (WT) analysis used in the manuscript (scales 1–2048, corresponding to sensitivity to transients of approximately 1 s to ~150 minutes), we performed additional WT analyses using extended scales (4000–10000), which are sensitive to transients with durations of up to ~15 hours. To obtain a reasonable time–frequency resolution at these longer scales, we applied the WT to weekly time windows rather than daily segments. Using this extended-scale WT analysis, we systematically examined the strainmeter data for evidence of longer-duration tectonic transients. We did not identify any SSEs beyond the short-duration events already reported in this study.

Supplementary Note Fig. 1a shows an example of a 7-day WT computed using the extended scales (4000–10000), illustrating the absence of any localized transient energy that would indicate a longer-duration SSE. Supplementary Note Fig. 1b presents a known short-duration SSE, previously identified by our main analysis, displayed within a 7-day WT window using the extended-scale settings. While the short-duration SSE remains visible, its temporal localization is degraded at this scale. In Supplementary Note Fig. 1c, we show an example of a longer-duration transient identified during this analysis. A detailed inspection of the corresponding strain time series reveals that this signal is unlikely to be tectonic in origin, as the amplitude of the areal strain component is significantly larger than that of the differential and engineering components. This behavior is inconsistent with the expected strain signature of aseismic fault slip and instead suggests a non-tectonic source. These analyses indicate that no longer-duration SSEs (up to ~15 hours) are present in the Parkfield strainmeter data over the study period at station B073, beyond the short-duration events reported in this work.

To investigate the possible presence of even longer-duration slow slip events (lasting days to weeks), we did not apply automated detection methods, as such events can be more readily identified through visual inspection, although they are typically better resolved by GPS observations. Instead, we visually inspected continuous monthly and yearly strain time series for the entire 8-year study period. These plots, shown in Supplementary Note Fig. 2, do not exhibit abrupt or systematic strain changes indicative of day-long, multi-day, or multi-week tectonic transients.

Together, these analyses show that, using borehole strainmeter data, we detect short-duration SSEs at Parkfield but do not identify evidence for longer-duration SSEs during the study period. This

result highlights the particular advantage of strainmeters for resolving short-lived, small-amplitude aseismic slip transients that are impossible to detect with GPS.

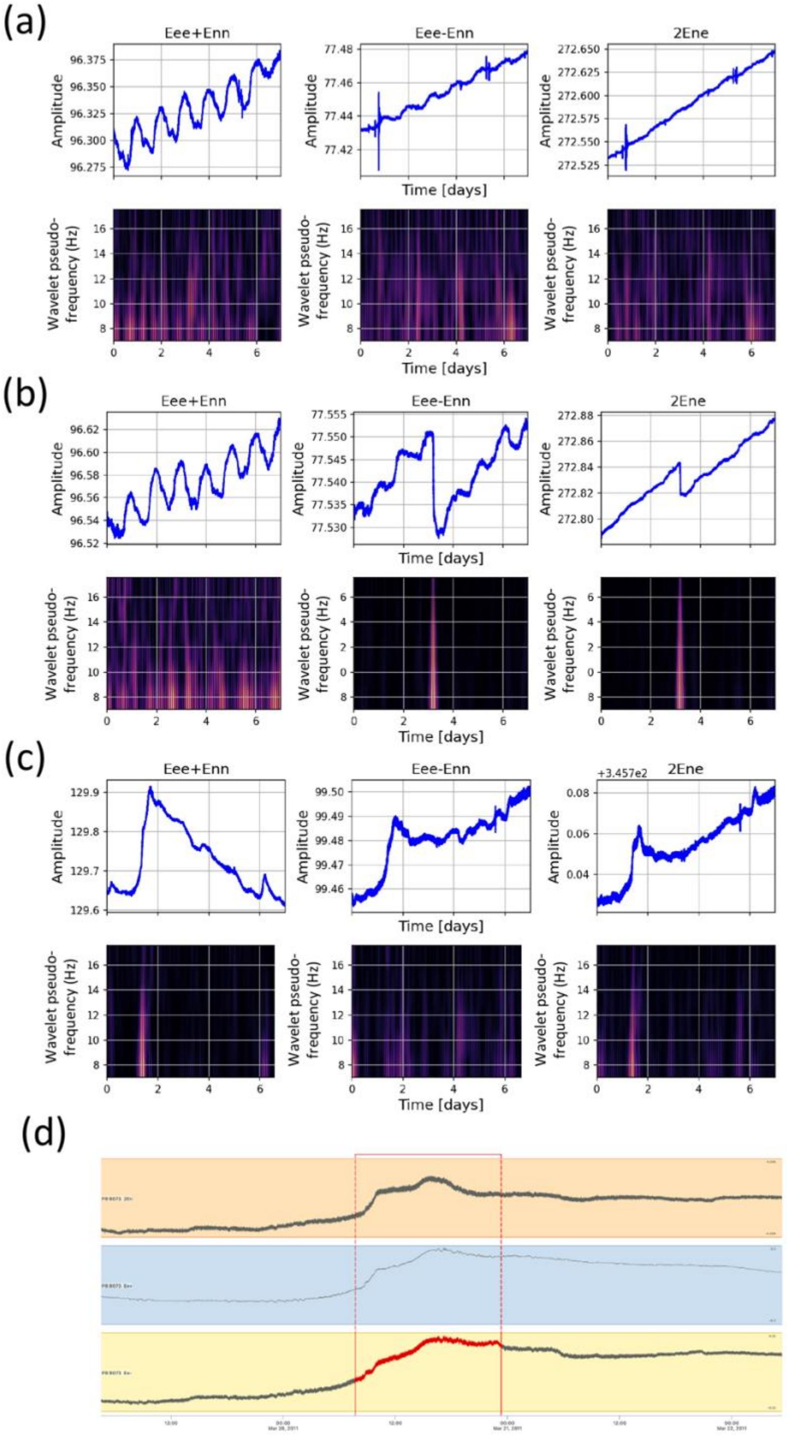

Supplementary Note Fig. 1: Examples illustrating the behavior of strainmeter signals and their wavelet transforms (WT) with scale (4000, 1000), over 7-day windows at station B073. In each

row, the top panels show the time series of the areal (Eee+Enn), differential (Eee–Enn), and engineering (2Ene) strain components, and the bottom panels show the corresponding WT. **a**, A 7-day window without any transient deformation, showing background variability. The plot is related to the week starting from January 15, 2009. The y-axis shows wavelet pseudo-frequencies obtained from the scale–frequency mapping of the continuous wavelet transform. **b**, A 7-day window containing a short-duration SSE, which appears as a localized transient in the strain time series and as a concentrated burst of energy in the WT. The plot is related to the week starting from January 29, 2009, and the SSE is on February 1, 2009. **c**, and **d**, An example of a longer-duration transient (~16 hours) identified in the WT, which, upon inspection of the strain components, is interpreted as non-tectonic due to larger amplitudes on the areal component compared to the engineering and differential components.

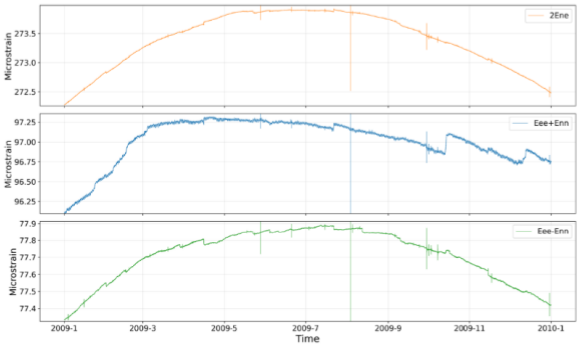
2Ene
Eee+Enn
Eee-Enn
Microstrain
2009-1
2009-3
2009-5
2009-7
2009-9
2009-11
2010-1
Time

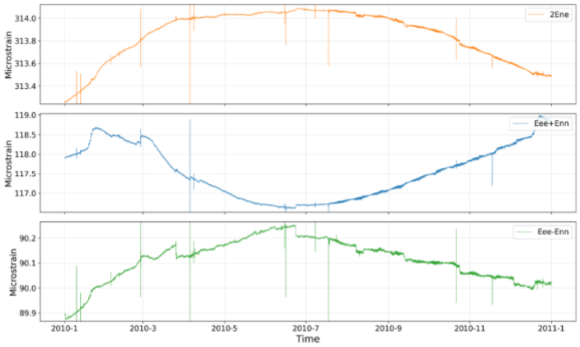
2Ene
Eee+Enn
Eee-Enn
Microstrain
2010-1
2010-3
2010-5
2010-7
2010-9
2010-11
2011-1
Time

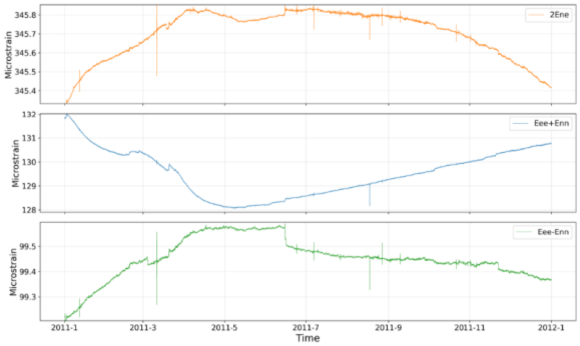
2Ene
Eee+Enn
Eee-Enn
Microstrain
2011-1
2011-3
2011-5
2011-7
2011-9
2011-11
2012-1
Time

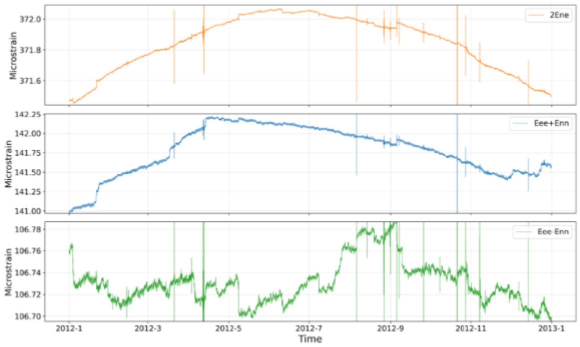
2Ene
Eee+Enn
Eee-Enn
Microstrain
2012-1
2012-3
2012-5
2012-7
2012-9
2012-11
2013-1
Time

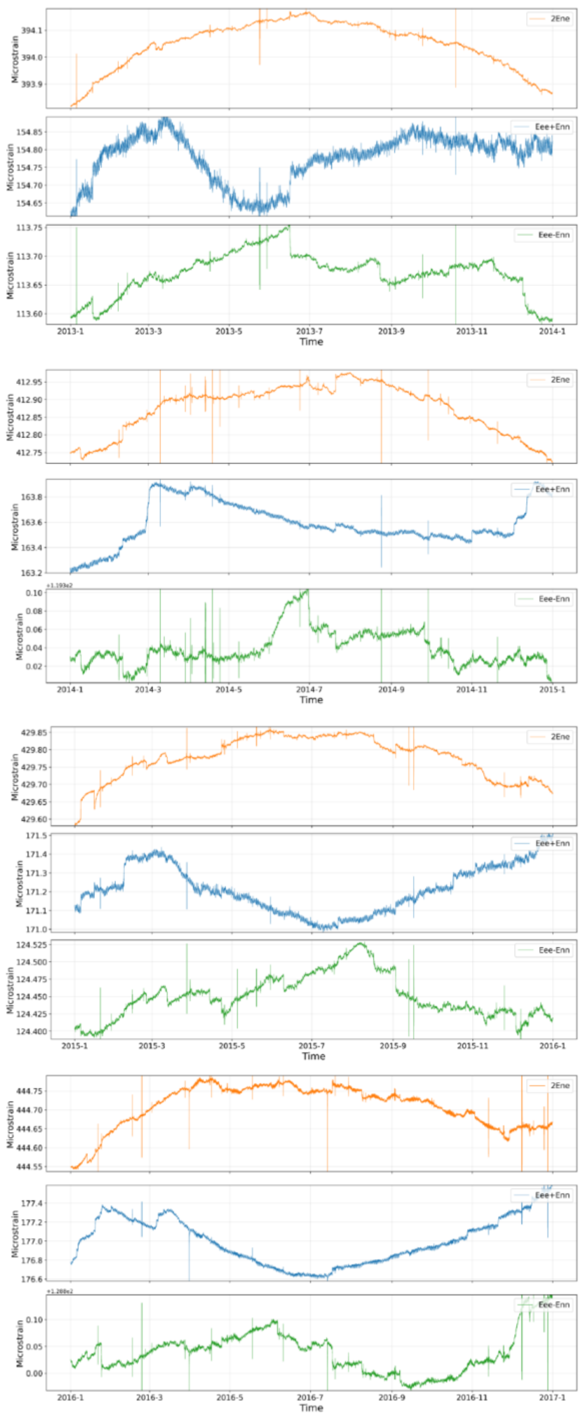

Supplementary Note Fig. 2: Corrected strainmeter time series at station B073 shown as yearly records for the study period (2009–2016). For each year, the three panels display the engineering (2Ene), areal (Eee+Enn), and differential (Eee–Enn) strain components. No clear day-long or multi-day step-like strain changes indicative of longer-duration SSEs are observed in any of the years. The observable step-like changes (e.g., in June 2011) correspond to large-amplitude short-duration SSEs already reported in this study.

Supplementary Note 2. Duration sensitivity of the wavelet transform for detecting transient strain signals

This note clarifies the range of transient durations that can be detected using the wavelet transform (WT) configuration applied in this study. With the Morlet wavelet and scales from 1 to 2048, the WT is sensitive to signal variations with characteristic periods from approximately 1 second up to ~40–45 minutes. Transient strain changes whose main deformation occurs within this time window produce concentrated energy at the largest scales and are therefore well resolved.

Although this scale range corresponds to durations up to ~40–45 minutes, step-like strain transients with longer rise times can still produce detectable WT energy. This is because the onset of a step-like increase, even when spread across several hours, contains a finite slope and curvature. These local gradients introduce higher-frequency components with characteristic periods that fall within the 10–40 minute band, where the WT is most responsive. As a result, events with rise times up to roughly 120–150 minutes can still generate visible WT amplitudes, because their short-period curvature projects energy into the resolved range of scales.

As the rise time increases further, the transient becomes increasingly smooth, and the curvature at its onset weakens. The higher-frequency content, therefore, diminishes, and the WT amplitude decreases correspondingly. For rise times of approximately 180 minutes and longer, most of the transient's spectral energy lies at periods beyond the largest resolved scale, making such gradual signals indistinguishable from background variability under this WT configuration.

To examine the duration sensitivity of our WT configuration, we generated synthetic 1-Hz signals that contain step-like transients of controlled duration. Each synthetic signal was constructed as a single continuous 24-hour time series composed of three additive components: a small linear baseline trend to mimic slow background variability, low-amplitude Gaussian noise, and (when included) a ramp-type transient. The transient was generated by placing an ideal step at the midpoint of the day and smoothing its onset through convolution with a Gaussian kernel, producing a gradual increase whose rise time we could prescribe. By varying this rise duration, we created signals with transients of 20, 40, 60, 120, 150, 180, and 240 minutes, as well as a signal with no transient. All synthetic signals were then processed using the same WT settings used in the main analysis (Morlet wavelet, scales 1–2048). Supplementary Note Fig. 3 presents the resulting time series and WT panels. Events lasting 20–60 minutes are clearly identifiable, events of 120–150 minutes remain detectable with reduced amplitude, and events of ≥180 minutes no longer produce a distinguishable WT signature.

These tests demonstrate that our WT configuration is most sensitive to short-duration transients and remains capable of detecting moderately longer events (up to ~150 minutes), whereas very gradual multi-hour deformation falls outside the resolvable period range.

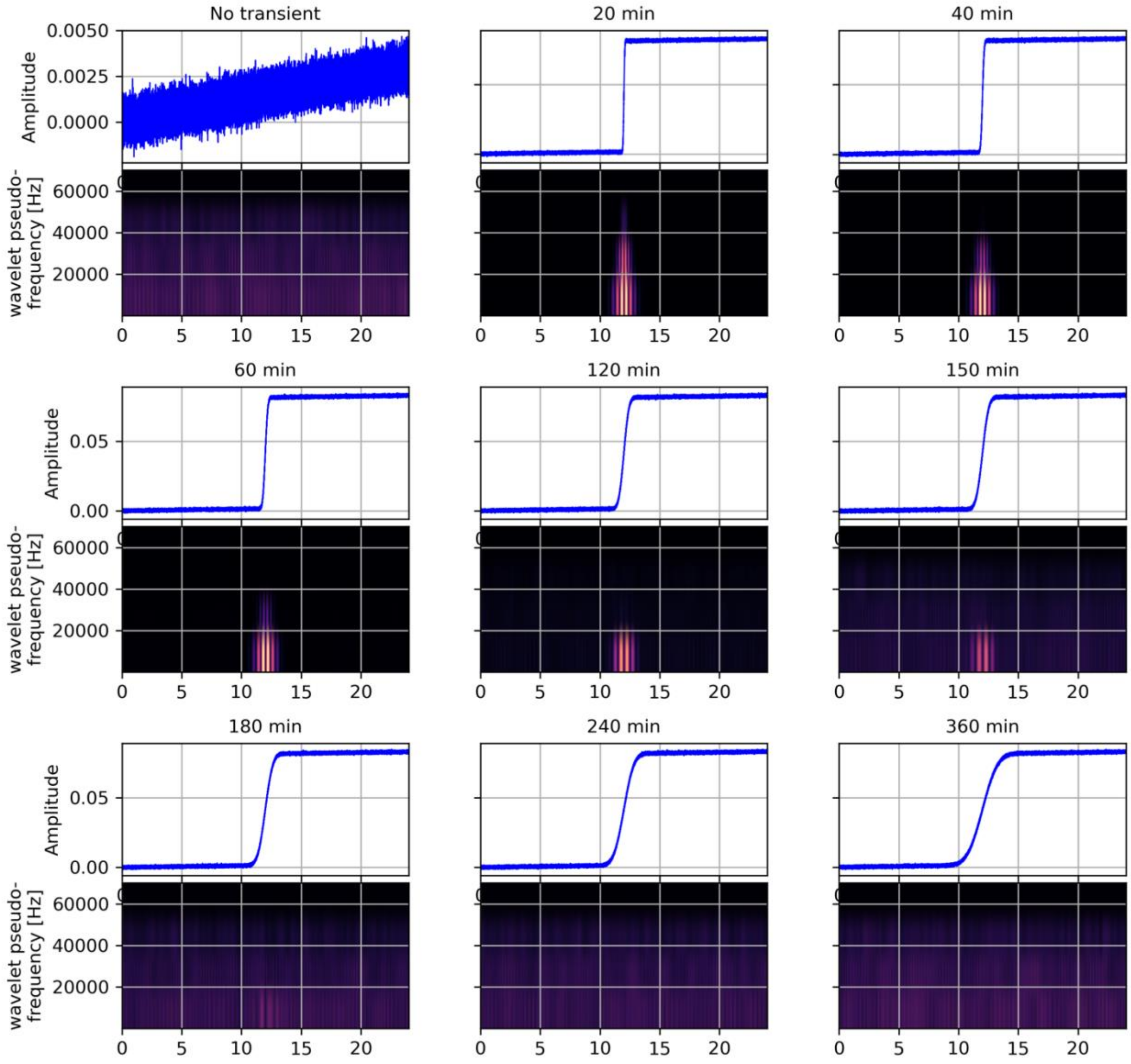

Supplementary Note Fig. 3: Wavelet-transform visibility of step-like transients with different rise times. Synthetic 1-Hz signals containing no transient (top left) and step-like increases with rise times of 20, 40, 60, 120, 150, 180, 240, and 360 minutes. Each panel shows the synthetic time series (top) and its continuous wavelet transform (bottom), computed using the same WT configuration applied in the main analysis (Morlet wavelet, scales 1–2048). Transients with rise times up to approximately 120–150 minutes produce concentrated energy at the largest wavelet scales (toward the bottom of the WT panels), whereas events with rise times ≥ 180 minutes no longer generate a distinct WT signature. These results illustrate the duration range over which the WT can resolve step-like transient deformation under the settings used in this study. The y-axis shows wavelet pseudo-frequencies obtained from the scale–frequency mapping of the continuous wavelet transform.

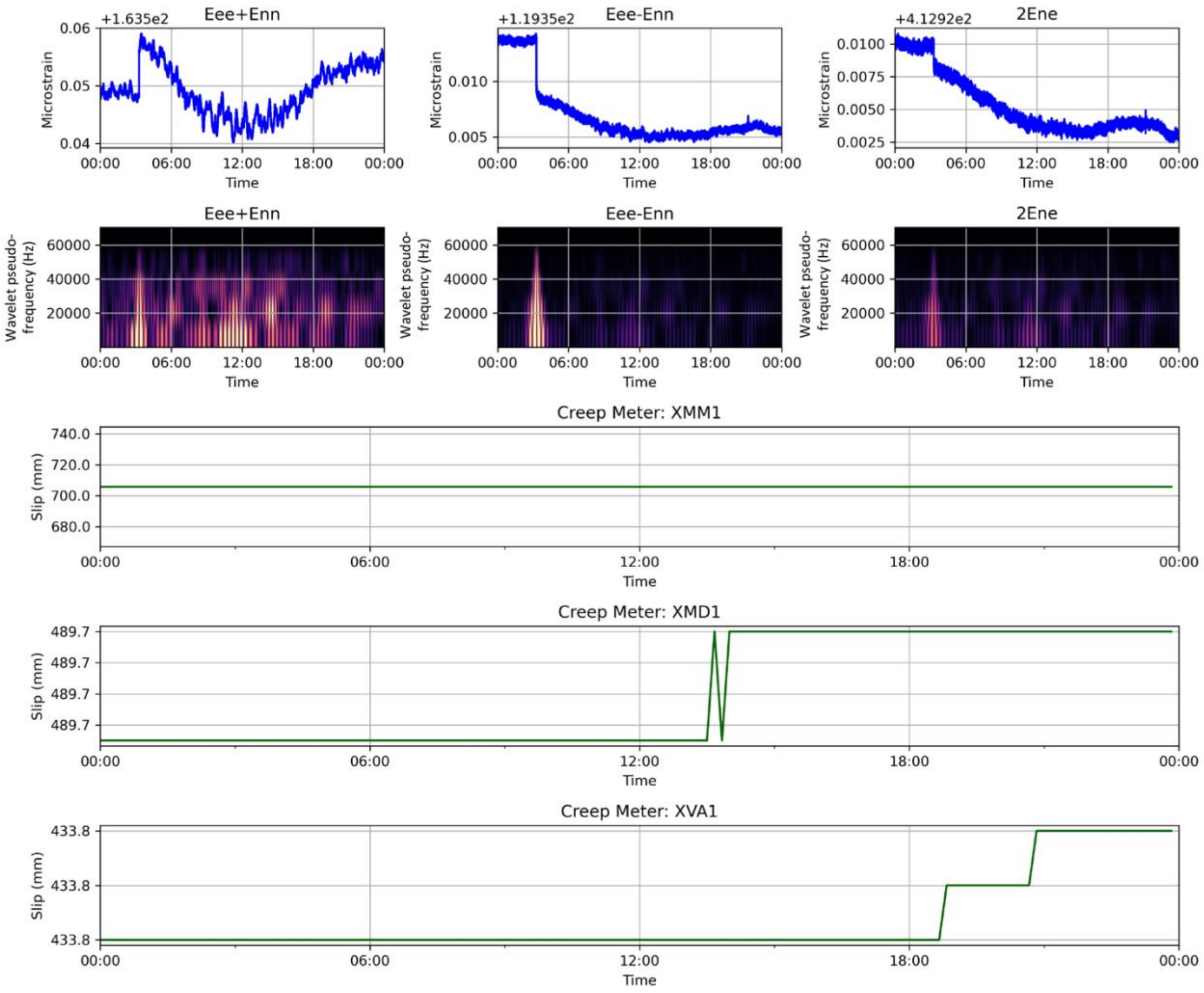

Supplementary Fig. 1: False detection on 2014-07-15 interpreted as non-SSE. Despite a transient in the wavelet transform, the amplitude distribution across components is inconsistent with typical SSE signatures. Specifically, the areal component (Eee+Enn) shows a significantly larger amplitude than the differential and engineering components, suggesting a non-tectonic origin. The y-axis in the wavelet transform plot shows wavelet pseudo-frequencies obtained from the scale–frequency mapping of the continuous wavelet transform.

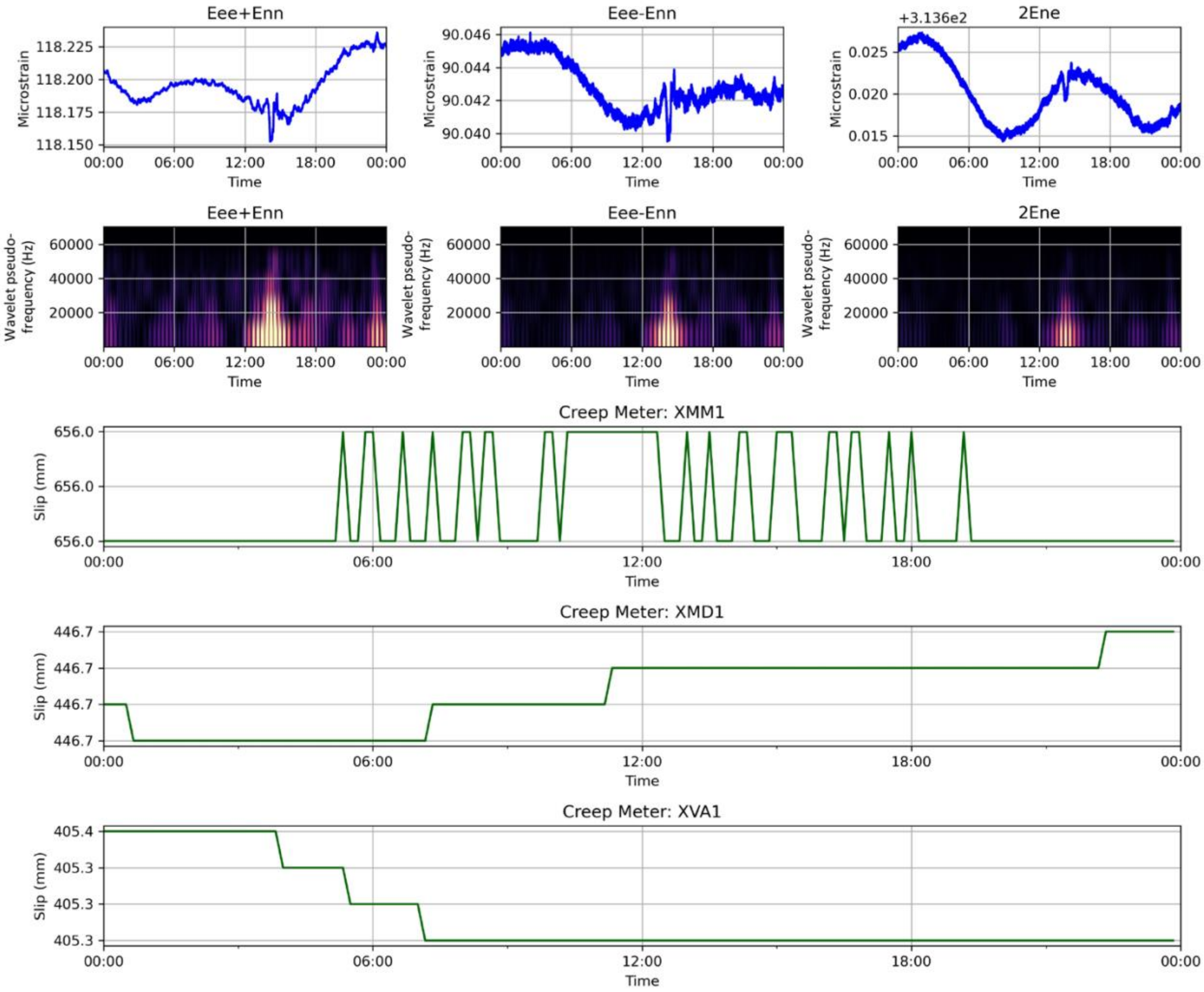

Supplementary Fig. 2: False detection on 2010-11-27 interpreted as non-SSE. The signal exhibits abrupt changes in amplitude but lacks the waveform characteristics of confirmed SSEs. The y-axis in the wavelet transform plot shows wavelet pseudo-frequencies obtained from the scale–frequency mapping of the continuous wavelet transform.

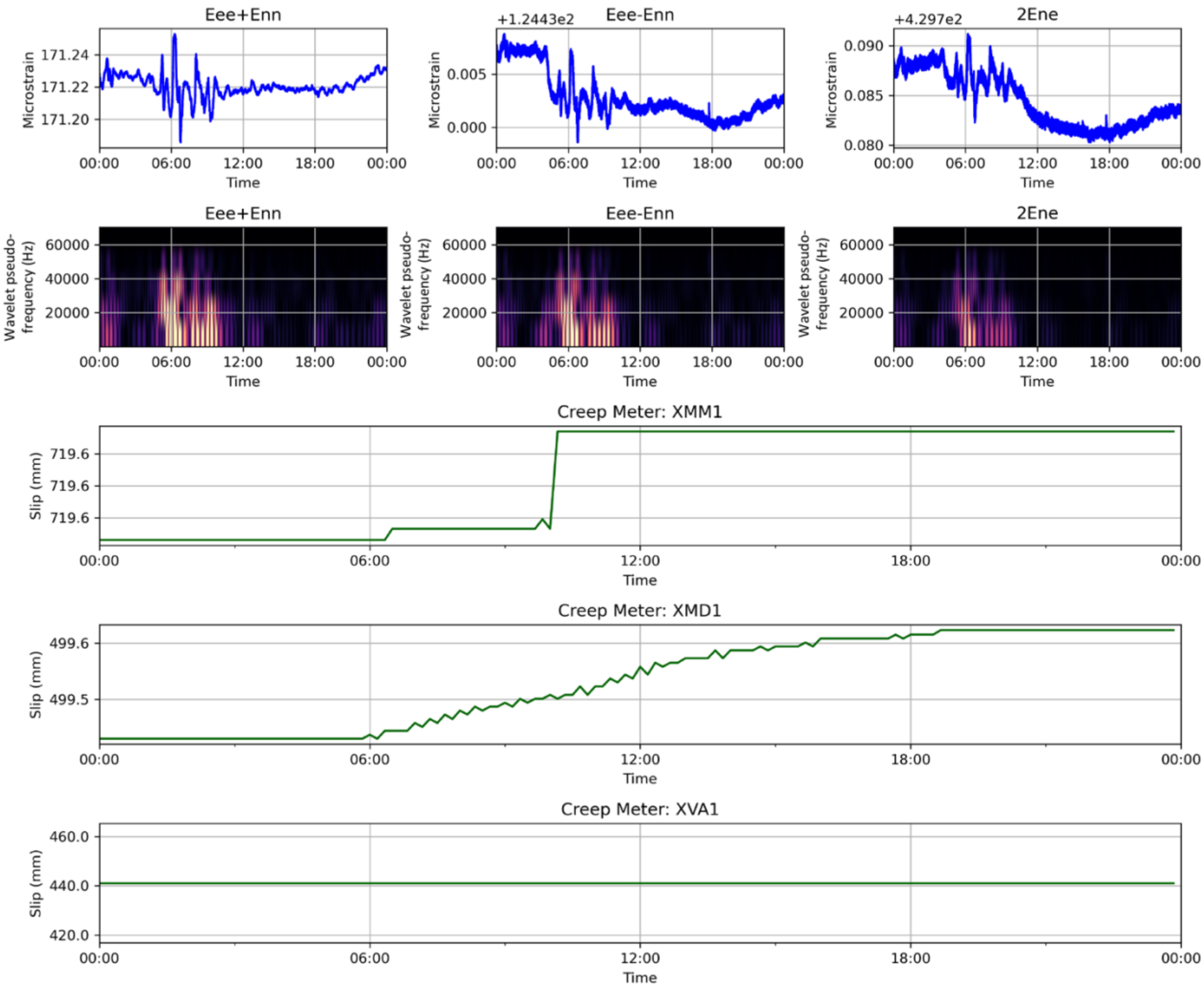

Supplementary Fig. 3: False detection on 2015-10-15 interpreted as non-SSE. Although initially detected by the algorithm, this signal might be attributed to an earthquake. The y-axis in the wavelet transform plot shows wavelet pseudo-frequencies obtained from the scale–frequency mapping of the continuous wavelet transform.

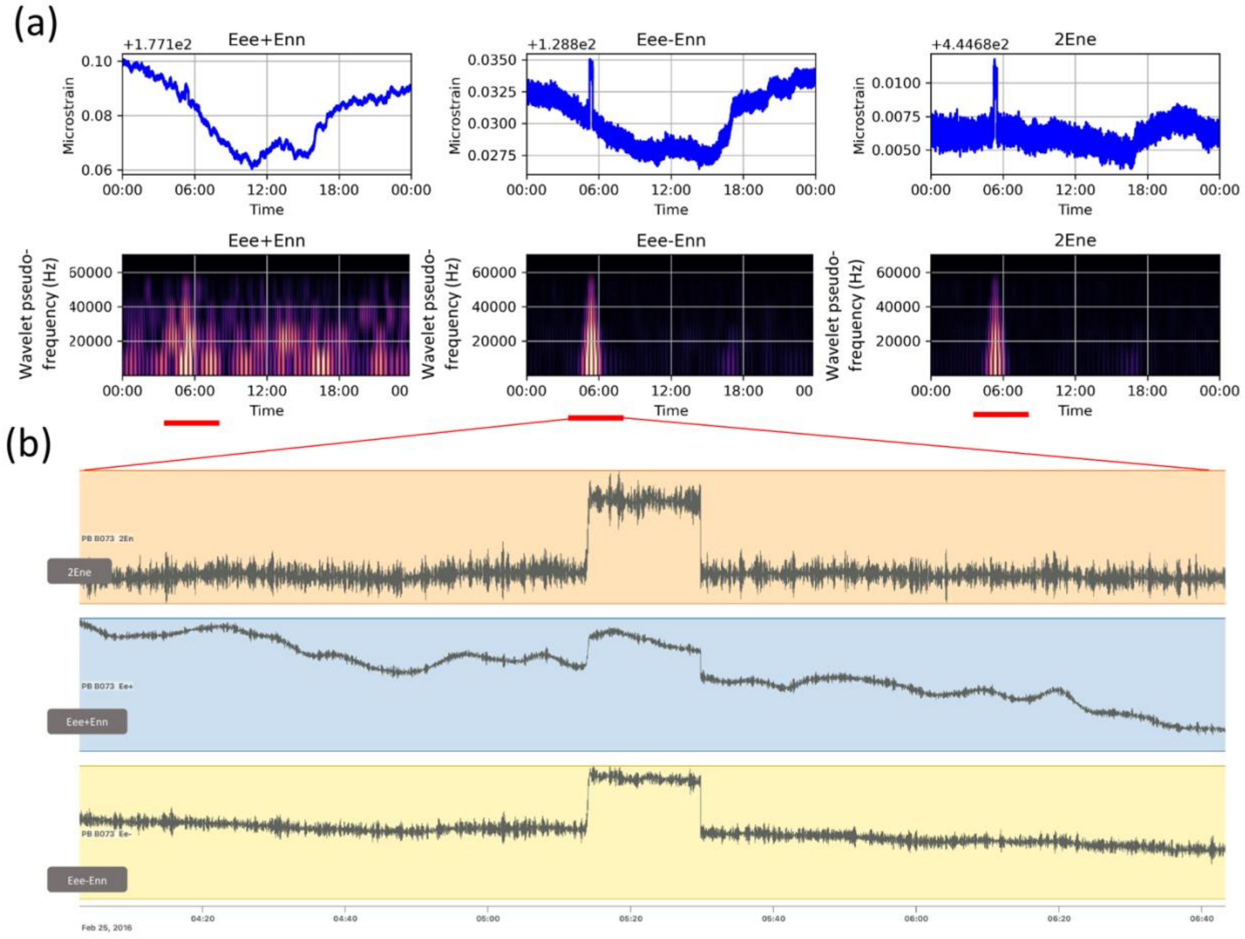

Supplementary Fig. 4: Example of short-duration step-like jumps misidentified as transients by the clustering algorithm. a, Daily strain and corresponding wavelet transform (WT) for the three components (Eee+Enn, Eee–Enn, 2Ene). The WT shows a narrow, high-amplitude feature produced by the abrupt jump near 06:00. **b,** Zoomed view of the three components illustrating the characteristic shape of these artifacts: an instantaneous step-like jump lasting ~16 minutes, followed by a sharp return to the baseline. These features occur only in a limited portion of the 2016 record, and lack the gradual, ramp-like evolution expected for shallow aseismic slip, confirming their non-tectonic origin.

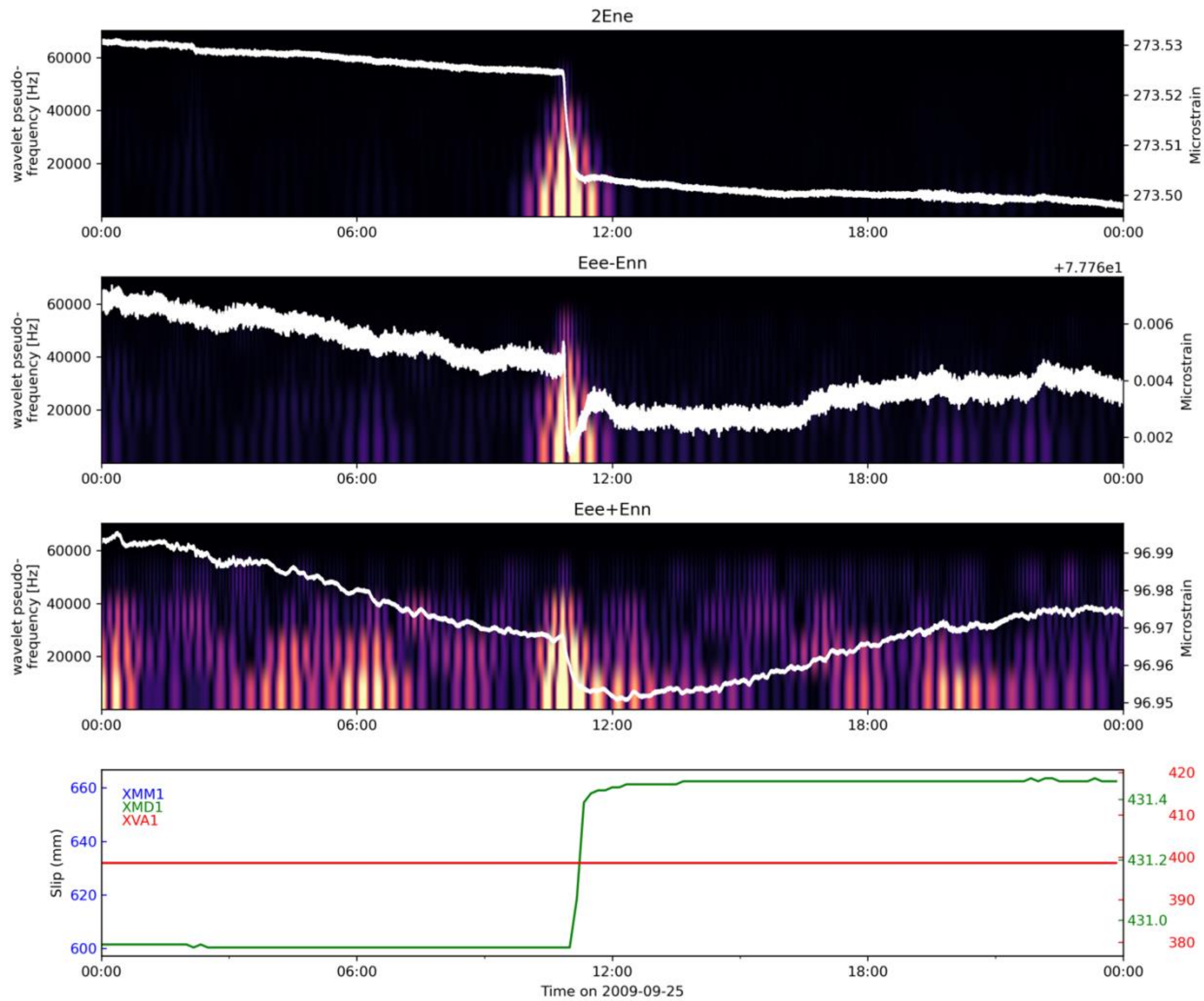

Supplementary Fig. 5: Newly detected SSE not included in the manual catalog. Wavelet transform and strain time series from the three components of strainmeter B073 show a clear transient signal consistent with an SSE, which is also observable on the nearby creepmeter. The y-axis in the wavelet transform plot shows wavelet pseudo-frequencies obtained from the scale–frequency mapping of the continuous wavelet transform.

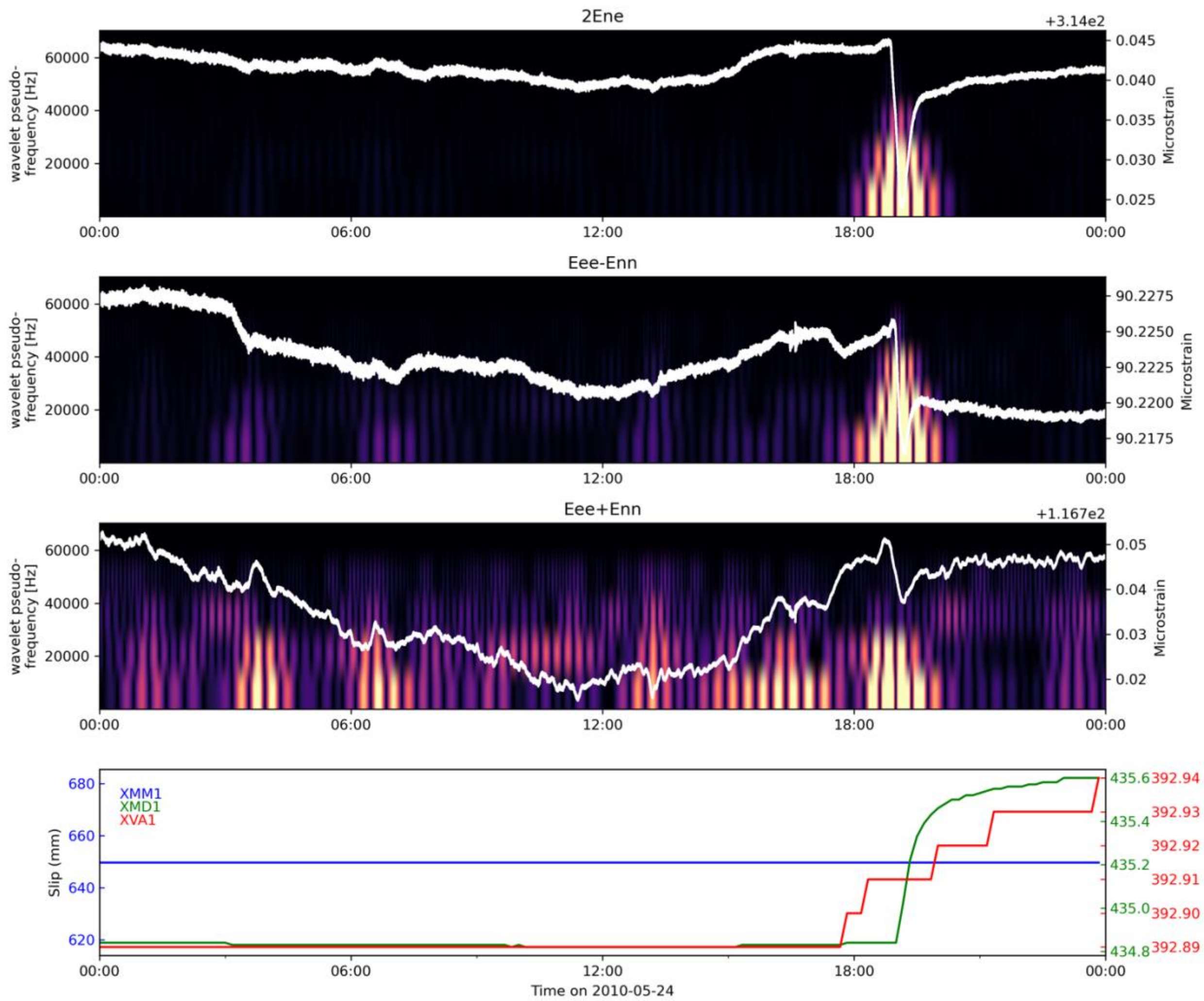

Supplementary Fig. 6: Newly detected SSE confirmed by strain and creepmeter data. Example of an automatically detected SSE supported by coherent signals in the components of B073 and coincident slip on the nearby creepmeter. The y-axis in the wavelet transform plot shows wavelet pseudo-frequencies obtained from the scale–frequency mapping of the continuous wavelet transform.

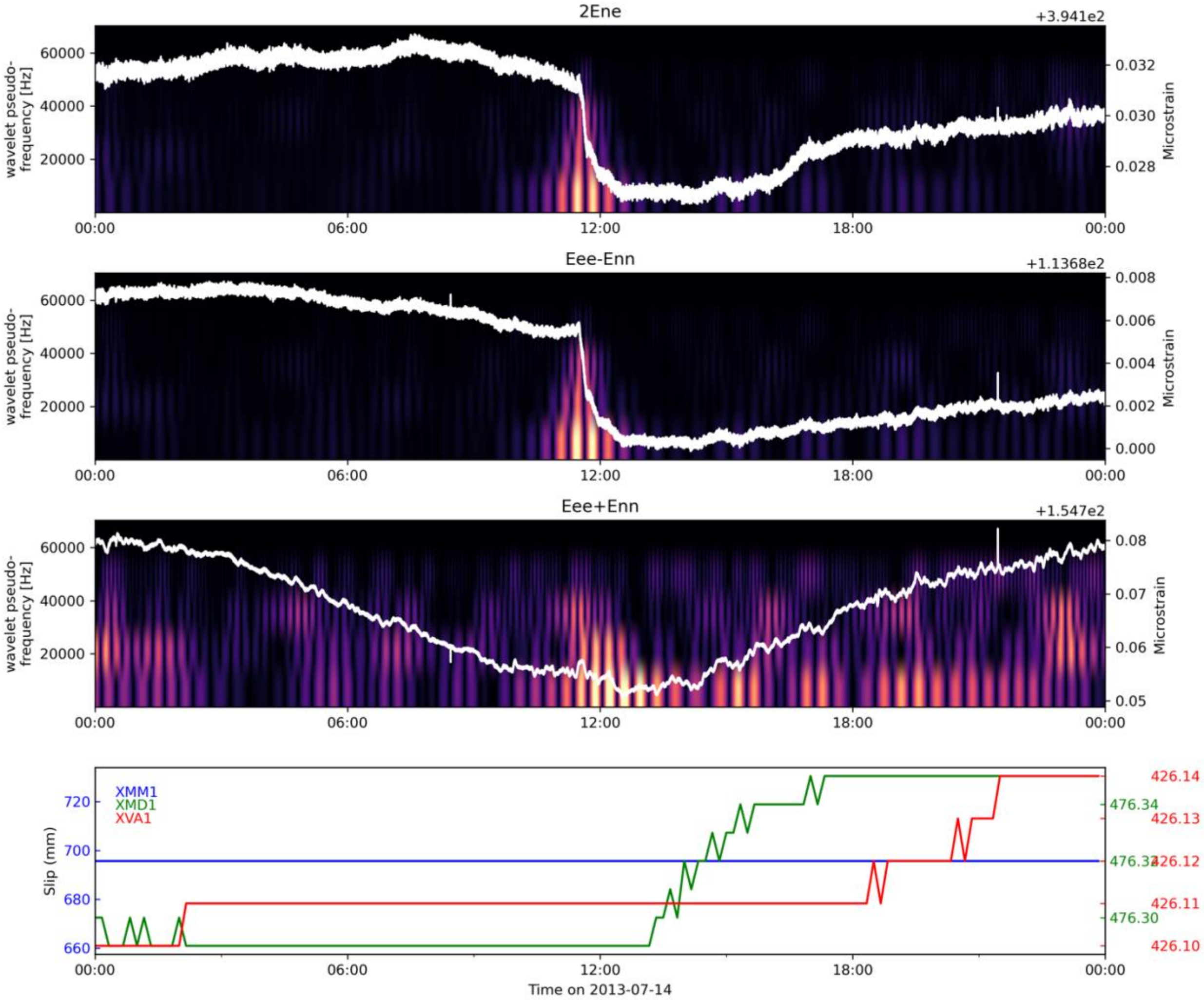

Supplementary Fig. 7: Newly detected SSE with weak expression on creepmeters. Despite the lack of a clear creep signal, the SSE is visible in two components of B073 and confirmed by its waveform and wavelet pattern. The y-axis in the wavelet transform plot shows wavelet pseudo-frequencies obtained from the scale–frequency mapping of the continuous wavelet transform.

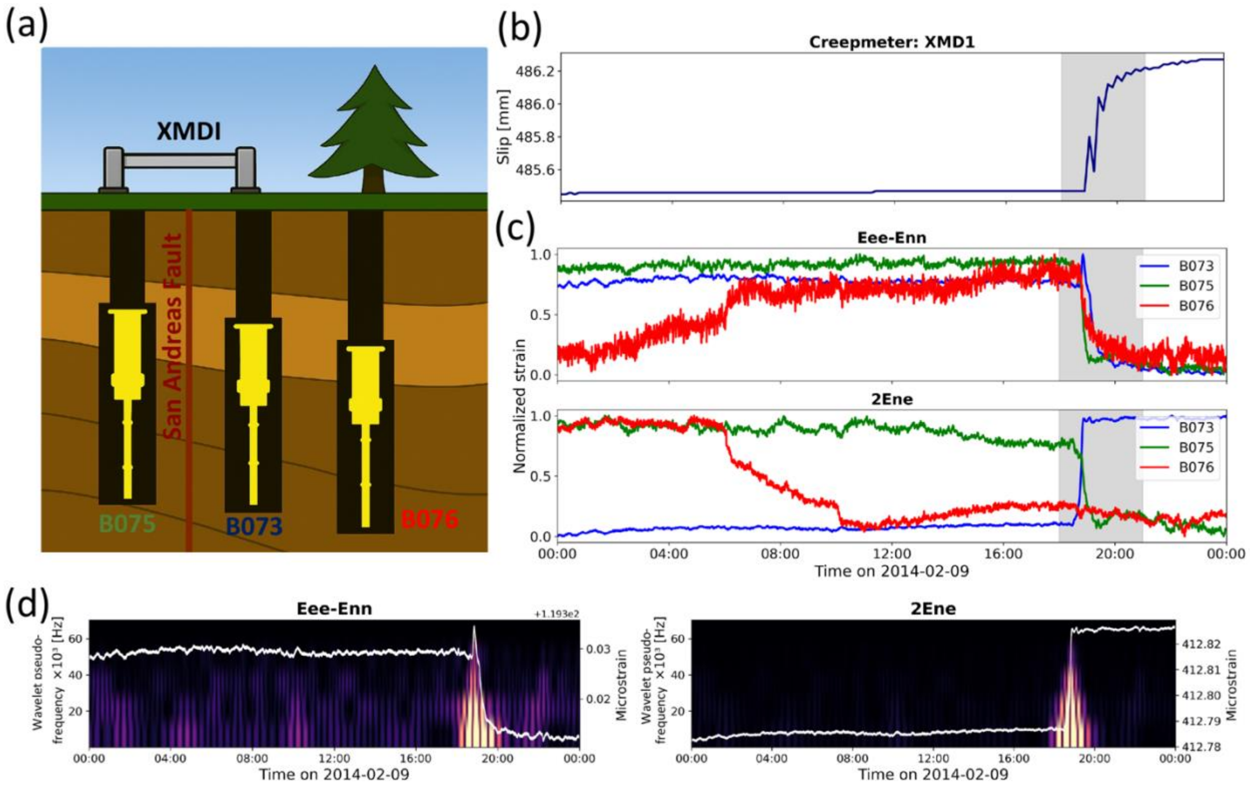

Supplementary Fig. 8: Example of an SSE from the manual catalog also identified by our algorithm. a, Schematic illustration of the instrument configuration, showing the surface creepmeter XMD1 and three borehole strainmeters (B075, B073, B076) installed at ~150 m depth around the San Andreas Fault. **b**, Creepmeter signal indicating surface slip associated with the SSE. **c**, Normalized strainmeter time series from the three strainmeters. The largest strain signal is observed at B073, followed by B075, with the smallest at B076, consistent with their relative positions to the fault. **d,** Daily wavelet transform (WT) of the two strainmeter components. The corresponding strainmeter signals are overlaid in white. The y-axis shows wavelet pseudo-frequencies obtained from the scale–frequency mapping of the continuous wavelet transform.

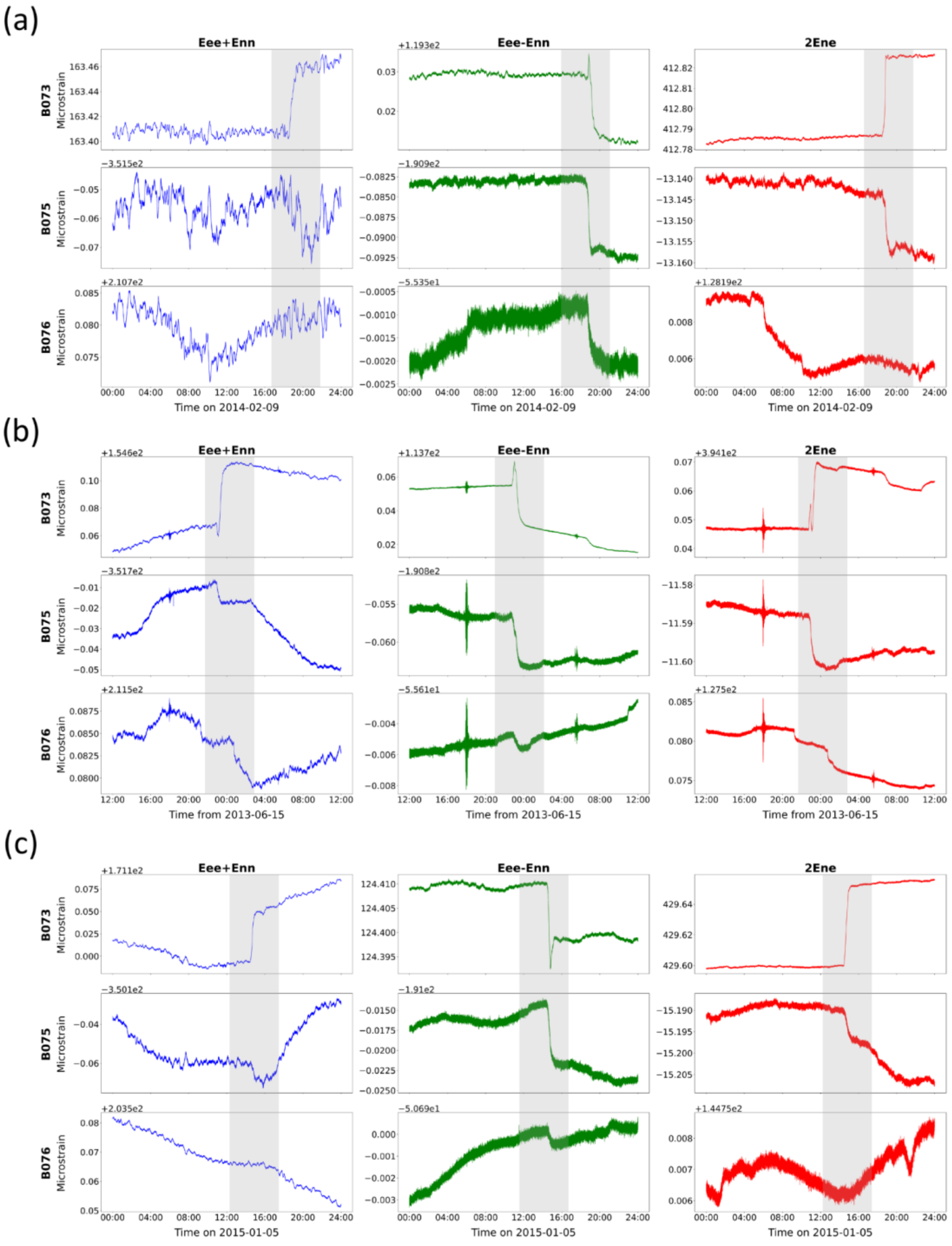

Supplementary Fig. 9: **Examples of SSEs detected on all three strainmeters. a, b, c,** Show strain time series for three different SSEs recorded at stations B073, B075, and B076, respectively. For each station, the areal (Eee+Enn), differential (Eee−Enn), and engineering (2Een) strain components are plotted. The SSEs exhibit consistent transient signals across the stations.

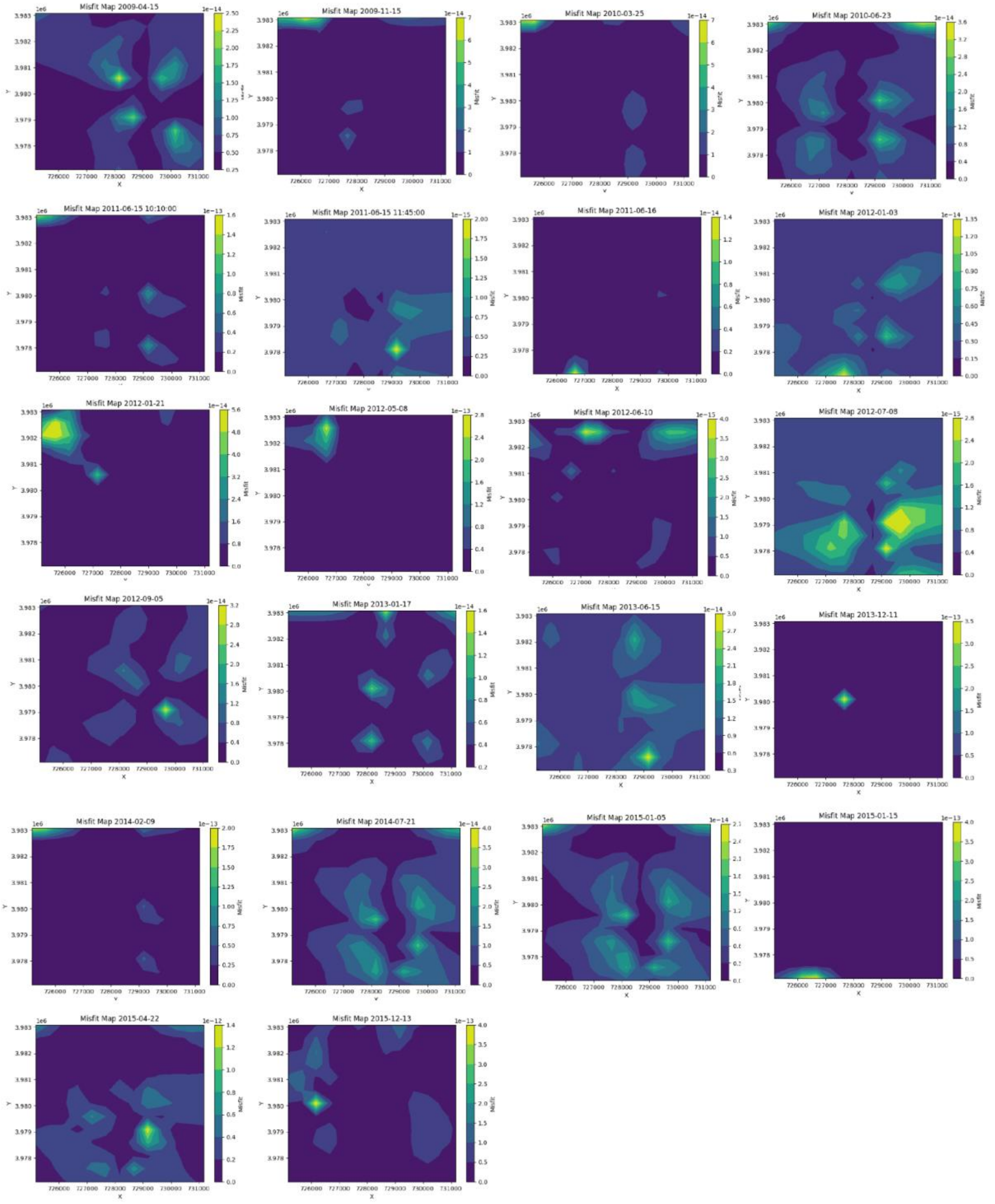

Supplementary Fig. 10: **Misfit maps for horizontal source location (x–y) for all SSEs used in the source analysis.** Colors indicate the misfit between observed and modeled strain for each candidate source location. In some cases, misfit minima form elongated or broad regions rather than sharp point minima, illustrating the limited horizontal resolution imposed by the sparse three-station geometry.

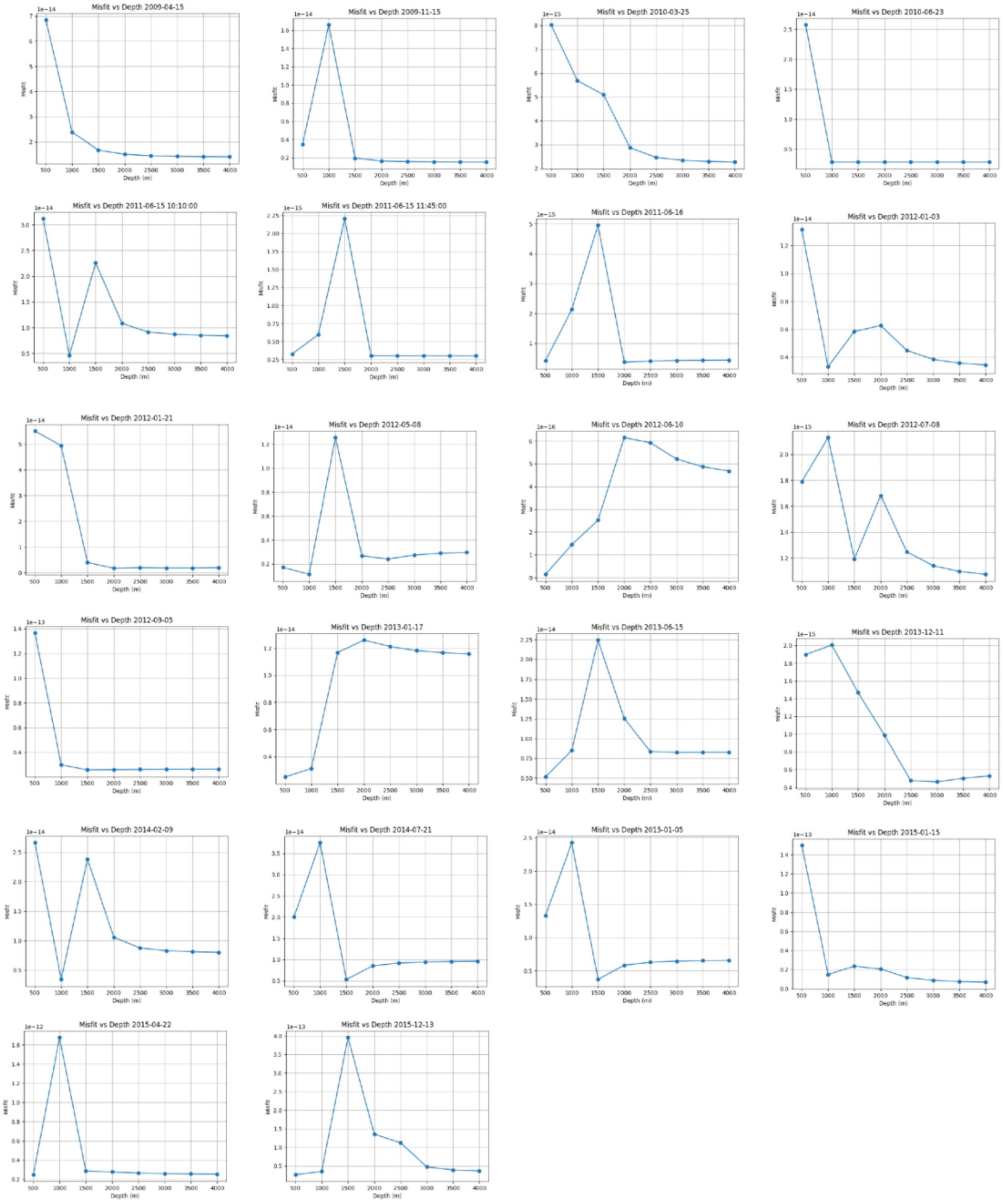

Supplementary Fig. 11: **Misfit as a function of source depth for all SSEs included in the source inversion.** Each panel shows the variation of misfit with depth while other parameters are fixed at their best-fitting values. In most cases, the misfit curves exhibit shallow minima and become relatively flat below ~3–4 km depth, indicating limited depth resolution with the available three-station strainmeter network.

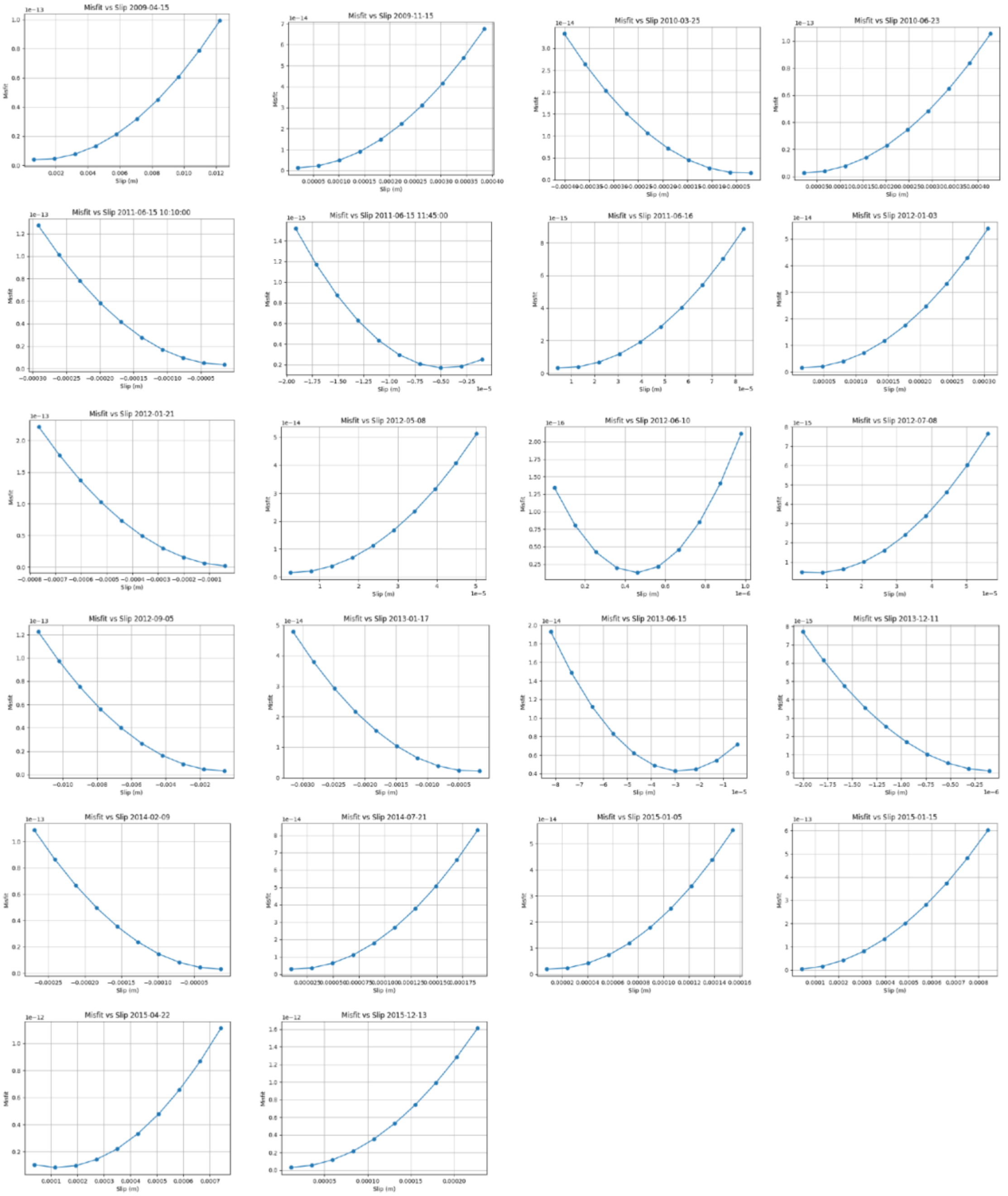

Supplementary Fig. 12: **Misfit as a function of slip amplitude for all SSEs included in the source analysis.** The convex misfit curves indicate that slip magnitude is generally better resolved than source depth or horizontal location, supporting the robustness of moment estimates compared to geometric parameters.

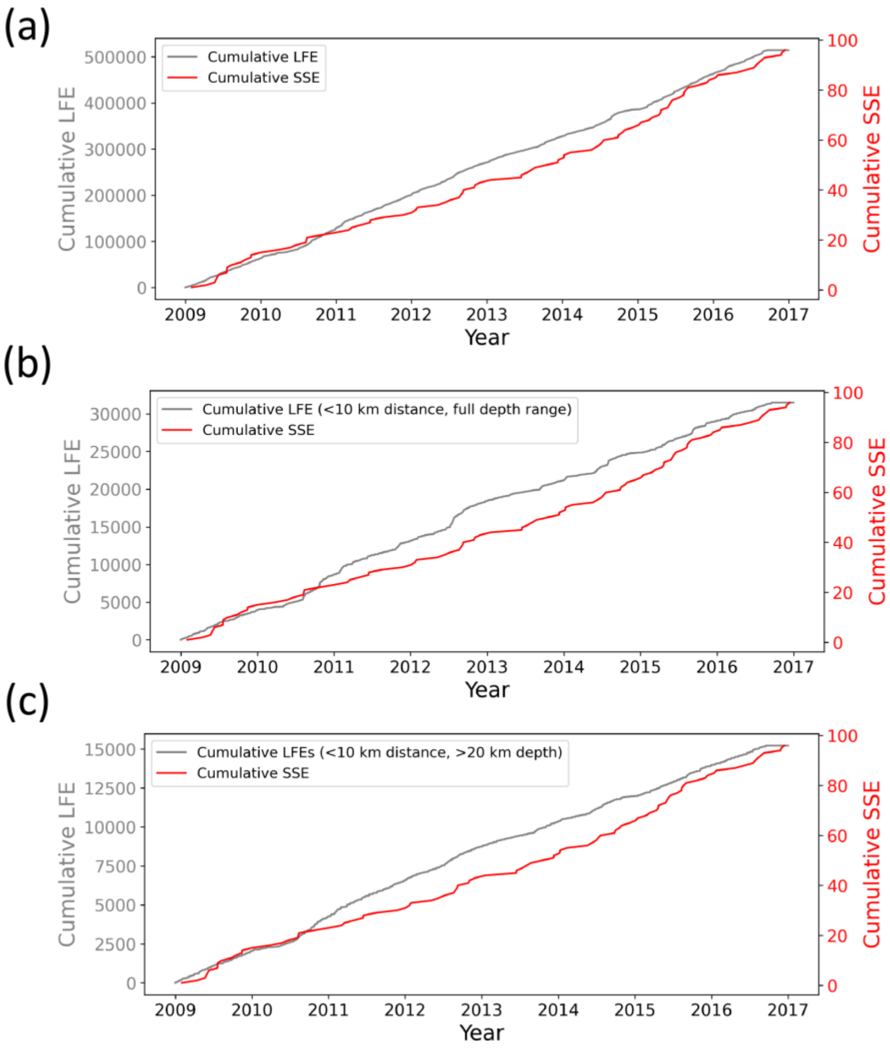

Supplementary Fig. 13: Temporal relationship between SSEs and LFEs. a, Cumulative number of SSEs and LFEs from 2009 to 2016. **b**, Cumulative counts restricted to LFEs within 10 km of station B073. **c**, Cumulative counts restricted to LFEs within 10 km of station B073 and at depths greater than 20 km.

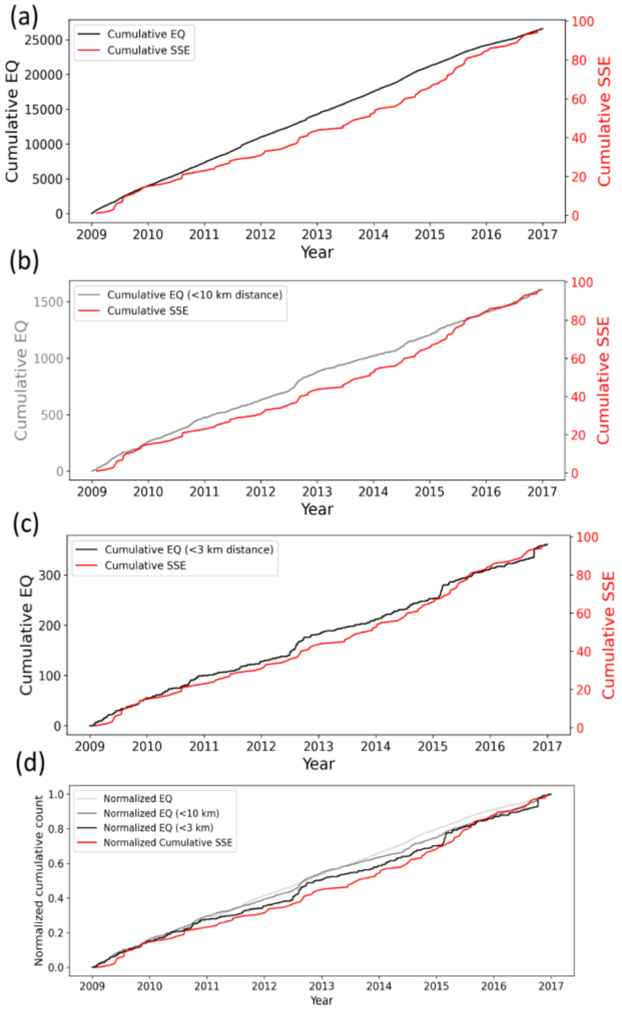

Supplementary Fig. 14: Cumulative number of SSEs with that of earthquakes. a, Cumulative number of SSEs and earthquakes in the central San Andreas Fault region for all events in the catalog (Latitude range: 34.50033; 36.99983, Longitude range: -121.99733; -119.009). No temporal correlation is observed between SSEs and earthquakes. **b,** Cumulative number of SSEs and earthquakes within 10 km of B073. **c,** Cumulative number of SSEs and earthquakes within 3 km of B073. **d**, Normalized cumulative counts of SSEs and earthquakes for all events in the catalog (light gray), events within 10 km (black), and events within 3 km (dark gray) of B073. A weak correlation is observed between SSEs and 3–10 km earthquakes, but the association is notably weaker than that with LFEs (Fig. 5a). The earthquake catalog is from the Northern California Earthquake Data Center.

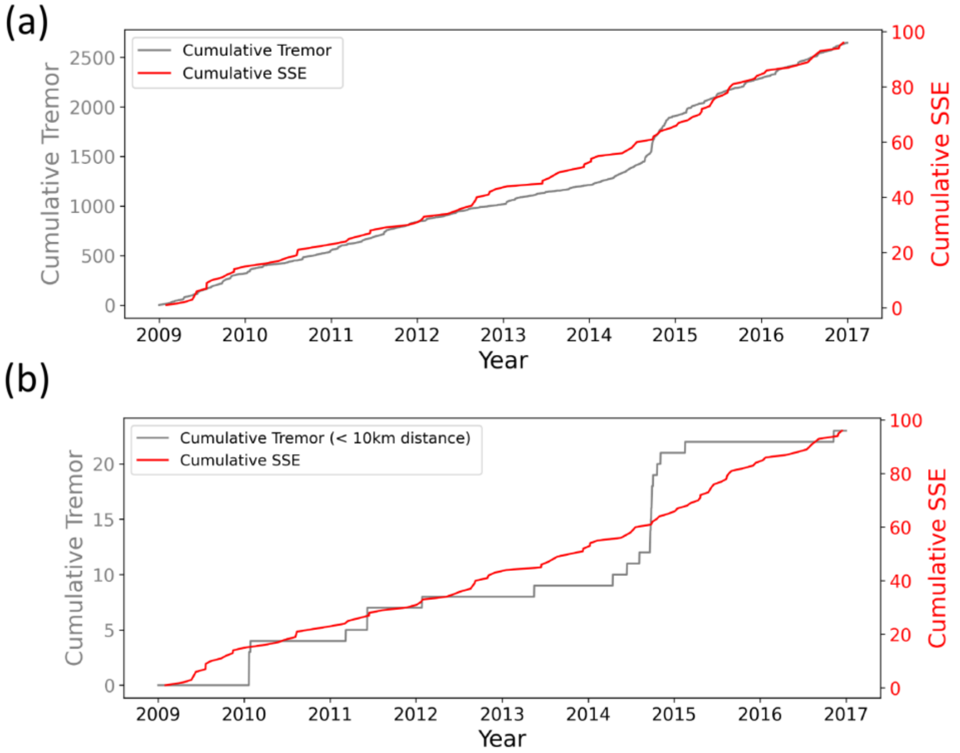

Supplementary Fig. 15: Cumulative number of SSEs with that of tremors. a, Cumulative number of SSEs and tremors in the central San Andreas Fault region (Latitude range: 31.88819; 37.27997, Longitude range: -124.2713; -118.98015). **b**, Cumulative number of SSEs and tremors within 10 km of station B073. No temporal correlation is observed between SSEs and tremor in both plots. The tremor catalog is from the TremorScope catalog (Berkeley Seismology Lab).

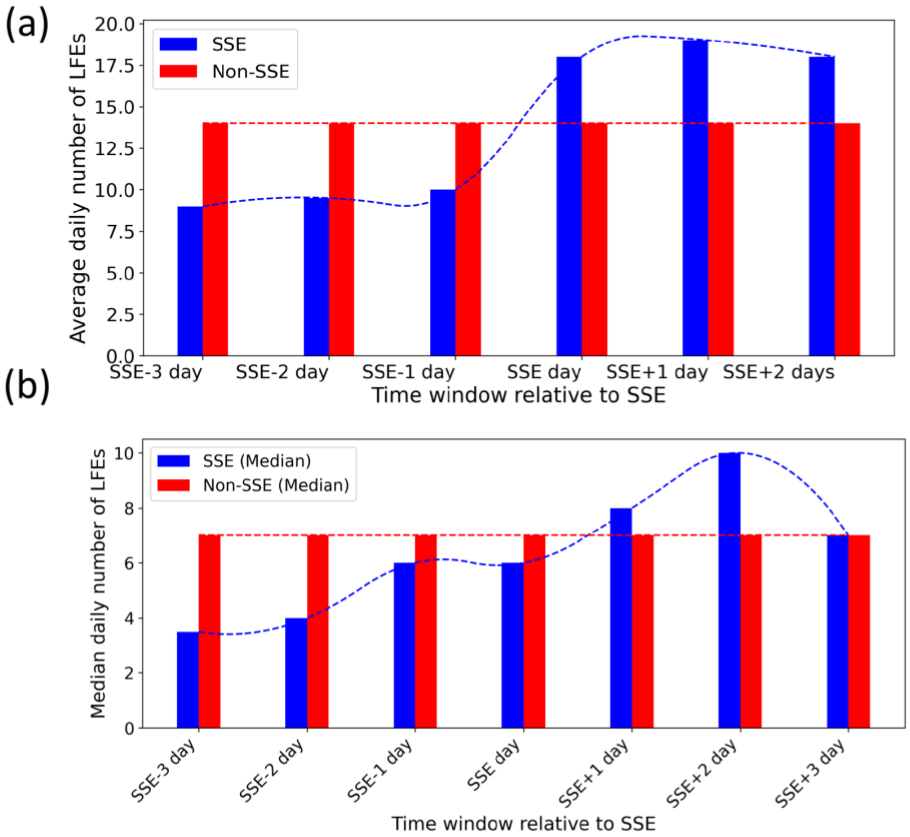

Supplementary Fig. 16: Comparison of low-frequency earthquake (LFE) activity during SSE and non-SSE periods. a, Average daily number of LFEs in time windows from three days before to two days after SSE occurrence (blue) compared with non-SSE reference periods (red). In the main manuscript, the mean LFE rate is computed using all calendar days over the 8-year study period, including days without LFEs, while this figure shows the average daily number of LFEs calculated only from days that contain at least one LFE. The increase in LFE activity during and after SSEs is robust regardless of whether all days or only LFE-active days are considered. **b**, Same as **a**, but showing the median daily number of LFEs, computed using only days with at least one detected LFE. Including all calendar days would yield a median of zero due to the large number of LFE-free days. LFEs show increased activity during and after SSE days compared to non-SSE periods, while reduced activity is observed prior to SSE onset.

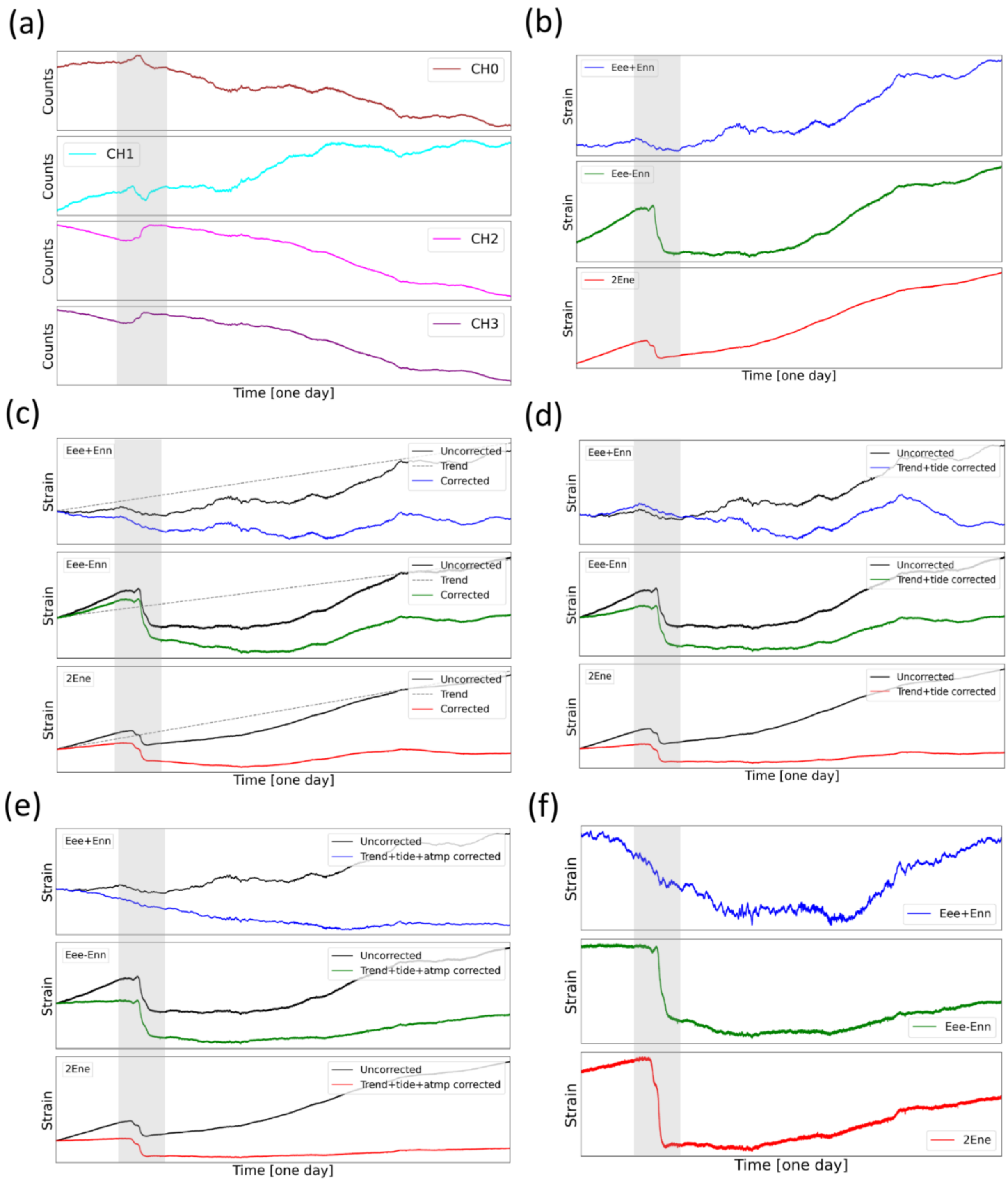

Supplementary Fig. 17: Preprocessing steps for one SSE (on 2009-02-01). a, Raw data from the four gauges of the borehole strainmeter. **b**, Calibration of gauge readings to convert them into strain units. **c-e**, Corrections applied for long-term trend, tidal effects, and atmospheric pressure, respectively. **f**, Final areal, differential, and engineering strain components used for the analysis.

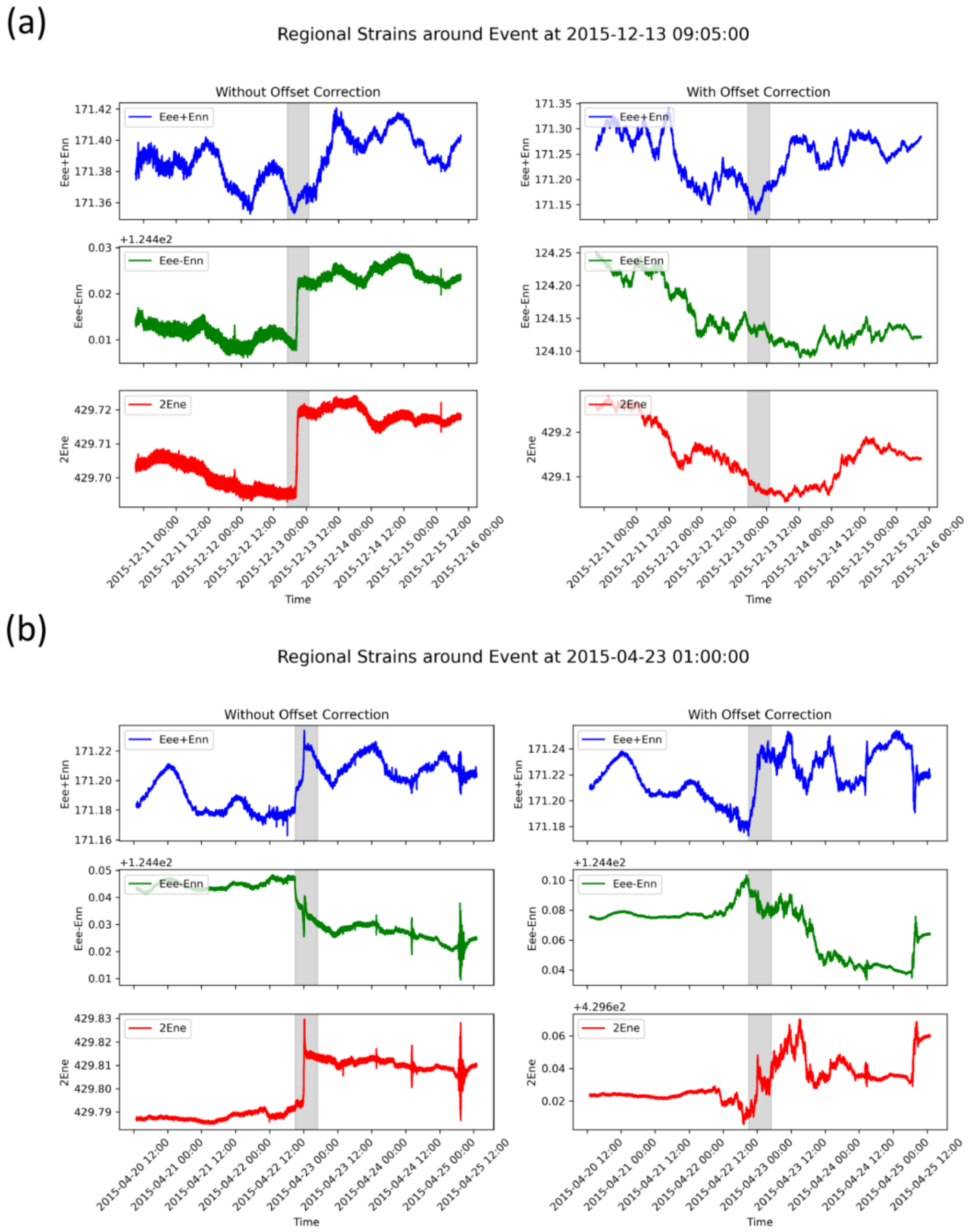

Supplementary Fig. 18: Effect of offset correction on SSE detection. a, and **b,** show two examples of areal (Eee+Enn), differential (Eee−Enn), and engineering (2Een) strain components over a 5-day period, after correction for trend, tidal, and atmospheric pressure effects. The right column shows the original signals, while the left column shows the same data after applying automatic offset correction. In both cases, the SSE signal is distorted or suppressed by the offset correction, potentially leading to failed detection. Therefore, we exclude offset correction from our workflow.

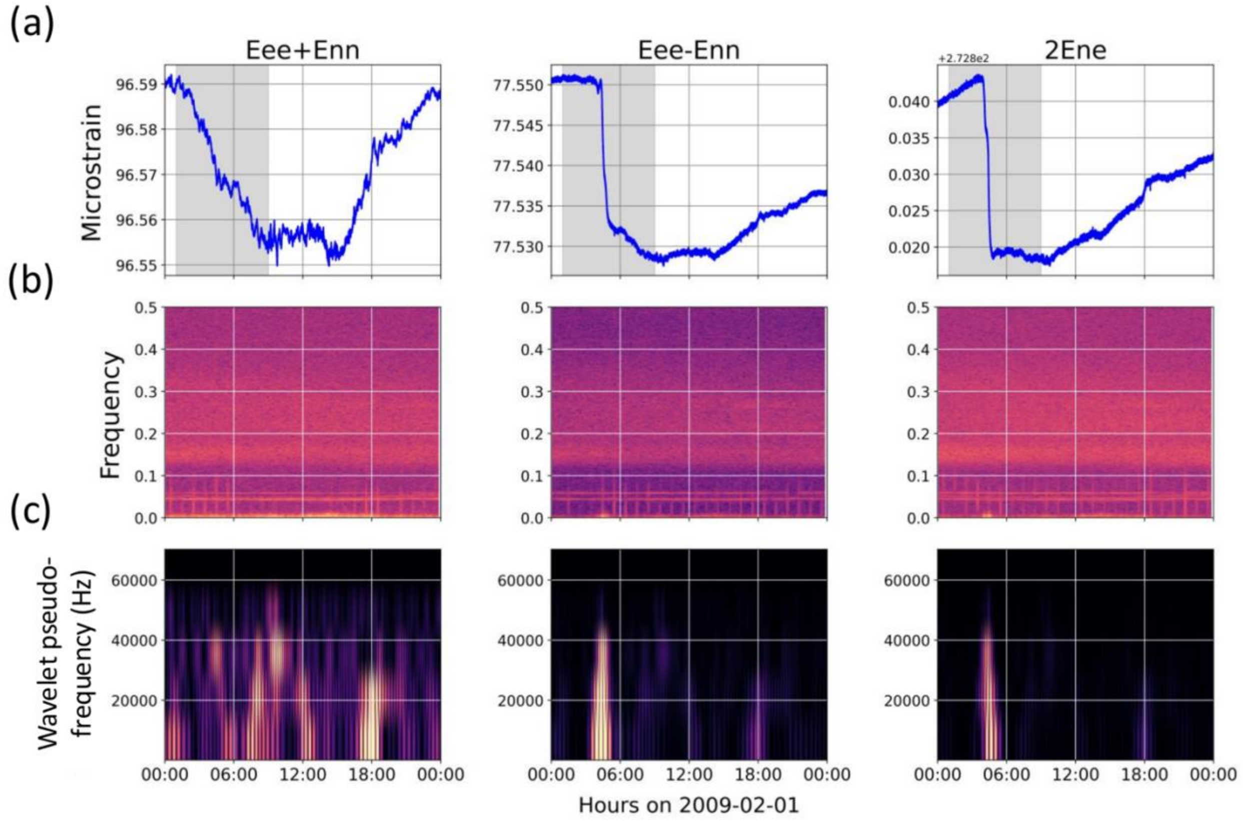

Supplementary Fig. 19: Comparison of STFT and Wavelet Transform for SSE representation. a, shows time series of the areal (Eee+Enn), differential (Eee−Enn), and engineering (2Een) strain components, highlighting a short-term SSE as a transient strain change (gray shaded window). **b,** displays the Short-Time Fourier Transform (STFT) spectrograms of the same components. The SSE does not produce a clear signature in the STFT domain. **c,** shows the corresponding wavelet transform spectrograms, where the SSE appears as a localized burst of energy, particularly in the lower frequency bands. The wavelet transform captures the transient nature of the event more effectively than STFT, making it a better tool for identifying SSEs. The wavelet pseudo-frequencies are converted from wavelet scales and correspond to the same range of periods as the Fourier frequencies shown in the STFT panels. Both STFT and wavelet transforms are computed from the same 1-Hz sampled data and over identical time windows.

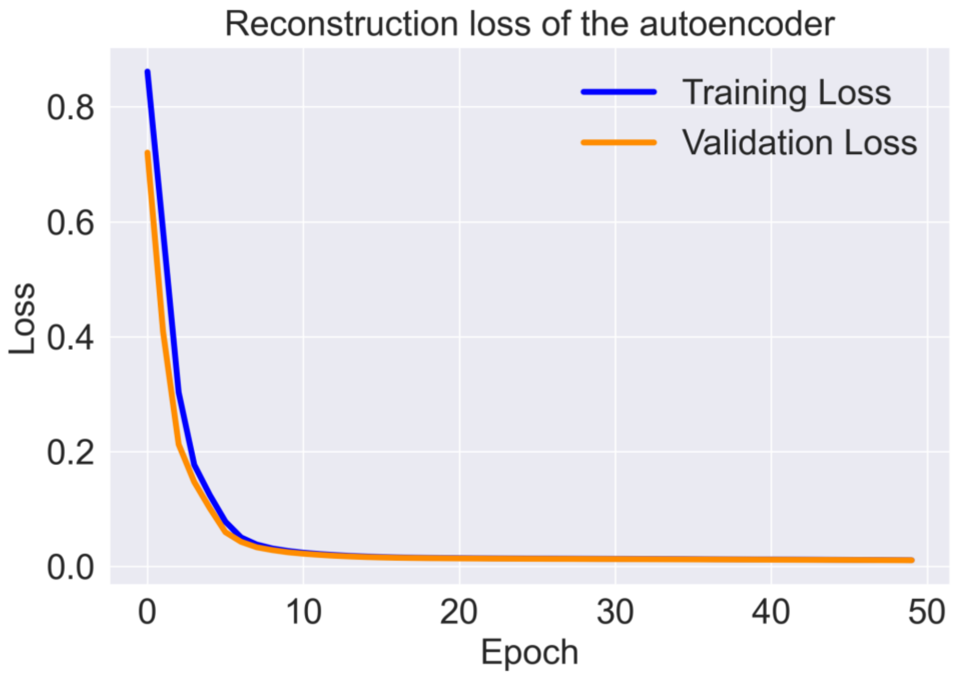

Supplementary Fig. 20: Training performance of AutoencoderZ. Reconstruction loss during training of AutoencoderZ for the B073 differential component, showing good convergence and model performance over epochs.

Supplementary Table 1. Summary of daily LFE rate statistics relative to SSE occurrence.

Category	Mean	Std	IQR
All days (2009–2016)	5.57	13.93	4.0
SSE −3 days	3.91	8.98	3.0
SSE −2 days	4.71	9.58	4.0
SSE −1 days	4.54	8.78	5.0
SSE day	8.09	20.9	4.0
SSE +1 days	8.77	23.14	6.0
SSE +2 days	7.15	15.35	5.0
Mean before SSE	4.39	7.17	nan
Mean after SSE	7.86	16.87	nan
Mean increase (after − before)	3.47	16.57	nan

This table summarizes the statistics of daily low-frequency earthquake (LFE) counts within 10 km of station B073 (depth < 20 km) during 2009–2016. The first row ("All days") reports variability across the entire 8-year daily time series, including days with zero LFEs. The next rows list the mean, standard deviation (Std), and interquartile range (IQR) of LFE counts for each day at fixed offsets from all short-duration SSEs, from 3 days before the onset (SSE − 3 days) to 2 days after (SSE + 2 days). These values represent the distribution of daily LFE counts across all SSEs for a given offset. The lower portion of the table reports averages computed on a per-SSE basis: Mean before SSE is the average of LFE counts over the three days preceding each SSE (-3, -2, -1), then averaged across all SSEs. Mean after SSE is the corresponding average over the first three days following each SSE (0, +1, +2). Mean increase (after − before) quantifies the systematic change in LFE rate associated with SSEs. The IQR represents the central 50% of the distribution and is less sensitive to outliers than the standard deviation. Together, these statistics show that while LFE activity exhibits substantial day-to-day variability, the mean LFE rate consistently increases after SSEs relative to the days preceding them.
