# Supplementary material for: Slow slip modulates low-frequency seismicity on the Parkfield segment of the San Andreas Fault
Source: Nat Commun. 2026 Jun 9;17:5137. doi: 10.1038/s41467-026-74095-9 (PMC13249816; doi:10.1038/s41467-026-74095-9)
Supplement: Supplementary file 2 — Description of Additional Supplementary File [file 41467_2026_74095_MOESM2_ESM.pdf]

**Description of Additional Supplementary Files**

**Supplementary Data 1:** Excel file containing the complete catalog of 92 slow slip events (SSEs) identified in this study, with newly detected SSEs highlighted relative to the previously reported manual catalog. The file also includes the subset of 22 SSEs observed across all three strainmeter stations and used for Okada dislocation modeling, together with strain amplitude changes at each station, associated creepmeter observations, and modeled source parameters including location, depth, moment magnitude, and duration.

**Supplementary Data 2**: This document contains daily plots of the corrected strainmeter signals, wavelet transform results, and traces from three nearby creepmeters for all SSEs identified in this study (2009–2016). The figures provide visual support for the complete SSE catalog presented in Supplementary Data 1.
