# Supplementary material for: Slow slip modulates low-frequency seismicity on the Parkfield segment of the San Andreas Fault
Source: Nat Commun. 2026 Jun 9;17:5137. doi: 10.1038/s41467-026-74095-9 (PMC13249816; doi:10.1038/s41467-026-74095-9)
Supplement: Supplementary file 4 — Supplementary_Data_2_SSE_Figures [file 41467_2026_74095_MOESM4_ESM.zip › Supplementary_Data_2_SSE_Figures_Zali_et_al2026.pdf]

Supplementary Data 2: Strainmeter and Creepmeter Signals for All SSEs

Slow slip modulates low-frequency seismicity on the San Andreas Fault

Zahra Zali[1], *Patricia Martínez-Garzón*[1,2], *David Mencin*[3], *Gregory C. Beroza*[4]

[1]GFZ Helmholtz Centre for Geosciences, Potsdam, Germany

[2]RWTH Aachen University, Aachen, Germany

[3] EarthScope Consortium, Washington DC, U.S.A.

[4] Department of Geophysics, Stanford University, Stanford, California, USA

Correspondence to: Zahra Zali (zali@gfz.de)

Description: This document contains daily plots of the corrected strainmeter signals, wavelet transform results, and traces from three nearby creepmeters for all SSEs identified in this study (2009–2016). The figures provide visual support for the complete SSE catalog presented in Supplementary Data 1.

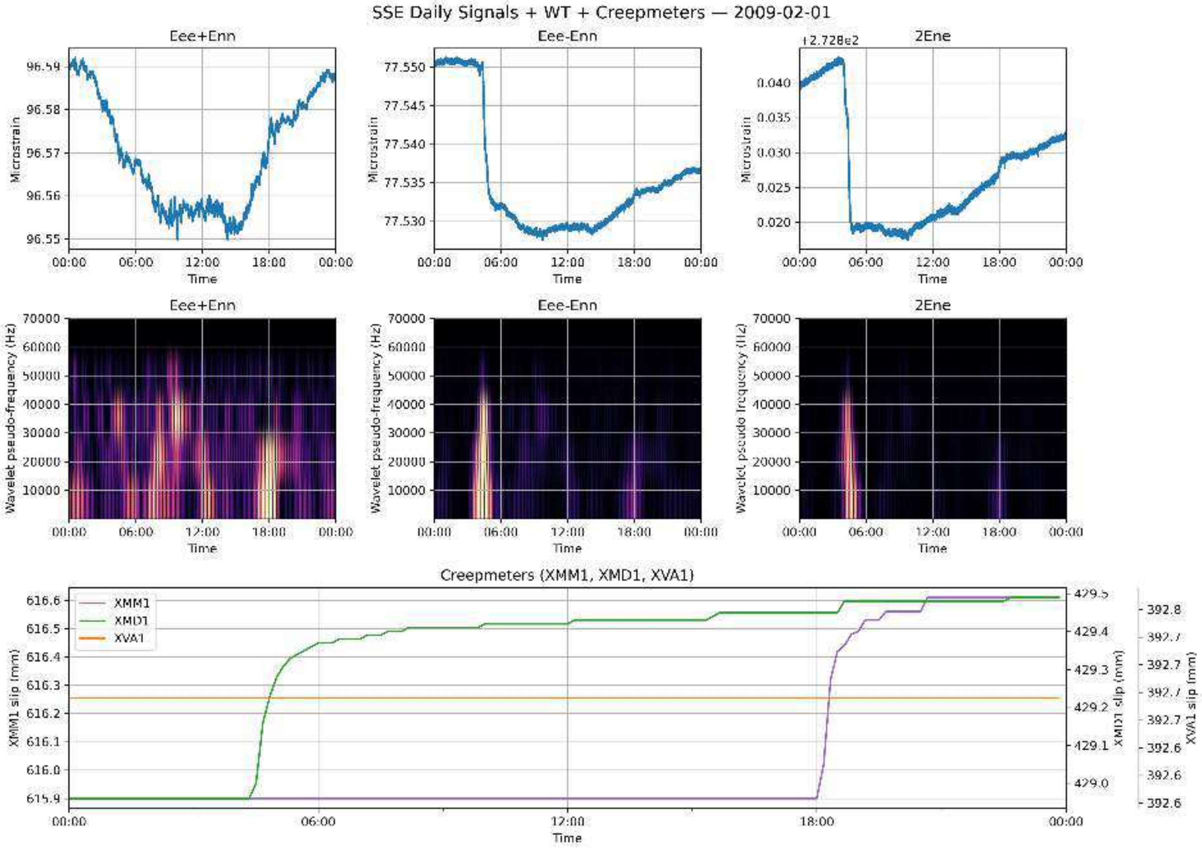
SSE Daily Signals + WT + Creepmeters — 2009-02-01
Eee+Enn
Eee-Enn
2Ene
+2.728e2
Microstrain
Time
Wavelet pseudo-frequency (Hz)
Creepmeters (XMM1, XMD1, XVA1)
XMM1
XMD1
XVA1
XMM1 slip (mm)
XMD1 slip (mm)
XVA1 slip (mm)

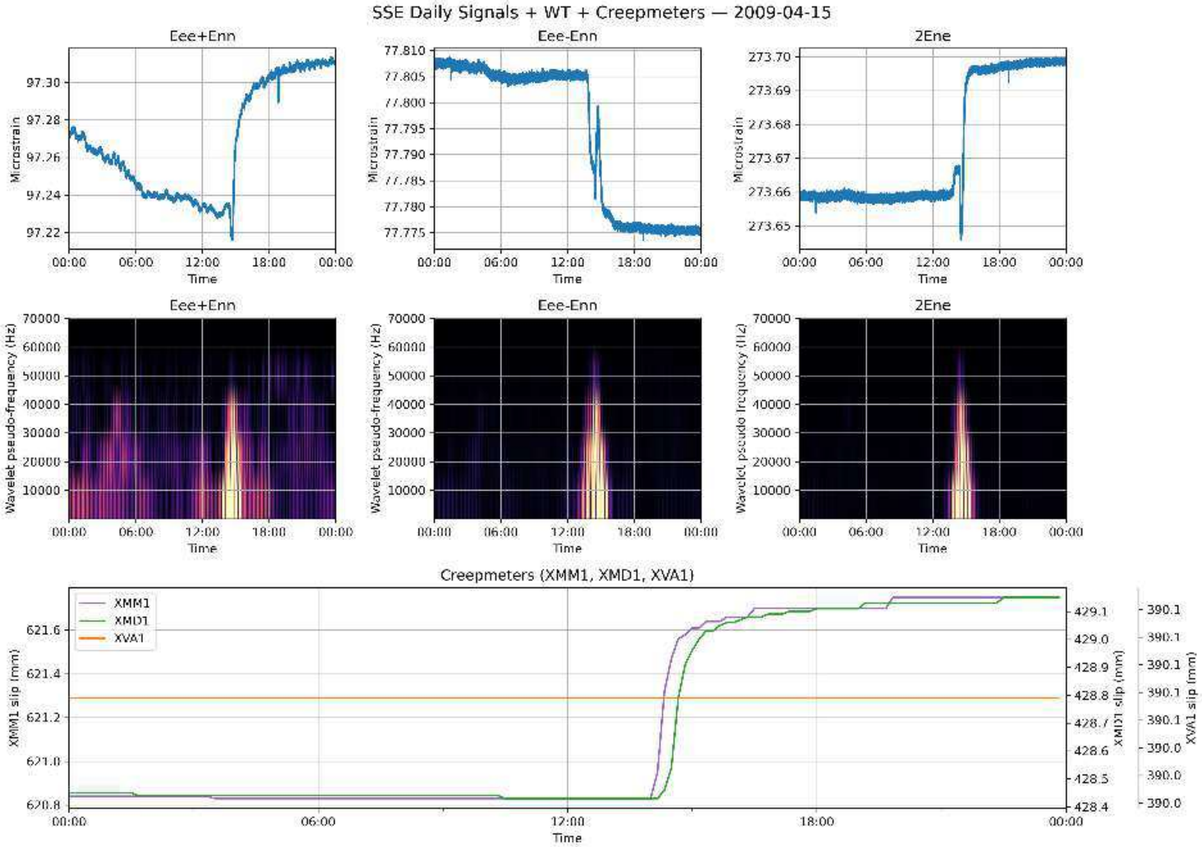
SSE Daily Signals + WT + Creepmeters — 2009-04-15
Eee+Enn
Eee-Enn
2Ene
Microstrain
Time
Wavelet pseudo-frequency (Hz)
Creepmeters (XMM1, XMD1, XVA1)
XMM1
XMD1
XVA1
XMM1 slip (mm)
XMD1 slip (mm)
XVA1 slip (mm)

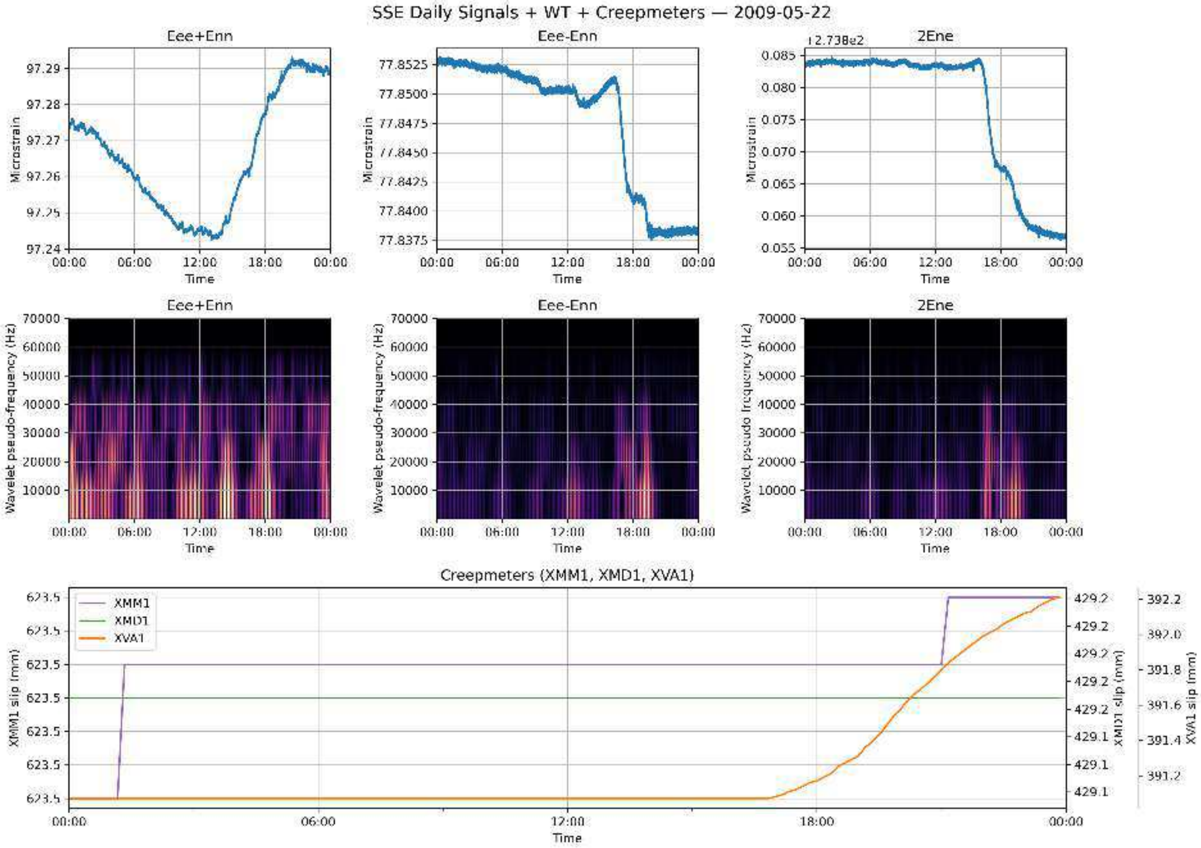
SSE Daily Signals + WT + Creepmeters — 2009-05-22
Eee+Enn
Eee-Enn
2Ene
1e2.738e2
Microstrain
Time
Wavelet pseudo-frequency (Hz)
Creepmeters (XMM1, XMD1, XVA1)
XMM1
XMD1
XVA1
XMM1 slip (mm)
XMD1 slip (mm)
XVA1 slip (mm)

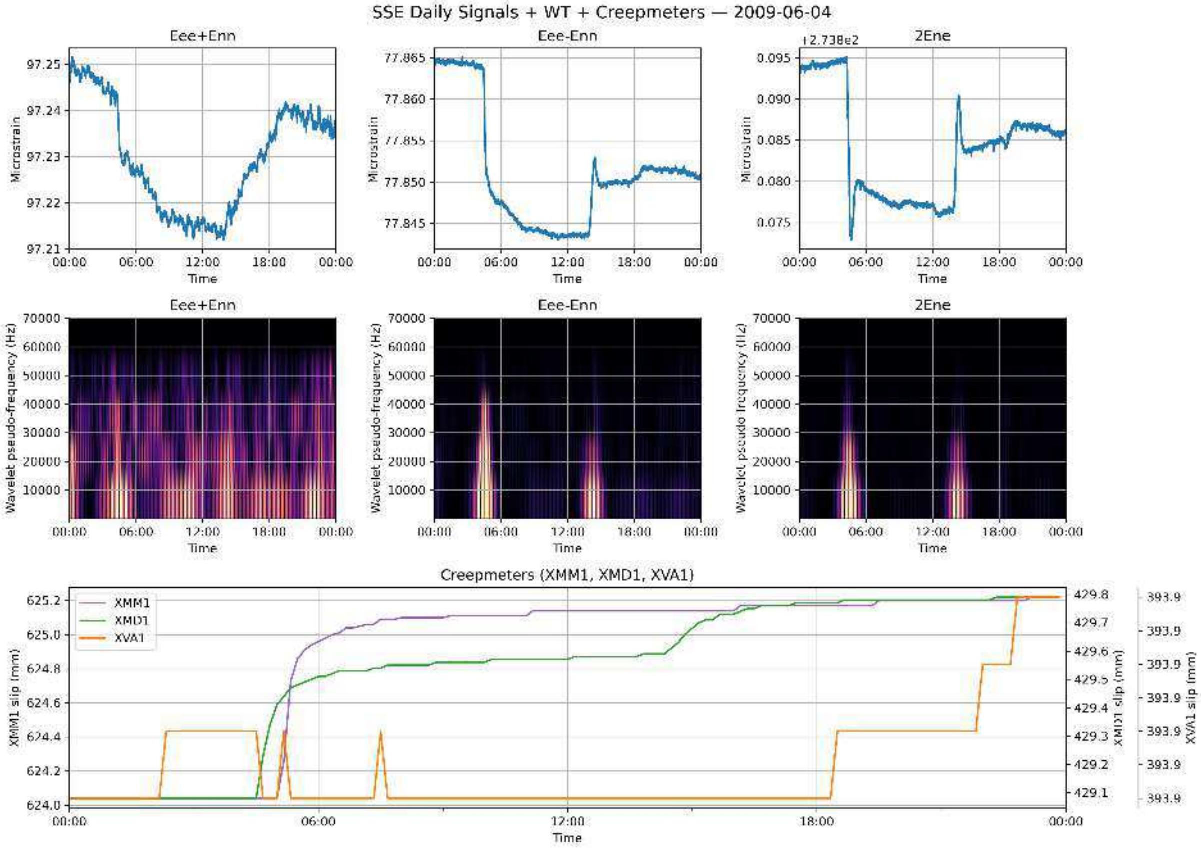
SSE Daily Signals + WT + Creepmeters — 2009-06-04
Eee+Enn
Eee-Enn
2Ene
1e2.738e2
Microstrain
Time
Wavelet pseudo-frequency (Hz)
Creepmeters (XMM1, XMD1, XVA1)
XMM1
XMD1
XVA1
XMM1 slip (mm)
XMD1 slip (mm)
XVA1 slip (mm)

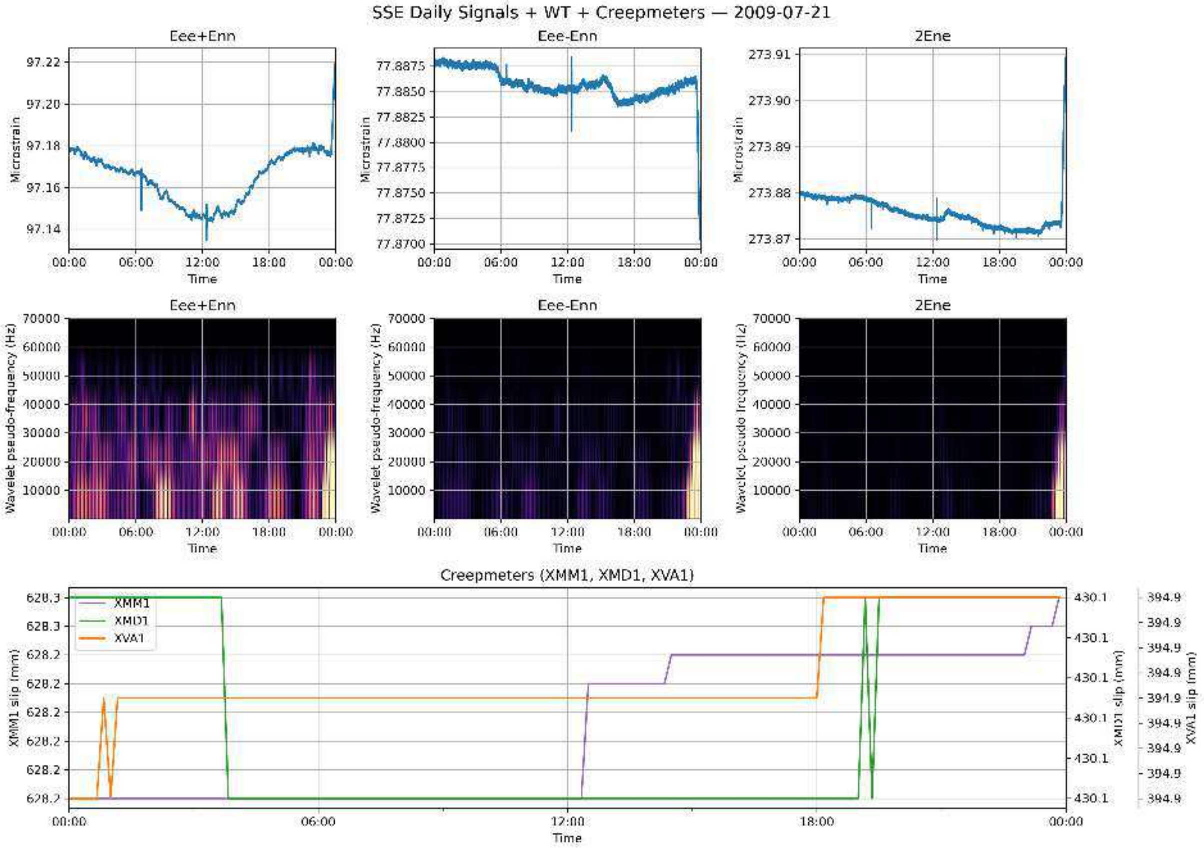
SSE Daily Signals + WT + Creepmeters — 2009-07-21
Eee+Enn
Eee-Enn
2Ene
Microstrain
Time
Wavelet pseudo-frequency (Hz)
Creepmeters (XMM1, XMD1, XVA1)
XMM1
XMD1
XVA1
XMM1 slip (mm)
XMD1 slip (mm)
XVA1 slip (mm)

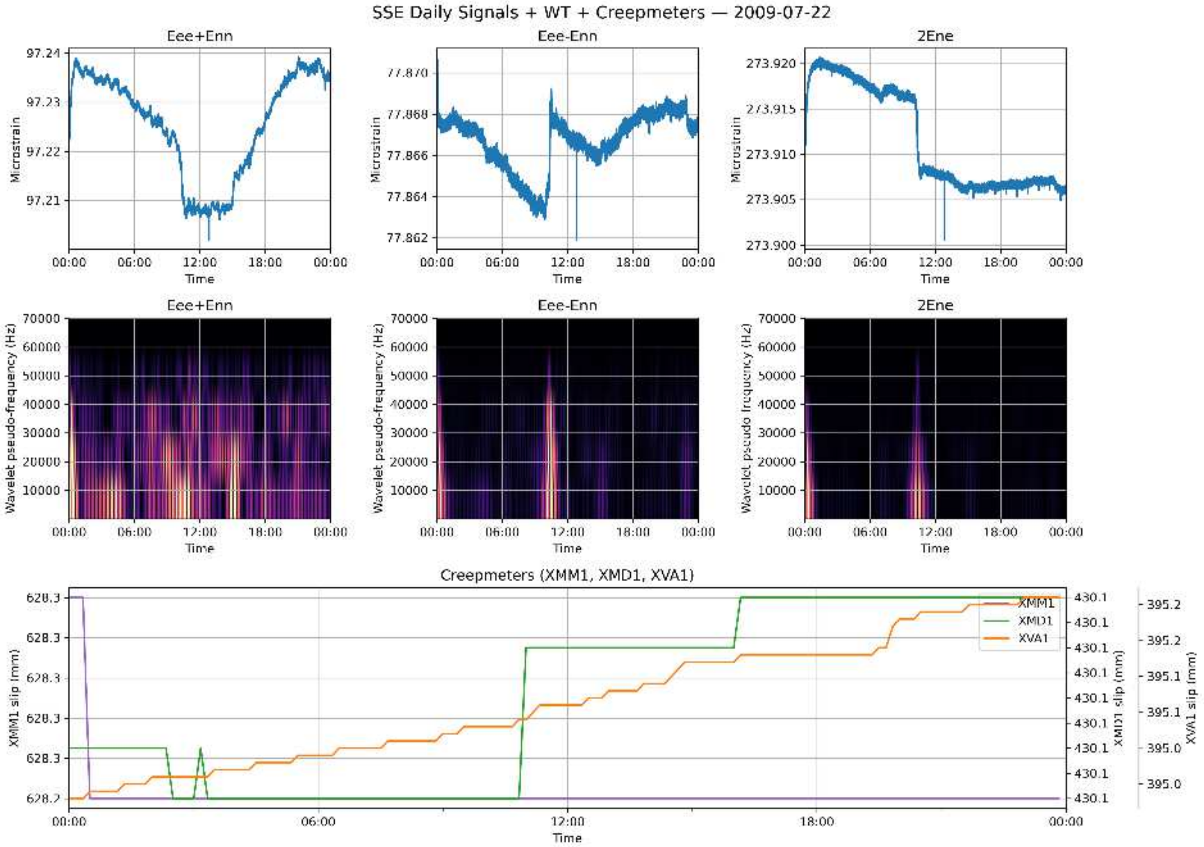
SSE Daily Signals + WT + Creepmeters — 2009-07-22
Eee+Enn
Eee-Enn
2Ene
Microstrain
Time
Wavelet pseudo-frequency (Hz)
Creepmeters (XMM1, XMD1, XVA1)
XMM1
XMD1
XVA1
XMM1 slip (mm)
XMD1 slip (mm)
XVA1 slip (mm)

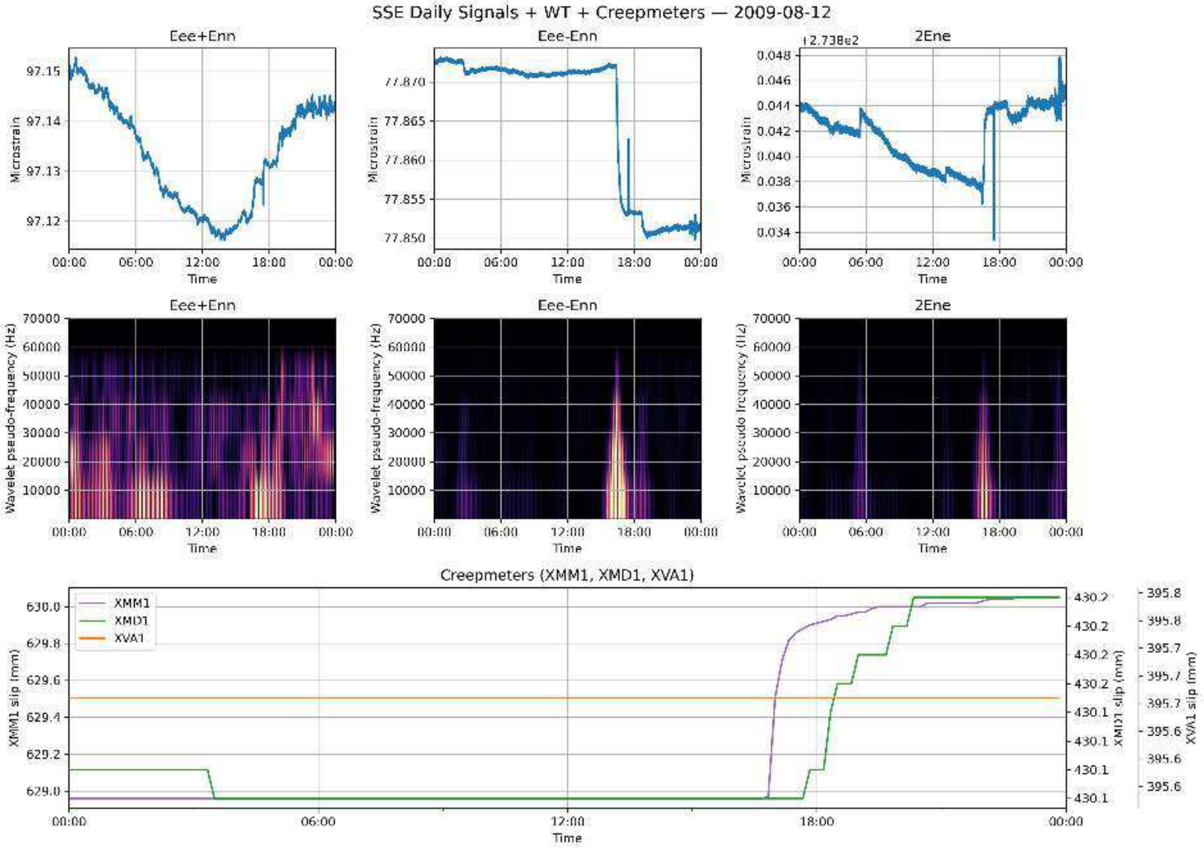
SSE Daily Signals + WT + Creepmeters — 2009-08-12
Eee+Enn
Eee-Enn
2Ene
Microstrain
Time
Wavelet pseudo-frequency (Hz)
Creepmeters (XMM1, XMD1, XVA1)
XMM1
XMD1
XVA1
XMM1 slip (mm)
XMD1 slip (mm)
XVA1 slip (mm)

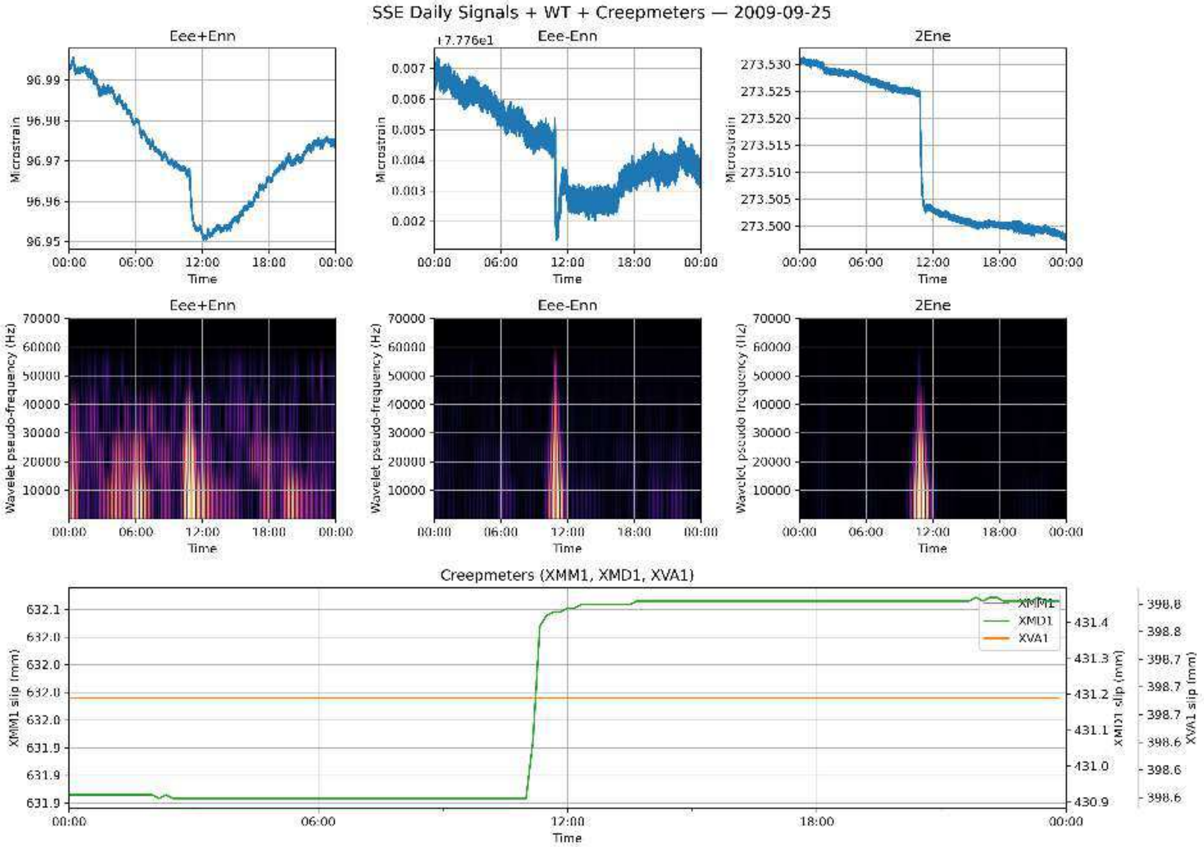
SSE Daily Signals + WT + Creepmeters — 2009-09-25
Eee+Enn
Eee-Enn
2Ene
Microstrain
Time
Wavelet pseudo-frequency (Hz)
Creepmeters (XMM1, XMD1, XVA1)
XMM1
XMD1
XVA1
XMM1 slip (mm)
XMD1 slip (mm)
XVA1 slip (mm)

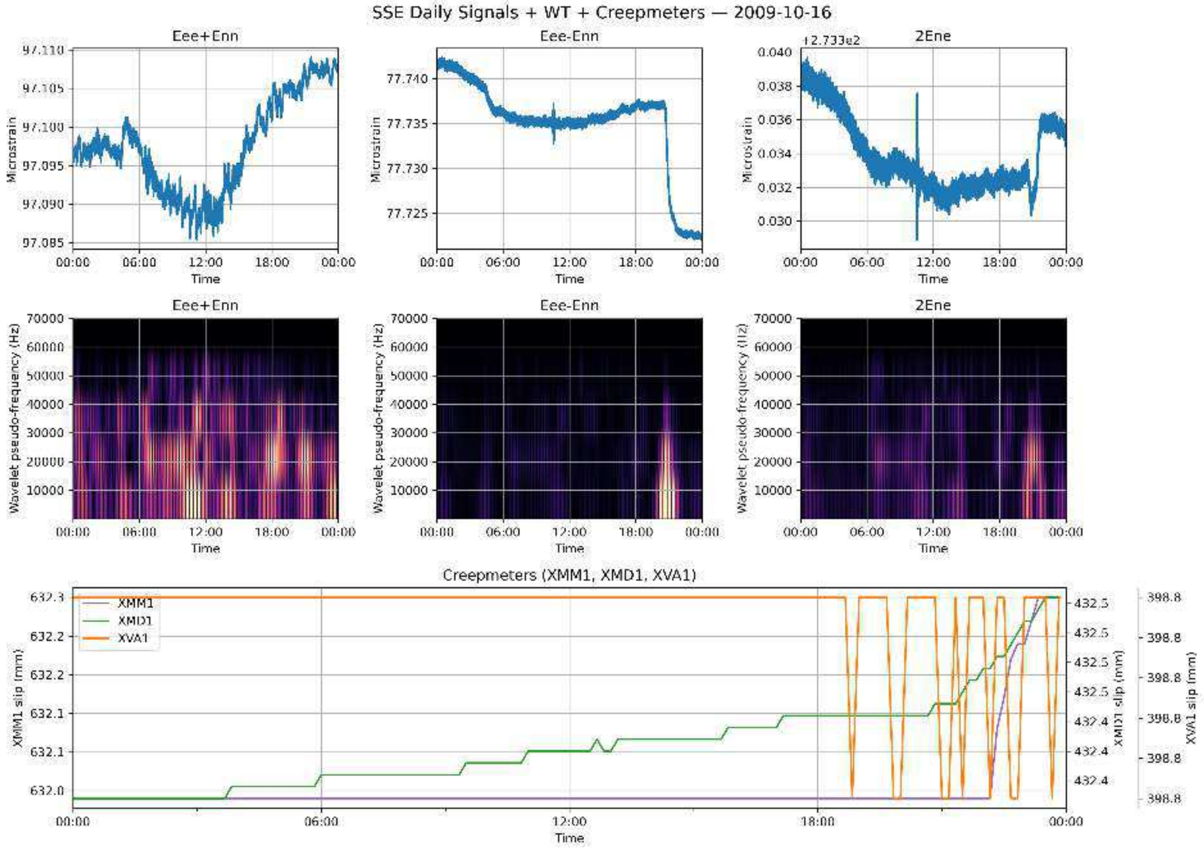
SSE Daily Signals + WT + Creepmeters — 2009-10-16
Eee+Enn
Eee-Enn
2Ene
Microstrain
Time
Wavelet pseudo-frequency (Hz)
Creepmeters (XMM1, XMD1, XVA1)
XMM1
XMD1
XVA1
XMM1 slip (mm)
XMD1 slip (mm)
XVA1 slip (mm)

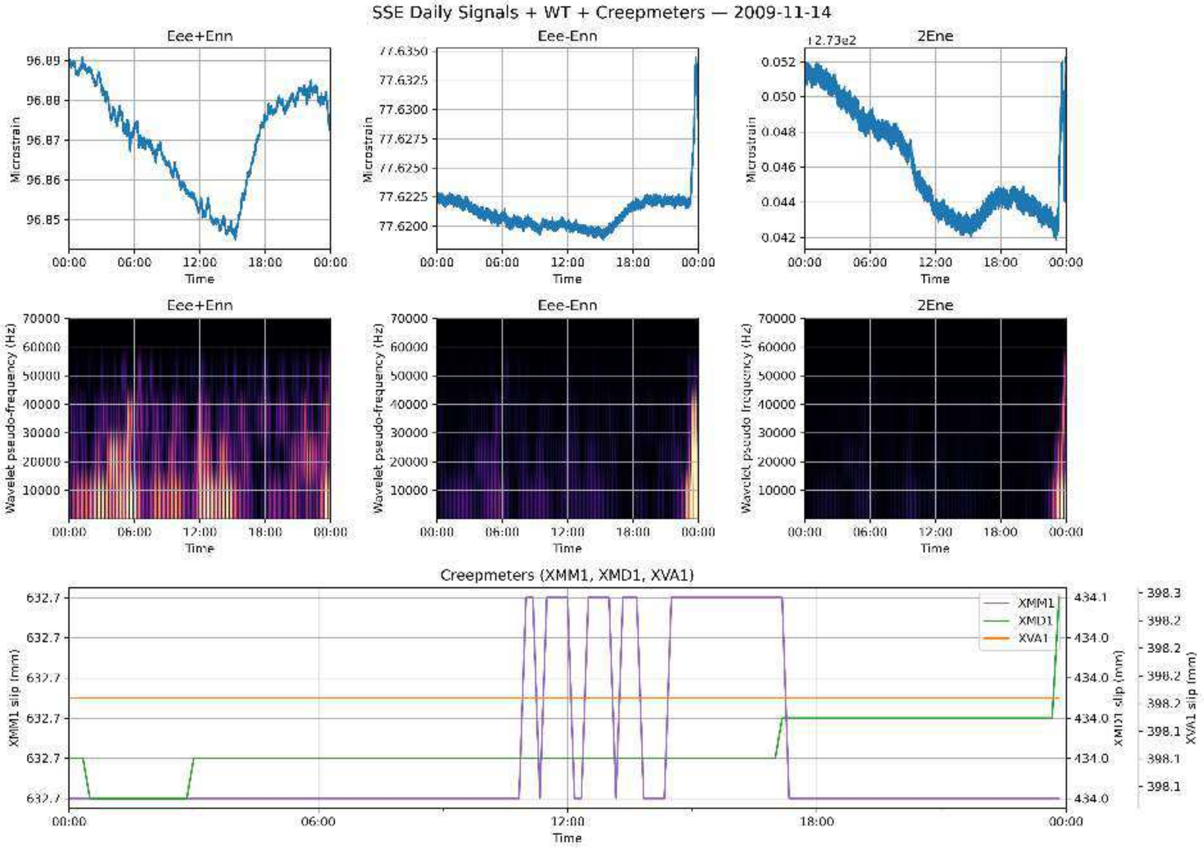
SSE Daily Signals + WT + Creepmeters — 2009-11-14
Eee+Enn
Eee-Enn
2Ene
Microstrain
Time
Wavelet pseudo-frequency (Hz)
Creepmeters (XMM1, XMD1, XVA1)
XMM1
XMD1
XVA1
XMM1 slip (mm)
XMD1 slip (mm)
XVA1 slip (mm)

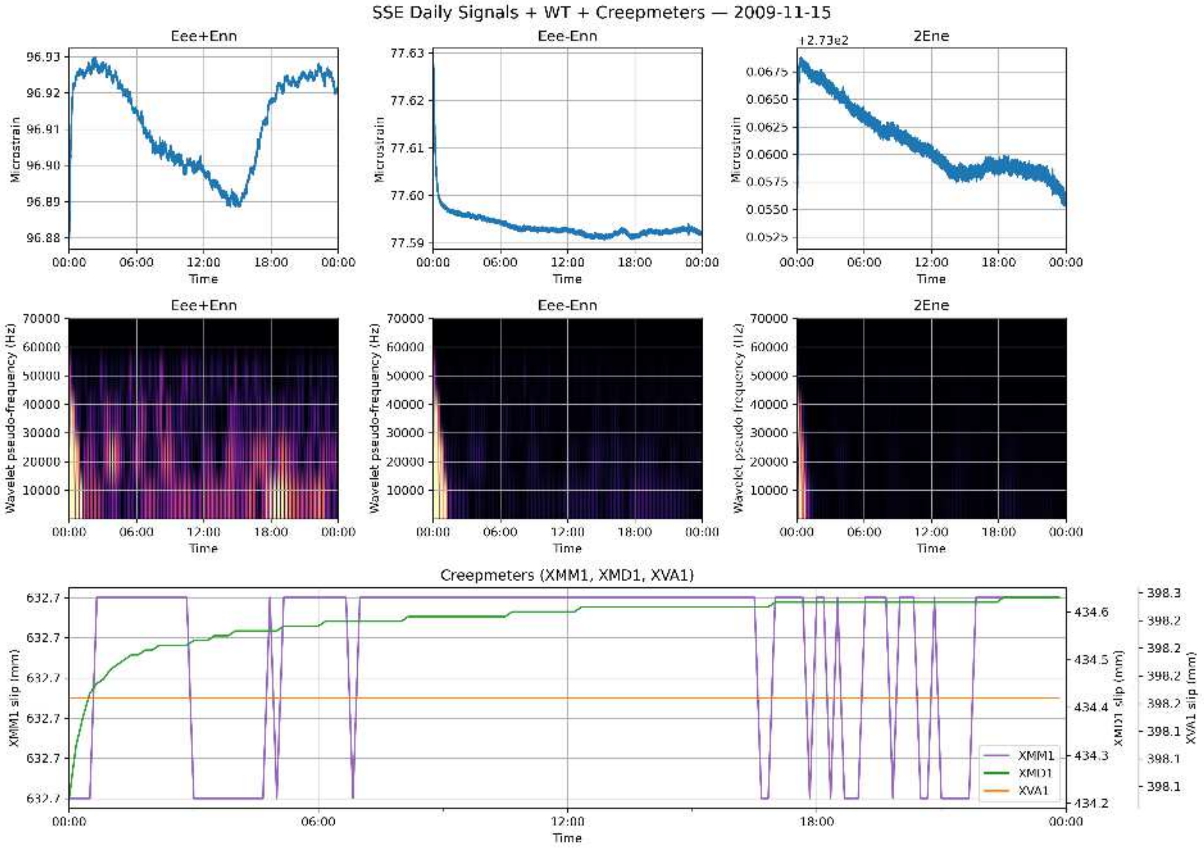
SSE Daily Signals + WT + Creepmeters — 2009-11-15
Eee+Enn
Eee-Enn
2Ene
Microstrain
Time
Wavelet pseudo-frequency (Hz)
Creepmeters (XMM1, XMD1, XVA1)
XMM1 slip (mm)
XMD1 slip (mm)
XVA1 slip (mm)
XMM1
XMD1
XVA1

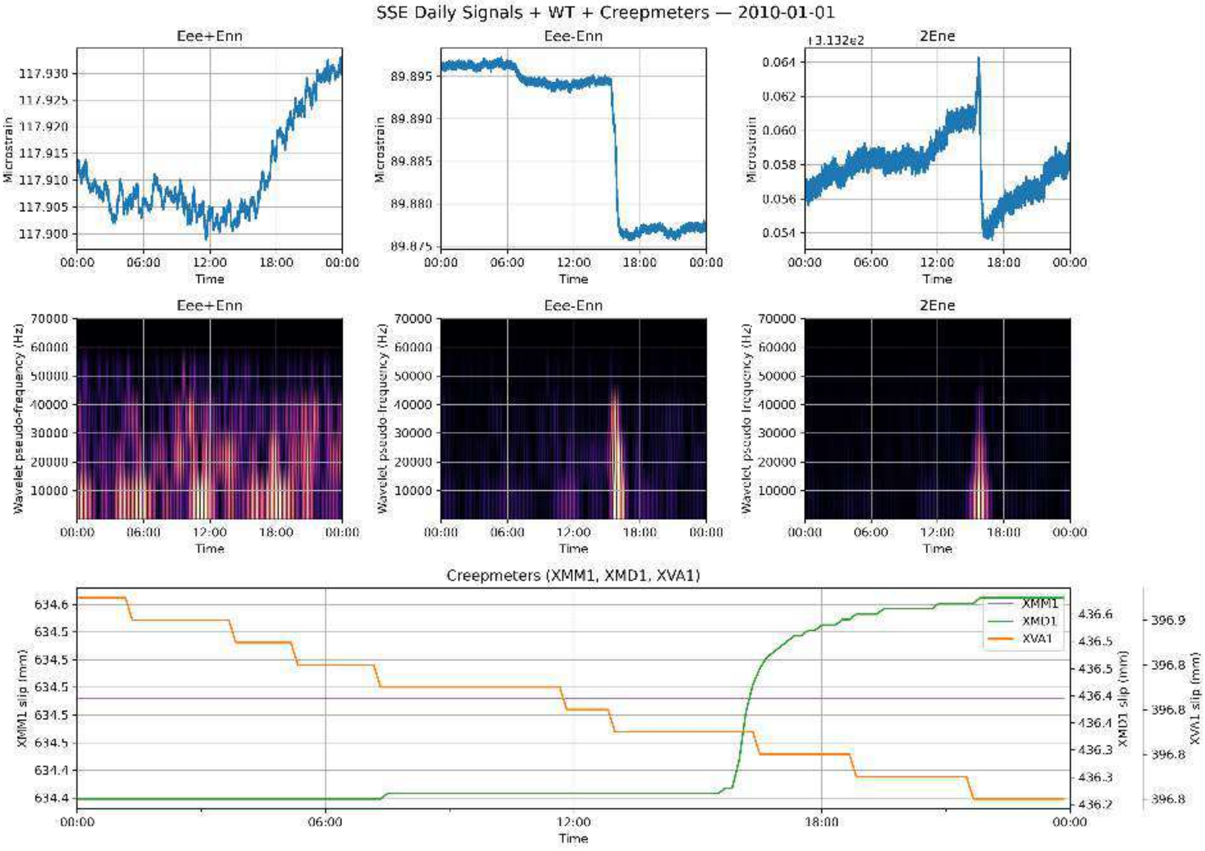
SSE Daily Signals + WT + Creepmeters — 2010-01-01
Eee+Enn
Eee-Enn
2Ene
Microstrain
Time
Wavelet pseudo-frequency (Hz)
Creepmeters (XMM1, XMD1, XVA1)
XMM1 slip (mm)
XMD1 slip (mm)
XVA1 slip (mm)
XMM1
XMD1
XVA1

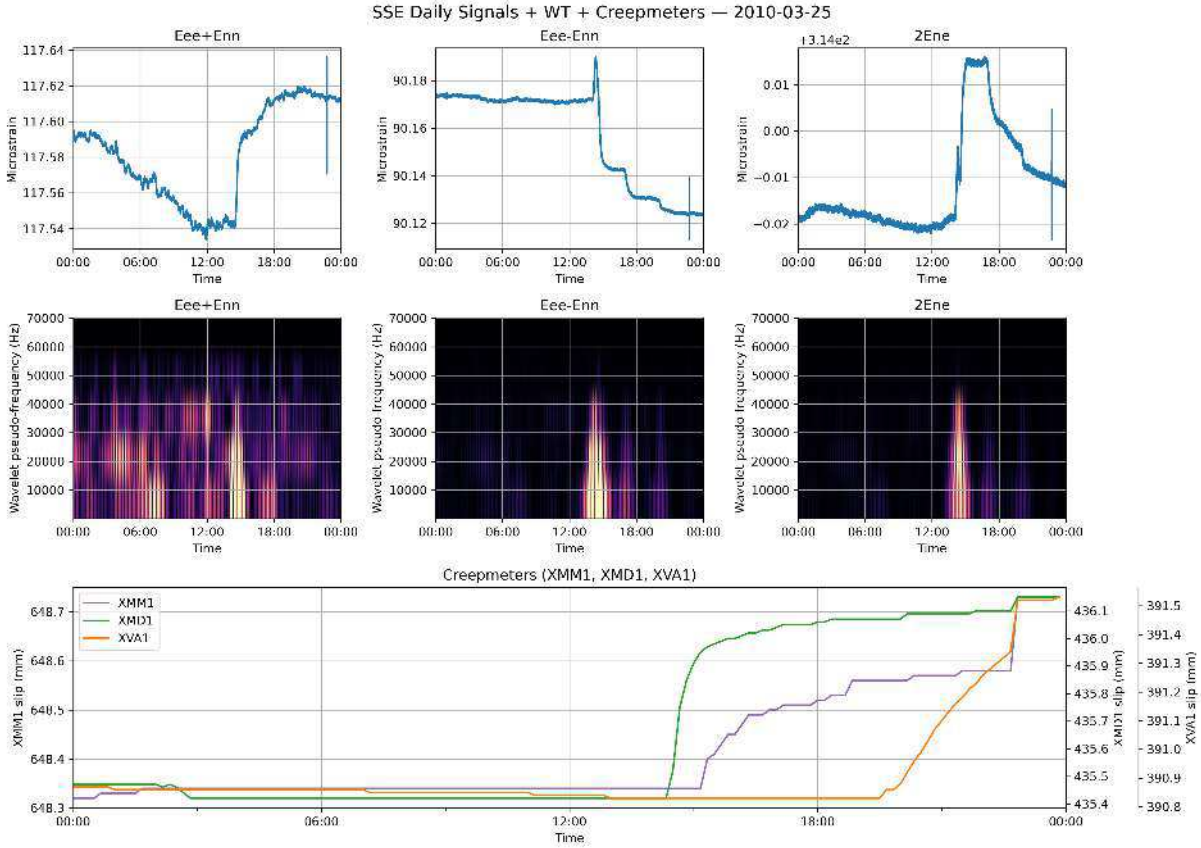
SSE Daily Signals + WT + Creepmeters — 2010-03-25
Eee+Enn
Eee-Enn
2Ene
Microstrain
Time
Wavelet pseudo-frequency (Hz)
Creepmeters (XMM1, XMD1, XVA1)
XMM1
XMD1
XVA1
XMM1 slip (mm)
XMD1 slip (mm)
XVA1 slip (mm)

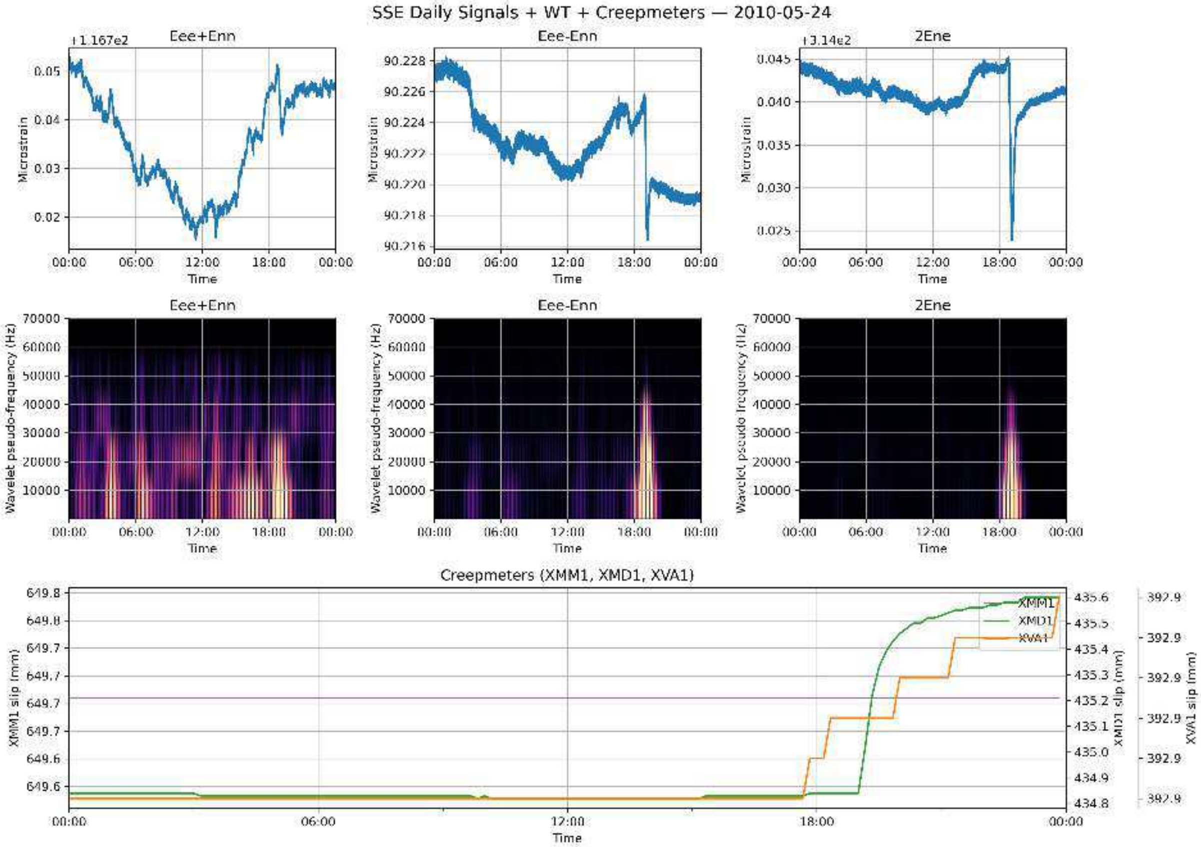
SSE Daily Signals + WT + Creepmeters — 2010-05-24
Eee+Enn
Eee-Enn
2Ene
Microstrain
Time
Wavelet pseudo-frequency (Hz)
Creepmeters (XMM1, XMD1, XVA1)
XMM1
XMD1
XVA1
XMM1 slip (mm)
XMD1 slip (mm)
XVA1 slip (mm)

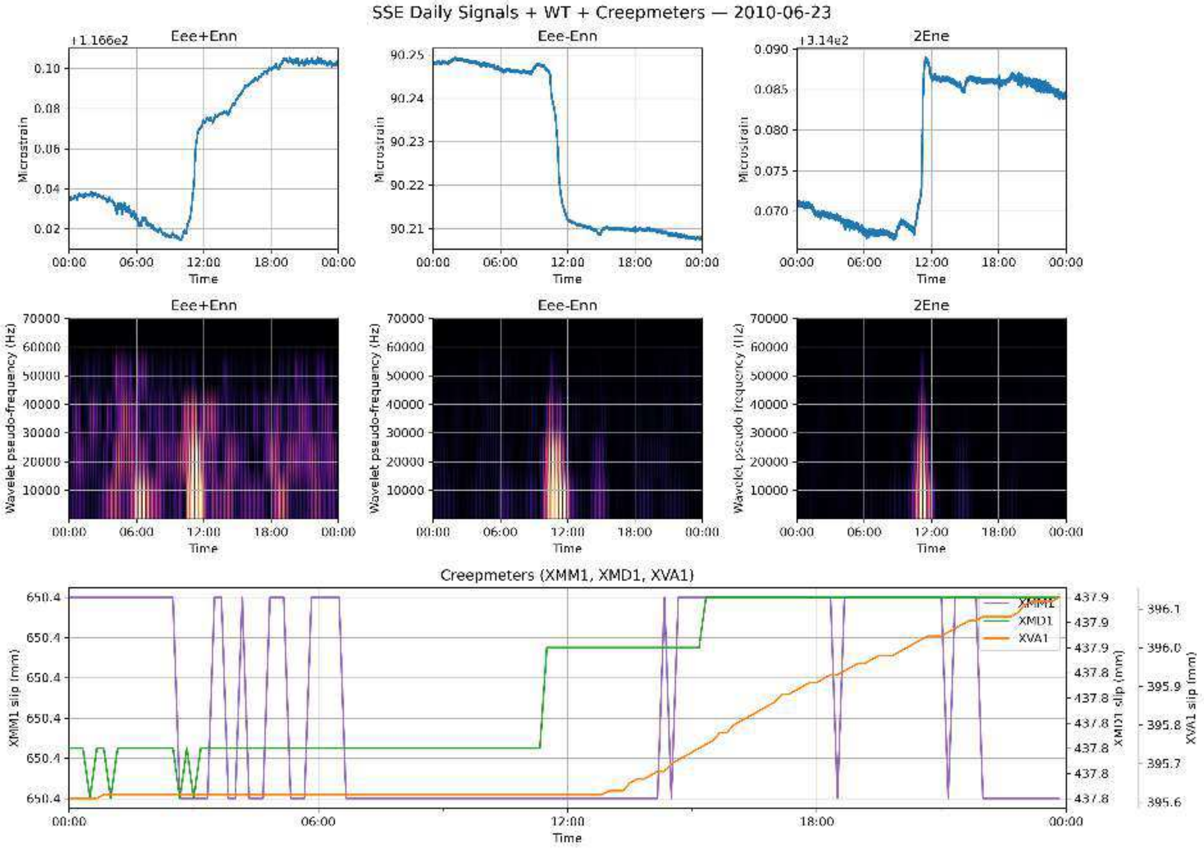

SSE Daily Signals + WT + Creepmeters — 2010-06-23
Eee+Enn
Eee-Enn
2Ene
Microstrain
Time
Wavelet pseudo-frequency (Hz)
Creepmeters (XMM1, XMD1, XVA1)
XMM1 slip (mm)
XMD1 slip (mm)
XVA1 slip (mm)
XMM1
XMD1
XVA1

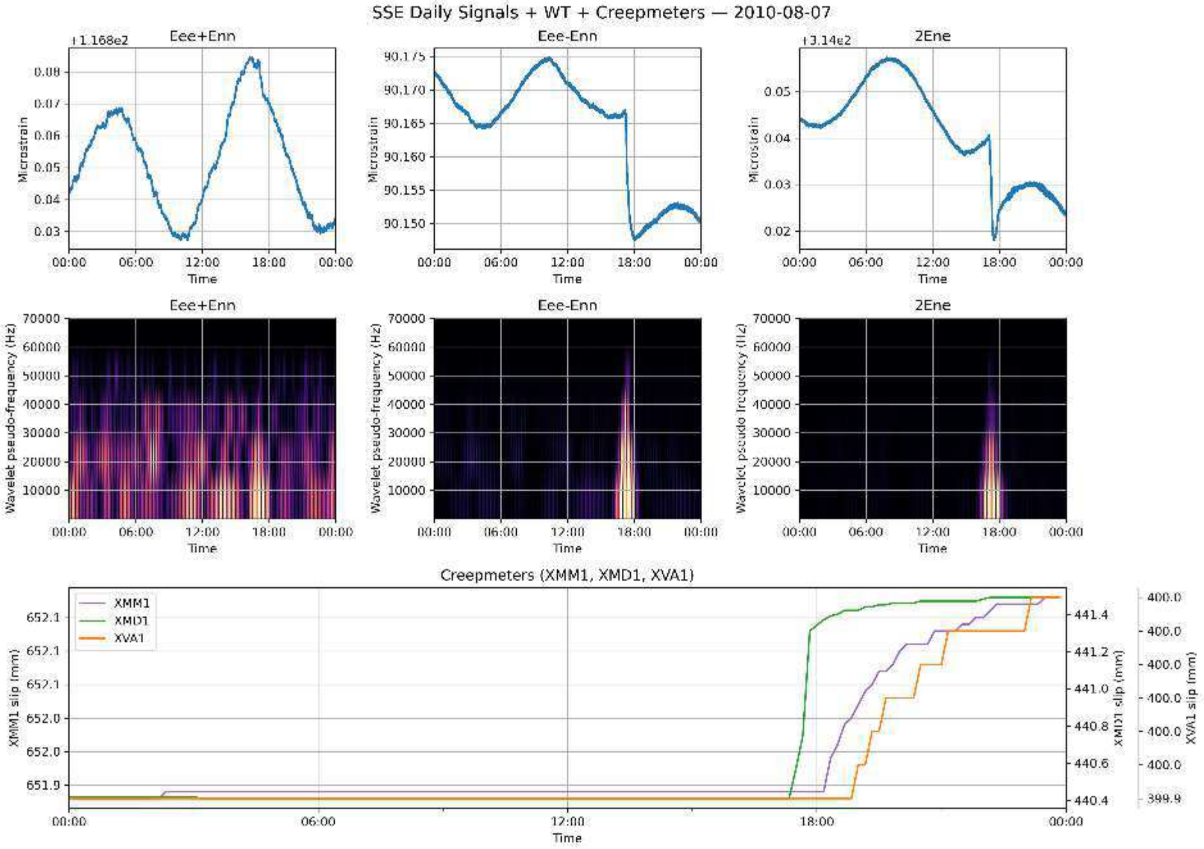

SSE Daily Signals + WT + Creepmeters — 2010-08-07
Eee+Enn
Eee-Enn
2Ene
Microstrain
Time
Wavelet pseudo-frequency (Hz)
Creepmeters (XMM1, XMD1, XVA1)
XMM1 slip (mm)
XMD1 slip (mm)
XVA1 slip (mm)
XMM1
XMD1
XVA1

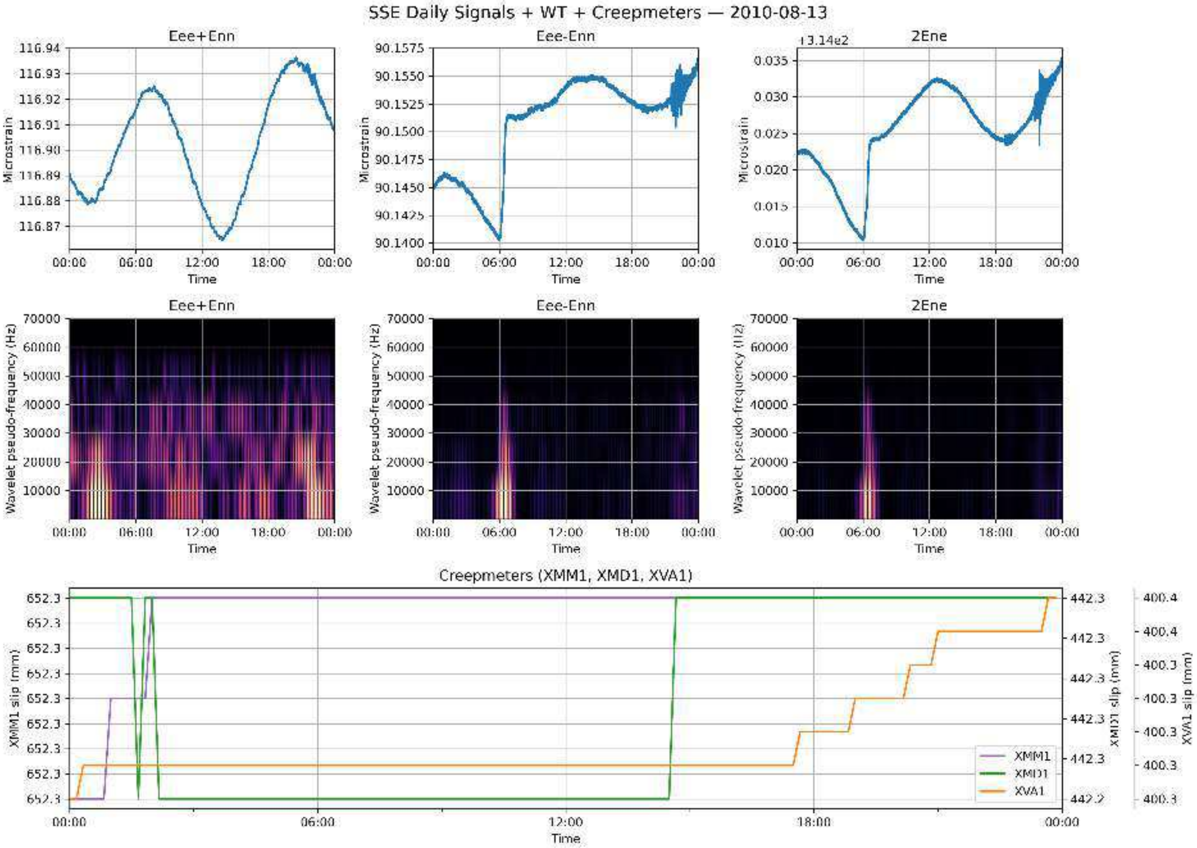
SSE Daily Signals + WT + Creepmeters — 2010-08-13
Eee+Enn
Eee-Enn
2Ene
1e2+3.14e2
Microstrain
Time
Wavelet pseudo-frequency (Hz)
Creepmeters (XMM1, XMD1, XVA1)
XMM1 slip (mm)
XMD1 slip (mm)
XVA1 slip (mm)
XMM1
XMD1
XVA1

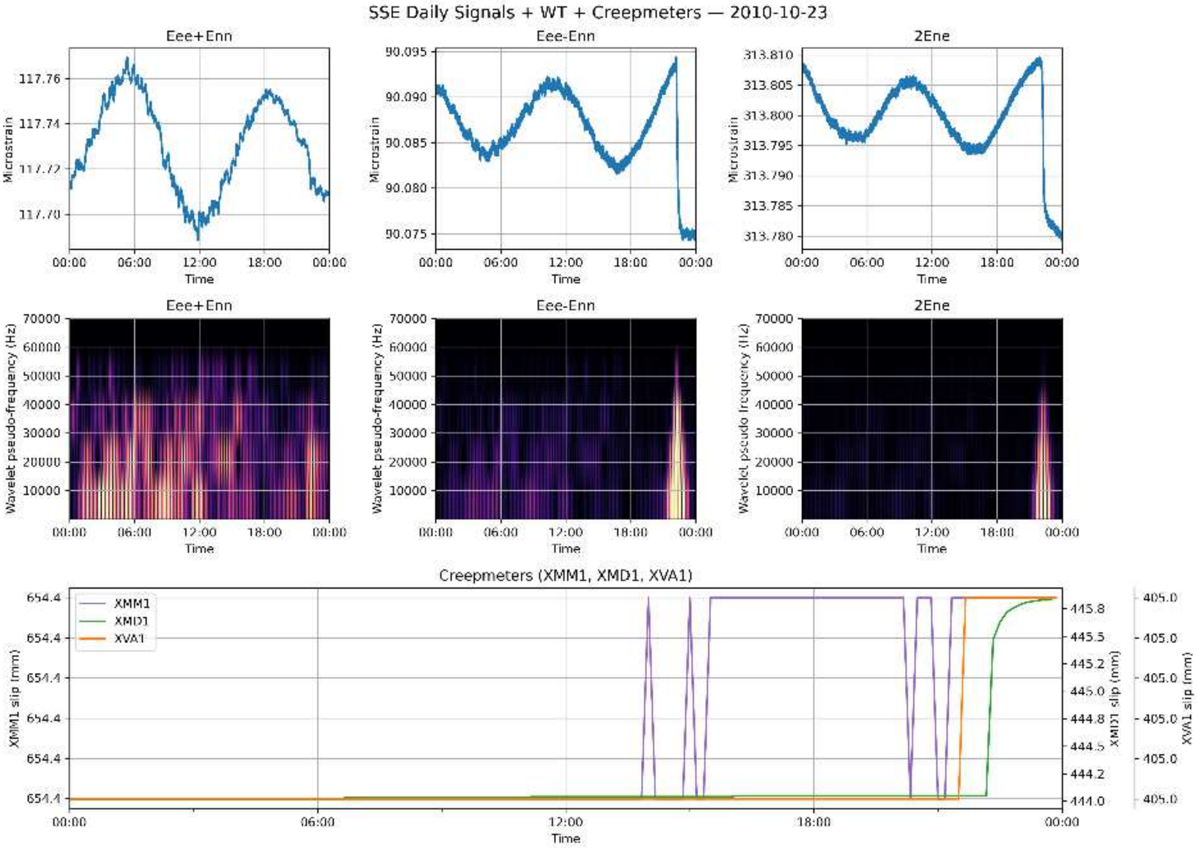
SSE Daily Signals + WT + Creepmeters — 2010-10-23
Eee+Enn
Eee-Enn
2Ene
Microstrain
Time
Wavelet pseudo-frequency (Hz)
Creepmeters (XMM1, XMD1, XVA1)
XMM1 slip (mm)
XMD1 slip (mm)
XVA1 slip (mm)
XMM1
XMD1
XVA1

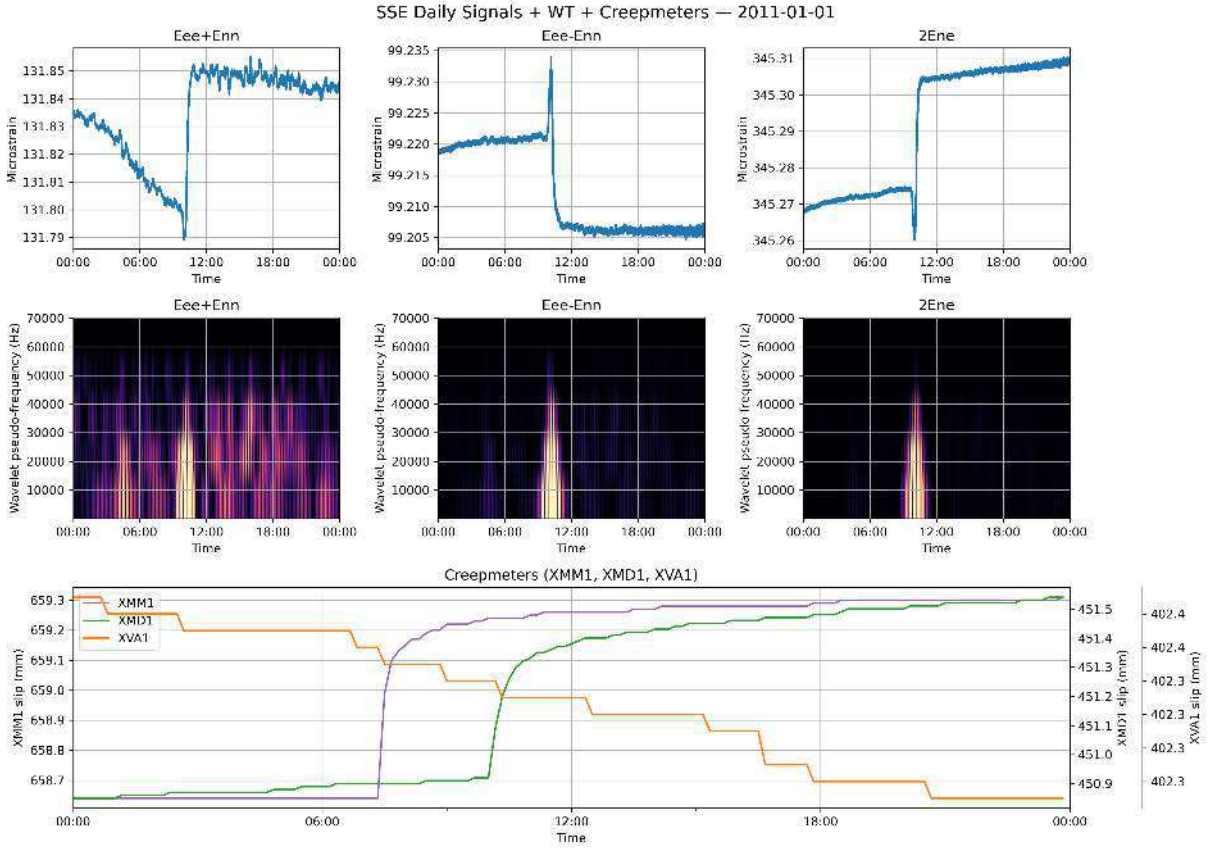
SSE Daily Signals + WT + Creepmeters — 2011-01-01
Eee+Enn
Eee-Enn
2Ene
Microstrain
Time
Wavelet pseudo-frequency (Hz)
Creepmeters (XMM1, XMD1, XVA1)
XMM1
XMD1
XVA1
XMM1 slip (mm)
XMD1 slip (mm)
XVA1 slip (mm)

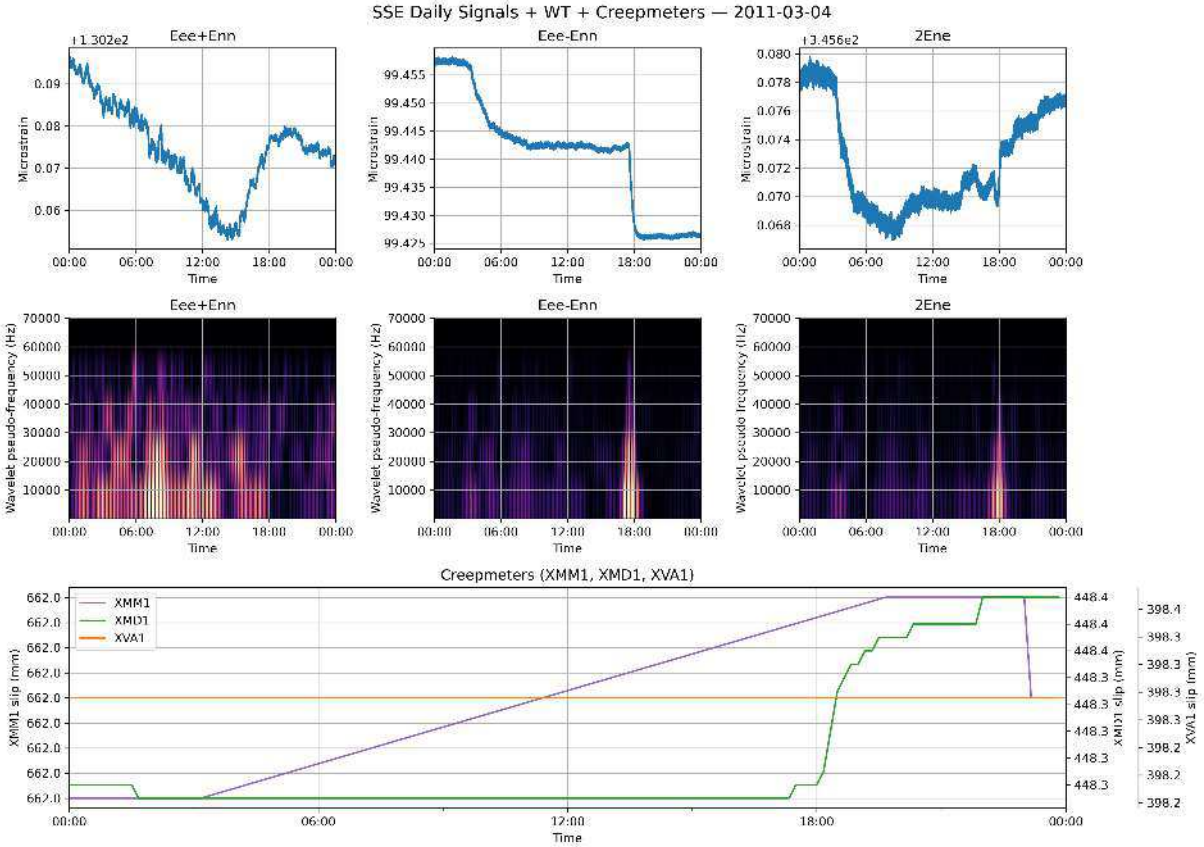
SSE Daily Signals + WT + Creepmeters — 2011-03-04
+1.302e2
+3.456e2
Eee+Enn
Eee-Enn
2Ene
Microstrain
Time
Wavelet pseudo-frequency (Hz)
Creepmeters (XMM1, XMD1, XVA1)
XMM1
XMD1
XVA1
XMM1 slip (mm)
XMD1 slip (mm)
XVA1 slip (mm)

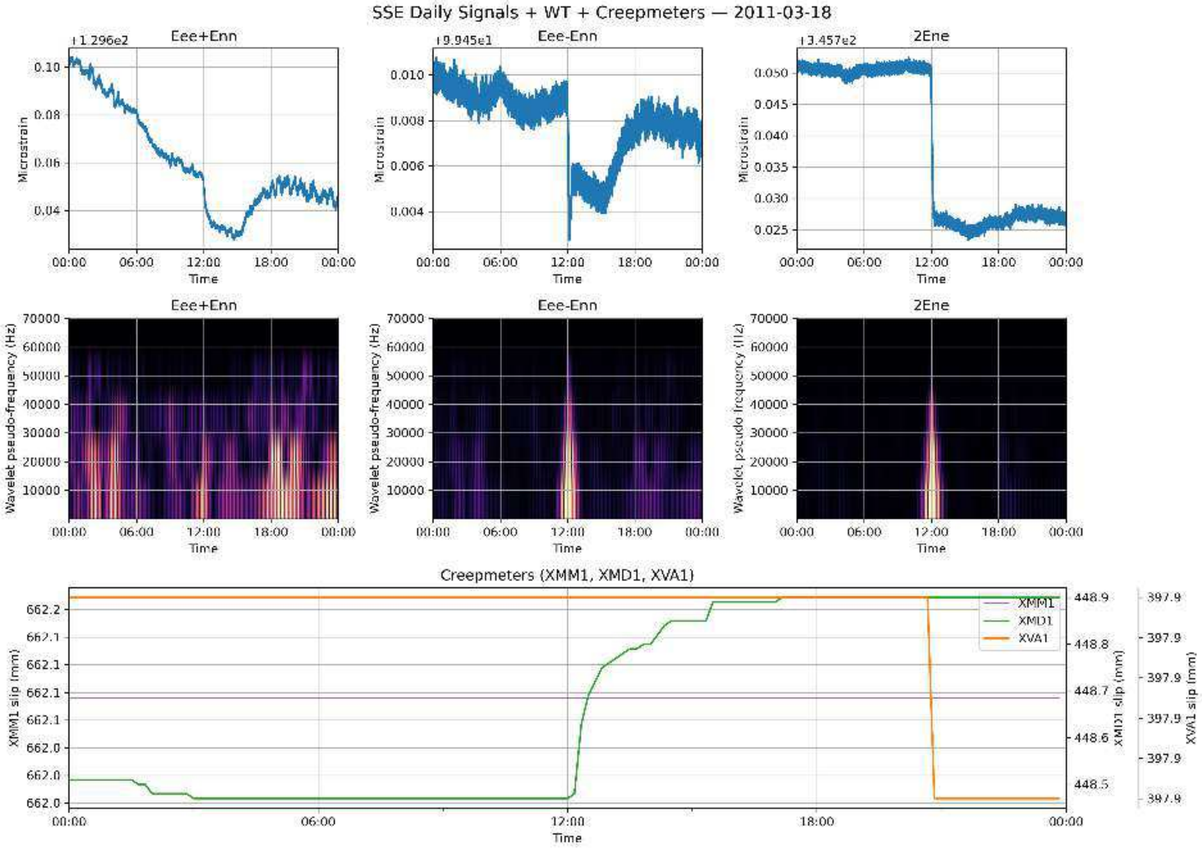
SSE Daily Signals + WT + Creepmeters — 2011-03-18
Eee+Enn
Eee-Enn
2Ene
Microstrain
Time
Wavelet pseudo-frequency (Hz)
Creepmeters (XMM1, XMD1, XVA1)
XMM1
XMD1
XVA1
XMM1 slip (mm)
XMD1 slip (mm)
XVA1 slip (mm)

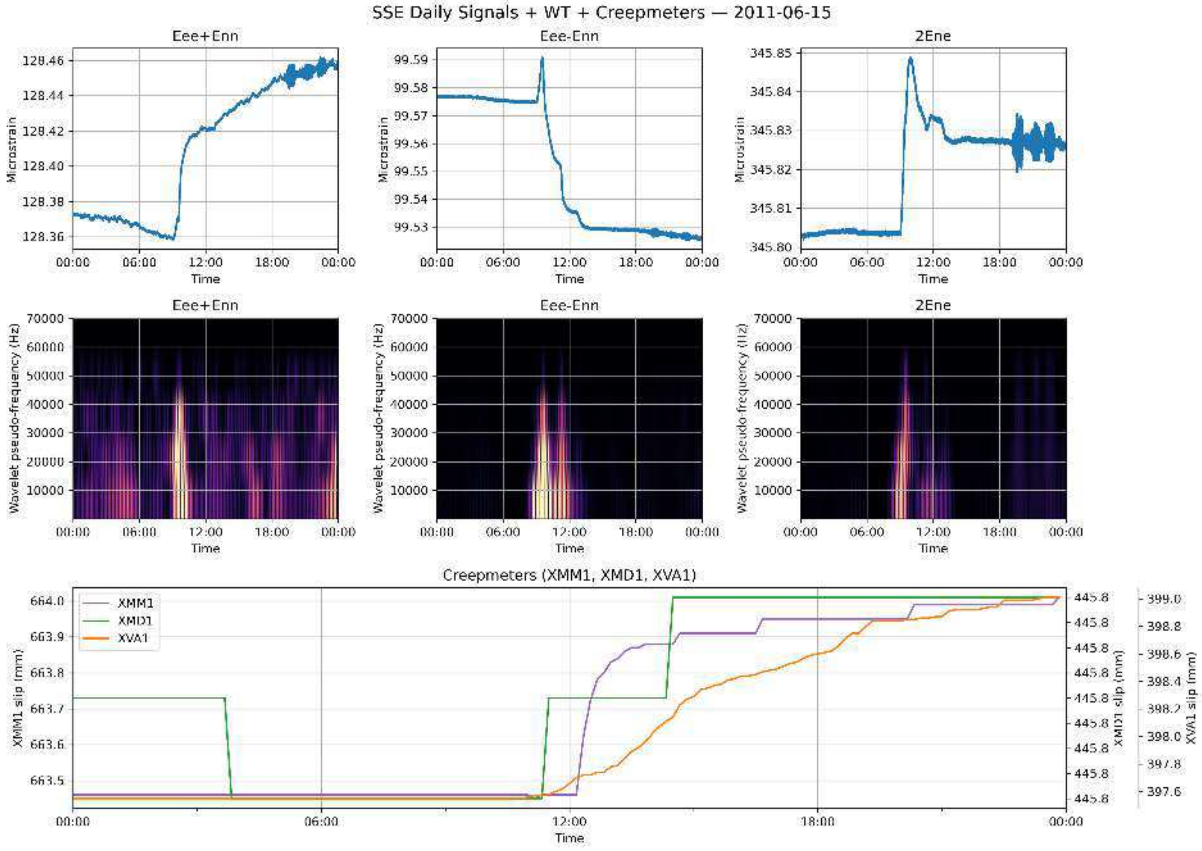
SSE Daily Signals + WT + Creepmeters — 2011-06-15
Eee+Enn
Eee-Enn
2Ene
Microstrain
Time
Wavelet pseudo-frequency (Hz)
Creepmeters (XMM1, XMD1, XVA1)
XMM1
XMD1
XVA1
XMM1 slip (mm)
XMD1 slip (mm)
XVA1 slip (mm)

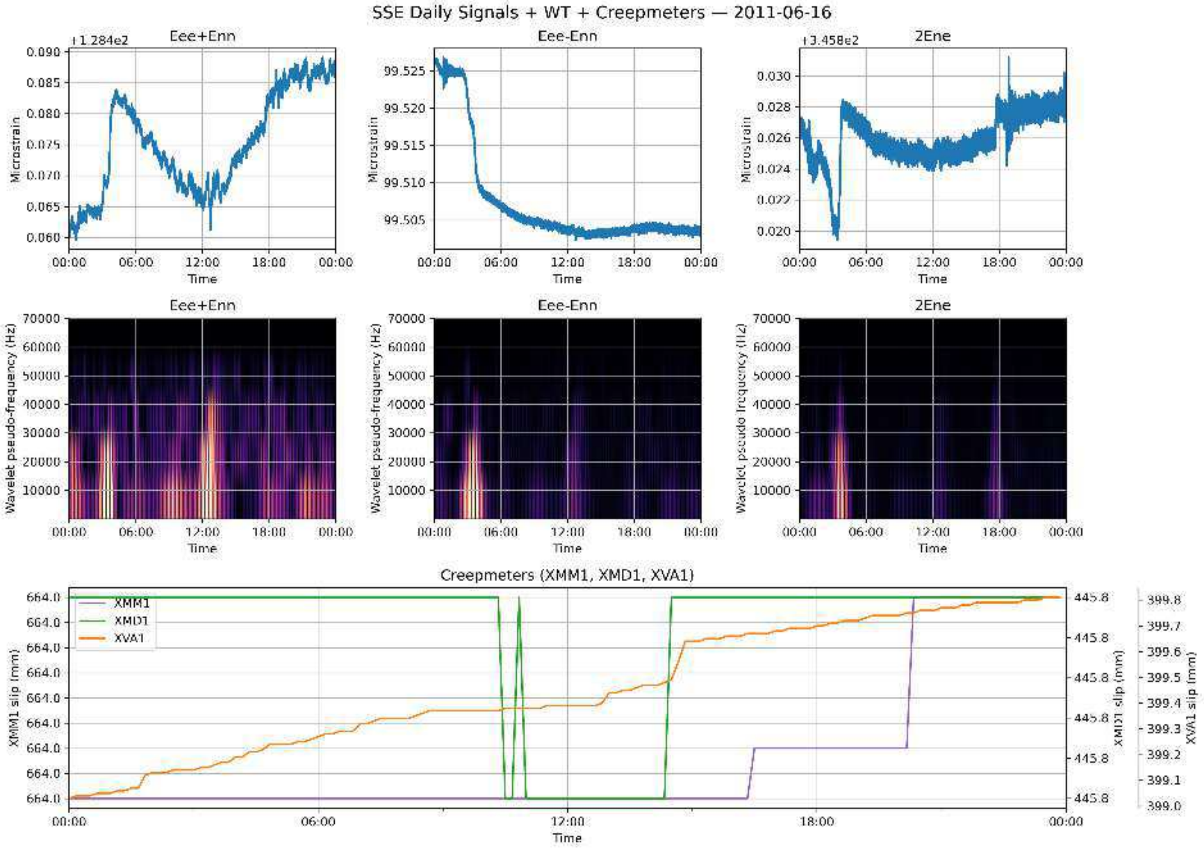
SSE Daily Signals + WT + Creepmeters — 2011-06-16
Eee+Enn
Eee-Enn
2Ene
+1.284e2
+3.458e2
Microstrain
Time
Wavelet pseudo-frequency (Hz)
Creepmeters (XMM1, XMD1, XVA1)
XMM1
XMD1
XVA1
XMM1 slip (mm)
XMD1 slip (mm)
XVA1 slip (mm)

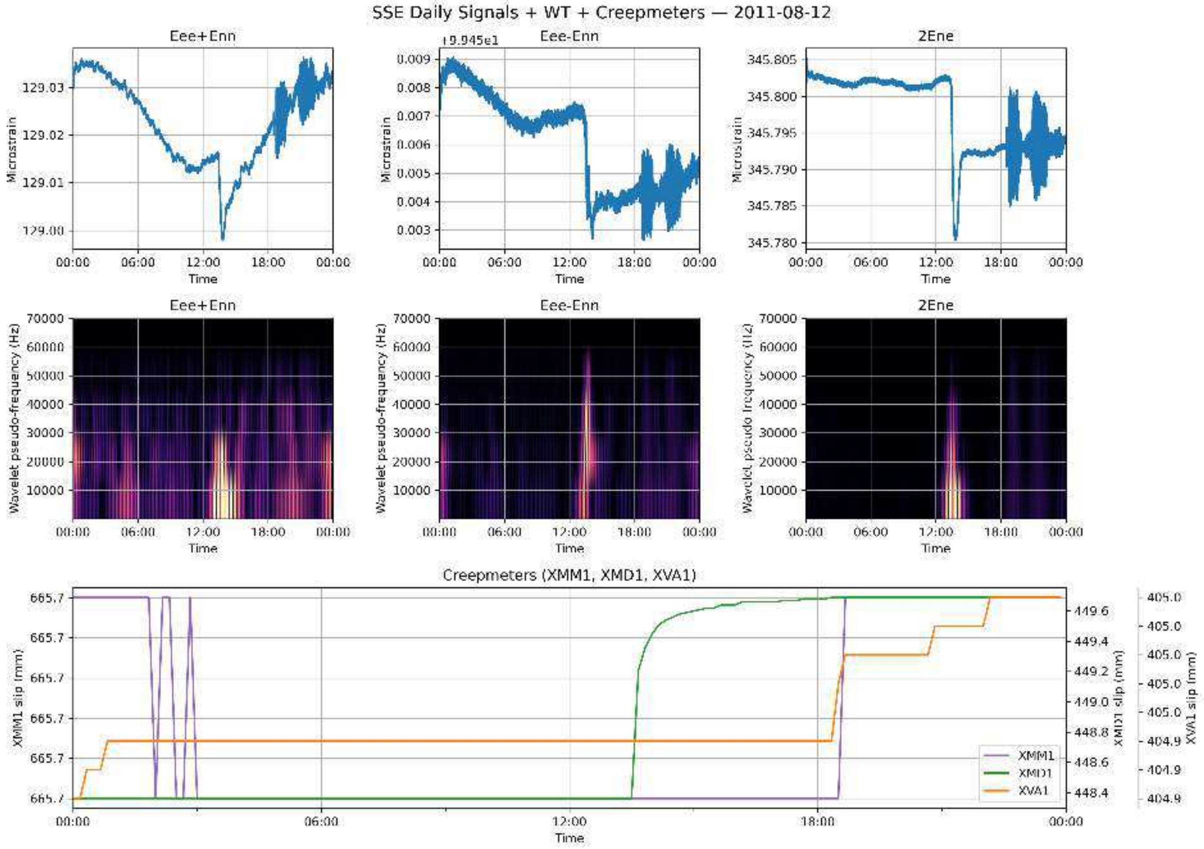
SSE Daily Signals + WT + Creepmeters — 2011-08-12
Eee+Enn
Eee-Enn
2Ene
+9.945e1
Microstrain
Time
Wavelet pseudo-frequency (Hz)
Creepmeters (XMM1, XMD1, XVA1)
XMM1
XMD1
XVA1
XMM1 slip (mm)
XMD1 slip (mm)
XVA1 slip (mm)

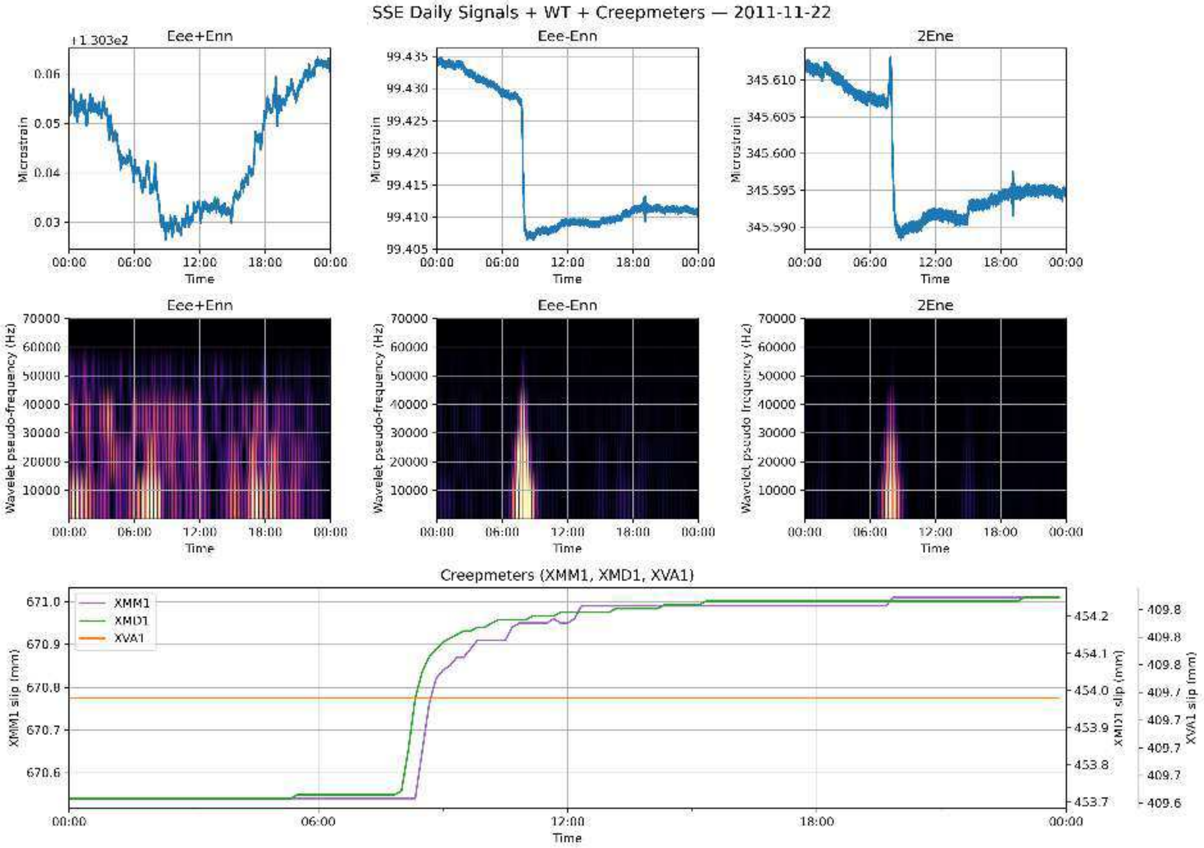
SSE Daily Signals + WT + Creepmeters — 2011-11-22
Eee+Enn
Eee-Enn
2Ene
Microstrain
Time
Wavelet pseudo-frequency (Hz)
Creepmeters (XMM1, XMD1, XVA1)
XMM1
XMD1
XVA1
XMM1 slip (mm)
XMD1 slip (mm)
XVA1 slip (mm)

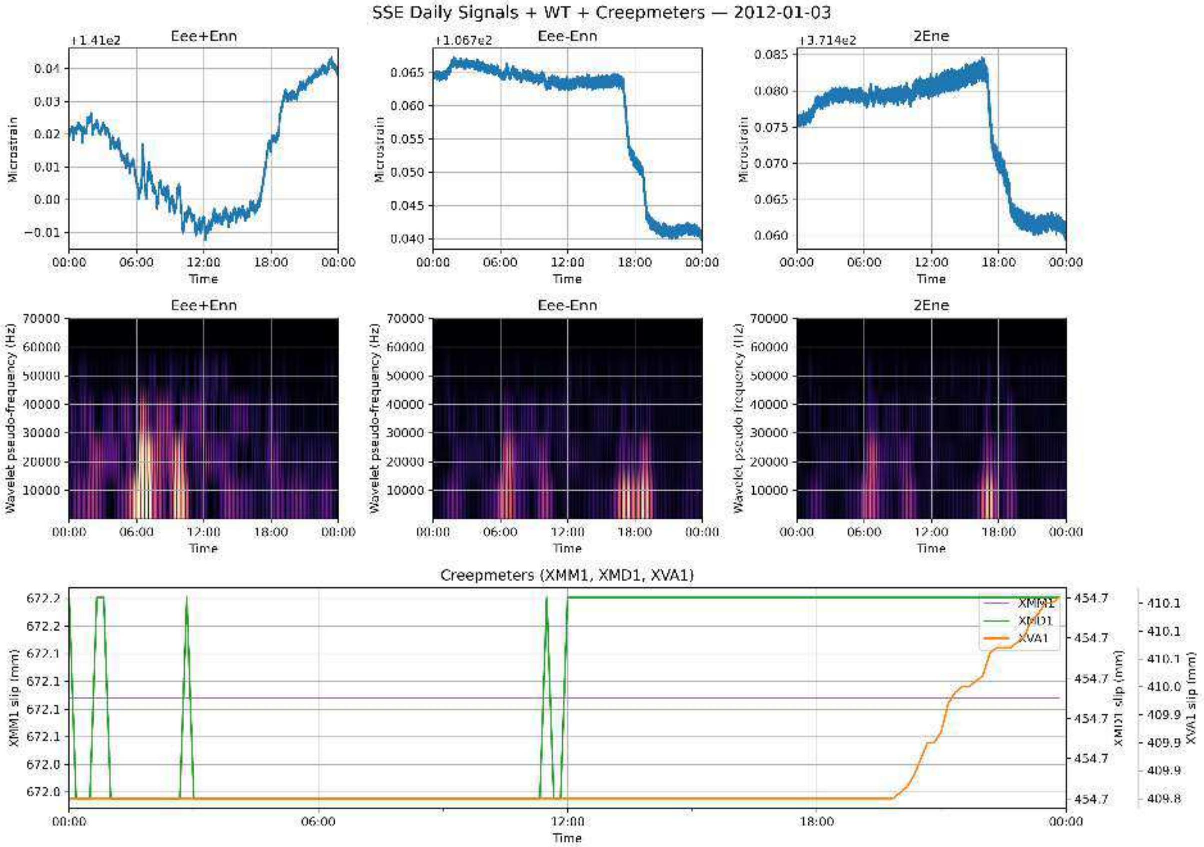
SSE Daily Signals + WT + Creepmeters — 2012-01-03
Eee+Enn
Eee-Enn
2Ene
Microstrain
Time
Wavelet pseudo-frequency (Hz)
Creepmeters (XMM1, XMD1, XVA1)
XMM1
XMD1
XVA1
XMM1 slip (mm)
XMD1 slip (mm)
XVA1 slip (mm)

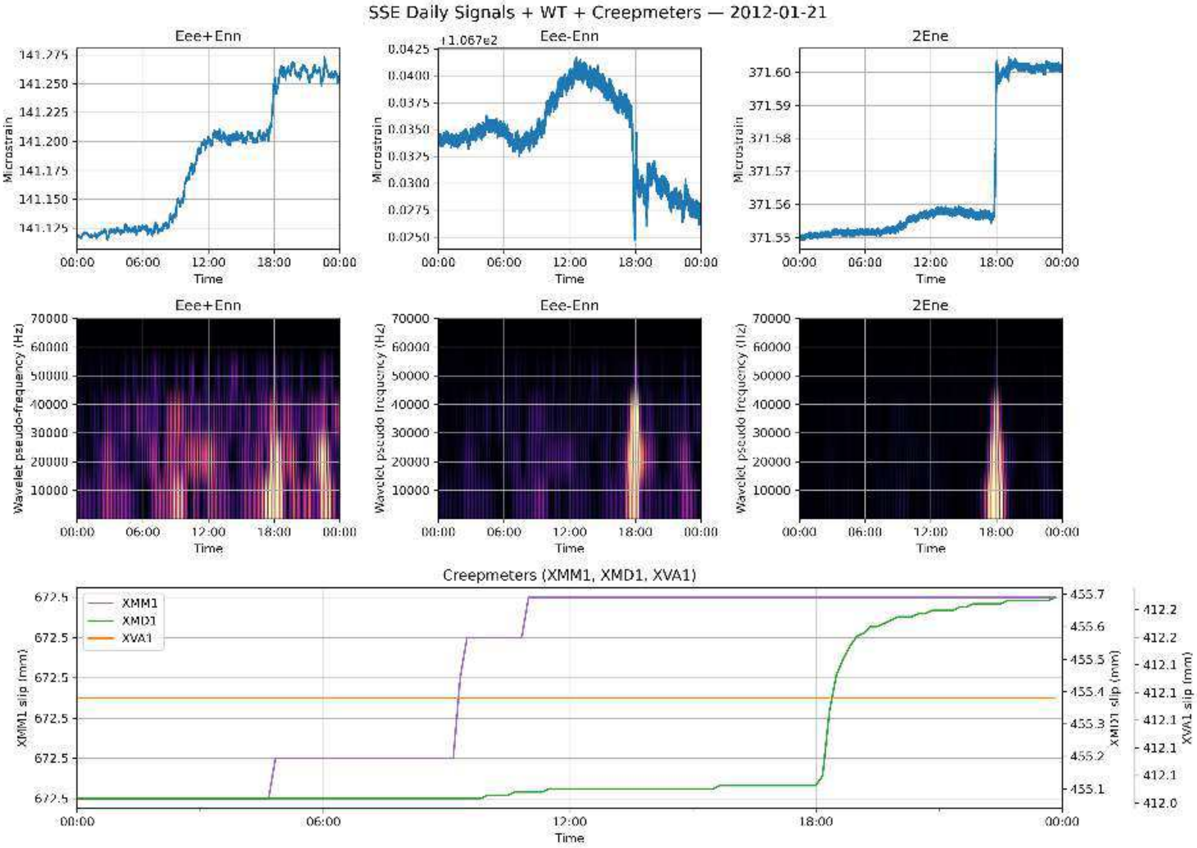
SSE Daily Signals + WT + Creepmeters — 2012-01-21
Eee+Enn
Eee-Enn
2Ene
+1.067e2
Microstrain
Time
Wavelet pseudo-frequency (Hz)
Creepmeters (XMM1, XMD1, XVA1)
XMM1
XMD1
XVA1
XMM1 slip (mm)
XMD1 slip (mm)
XVA1 slip (mm)

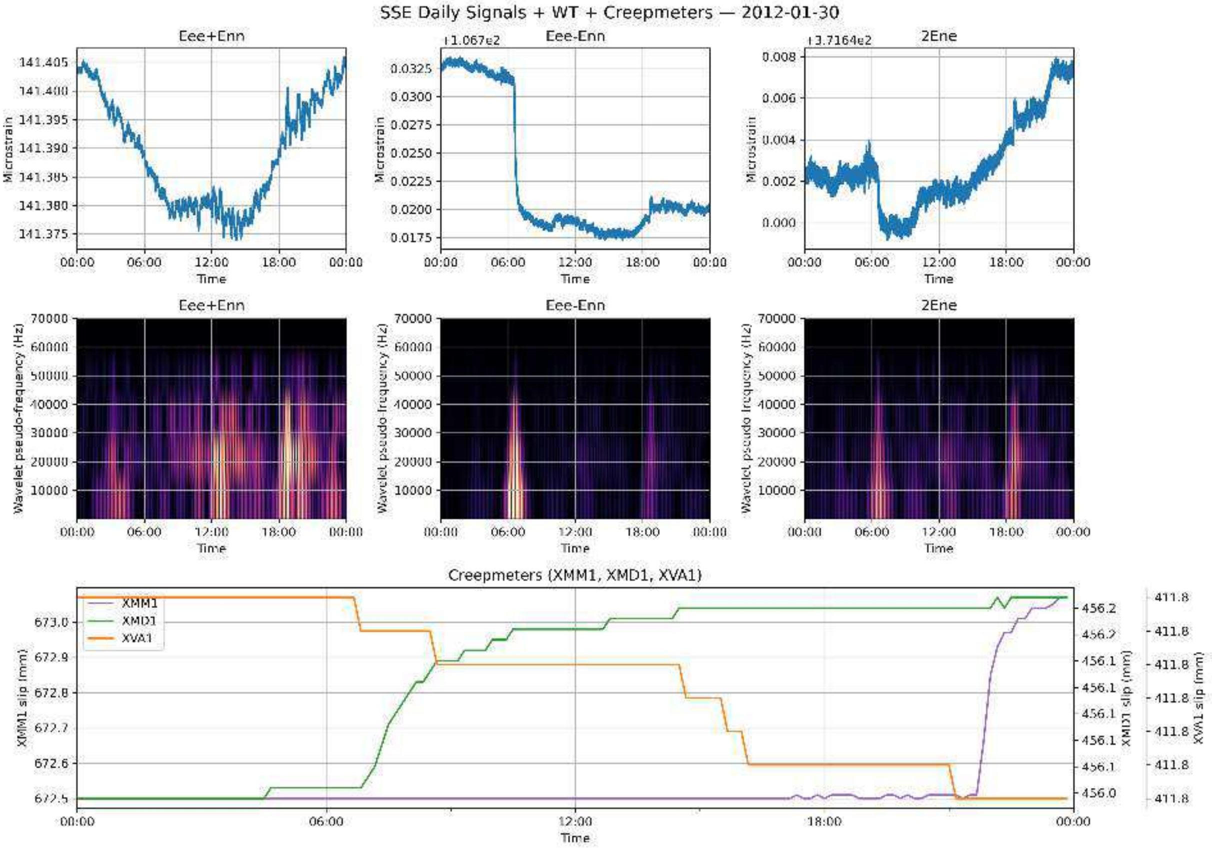
SSE Daily Signals + WT + Creepmeters — 2012-01-30
Eee+Enn
Eee-Enn
2Ene
+1.067e2
+3.7164e2
Microstrain
Time
Wavelet pseudo-frequency (Hz)
Creepmeters (XMM1, XMD1, XVA1)
XMM1
XMD1
XVA1
XMM1 slip (mm)
XMD1 slip (mm)
XVA1 slip (mm)

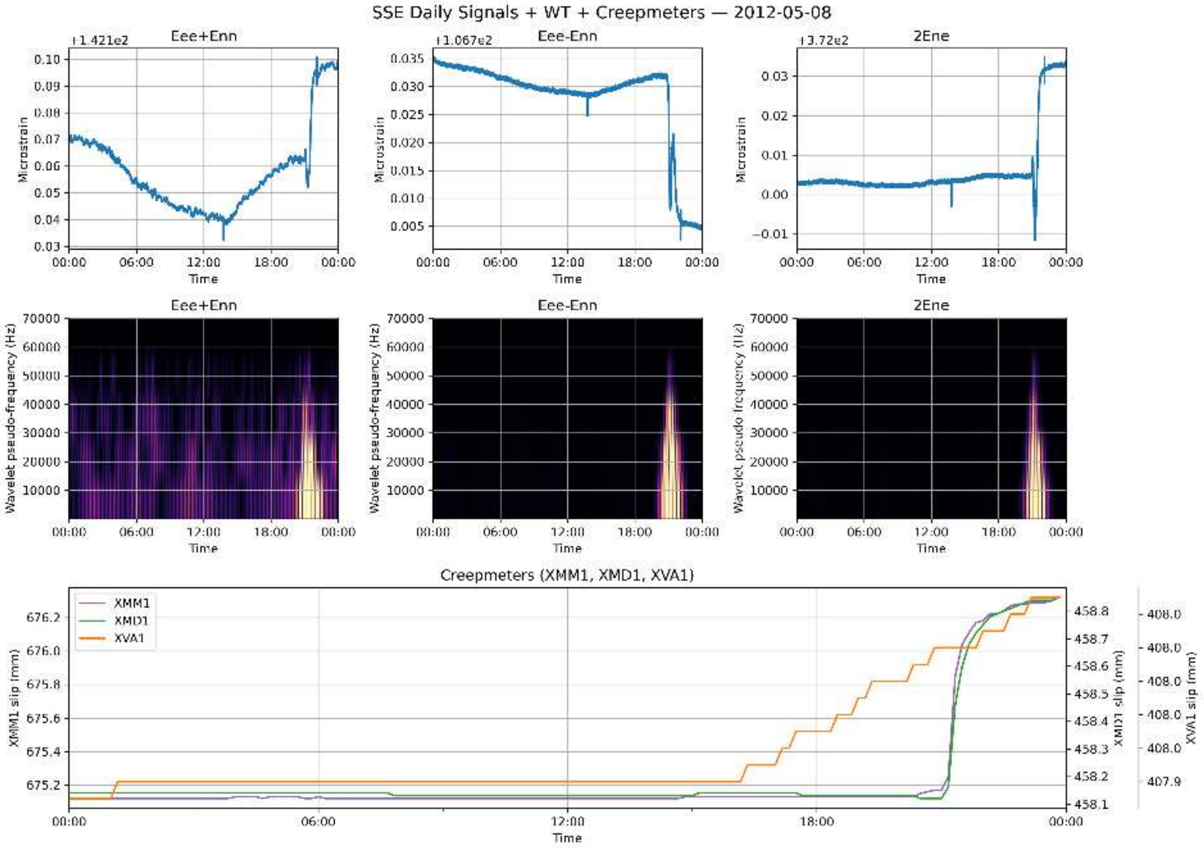
SSE Daily Signals + WT + Creepmeters — 2012-05-08
Eee+Enn
Eee-Enn
2Ene
Microstrain
Time
Wavelet pseudo-frequency (Hz)
Creepmeters (XMM1, XMD1, XVA1)
XMM1
XMD1
XVA1
XMM1 slip (mm)
XMD1 slip (mm)
XVA1 slip (mm)

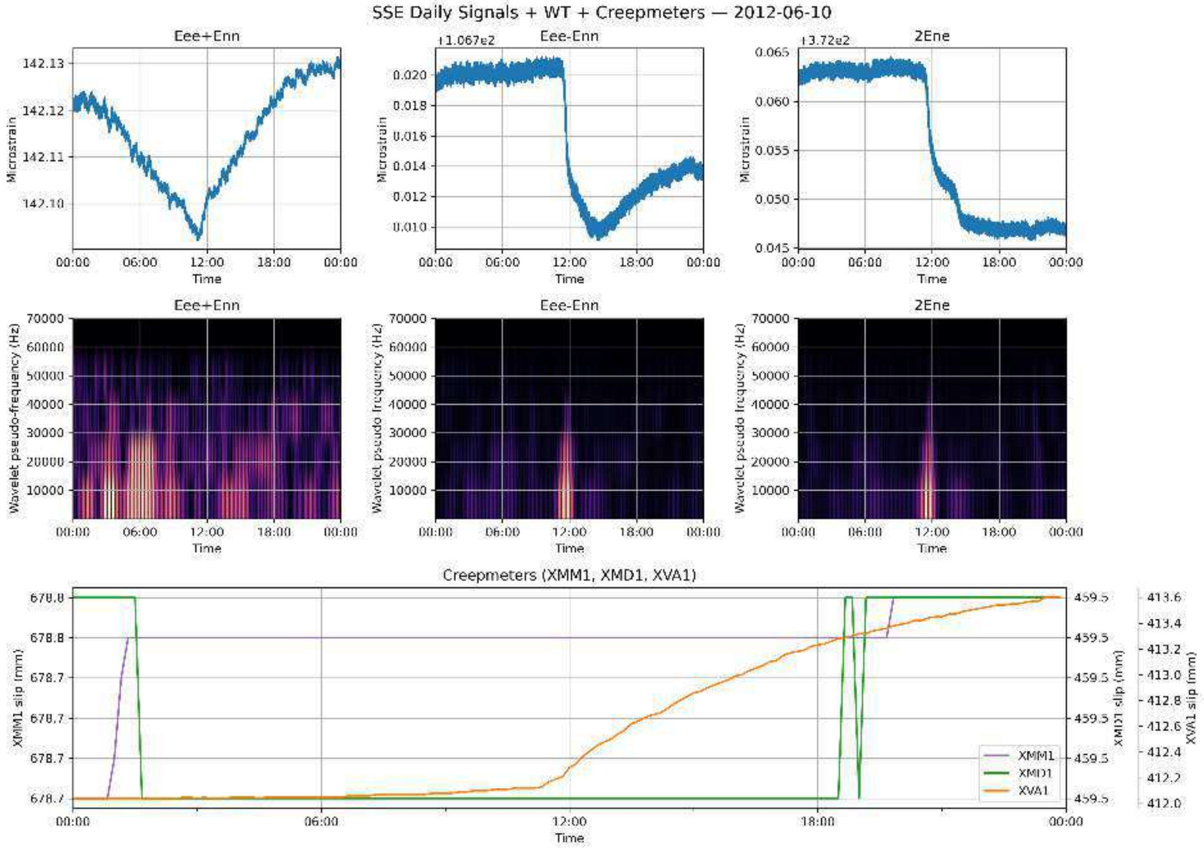
SSE Daily Signals + WT + Creepmeters — 2012-06-10
Eee+Enn
Eee-Enn
2Ene
Microstrain
Time
Wavelet pseudo-frequency (Hz)
Creepmeters (XMM1, XMD1, XVA1)
XMM1
XMD1
XVA1
XMM1 slip (mm)
XMD1 slip (mm)
XVA1 slip (mm)

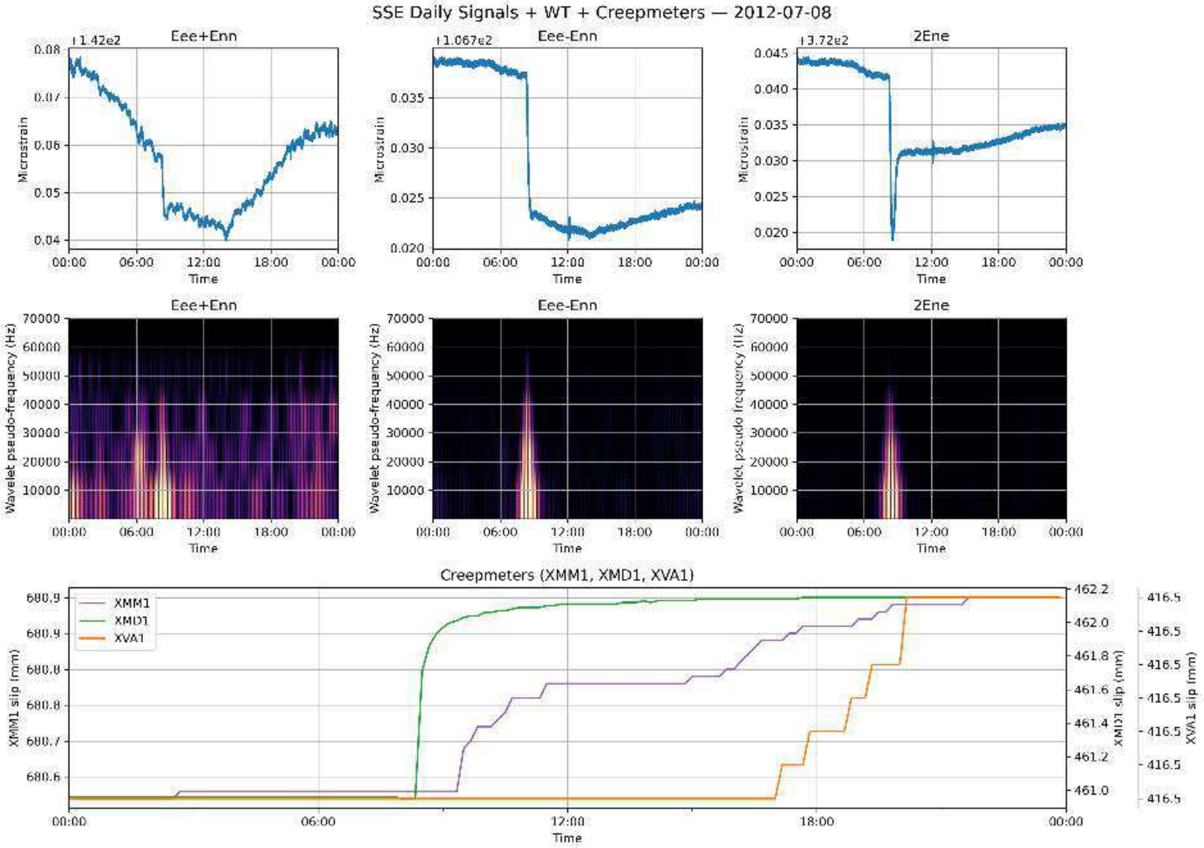
SSE Daily Signals + WT + Creepmeters — 2012-07-08
Eee+Enn
Eee-Enn
2Ene
Microstrain
Time
Wavelet pseudo-frequency (Hz)
Creepmeters (XMM1, XMD1, XVA1)
XMM1
XMD1
XVA1
XMM1 slip (mm)
XMD1 slip (mm)
XVA1 slip (mm)

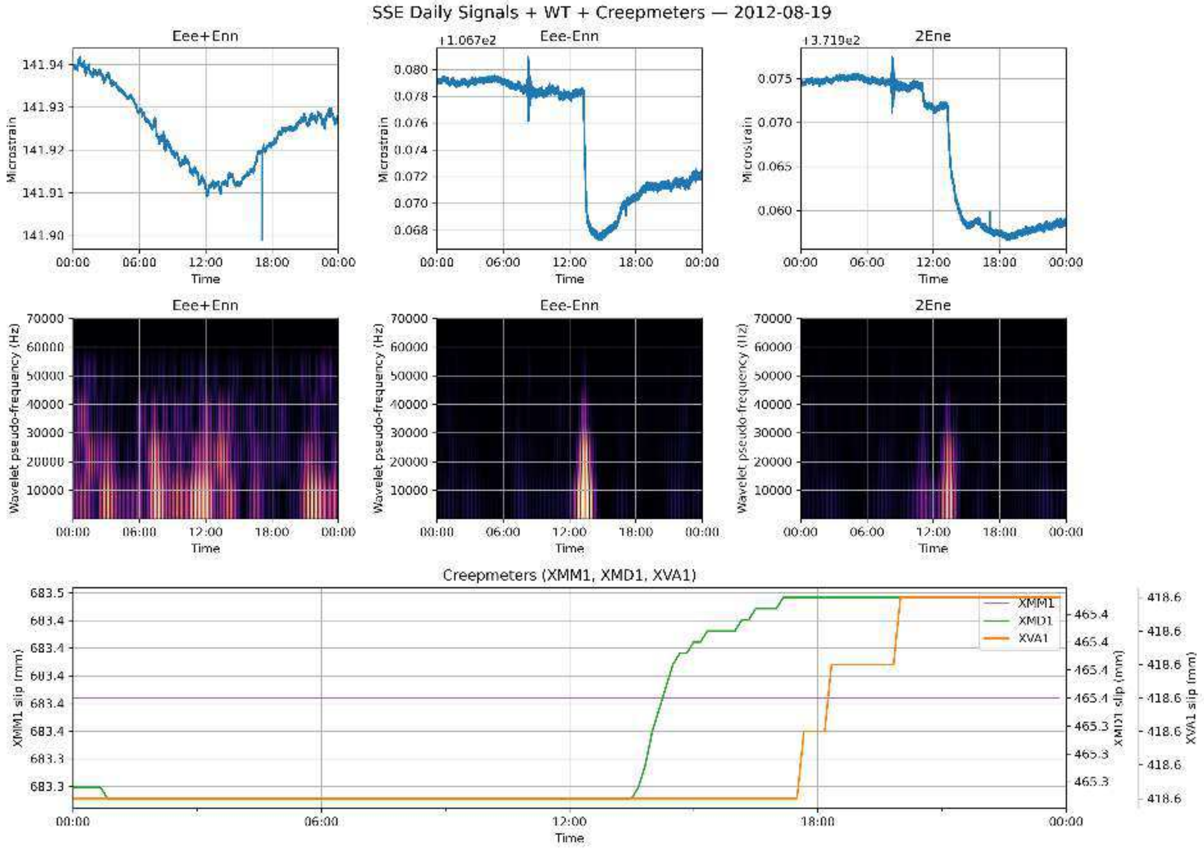
SSE Daily Signals + WT + Creepmeters — 2012-08-19
Eee+Enn
Eee-Enn
2Ene
Microstrain
Time
Wavelet pseudo-frequency (Hz)
Creepmeters (XMM1, XMD1, XVA1)
XMM1
XMD1
XVA1
XMM1 slip (mm)
XMD1 slip (mm)
XVA1 slip (mm)

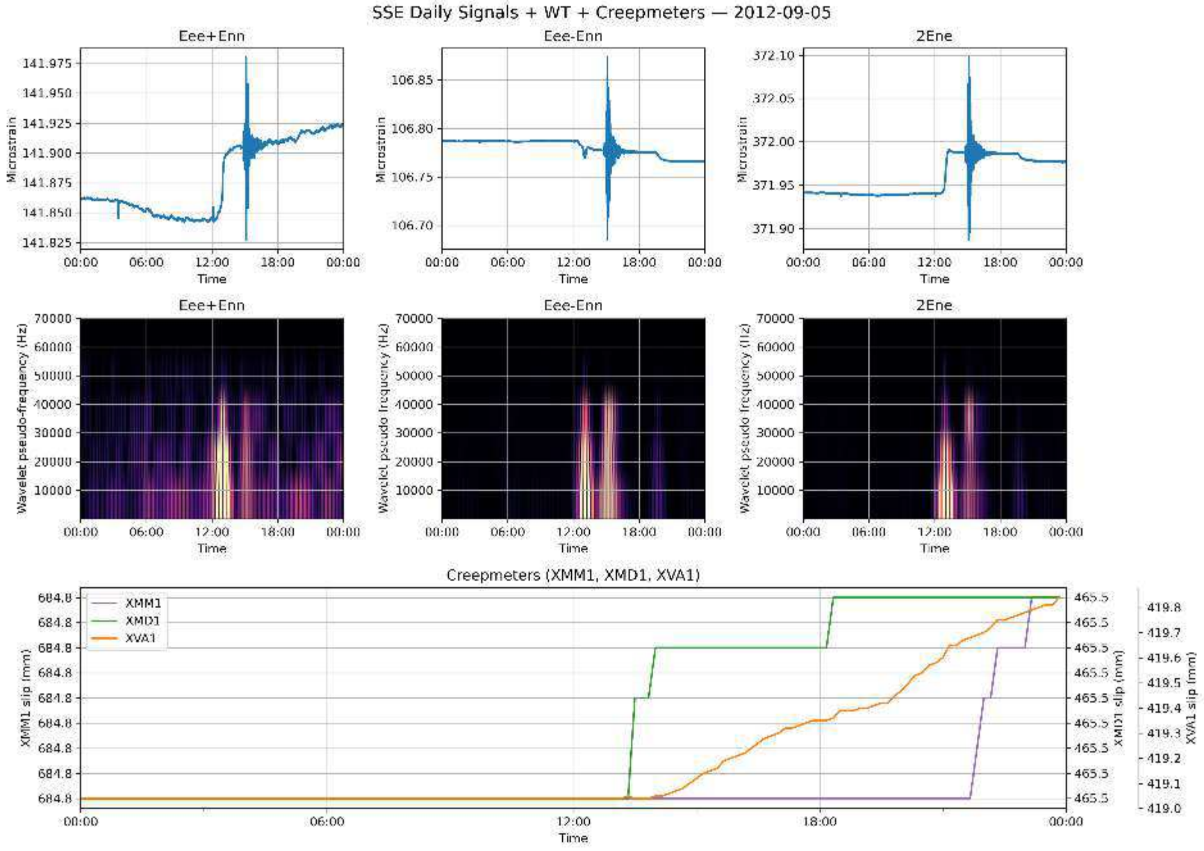
SSE Daily Signals + WT + Creepmeters — 2012-09-05
Eee+Enn
Eee-Enn
2Ene
Microstrain
Time
Wavelet pseudo-frequency (Hz)
Creepmeters (XMM1, XMD1, XVA1)
XMM1
XMD1
XVA1
XMM1 slip (mm)
XMD1 slip (mm)
XVA1 slip (mm)

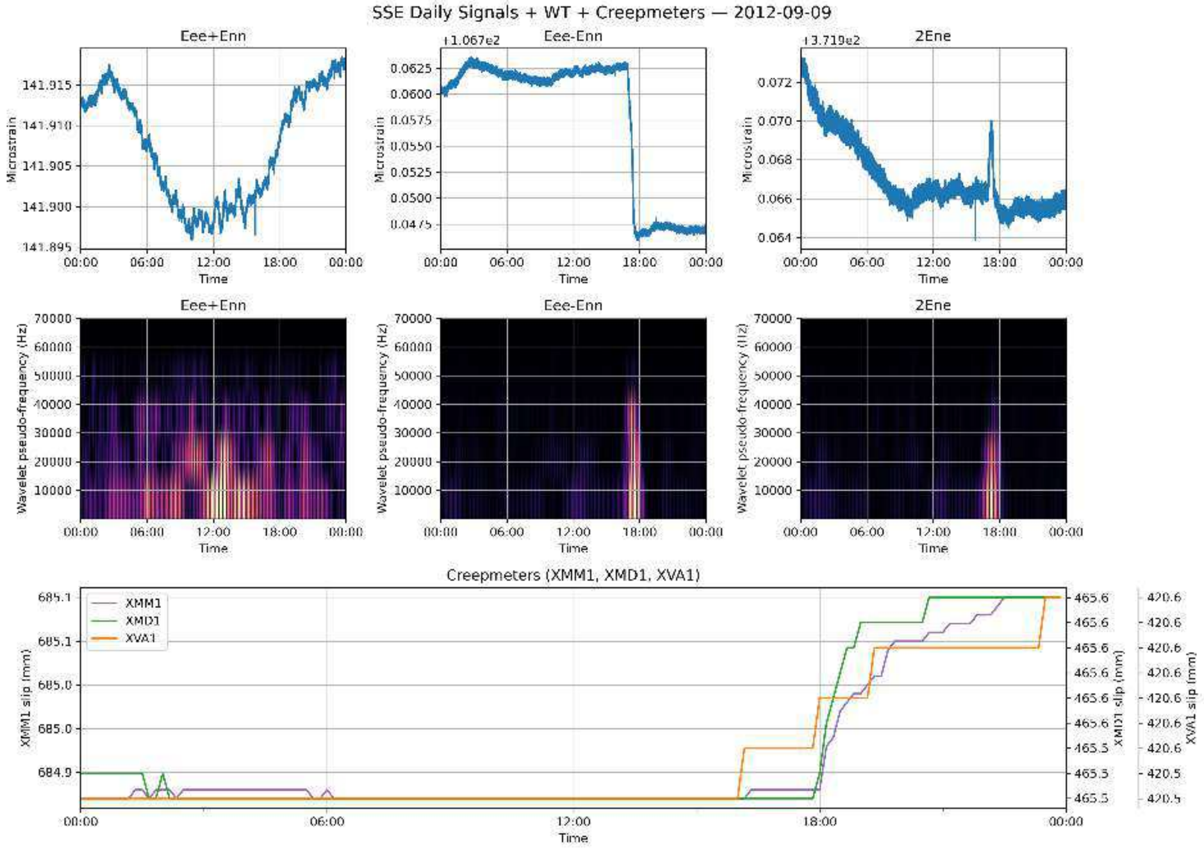
SSE Daily Signals + WT + Creepmeters — 2012-09-09
Eee+Enn
Eee-Enn
2Ene
+1.067e2
+3.719e2
Microstrain
Time
Wavelet pseudo-frequency (Hz)
Creepmeters (XMM1, XMD1, XVA1)
XMM1
XMD1
XVA1
XMM1 slip (mm)
XMD1 slip (mm)
XVA1 slip (mm)

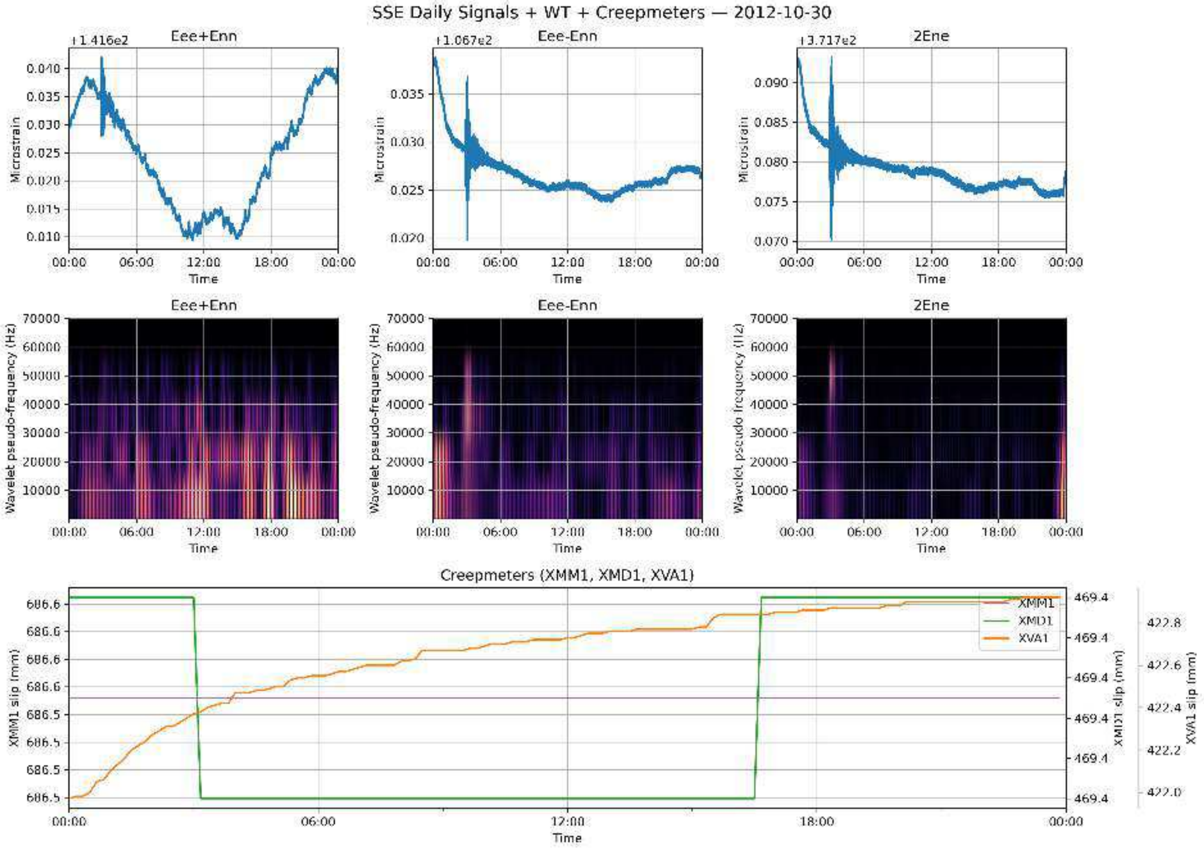

SSE Daily Signals + WT + Creepmeters — 2012-10-30
+1.416e2
Eee+Enn
+1.067e2
Eee-Enn
+3.717e2
2Ene
Microstrain
Time
Wavelet pseudo-frequency (Hz)
Creepmeters (XMM1, XMD1, XVA1)
XMM1
XMD1
XVA1
XMM1 slip (mm)
XMD1 slip (mm)
XVA1 slip (mm)

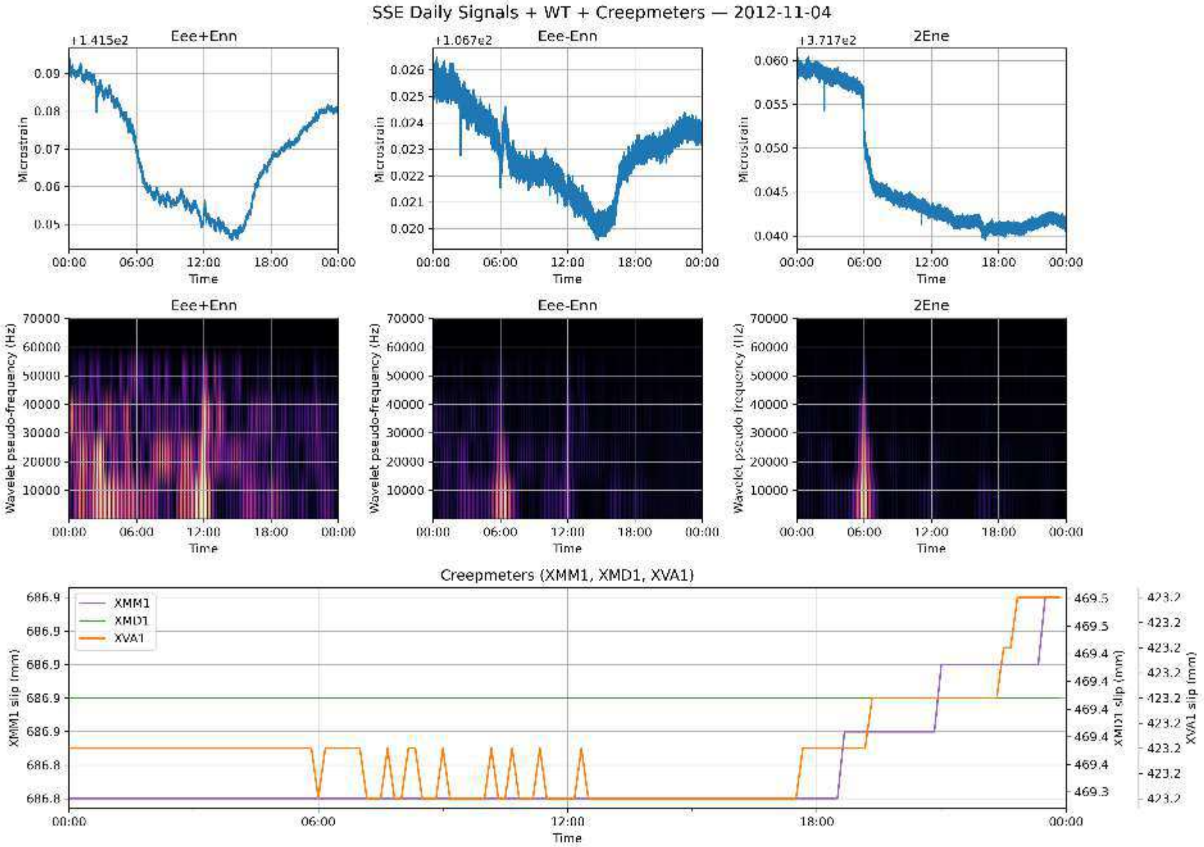

SSE Daily Signals + WT + Creepmeters — 2012-11-04
+1.415e2
Eee+Enn
+1.067e2
Eee-Enn
+3.717e2
2Ene
Microstrain
Time
Wavelet pseudo-frequency (Hz)
Creepmeters (XMM1, XMD1, XVA1)
XMM1
XMD1
XVA1
XMM1 slip (mm)
XMD1 slip (mm)
XVA1 slip (mm)

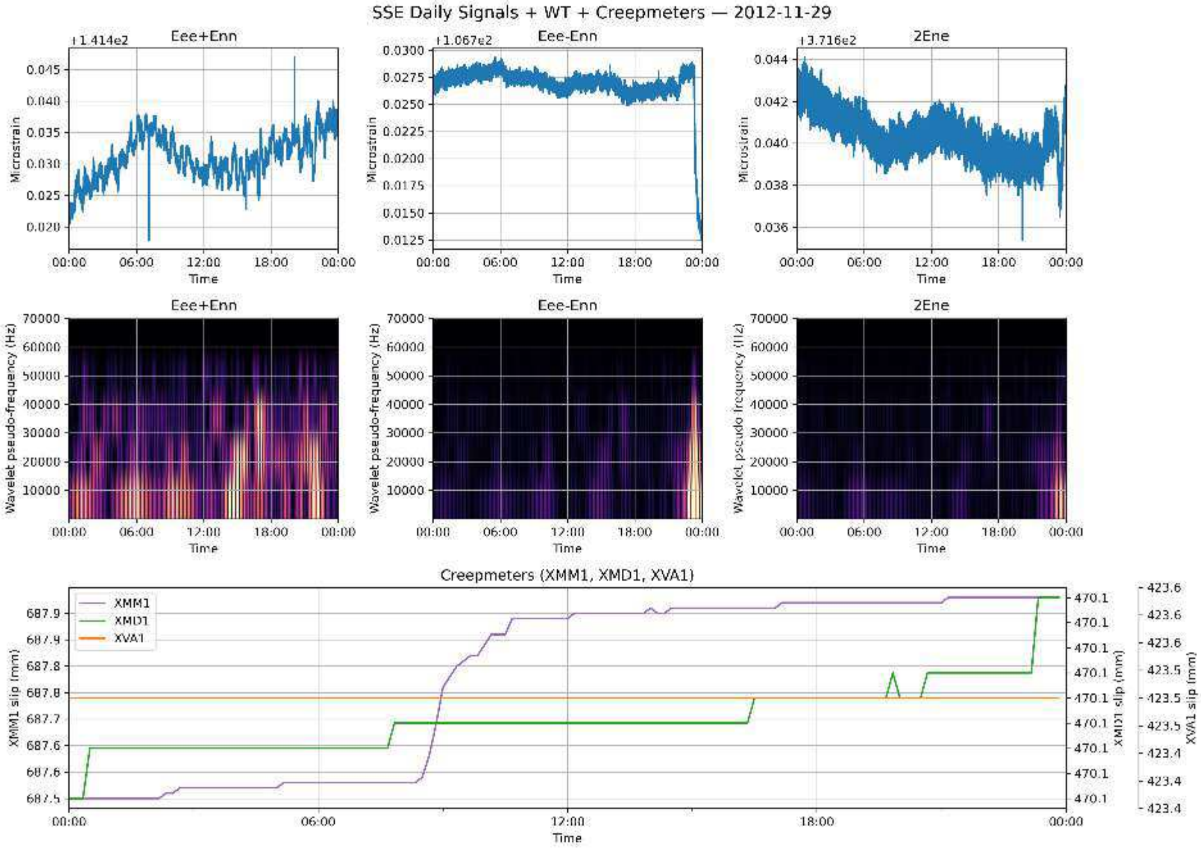
SSE Daily Signals + WT + Creepmeters — 2012-11-29
Eee+Enn
Eee-Enn
2Ene
+1.414e2
+1.067e2
+3.716e2
Microstrain
Time
Wavelet pseudo-frequency (Hz)
Creepmeters (XMM1, XMD1, XVA1)
XMM1
XMD1
XVA1
XMM1 slip (mm)
XMD1 slip (mm)
XVA1 slip (mm)

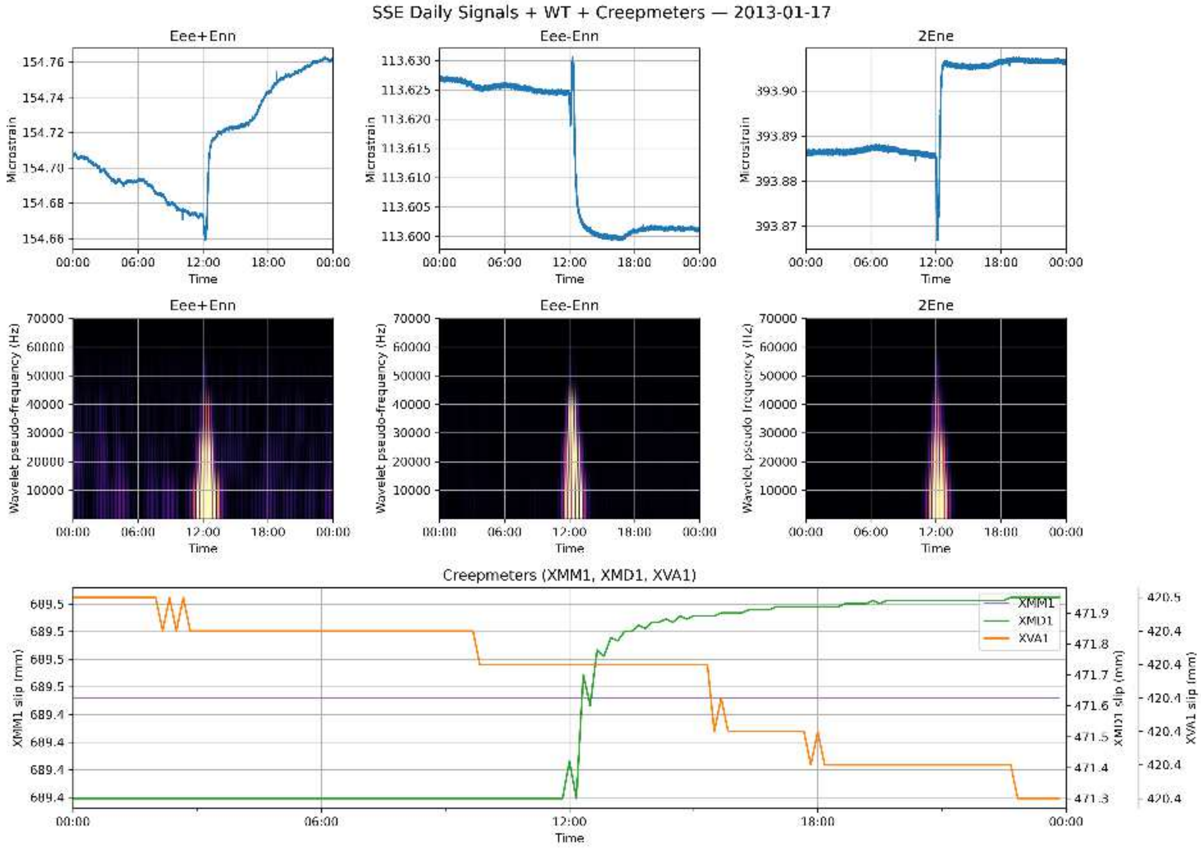
SSE Daily Signals + WT + Creepmeters — 2013-01-17
Eee+Enn
Eee-Enn
2Ene
Microstrain
Time
Wavelet pseudo-frequency (Hz)
Creepmeters (XMM1, XMD1, XVA1)
XMM1
XMD1
XVA1
XMM1 slip (mm)
XMD1 slip (mm)
XVA1 slip (mm)

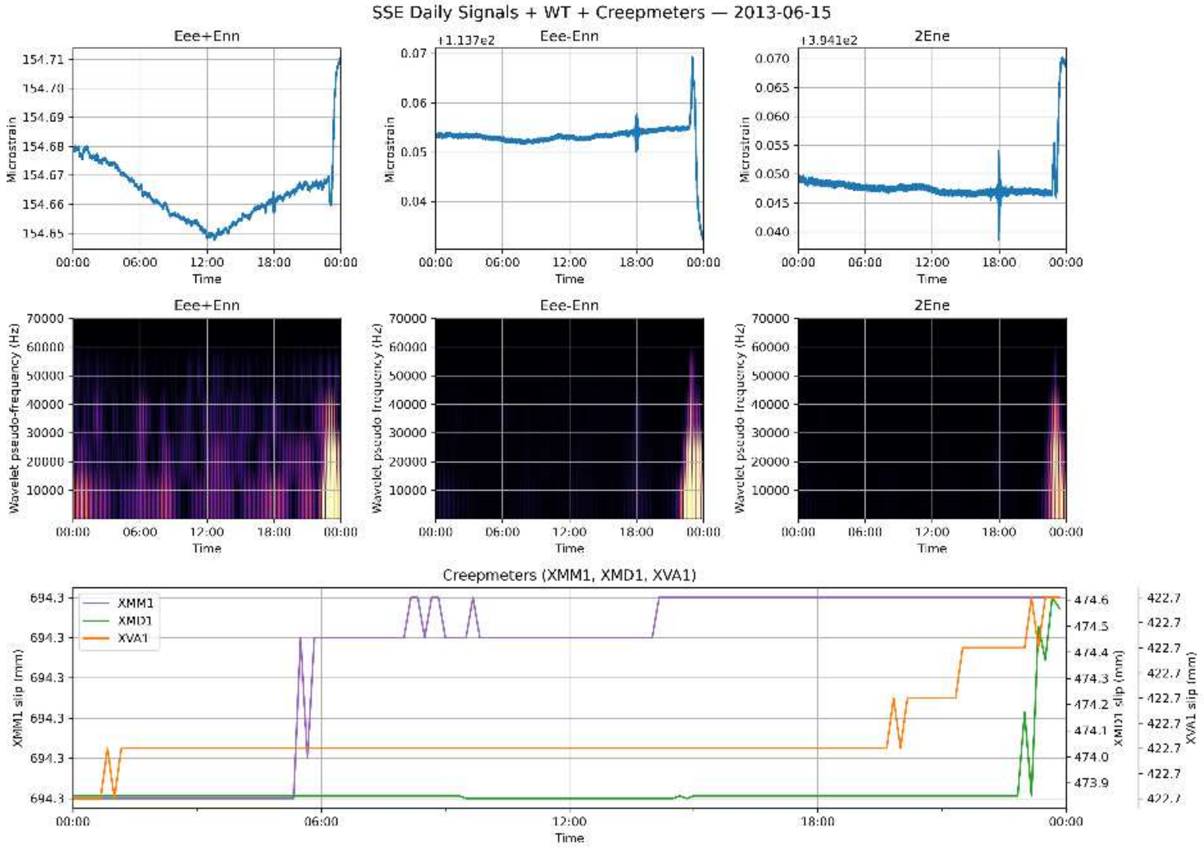
SSE Daily Signals + WT + Creepmeters — 2013-06-15
Eee+Enn
Eee-Enn
2Ene
+1.137e2
+3.941e2
Microstrain
Time
Wavelet pseudo-frequency (Hz)
Creepmeters (XMM1, XMD1, XVA1)
XMM1
XMD1
XVA1
XMM1 slip (mm)
XMD1 slip (mm)
XVA1 slip (mm)

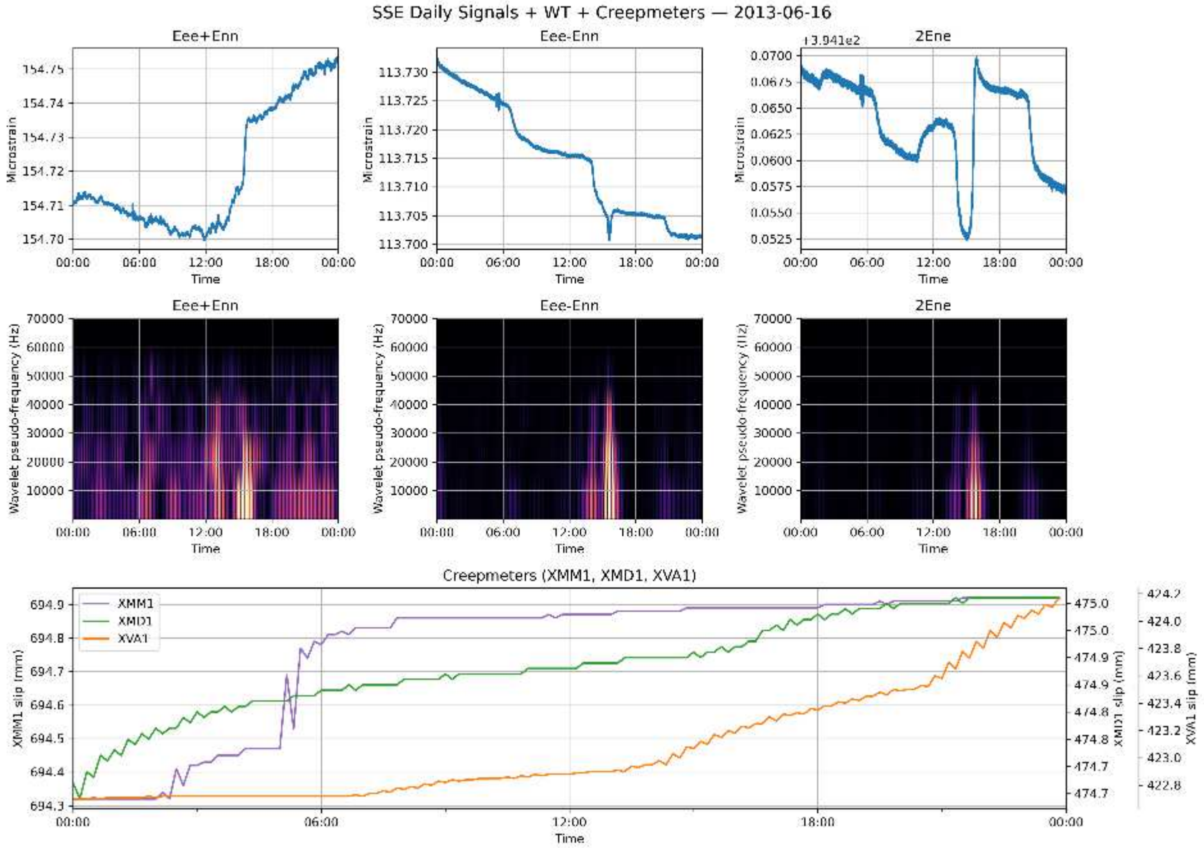
SSE Daily Signals + WT + Creepmeters — 2013-06-16
Eee+Enn
Eee-Enn
2Ene
+3.941e2
Microstrain
Time
Wavelet pseudo-frequency (Hz)
Creepmeters (XMM1, XMD1, XVA1)
XMM1
XMD1
XVA1
XMM1 slip (mm)
XMD1 slip (mm)
XVA1 slip (mm)

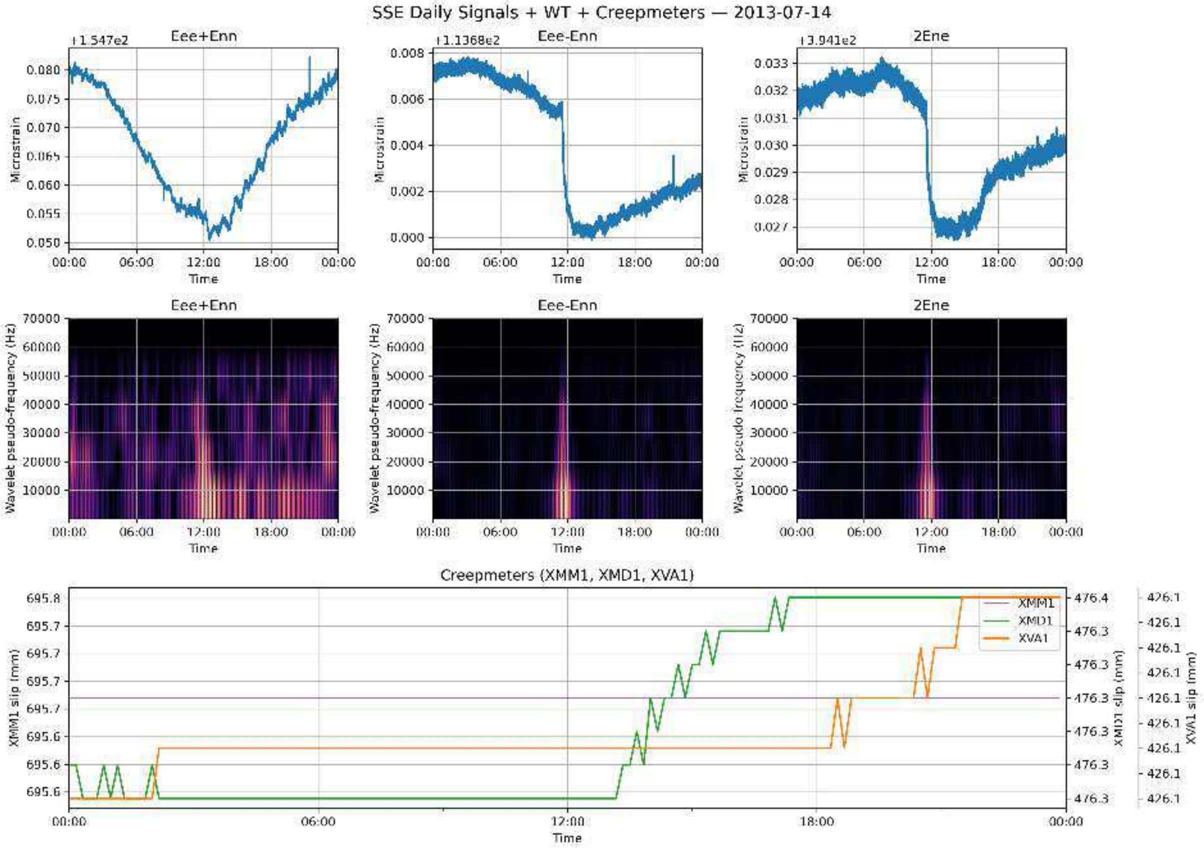
SSE Daily Signals + WT + Creepmeters — 2013-07-14
Eee+Enn
Eee-Enn
2Ene
+1.547e2
+1.1368e2
+3.941e2
Microstrain
Wavelet pseudo-frequency (Hz)
Time
Creepmeters (XMM1, XMD1, XVA1)
XMM1
XMD1
XVA1
XMM1 slip (mm)
XMD1 slip (mm)
XVA1 slip (mm)

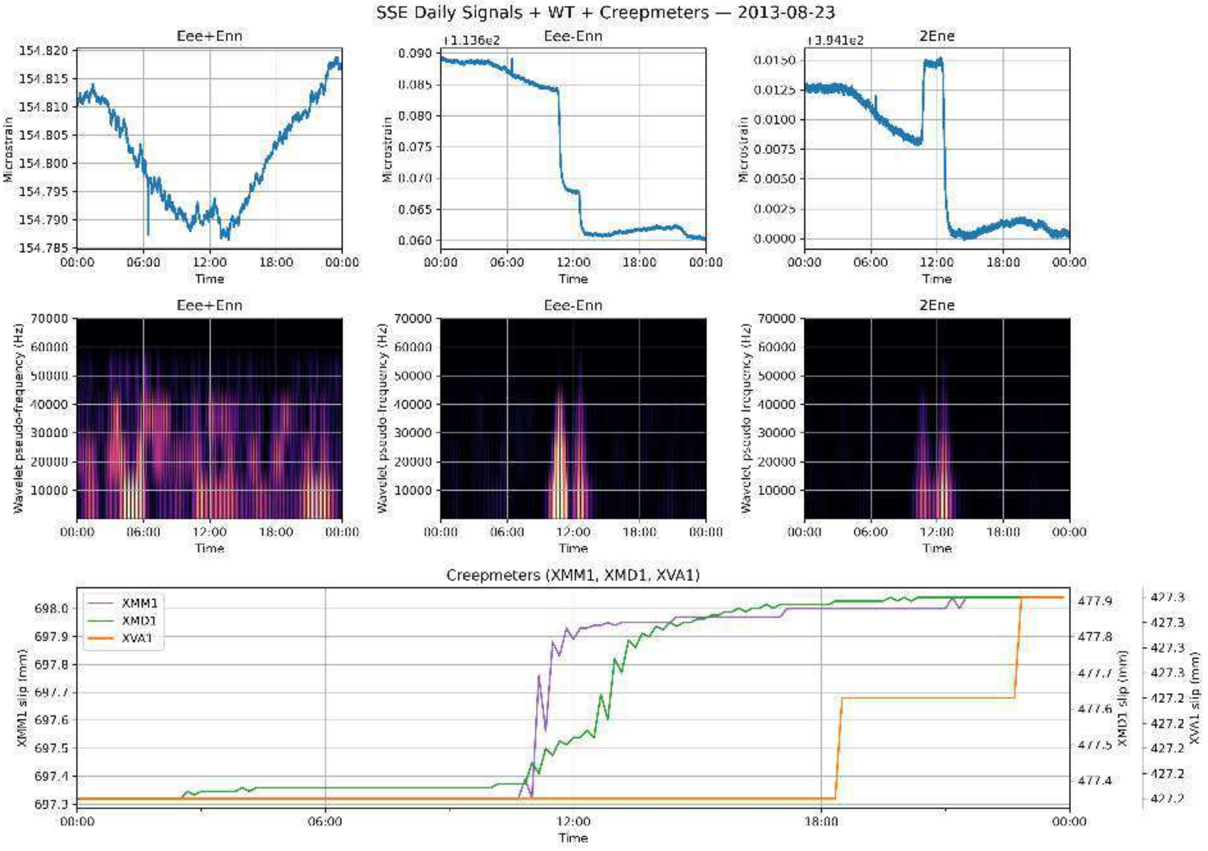
SSE Daily Signals + WT + Creepmeters — 2013-08-23
Eee+Enn
Eee-Enn
2Ene
+1.136e2
+3.941e2
Microstrain
Wavelet pseudo-frequency (Hz)
Time
Creepmeters (XMM1, XMD1, XVA1)
XMM1
XMD1
XVA1
XMM1 slip (mm)
XMD1 slip (mm)
XVA1 slip (mm)

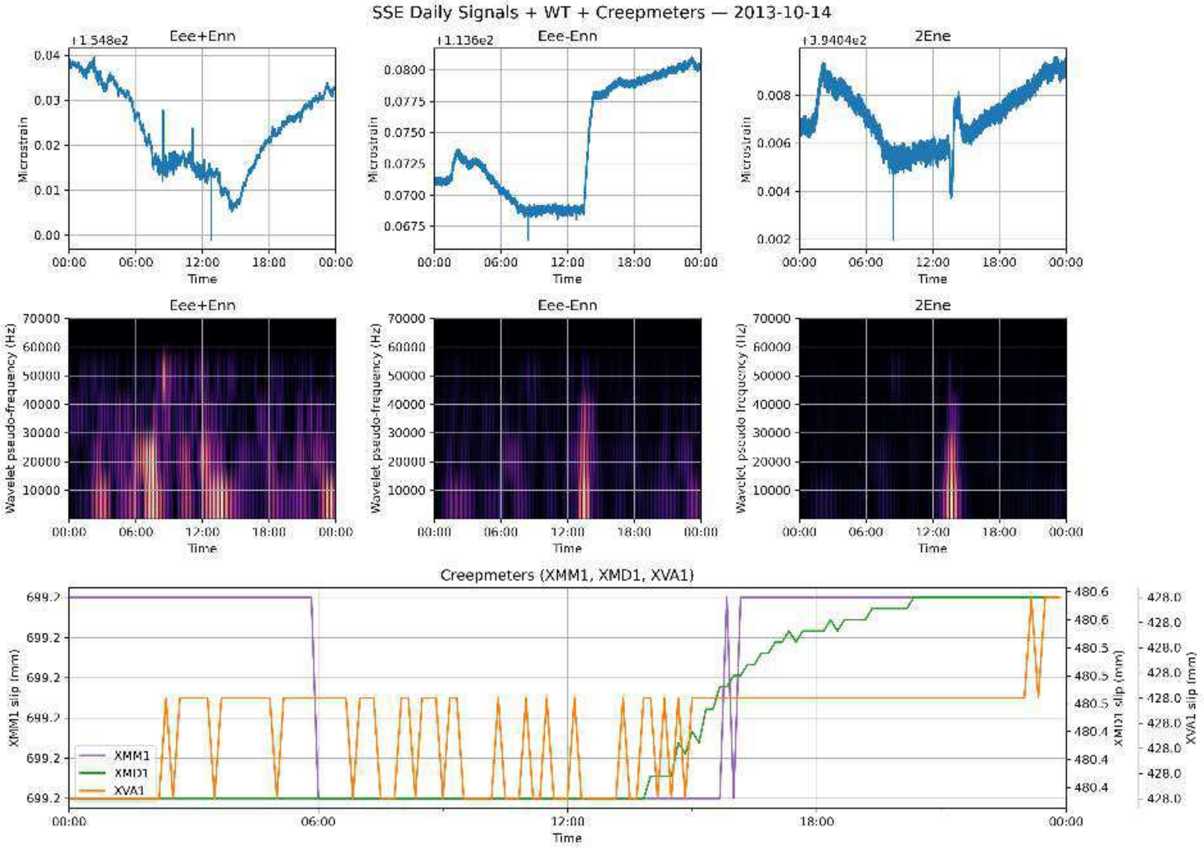
SSE Daily Signals + WT + Creepmeters — 2013-10-14
Eee+Enn
Eee-Enn
2Ene
Microstrain
Time
Wavelet pseudo-frequency (Hz)
Creepmeters (XMM1, XMD1, XVA1)
XMM1 slip (mm)
XMD1 slip (mm)
XVA1 slip (mm)
XMM1
XMD1
XVA1

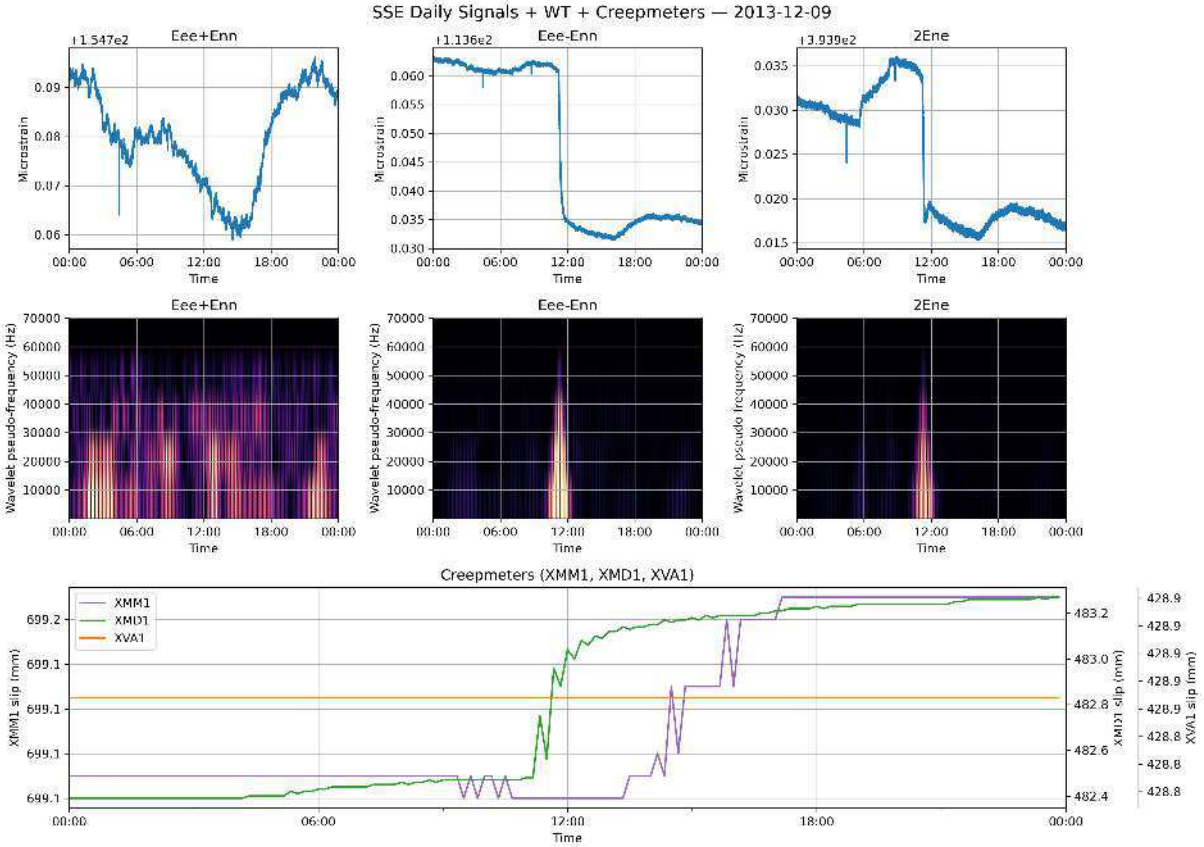
SSE Daily Signals + WT + Creepmeters — 2013-12-09
Eee+Enn
Eee-Enn
2Ene
Microstrain
Time
Wavelet pseudo-frequency (Hz)
Creepmeters (XMM1, XMD1, XVA1)
XMM1 slip (mm)
XMD1 slip (mm)
XVA1 slip (mm)
XMM1
XMD1
XVA1

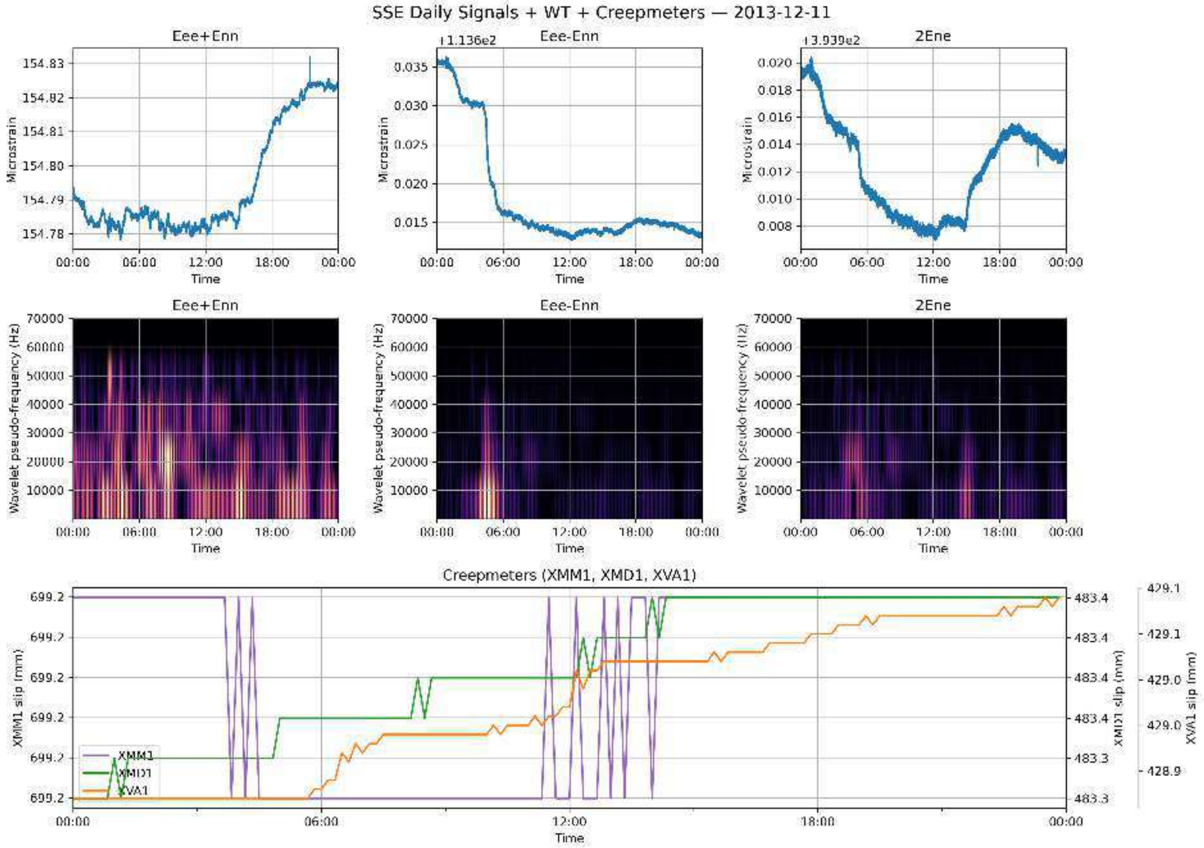
SSE Daily Signals + WT + Creepmeters — 2013-12-11
Eee+Enn
Eee-Enn
2Ene
Microstrain
Time
Wavelet pseudo-frequency (Hz)
Creepmeters (XMM1, XMD1, XVA1)
XMM1
XMD1
XVA1
XMM1 slip (mm)
XMD1 slip (mm)
XVA1 slip (mm)

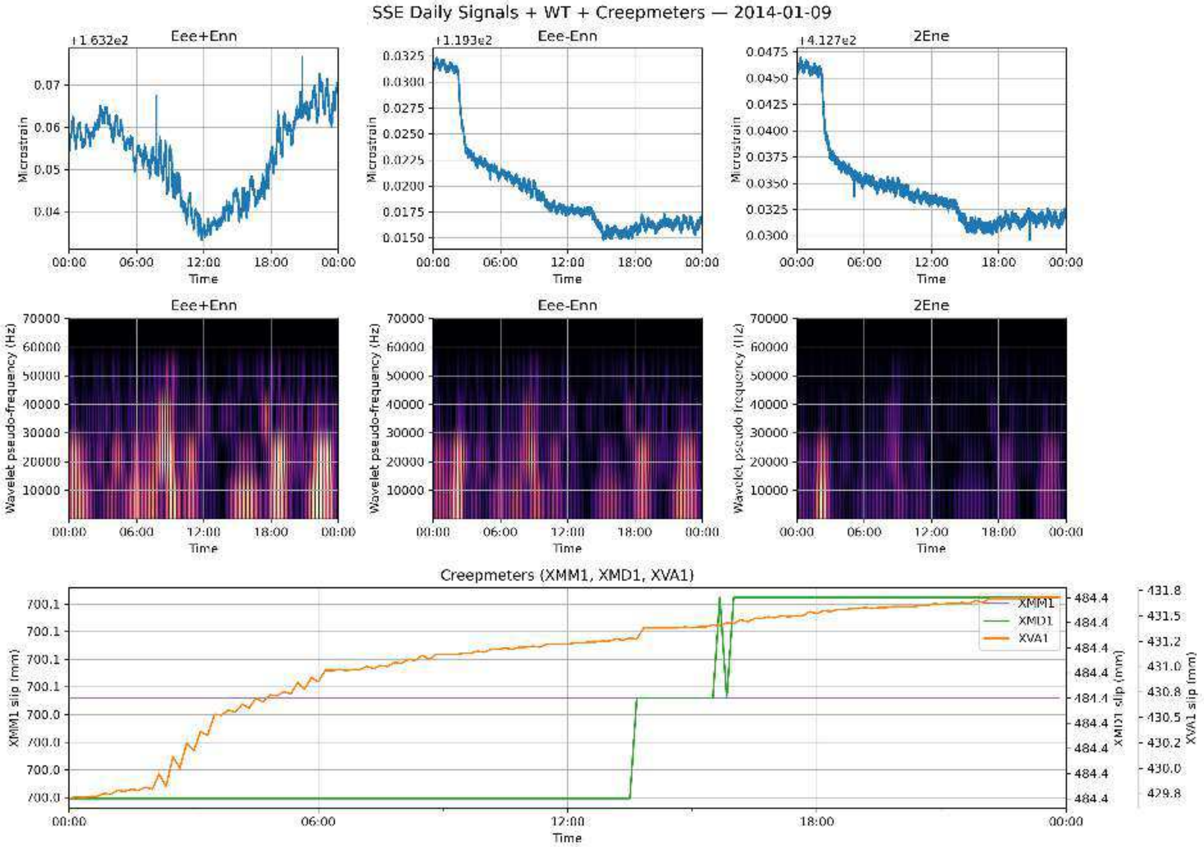
SSE Daily Signals + WT + Creepmeters — 2014-01-09
Eee+Enn
Eee-Enn
2Ene
Microstrain
Time
Wavelet pseudo-frequency (Hz)
Creepmeters (XMM1, XMD1, XVA1)
XMM1
XMD1
XVA1
XMM1 slip (mm)
XMD1 slip (mm)
XVA1 slip (mm)

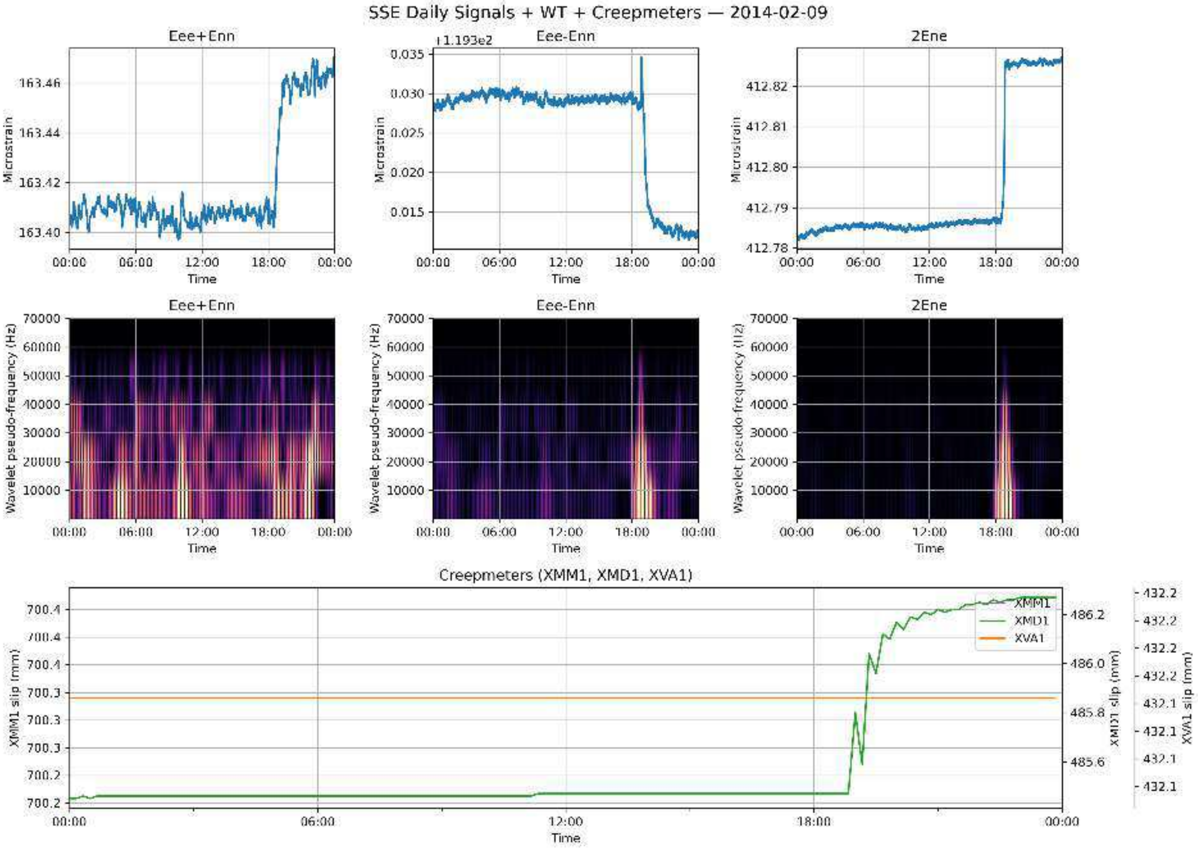
SSE Daily Signals + WT + Creepmeters — 2014-02-09
Eee+Enn
Eee-Enn
2Ene
Microstrain
Time
Wavelet pseudo-frequency (Hz)
Creepmeters (XMM1, XMD1, XVA1)
XMM1
XMD1
XVA1
XMM1 slip (mm)
XMD1 slip (mm)
XVA1 slip (mm)

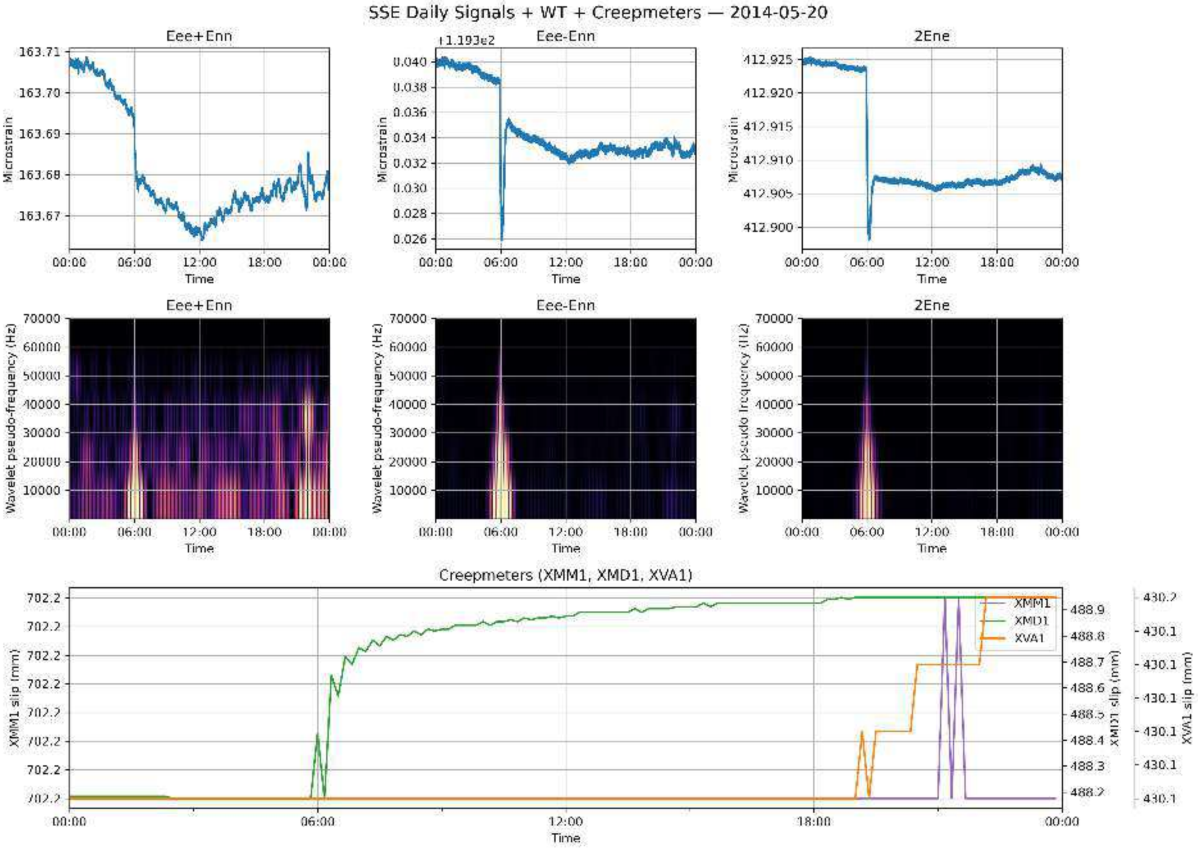
SSE Daily Signals + WT + Creepmeters — 2014-05-20
Eee+Enn
Eee-Enn
2Ene
Microstrain
Time
Wavelet pseudo-frequency (Hz)
Creepmeters (XMM1, XMD1, XVA1)
XMM1
XMD1
XVA1
XMM1 slip (mm)
XMD1 slip (mm)
XVA1 slip (mm)

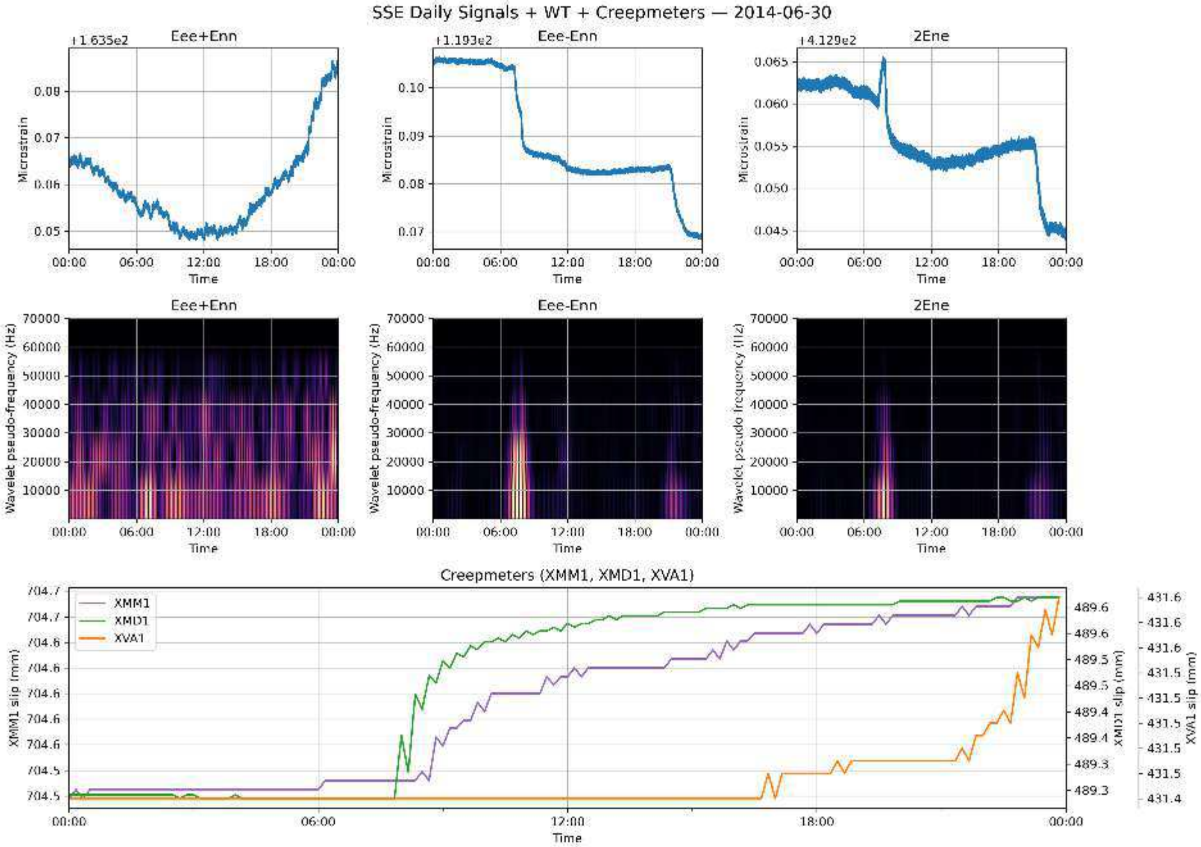
SSE Daily Signals + WT + Creepmeters — 2014-06-30
Eee+Enn
Eee-Enn
2Ene
Microstrain
Time
Wavelet pseudo-frequency (Hz)
Creepmeters (XMM1, XMD1, XVA1)
XMM1
XMD1
XVA1
XMM1 slip (mm)
XMD1 slip (mm)
XVA1 slip (mm)

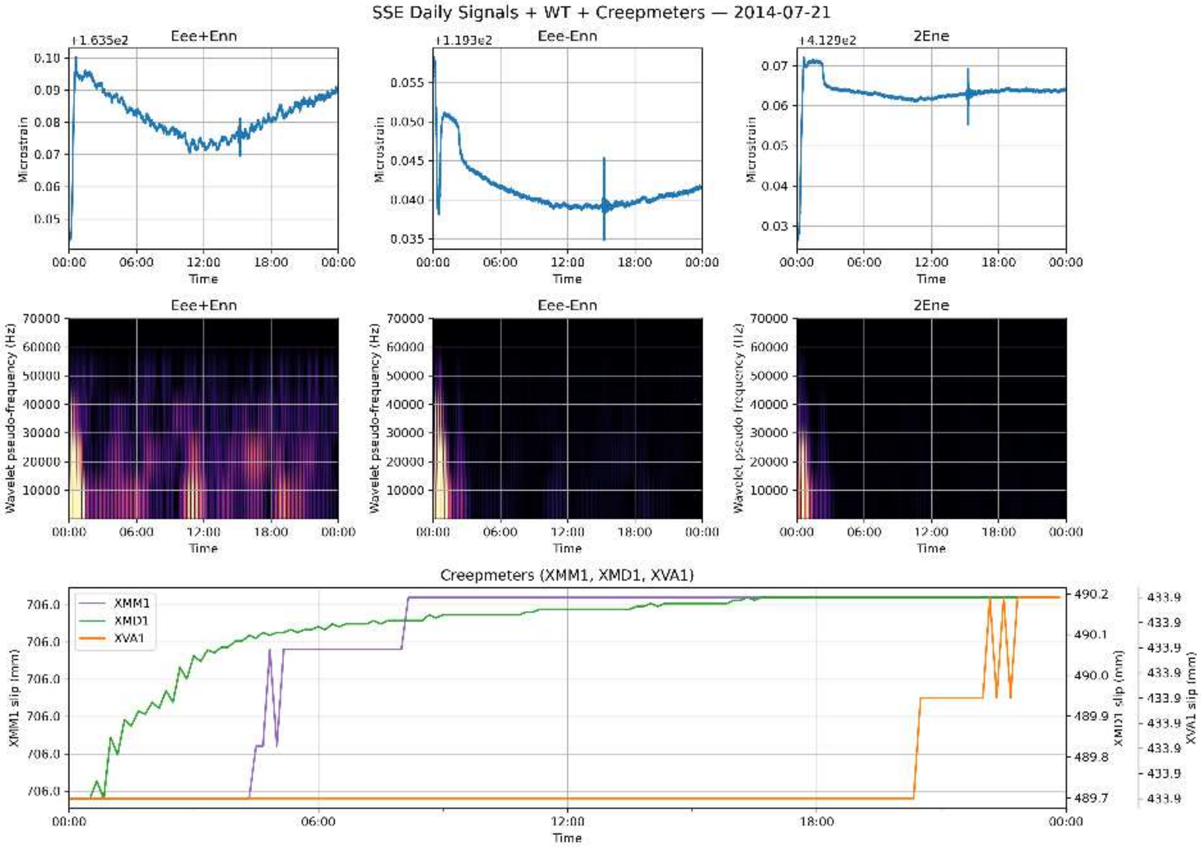
SSE Daily Signals + WT + Creepmeters — 2014-07-21
Eee+Enn
Eee-Enn
2Ene
Microstrain
Time
Wavelet pseudo-frequency (Hz)
Creepmeters (XMM1, XMD1, XVA1)
XMM1
XMD1
XVA1
XMM1 slip (mm)
XMD1 slip (mm)
XVA1 slip (mm)

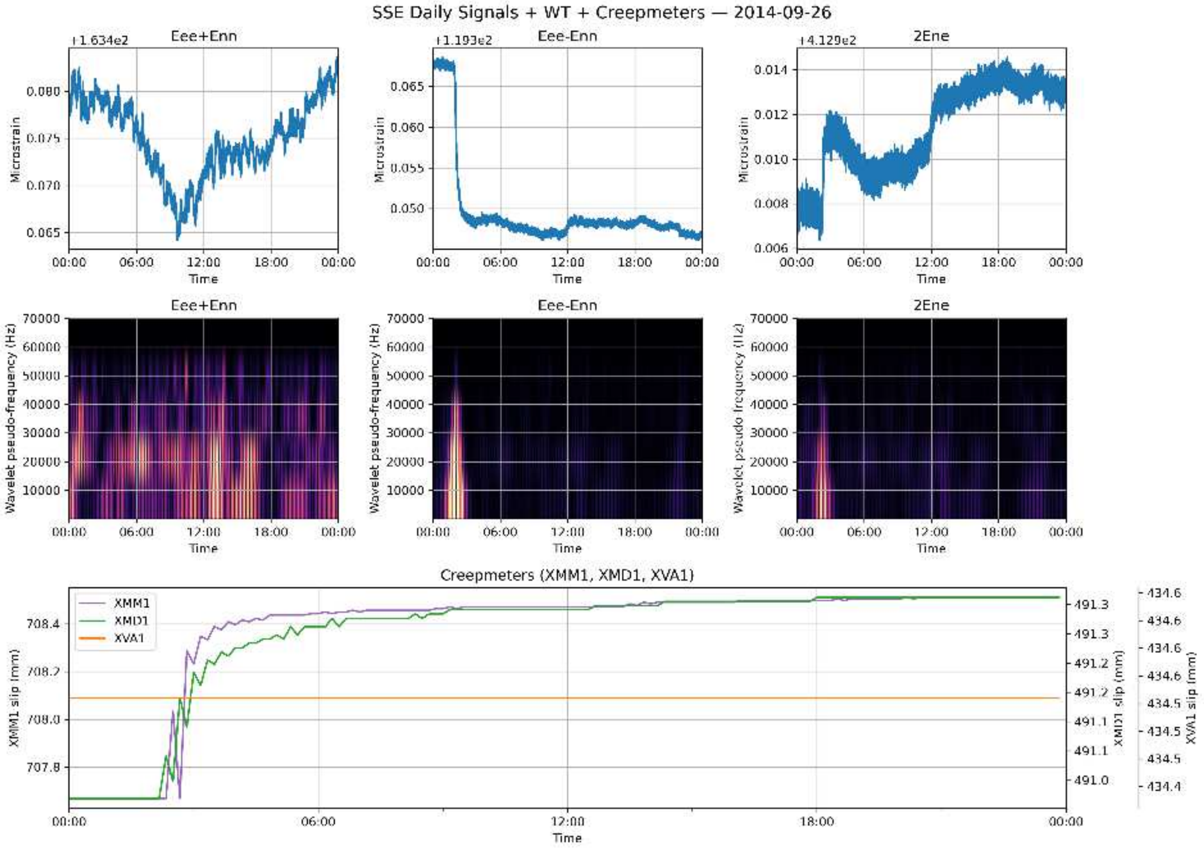
SSE Daily Signals + WT + Creepmeters — 2014-09-26
Eee+Enn
Eee-Enn
2Ene
Microstrain
Time
Wavelet pseudo-frequency (Hz)
Creepmeters (XMM1, XMD1, XVA1)
XMM1
XMD1
XVA1
XMM1 slip (mm)
XMD1 slip (mm)
XVA1 slip (mm)

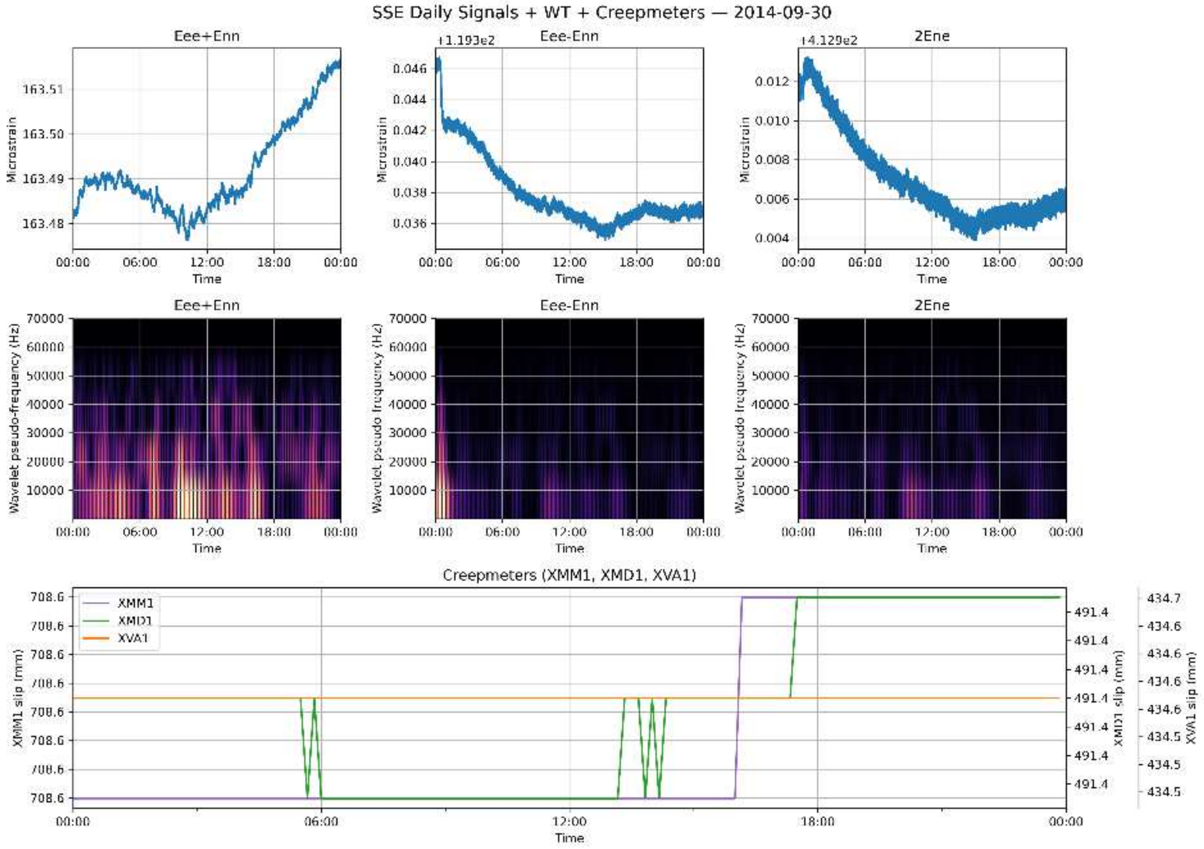
SSE Daily Signals + WT + Creepmeters — 2014-09-30
Eee+Enn
Eee-Enn
2Ene
Microstrain
Time
Wavelet pseudo-frequency (Hz)
Creepmeters (XMM1, XMD1, XVA1)
XMM1
XMD1
XVA1
XMM1 slip (mm)
XMD1 slip (mm)
XVA1 slip (mm)

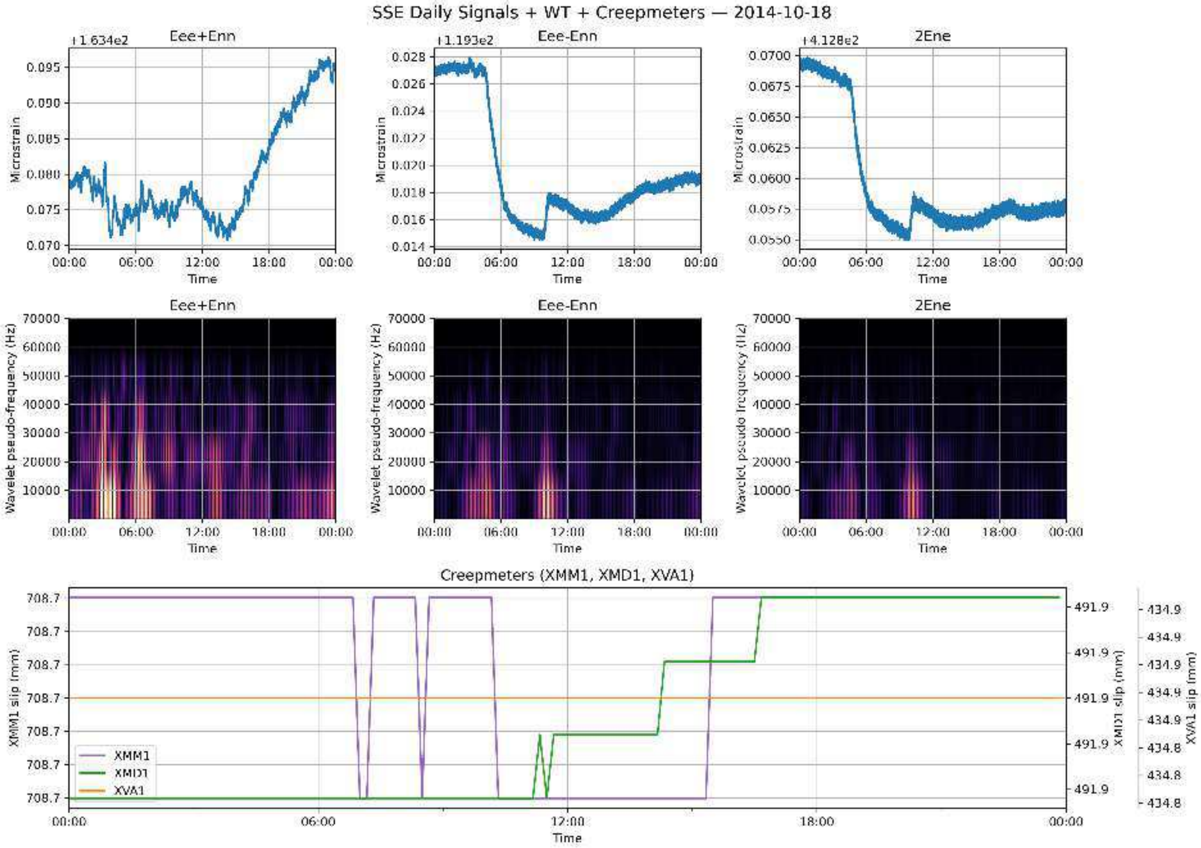
SSE Daily Signals + WT + Creepmeters — 2014-10-18
Eee+Enn
Eee-Enn
2Ene
Microstrain
Time
Wavelet pseudo-frequency (Hz)
Creepmeters (XMM1, XMD1, XVA1)
XMM1 slip (mm)
XMD1 slip (mm)
XVA1 slip (mm)
XMM1
XMD1
XVA1

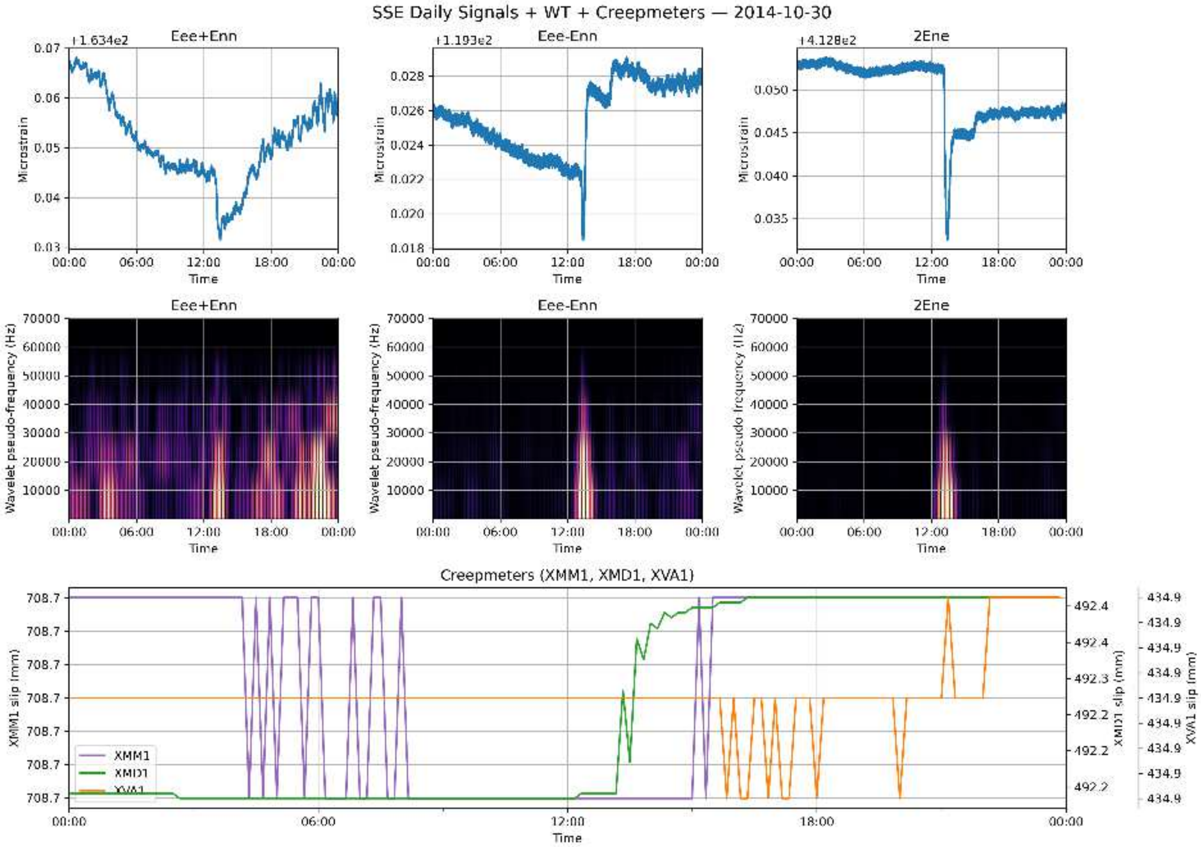
SSE Daily Signals + WT + Creepmeters — 2014-10-30
Eee+Enn
Eee-Enn
2Ene
Microstrain
Time
Wavelet pseudo-frequency (Hz)
Creepmeters (XMM1, XMD1, XVA1)
XMM1 slip (mm)
XMD1 slip (mm)
XVA1 slip (mm)
XMM1
XMD1
XVA1

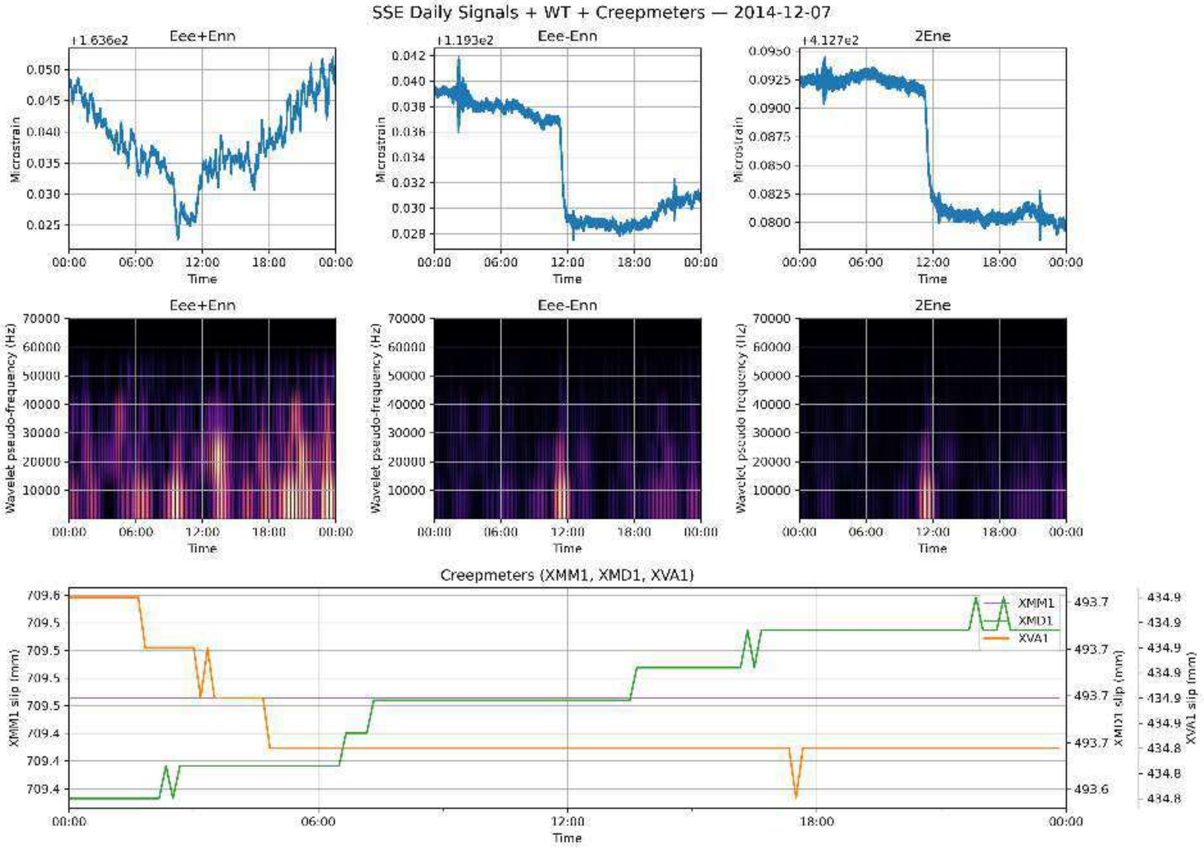
SSE Daily Signals + WT + Creepmeters — 2014-12-07
Eee+Enn
Eee-Enn
2Ene
Microstrain
Time
Wavelet pseudo-frequency (Hz)
Creepmeters (XMM1, XMD1, XVA1)
XMM1
XMD1
XVA1
XMM1 slip (mm)
XMD1 slip (mm)
XVA1 slip (mm)

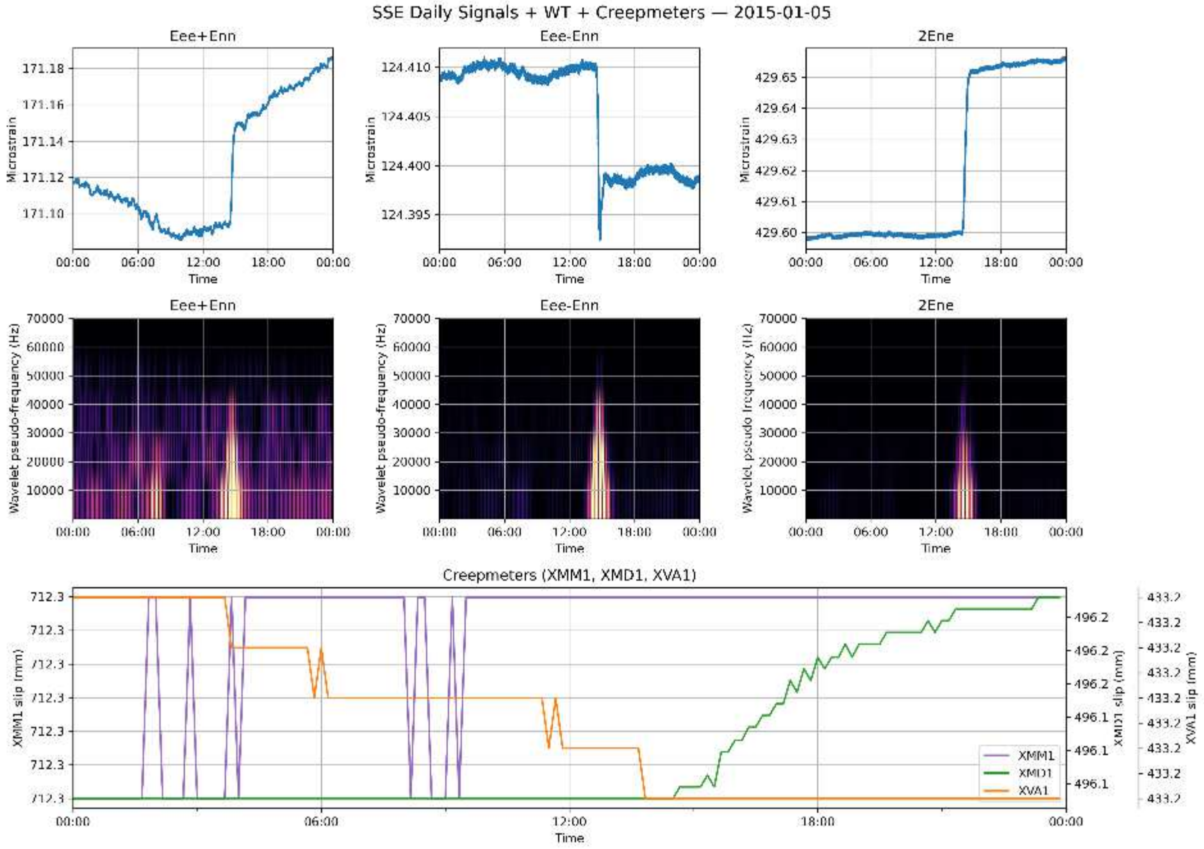
SSE Daily Signals + WT + Creepmeters — 2015-01-05
Eee+Enn
Eee-Enn
2Ene
Microstrain
Time
Wavelet pseudo-frequency (Hz)
Creepmeters (XMM1, XMD1, XVA1)
XMM1
XMD1
XVA1
XMM1 slip (mm)
XMD1 slip (mm)
XVA1 slip (mm)

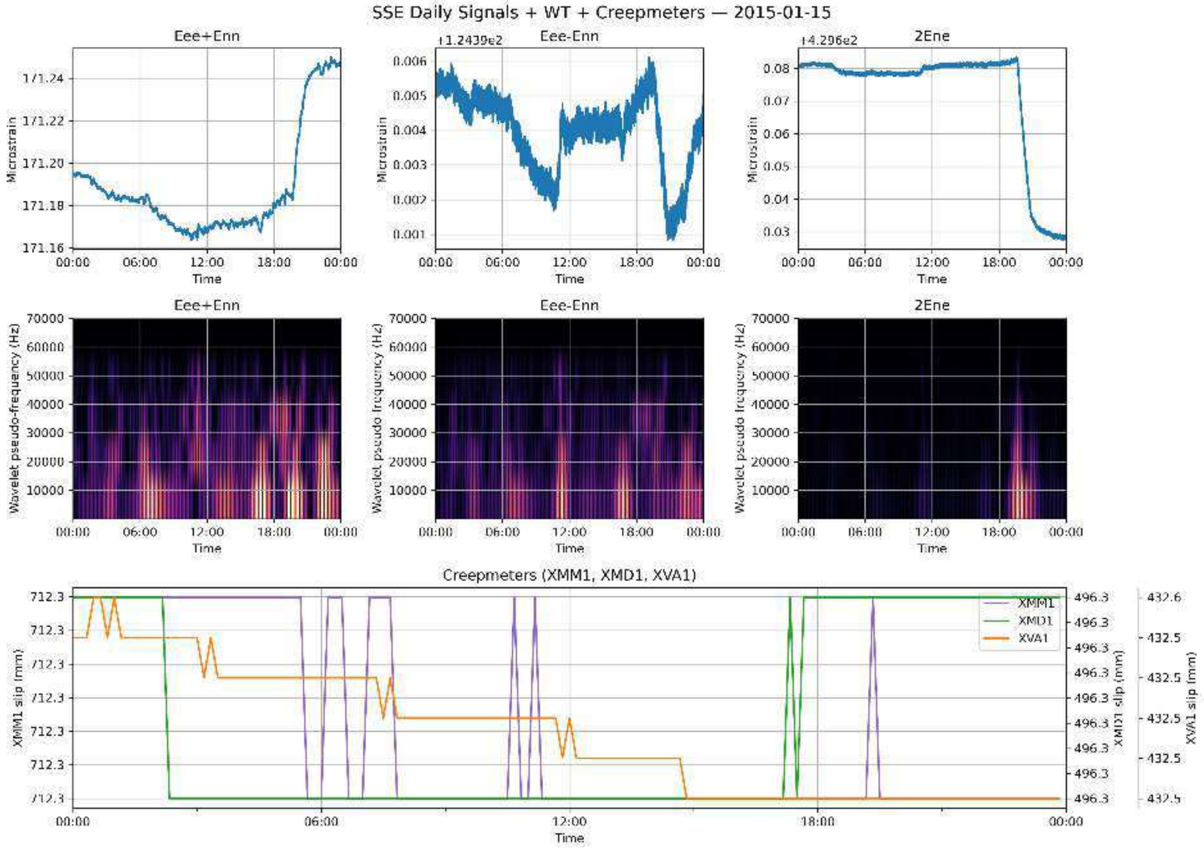

SSE Daily Signals + WT + Creepmeters — 2015-01-15
Eee+Enn
Eee-Enn
2Ene
+1.2439e2
+4.296e2
Microstrain
Time
Wavelet pseudo-frequency (Hz)
Creepmeters (XMM1, XMD1, XVA1)
XMM1
XMD1
XVA1
XMM1 slip (mm)
XMD1 slip (mm)
XVA1 slip (mm)

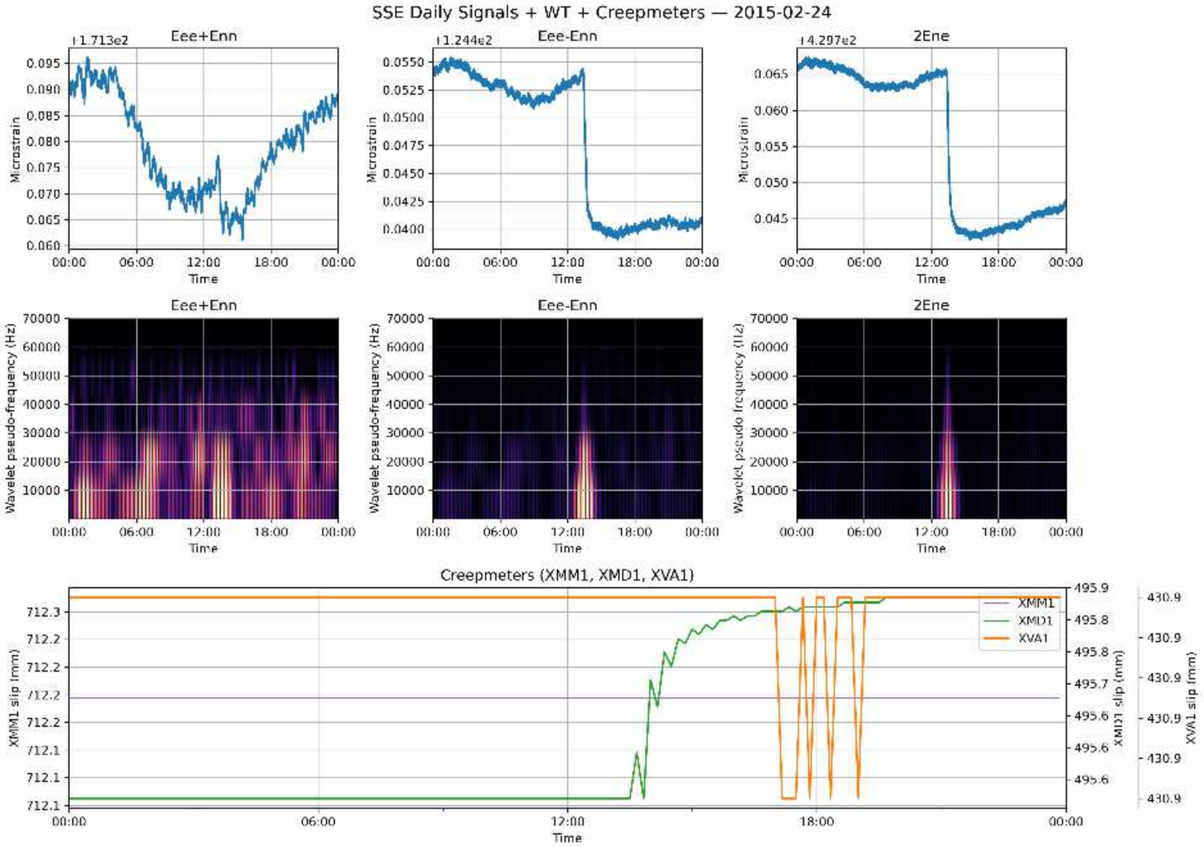

SSE Daily Signals + WT + Creepmeters — 2015-02-24
Eee+Enn
Eee-Enn
2Ene
+1.713e2
+1.244e2
+4.297e2
Microstrain
Time
Wavelet pseudo-frequency (Hz)
Creepmeters (XMM1, XMD1, XVA1)
XMM1
XMD1
XVA1
XMM1 slip (mm)
XMD1 slip (mm)
XVA1 slip (mm)

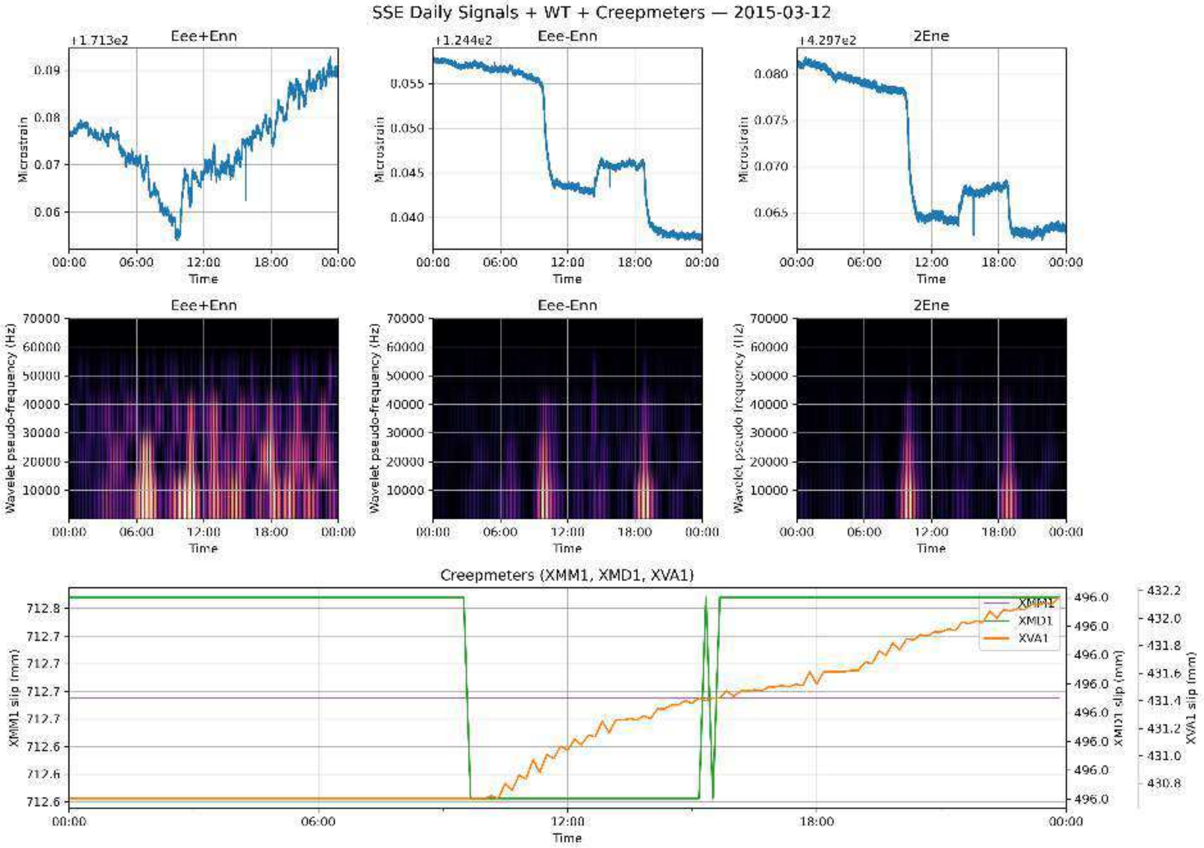
SSE Daily Signals + WT + Creepmeters — 2015-03-12
Eee+Enn
Eee-Enn
2Ene
+1.713e2
+1.244e2
+4.297e2
Microstrain
Time
Wavelet pseudo-frequency (Hz)
Creepmeters (XMM1, XMD1, XVA1)
XMM1
XMD1
XVA1
XMM1 slip (mm)
XMD1 slip (mm)
XVA1 slip (mm)

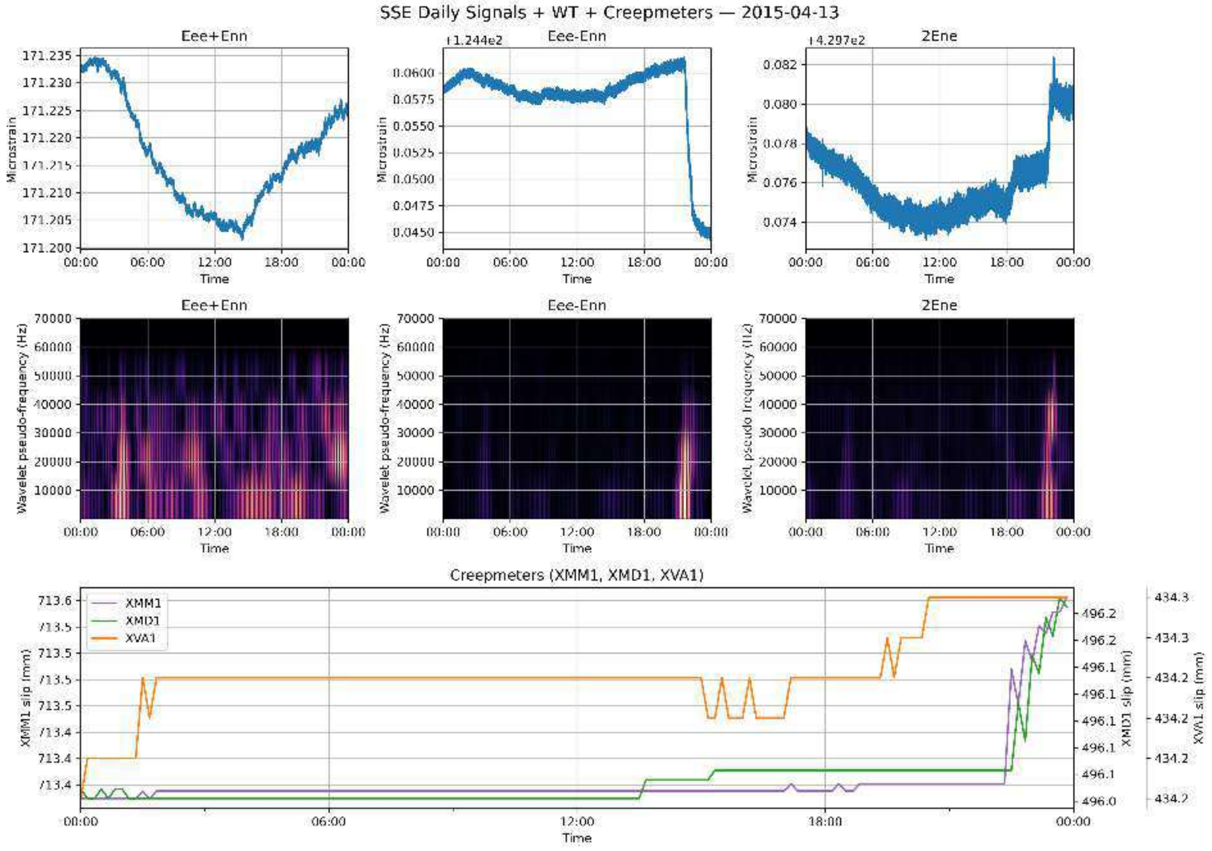
SSE Daily Signals + WT + Creepmeters — 2015-04-13
Eee+Enn
Eee-Enn
2Ene
+1.244e2
+4.297e2
Microstrain
Time
Wavelet pseudo-frequency (Hz)
Creepmeters (XMM1, XMD1, XVA1)
XMM1
XMD1
XVA1
XMM1 slip (mm)
XMD1 slip (mm)
XVA1 slip (mm)

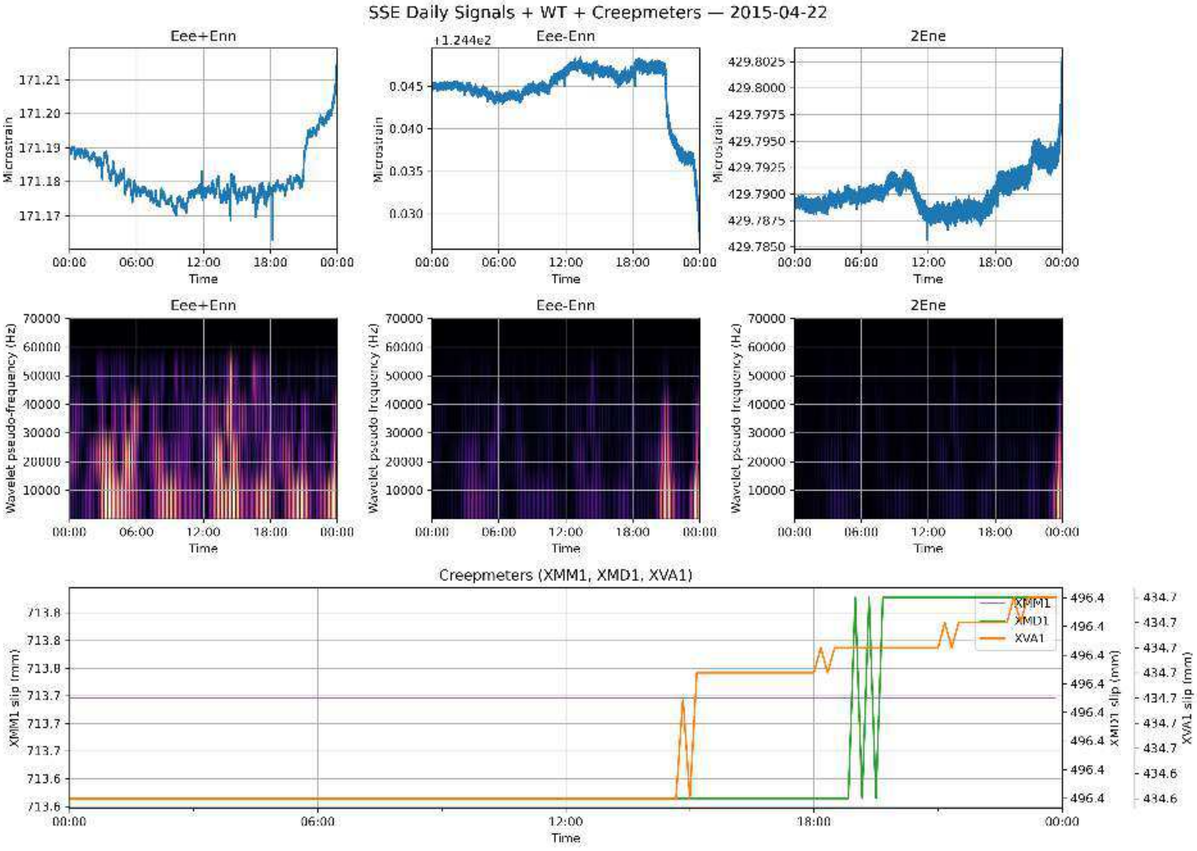

SSE Daily Signals + WT + Creepmeters — 2015-04-22
Eee+Enn
Ee-Enn
2Ene
Microstrain
Time
Wavelet pseudo-frequency (Hz)
Creepmeters (XMM1, XMD1, XVA1)
XMM1
XMD1
XVA1
XMM1 slip (mm)
XMD1 slip (mm)
XVA1 slip (mm)

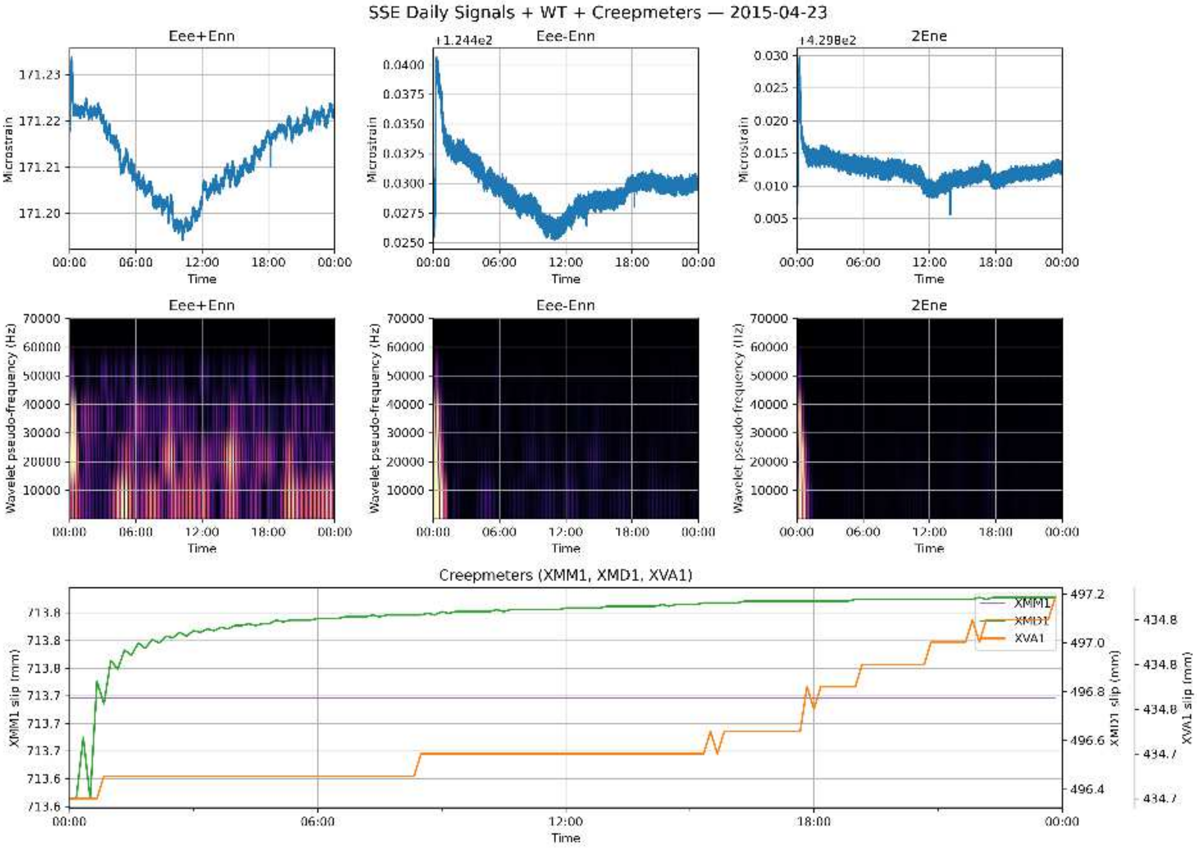

SSE Daily Signals + WT + Creepmeters — 2015-04-23
Eee+Enn
Ee-Enn
2Ene
Microstrain
Time
Wavelet pseudo-frequency (Hz)
Creepmeters (XMM1, XMD1, XVA1)
XMM1
XMD1
XVA1
XMM1 slip (mm)
XMD1 slip (mm)
XVA1 slip (mm)

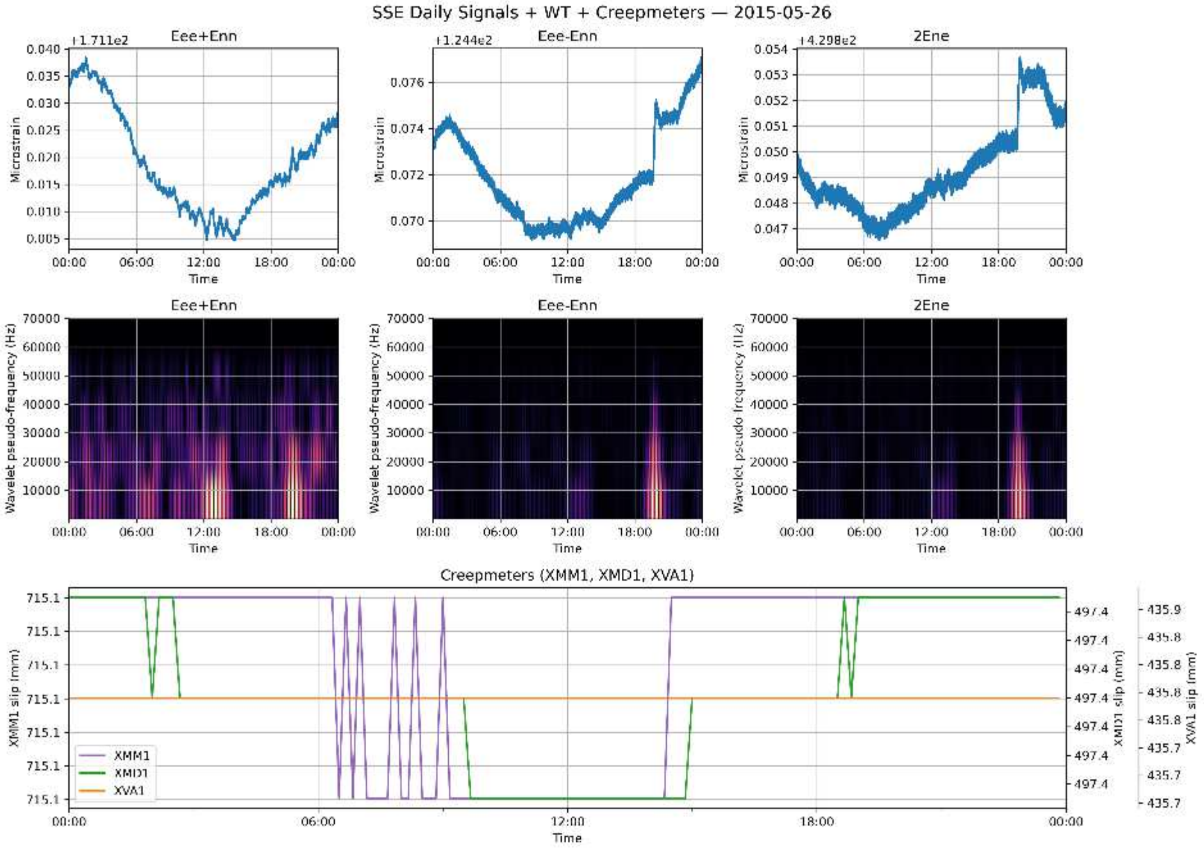
SSE Daily Signals + WT + Creepmeters — 2015-05-26
Eee+Enn
Eee-Enn
2Ene
+1.711e2
+1.244e2
+4.298e2
Microstrain
Time
Wavelet pseudo-frequency (Hz)
Creepmeters (XMM1, XMD1, XVA1)
XMM1 slip (mm)
XMD1 slip (mm)
XVA1 slip (mm)
XMM1
XMD1
XVA1

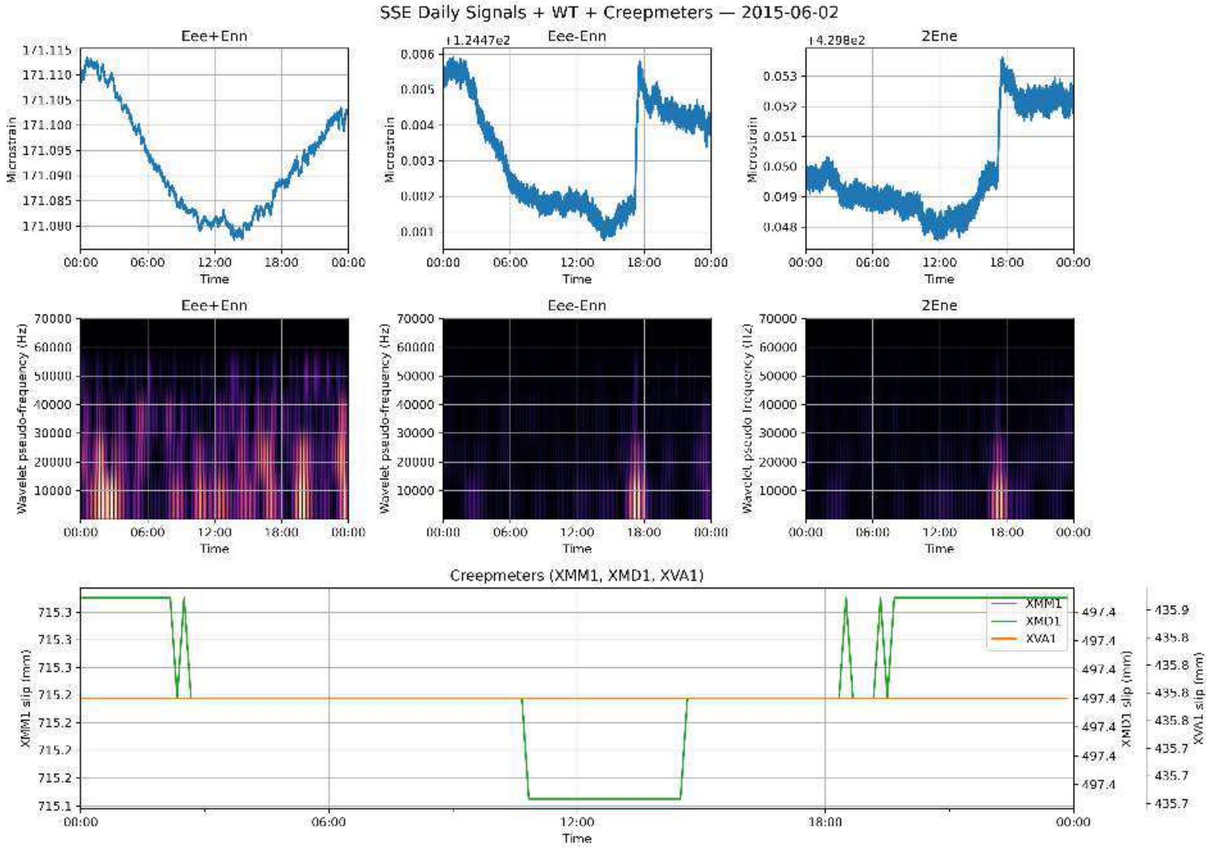
SSE Daily Signals + WT + Creepmeters — 2015-06-02
Eee+Enn
Eee-Enn
2Ene
+1.2447e2
+4.298e2
Microstrain
Time
Wavelet pseudo-frequency (Hz)
Creepmeters (XMM1, XMD1, XVA1)
XMM1 slip (mm)
XMD1 slip (mm)
XVA1 slip (mm)
XMM1
XMD1
XVA1

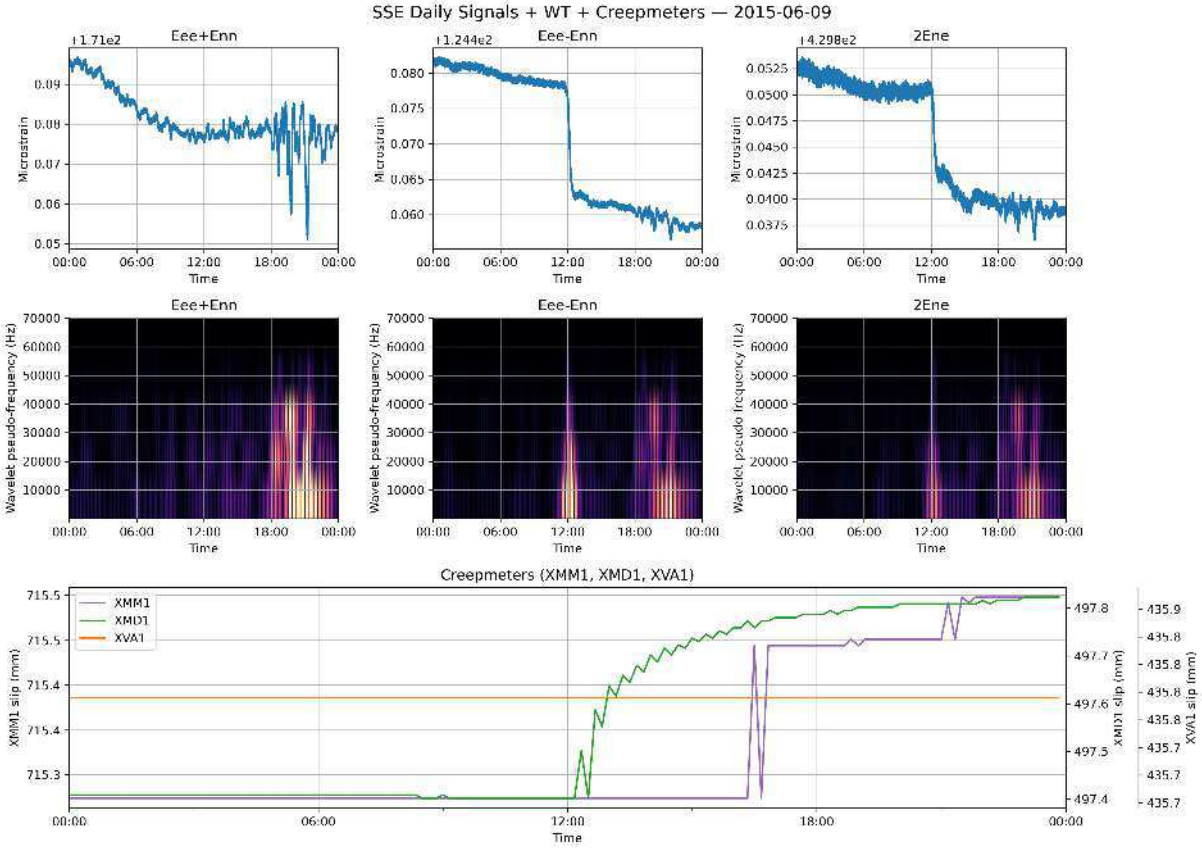

SSE Daily Signals + WT + Creepmeters — 2015-06-09
Eee+Enn
Eee-Enn
2Ene
+1.71e2
+1.244e2
+4.298e2
Microstrain
Time
Wavelet pseudo-frequency (Hz)
Creepmeters (XMM1, XMD1, XVA1)
XMM1
XMD1
XVA1
XMM1 slip (mm)
XMD1 slip (mm)
XVA1 slip (mm)

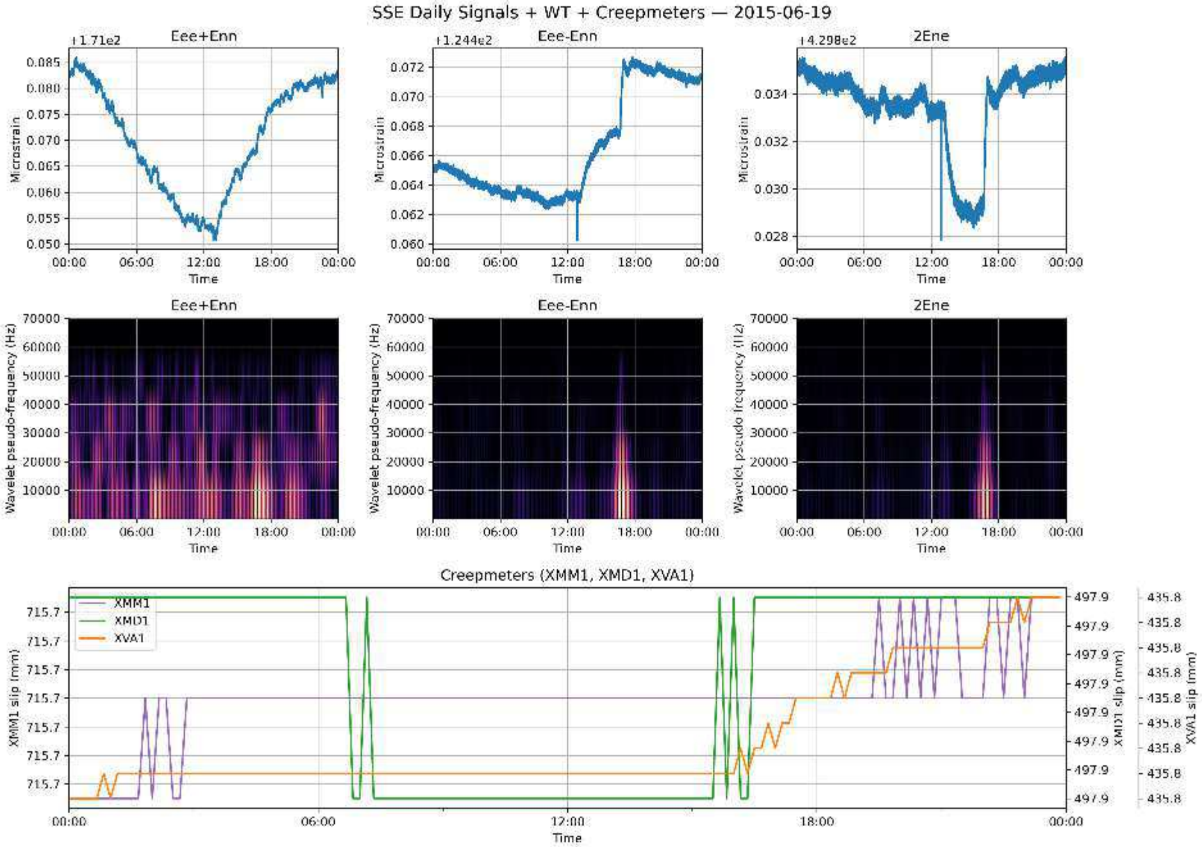

SSE Daily Signals + WT + Creepmeters — 2015-06-19
Eee+Enn
Eee-Enn
2Ene
+1.71e2
+1.244e2
+4.298e2
Microstrain
Time
Wavelet pseudo-frequency (Hz)
Creepmeters (XMM1, XMD1, XVA1)
XMM1
XMD1
XVA1
XMM1 slip (mm)
XMD1 slip (mm)
XVA1 slip (mm)

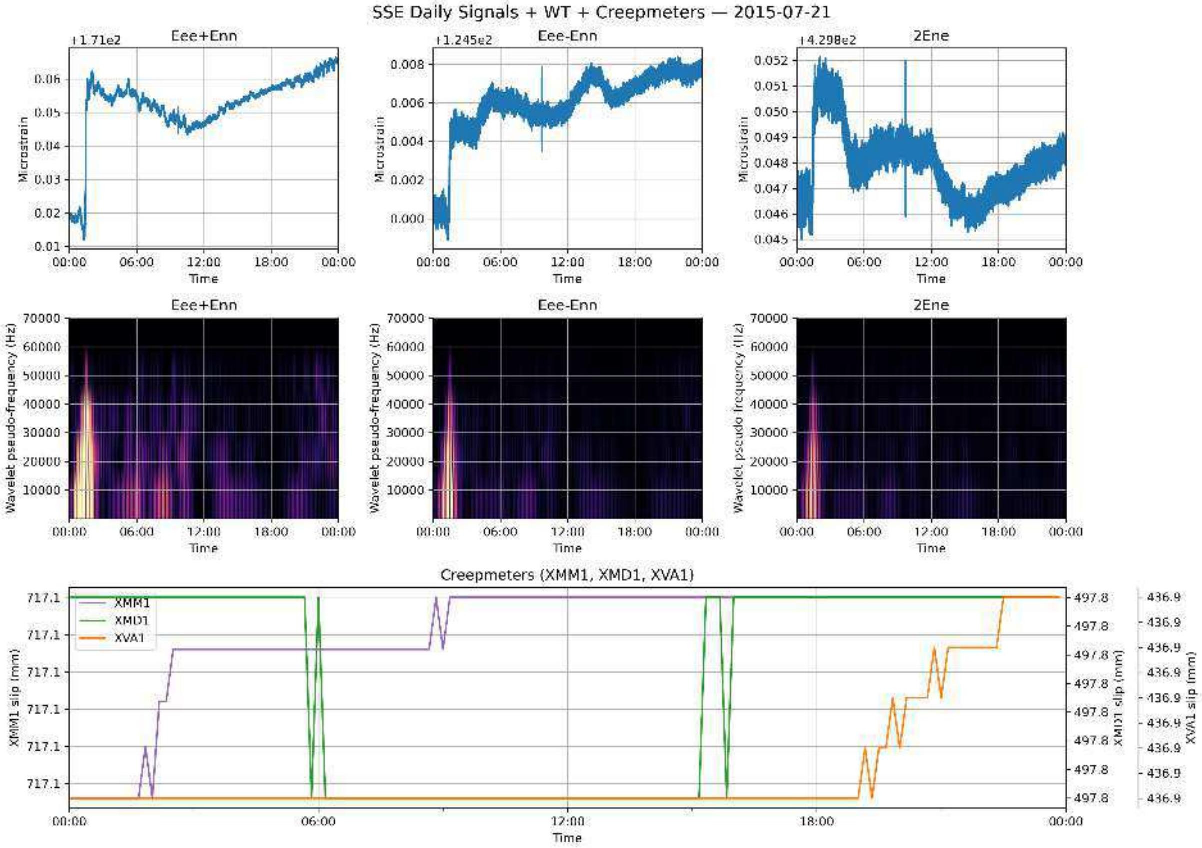
SSE Daily Signals + WT + Creepmeters — 2015-07-21
Eee+Enn
Eee-Enn
2Ene
Microstrain
Time
Wavelet pseudo-frequency (Hz)
Creepmeters (XMM1, XMD1, XVA1)
XMM1
XMD1
XVA1
XMM1 slip (mm)
XMD1 slip (mm)
XVA1 slip (mm)

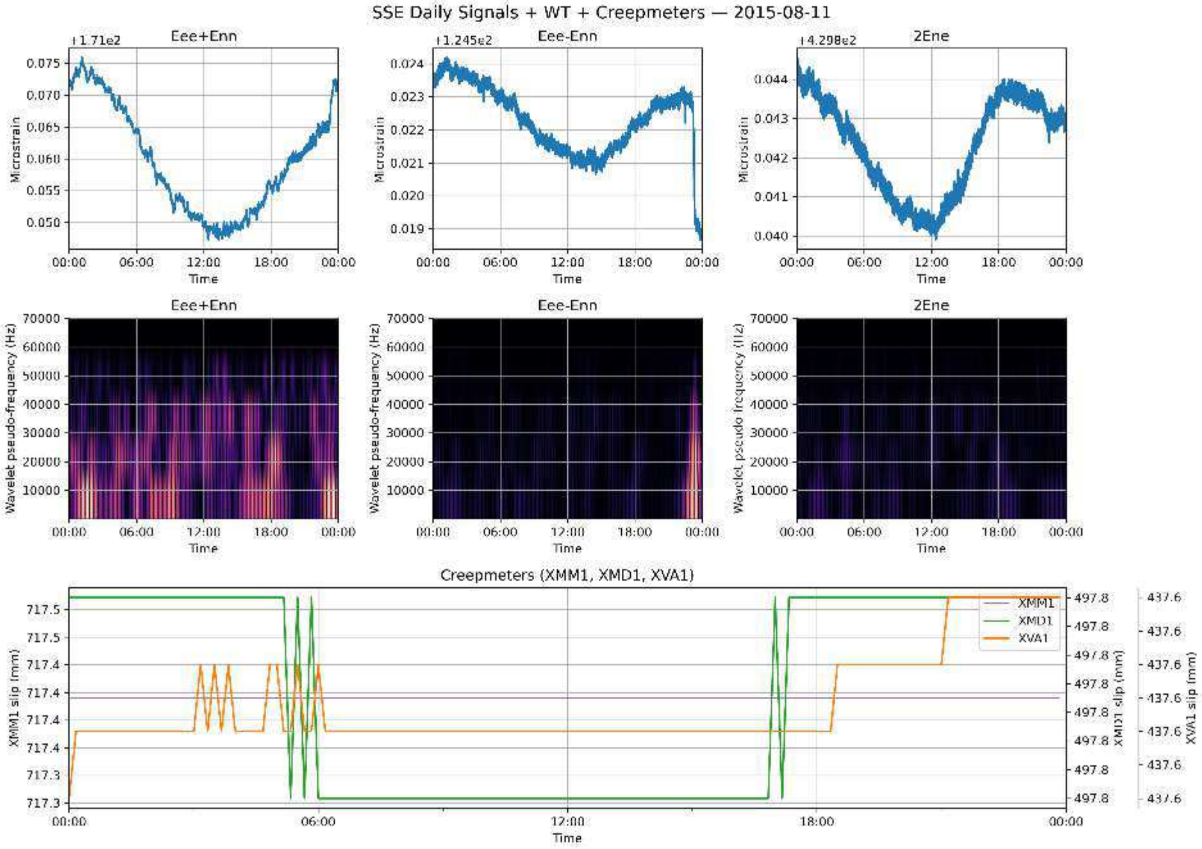
SSE Daily Signals + WT + Creepmeters — 2015-08-11
Eee+Enn
Eee-Enn
2Ene
Microstrain
Time
Wavelet pseudo-frequency (Hz)
Creepmeters (XMM1, XMD1, XVA1)
XMM1
XMD1
XVA1
XMM1 slip (mm)
XMD1 slip (mm)
XVA1 slip (mm)

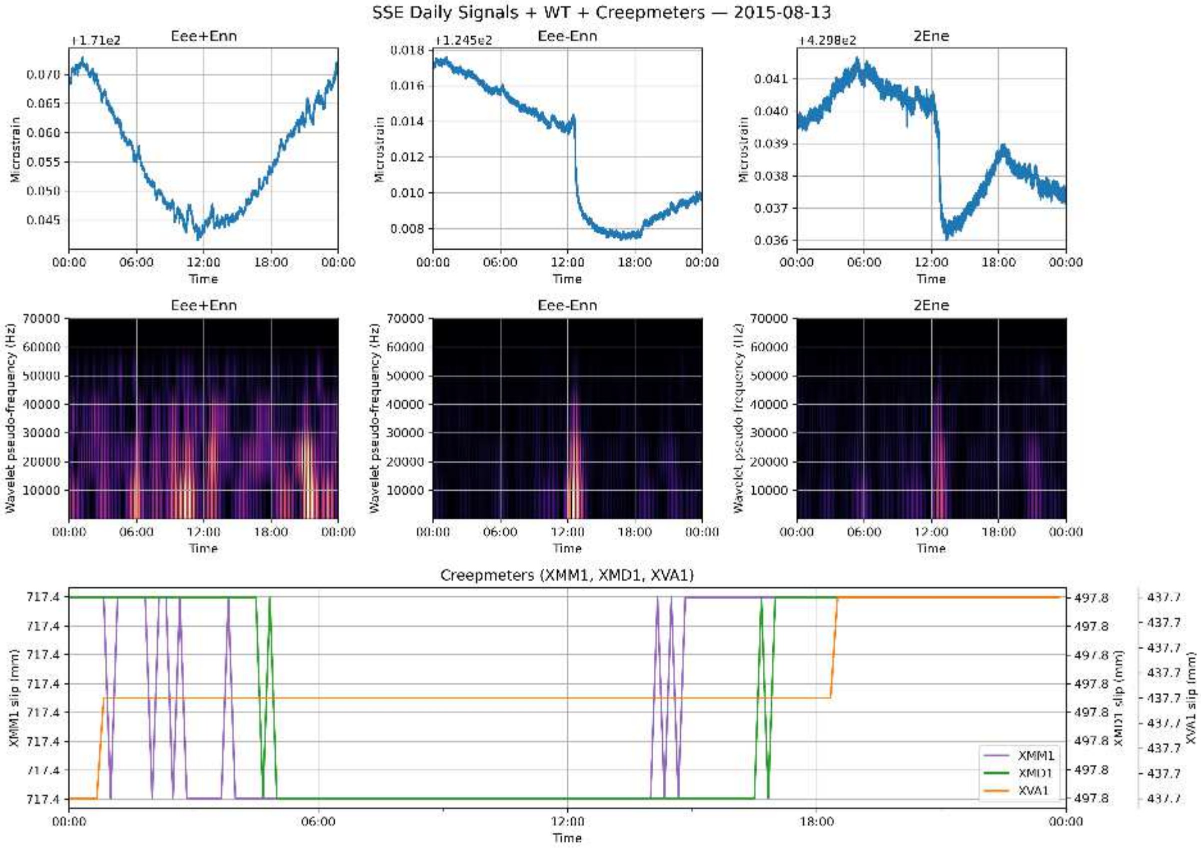
SSE Daily Signals + WT + Creepmeters — 2015-08-13
Eee+Enn
Eee-Enn
2Ene
+1.71e2
+1.245e2
+4.298e2
Microstrain
Time
Wavelet pseudo-frequency (Hz)
Creepmeters (XMM1, XMD1, XVA1)
XMM1 slip (mm)
XMD1 slip (mm)
XVA1 slip (mm)
XMM1
XMD1
XVA1

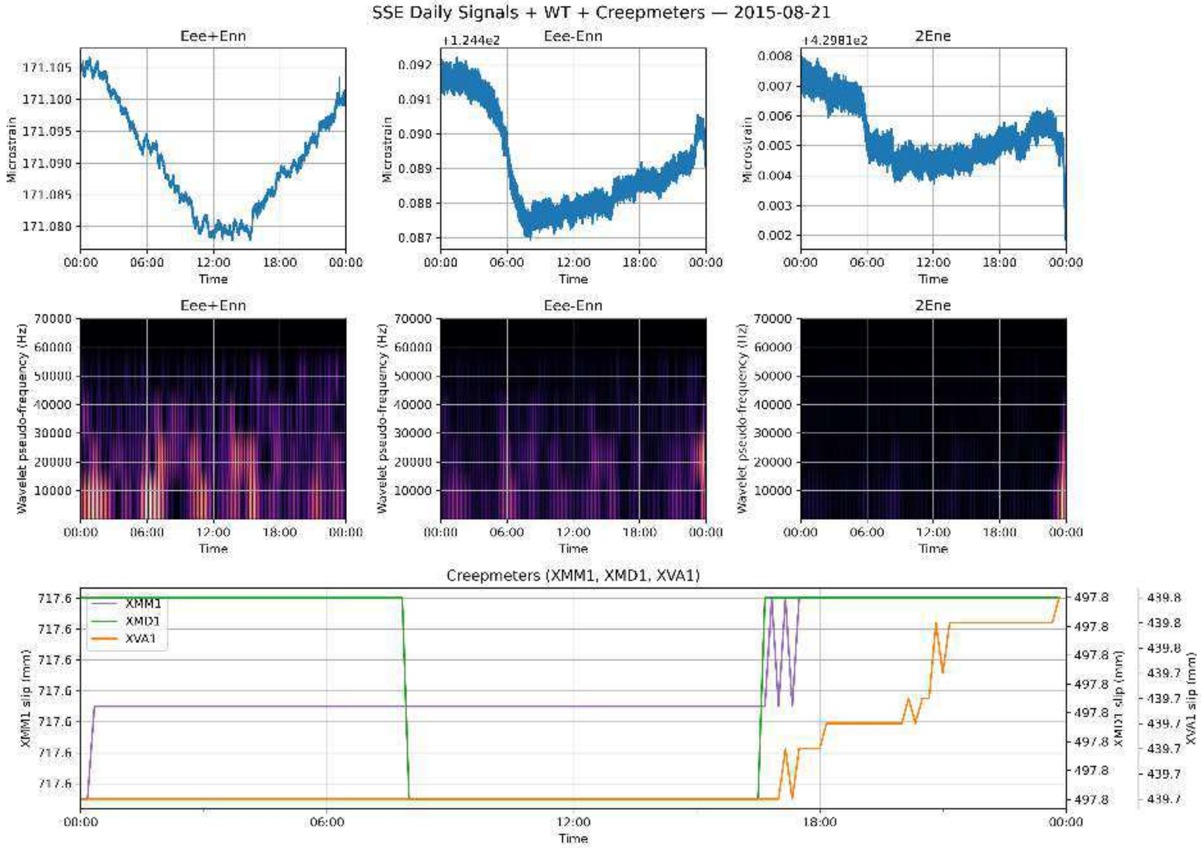
SSE Daily Signals + WT + Creepmeters — 2015-08-21
Eee+Enn
Eee-Enn
2Ene
+1.244e2
+4.2981e2
Microstrain
Time
Wavelet pseudo-frequency (Hz)
Creepmeters (XMM1, XMD1, XVA1)
XMM1 slip (mm)
XMD1 slip (mm)
XVA1 slip (mm)
XMM1
XMD1
XVA1

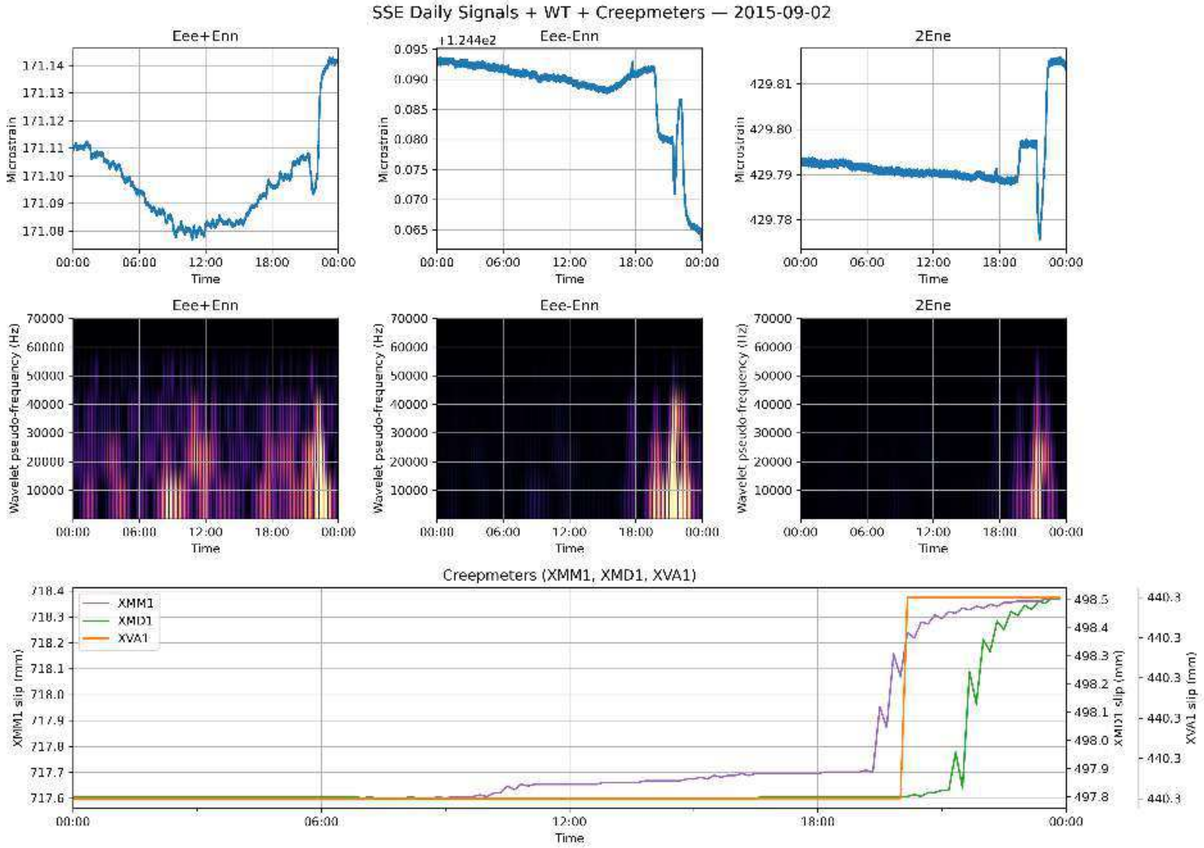
SSE Daily Signals + WT + Creepmeters — 2015-09-02
Eee+Enn
Eee-Enn
2Ene
Microstrain
Time
Wavelet pseudo-frequency (Hz)
Creepmeters (XMM1, XMD1, XVA1)
XMM1
XMD1
XVA1
XMM1 slip (mm)
XMD1 slip (mm)
XVA1 slip (mm)

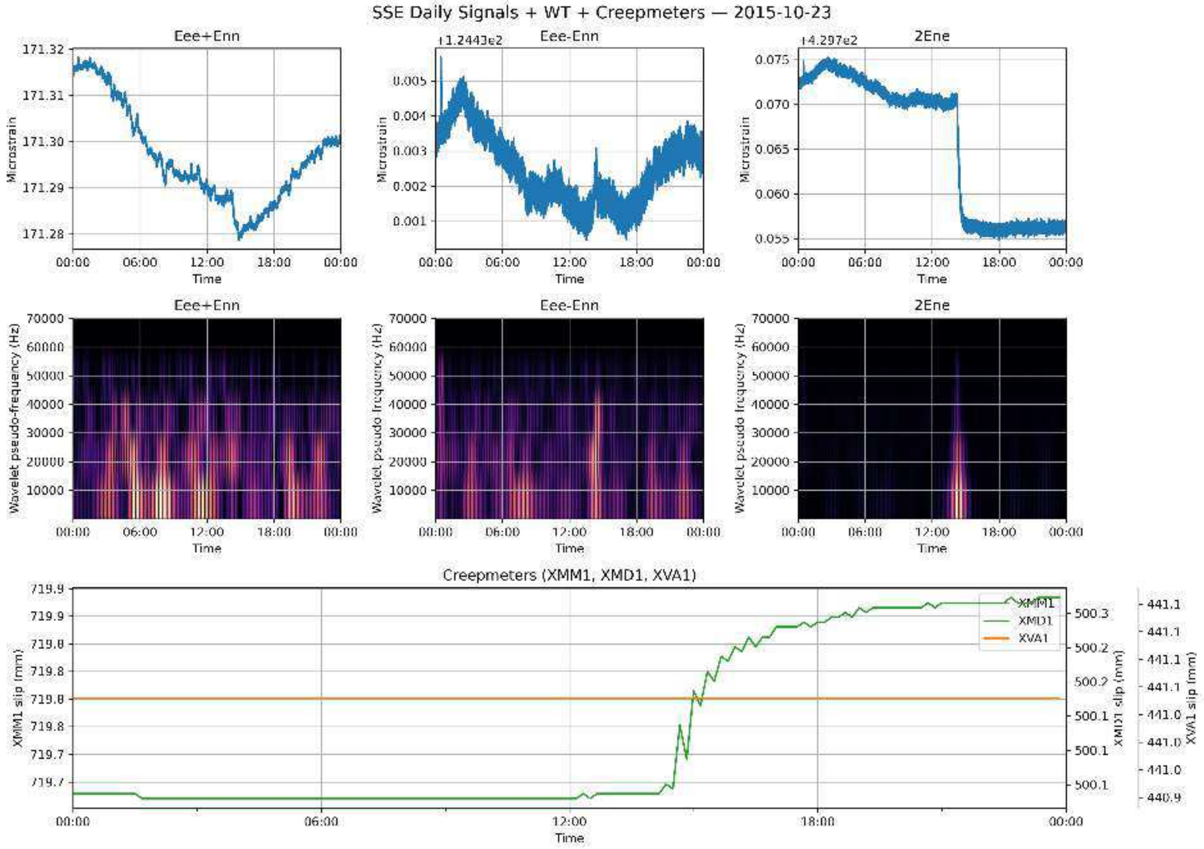
SSE Daily Signals + WT + Creepmeters — 2015-10-23
Eee+Enn
Eee-Enn
2Ene
Microstrain
Time
Wavelet pseudo-frequency (Hz)
Creepmeters (XMM1, XMD1, XVA1)
XMM1
XMD1
XVA1
XMM1 slip (mm)
XMD1 slip (mm)
XVA1 slip (mm)

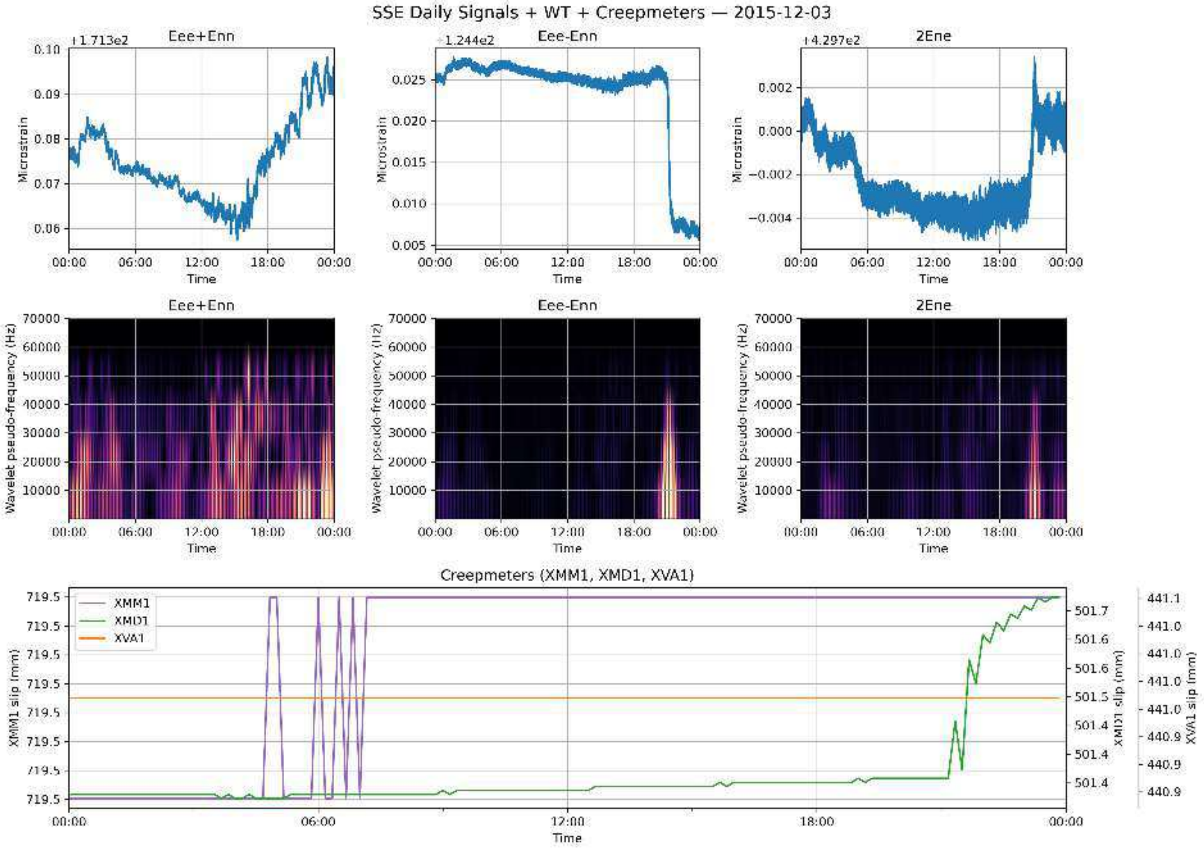
SSE Daily Signals + WT + Creepmeters — 2015-12-03
Eee+Enn
Eee-Enn
2Ene
Microstrain
Time
Wavelet pseudo-frequency (Hz)
Creepmeters (XMM1, XMD1, XVA1)
XMM1
XMD1
XVA1
XMM1 slip (mm)
XMD1 slip (mm)
XVA1 slip (mm)

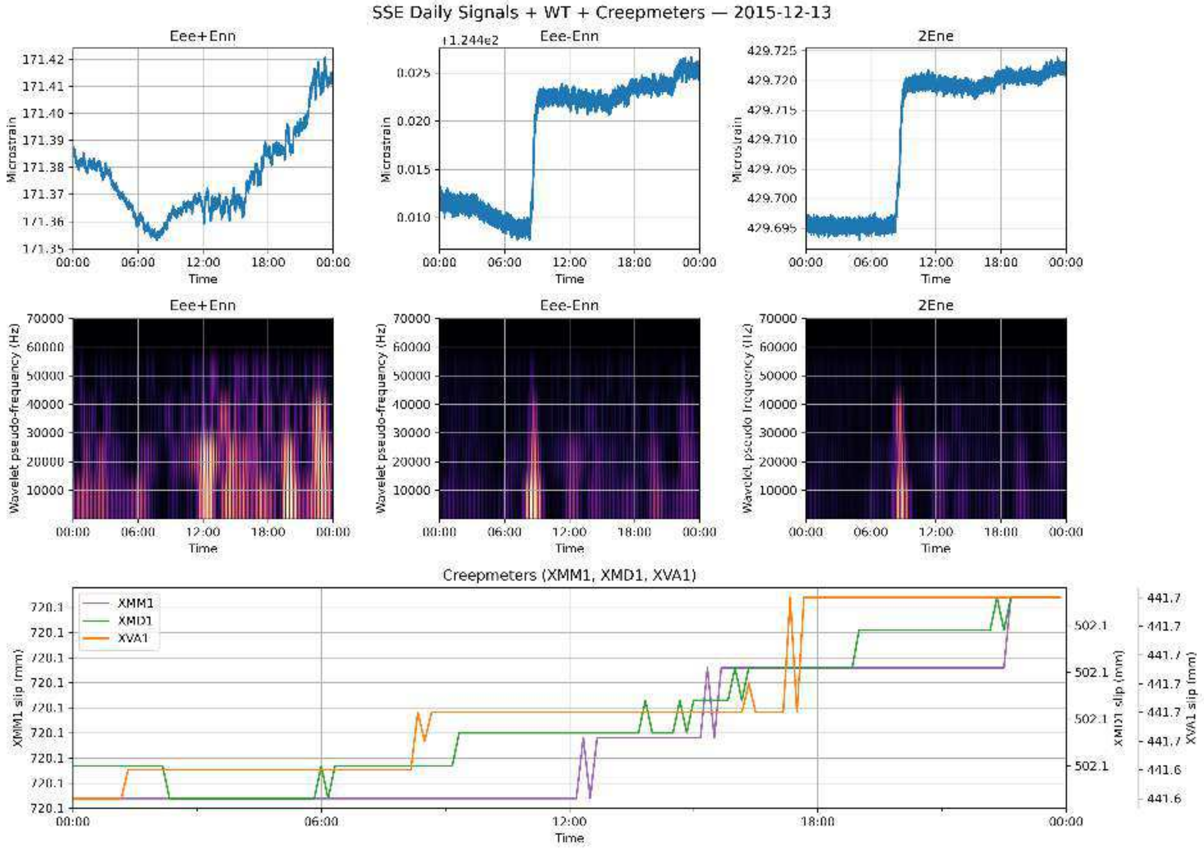
SSE Daily Signals + WT + Creepmeters — 2015-12-13
Eee+Enn
Eee-Enn
2Ene
Microstrain
Time
Wavelet pseudo-frequency (Hz)
Creepmeters (XMM1, XMD1, XVA1)
XMM1
XMD1
XVA1
XMM1 slip (mm)
XMD1 slip (mm)
XVA1 slip (mm)

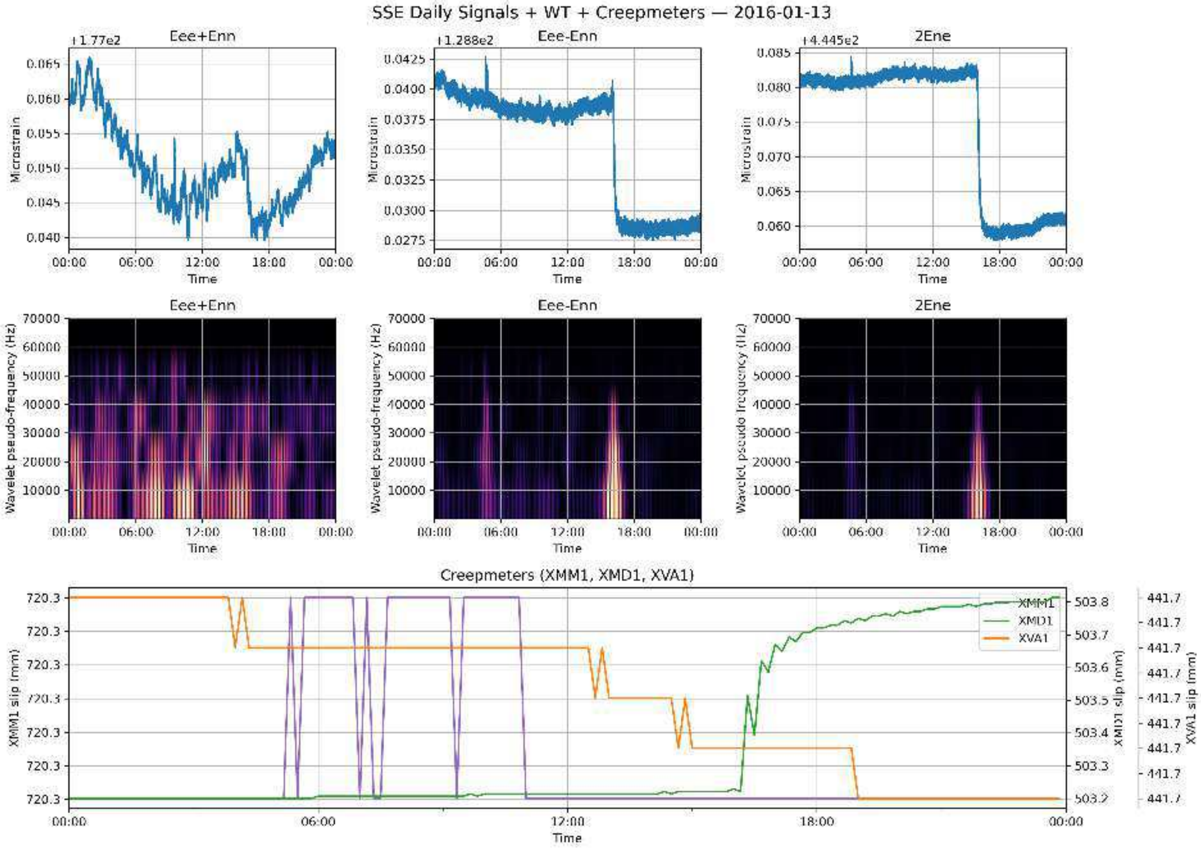
SSE Daily Signals + WT + Creepmeters — 2016-01-13
Eee+Enn
Eee-Enn
2Ene
Microstrain
Time
Wavelet pseudo-frequency (Hz)
Creepmeters (XMM1, XMD1, XVA1)
XMM1
XMD1
XVA1
XMM1 slip (mm)
XMD1 slip (mm)
XVA1 slip (mm)

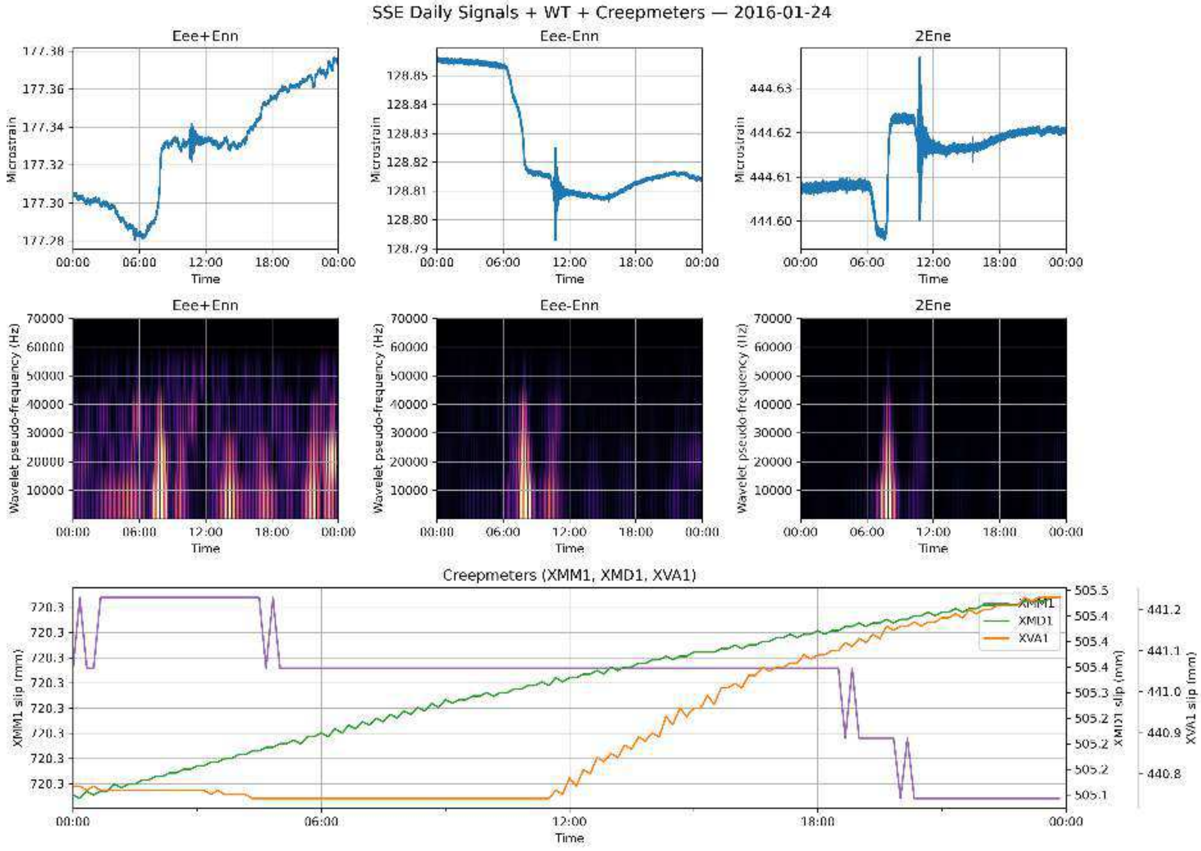
SSE Daily Signals + WT + Creepmeters — 2016-01-24
Eee+Enn
Eee-Enn
2Ene
Microstrain
Time
Wavelet pseudo-frequency (Hz)
Creepmeters (XMM1, XMD1, XVA1)
XMM1
XMD1
XVA1
XMM1 slip (mm)
XMD1 slip (mm)
XVA1 slip (mm)

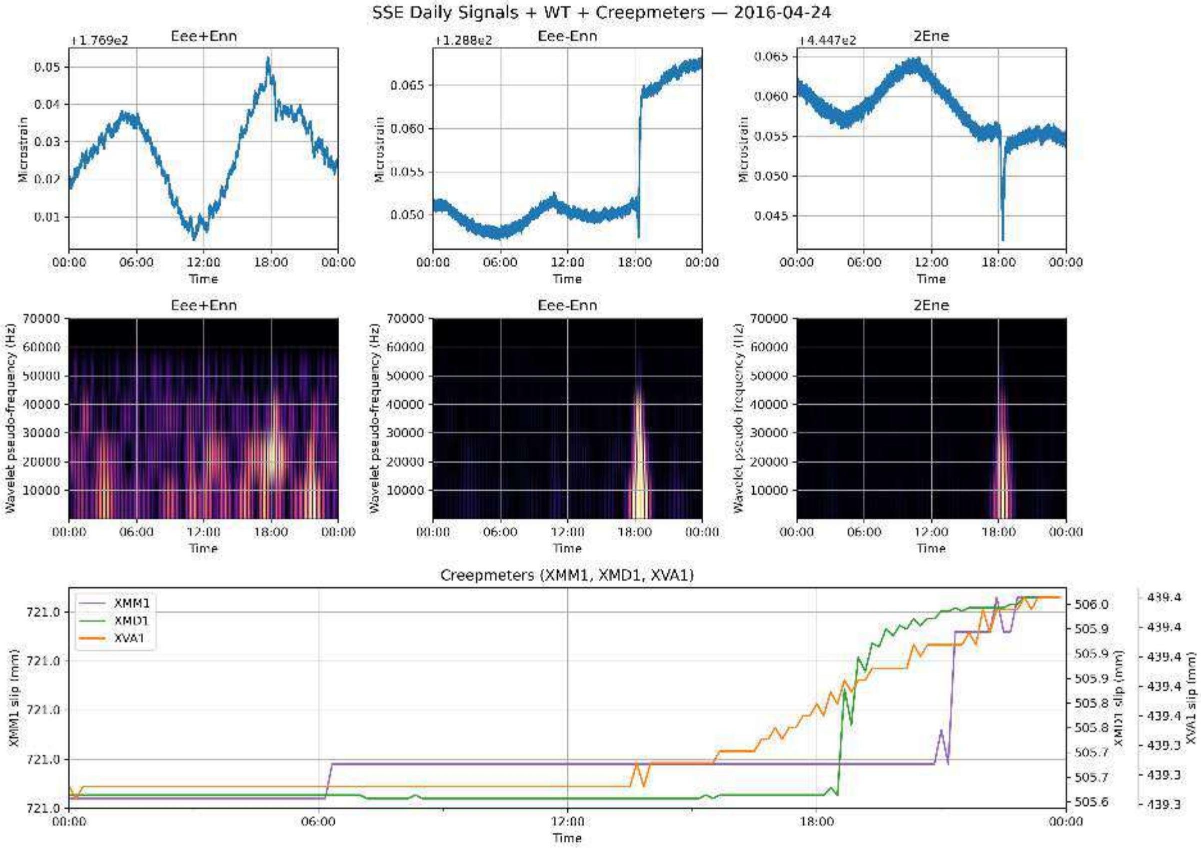
SSE Daily Signals + WT + Creepmeters — 2016-04-24
Eee+Enn
Eee-Enn
2Ene
Microstrain
Time
Wavelet pseudo-frequency (Hz)
Creepmeters (XMM1, XMD1, XVA1)
XMM1
XMD1
XVA1
XMM1 slip (mm)
XMD1 slip (mm)
XVA1 slip (mm)

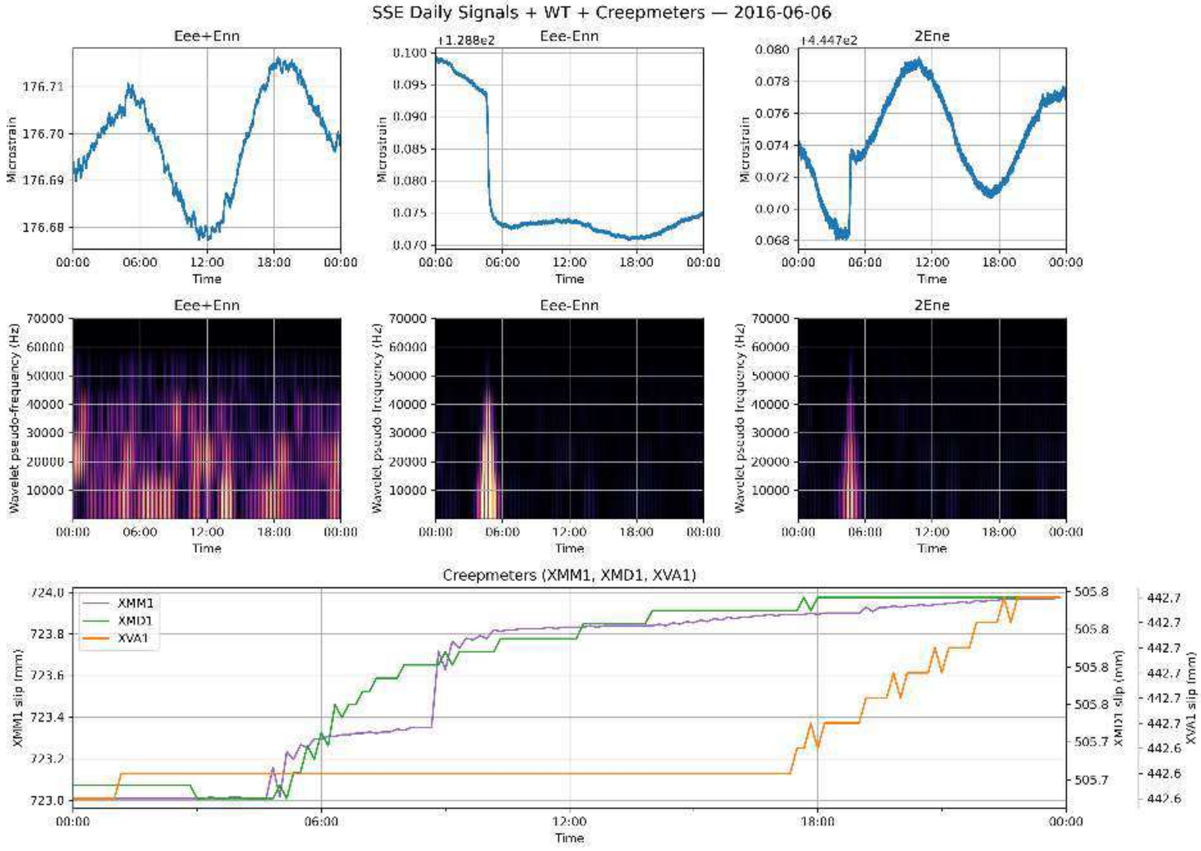
SSE Daily Signals + WT + Creepmeters — 2016-06-06
Eee+Enn
Eee-Enn
2Ene
Microstrain
Time
Wavelet pseudo-frequency (Hz)
Creepmeters (XMM1, XMD1, XVA1)
XMM1
XMD1
XVA1
XMM1 slip (mm)
XMD1 slip (mm)
XVA1 slip (mm)

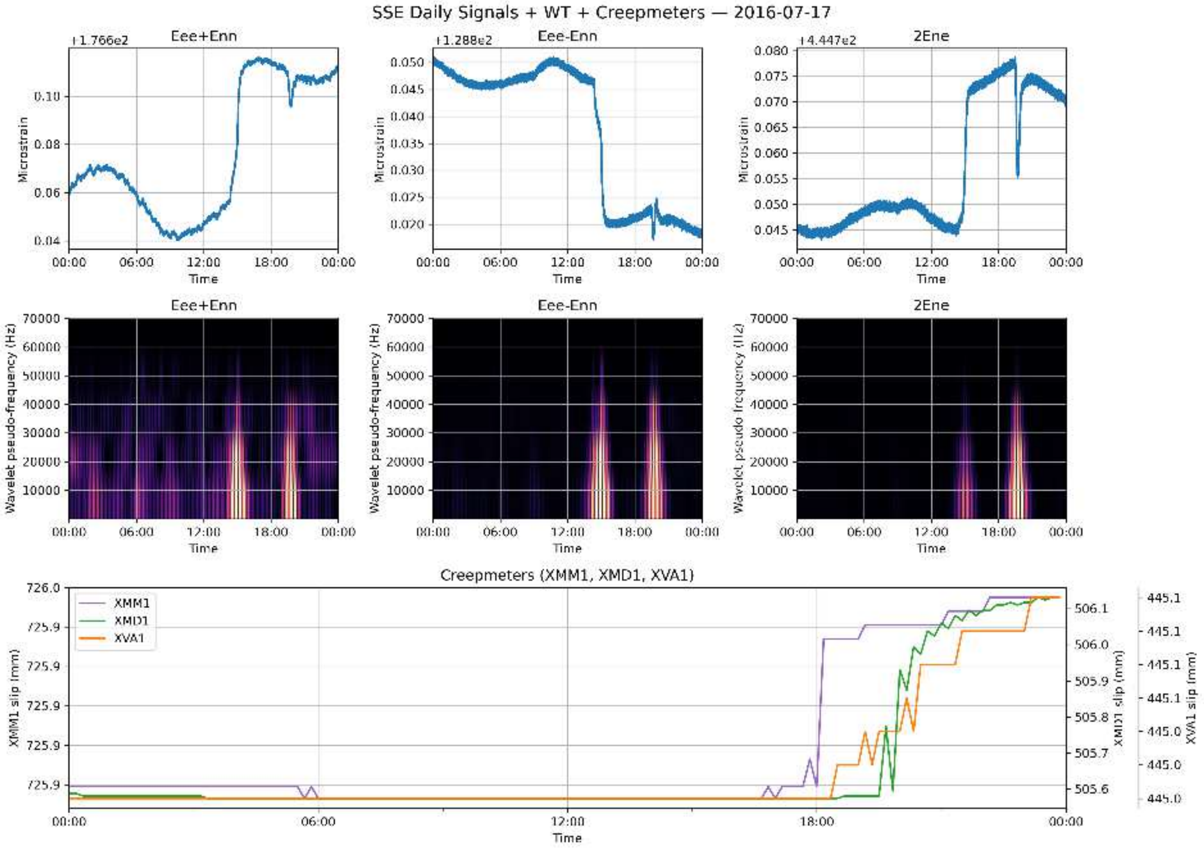
SSE Daily Signals + WT + Creepmeters — 2016-07-17
Eee+Enn
Eee-Enn
2Ene
Microstrain
Time
Wavelet pseudo-frequency (Hz)
Creepmeters (XMM1, XMD1, XVA1)
XMM1
XMD1
XVA1
XMM1 slip (mm)
XMD1 slip (mm)
XVA1 slip (mm)

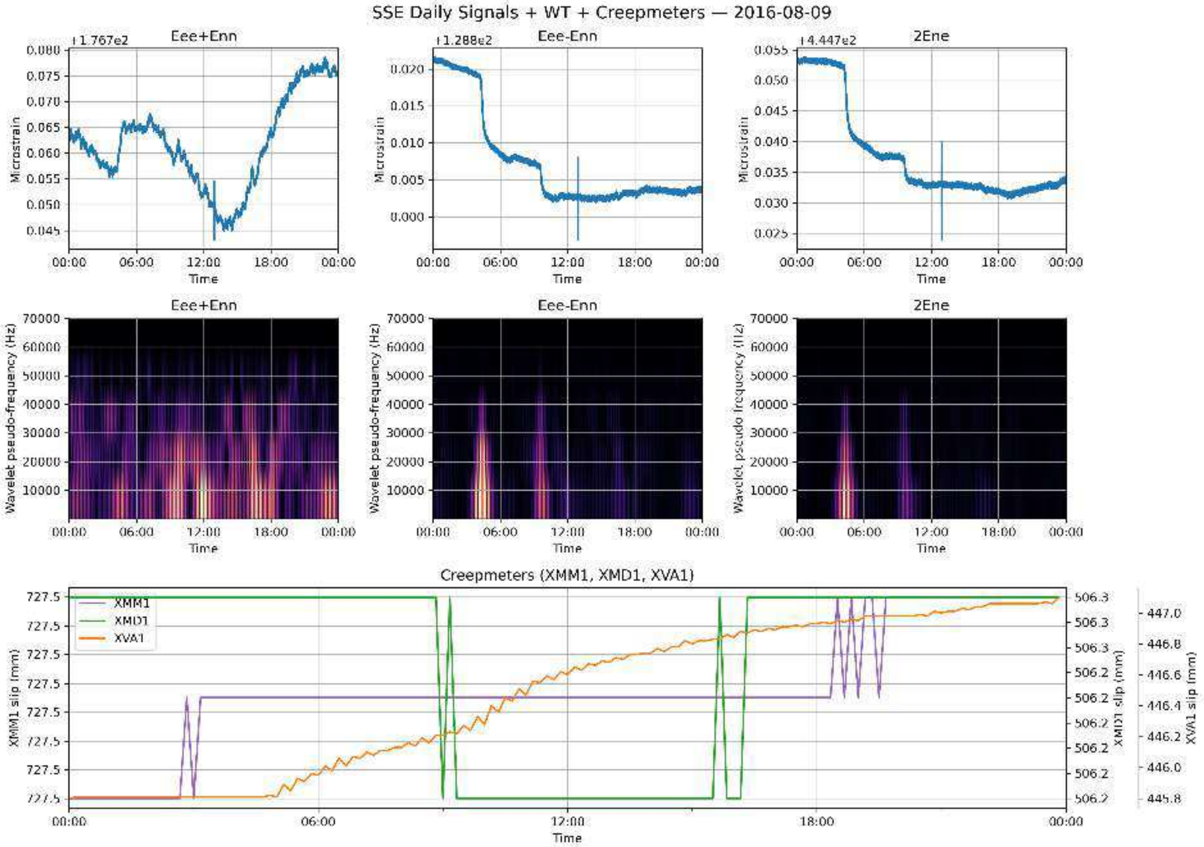
SSE Daily Signals + WT + Creepmeters — 2016-08-09
Eee+Enn
Eee-Enn
2Ene
Microstrain
Time
Wavelet pseudo-frequency (Hz)
Creepmeters (XMM1, XMD1, XVA1)
XMM1
XMD1
XVA1
XMM1 slip (mm)
XMD1 slip (mm)
XVA1 slip (mm)

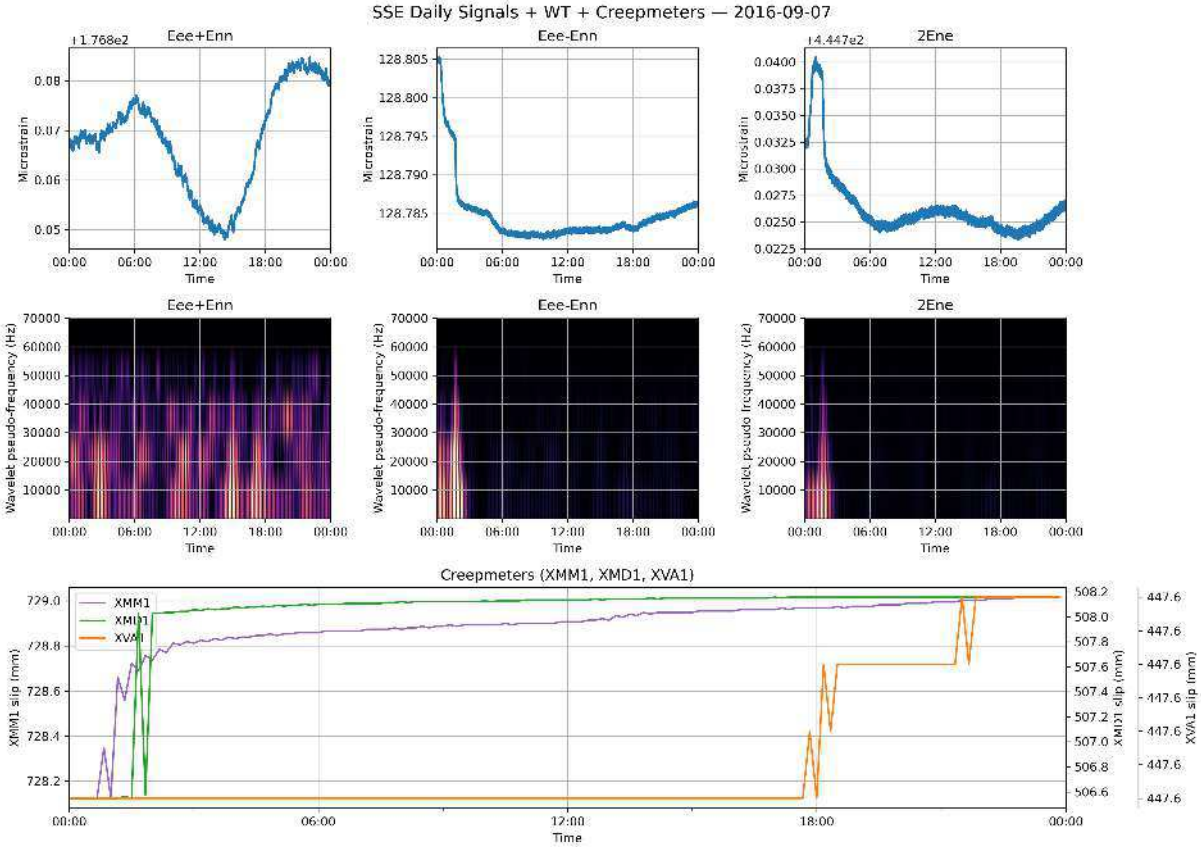
SSE Daily Signals + WT + Creepmeters — 2016-09-07
Eee+Enn
Eee-Enn
2Ene
Microstrain
Time
Wavelet pseudo-frequency (Hz)
Creepmeters (XMM1, XMD1, XVA1)
XMM1
XMD1
XVA1
XMM1 slip (mm)
XMD1 slip (mm)
XVA1 slip (mm)

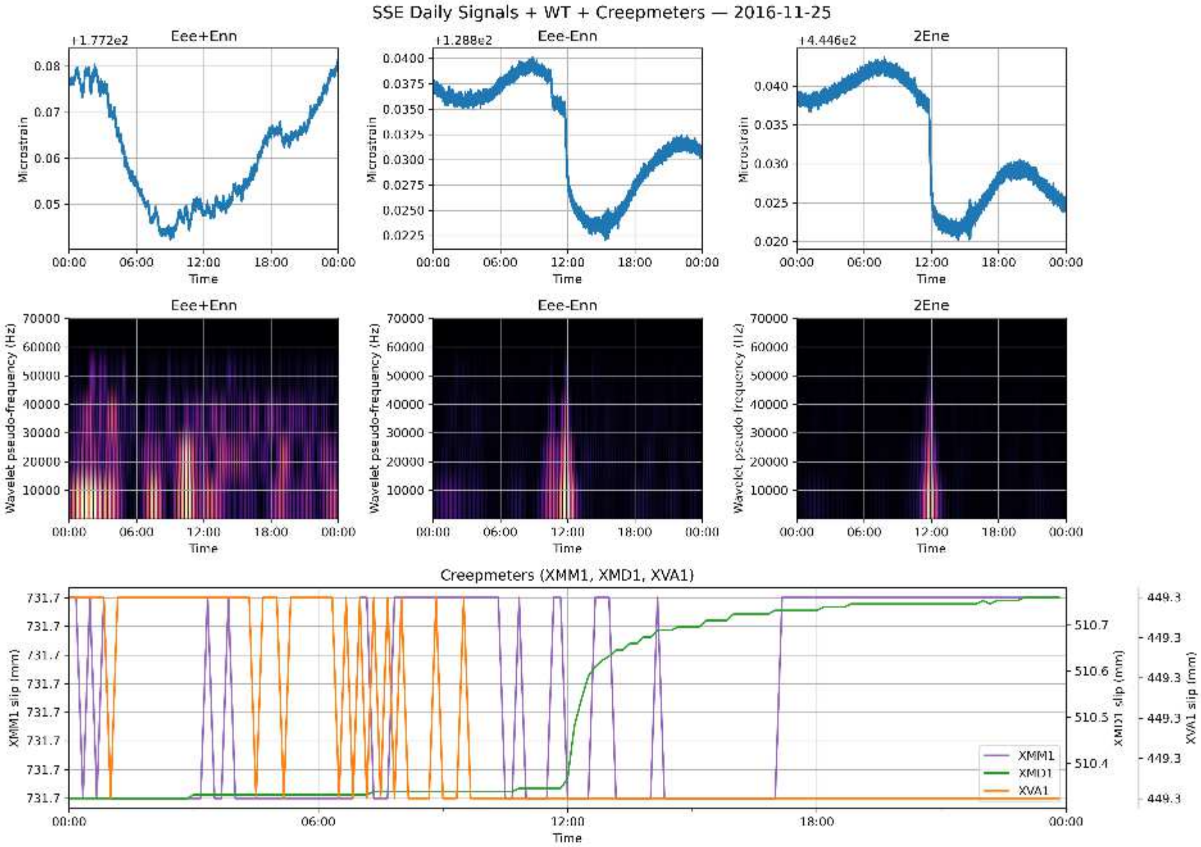
SSE Daily Signals + WT + Creepmeters — 2016-11-25
Eee+Enn
Eee-Enn
2Ene
Microstrain
Time
Wavelet pseudo-frequency (Hz)
Creepmeters (XMM1, XMD1, XVA1)
XMM1
XMD1
XVA1
XMM1 slip (mm)
XMD1 slip (mm)
XVA1 slip (mm)

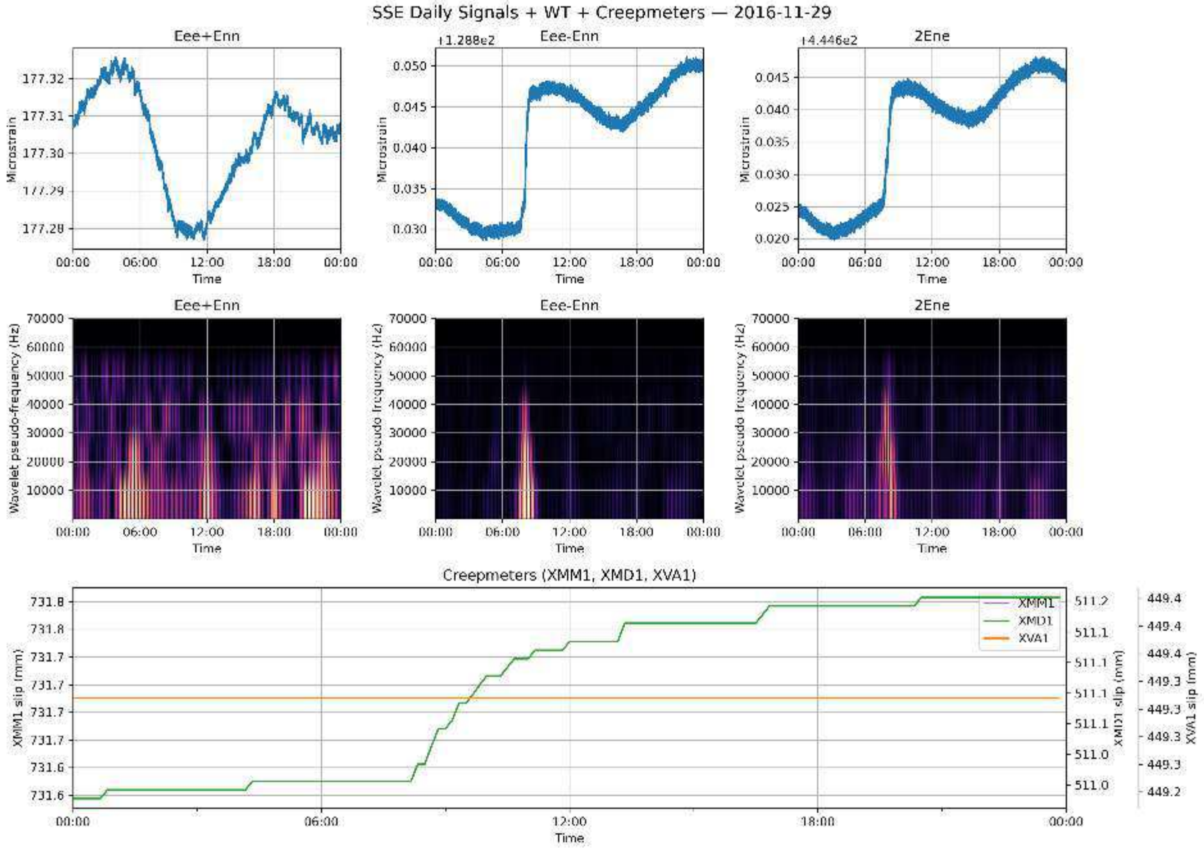
SSE Daily Signals + WT + Creepmeters — 2016-11-29
Eee+Enn
Eee-Enn
2Ene
+1.288e2
+4.446e2
Microstrain
Time
Wavelet pseudo-frequency (Hz)
Creepmeters (XMM1, XMD1, XVA1)
XMM1
XMD1
XVA1
XMM1 slip (mm)
XMD1 slip (mm)
XVA1 slip (mm)

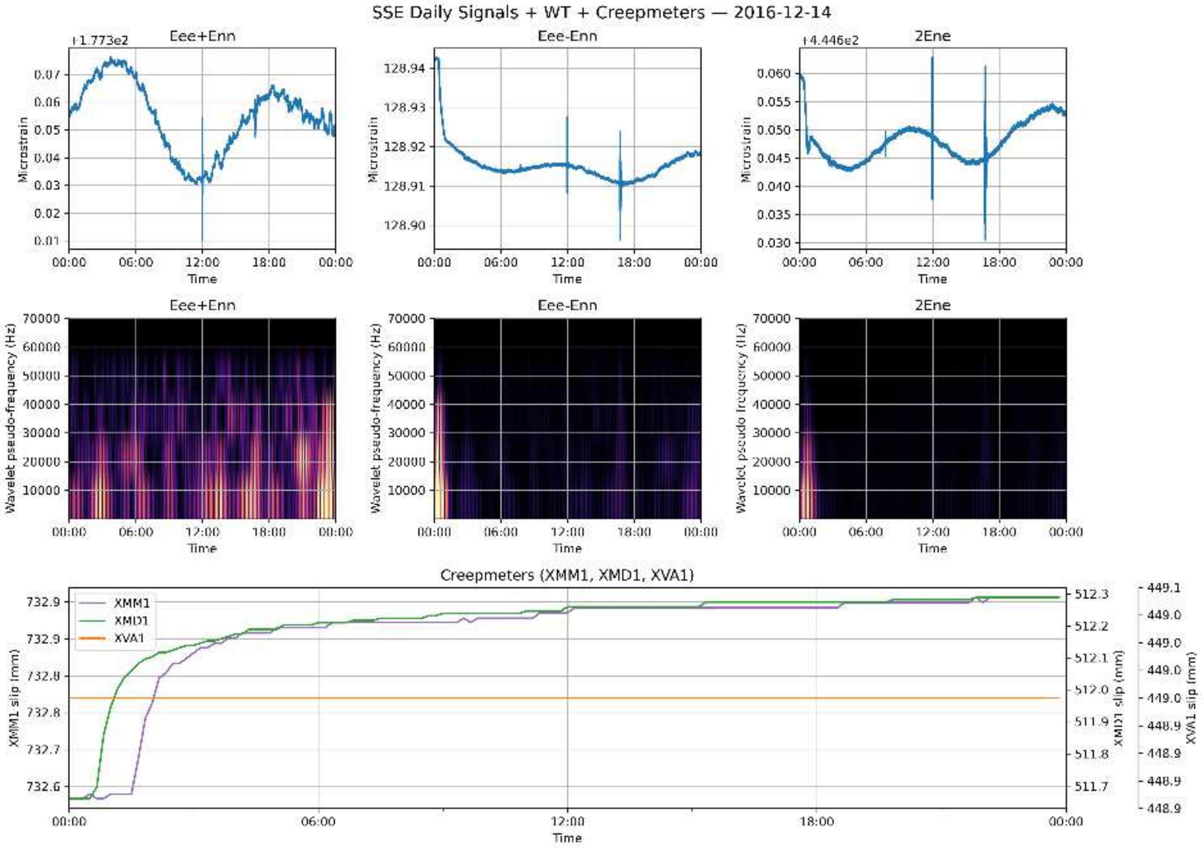
SSE Daily Signals + WT + Creepmeters — 2016-12-14
Eee+Enn
Eee-Enn
2Ene
+1.773e2
+4.446e2
Microstrain
Time
Wavelet pseudo-frequency (Hz)
Creepmeters (XMM1, XMD1, XVA1)
XMM1
XMD1
XVA1
XMM1 slip (mm)
XMD1 slip (mm)
XVA1 slip (mm)
