# Supplementary material for: Slow slip modulates low-frequency seismicity on the Parkfield segment of the San Andreas Fault
Source: Nat Commun. 2026 Jun 9;17:5137. doi: 10.1038/s41467-026-74095-9 (PMC13249816; doi:10.1038/s41467-026-74095-9)
Supplement: Supplementary file 5 — Transparent Peer Review file [file 41467_2026_74095_MOESM5_ESM.pdf]

nature portfolio

Peer Review File

Slow slip modulates low-frequency seismicity on the San Andreas Fault

Corresponding Author: Dr Zahra Zali

This file contains all reviewer reports in order by version, followed by all author rebuttals in order by version.

Parts of this Peer Review File have been redacted as indicated to remove third-party material.

Version 0:

Reviewer comments:

Reviewer #1

(Remarks to the Author)
This is an interesting paper about a timely subject, but in my opinion, requires either further clarifications and/or more work before it is ready for publication. The potentially novel aspects of the analyses and results are the use of strainmeter data to measure aseismic transients with temporal and spatial scales that have previously been challenging to make and possibly providing some observational constraints on linkages between aseismic and seismic fault slip. However, for reasons described below and in the annotated manuscript, I am not convinced that the measurements or the inferences are meaningful. I have attempted to provide some suggestions about how they may be made so.
The authors focus on ‘short duration’ slow slip events (SSEs), but the reason for doing so is not clear. Nothing in the scaling laws, or otherwise, suggests that SSEs or seismic events should have durations only of discrete and particular lengths, so an explanation of why this study focuses on short durations. The machine learning (ML) analysis used relies on wavelets that effectively are tuned to caption signals of “1 second to 40 minutes” (line 371) – one wonders why was this duration chosen? (Also, ‘short’ needs to be defined prior to the 25-100 minutes noted on line 136.)
The introductory discussion on lines 77-88 seems somewhat incomplete, as it seems to neglect the common model in which much smaller seismic events (in terms of moment release) are largely triggered by slow slip, and are proxies for SSEs. Additionally, it neglects the broader literature about SSEs on strike-slip faults. The statement “LFEs and SSEs may reflect a transition from stable, aseismic slip to more localized seismic activity” seems to imply that they occur in spatially distinct regions rather than the commonly invoked model in which LFEs originate from tiny patches embedded withing an otherwise slowly slipping larger fault area. Also, why is this discussion restricted to LFEs, and does include other seismic events that appear to be associated with SSEs, such as tremors, VLFEs, and earthquake swarms? The last sentence seems to suggest that SSEs near the surface appear to be associated with LFEs at depth (beneath the purely seismogenic zone, so 10+ km deeper?), which would be a somewhat novel observation. The observation of the LFEs following the SSEs thus implying the SSE modulates the LFE activity has been observed/inferred numerous times before, which should be noted. Finally, although not explicit, the tone of the introduction implies that observation or inference of SSEs on strike-slip faults is novel and that within California, there is something special about the San Andreas fault. SSEs have been studied on numerous other strike-slip faults in California (see Vavra et al. (2024), Materna et al. (2024), Sirorattanakul et al. (2022), Ross et al. (2020), among others) and there is a broader literature about swarms and SSEs on strike-slip faults generally, particularly transform faults offshore. To put this study in the appropriate context, these others should be noted in the introduction.

The explanation of the machine learning (ML) approach needs to be expanded (lines 94-111). In particular, how does the algorithm distinguish transient signals (SSEs?) from background noise, and SSEs from short term earthquakes and instrumental noise? What characteristics or features of each signal are unique that enable distinguishing between them? What does this feature extraction have to do with clustering (does this refer to spatial or temporal clustering of measurement sites or to features)? The statistical or algorithmic aspects of this are explained a bit more in the Methods section, but not in any conceptual terms that seem essential to understand to judge the appropriateness of the method for the problem being addressed.
Similarly, the discussion of the validation of the approach in the main text also needs improvement. No information is provided about how ‘true’ SSEs are identified by whomever did the visual inspection of the signals, nor is any documentation about the manual catalog available as it apparently is ‘in preparation’. What subjective or objective metrics were used to identify ‘true’ SSEs and distinguish them confidently from signals due to other sources? Data from three strainmeters near the fault were used, one of which is a ‘reference strainmeter’ (what does this mean?), presumably because these three strainmeters were thought most likely to record any SSEs. However, it seems that data from a strainmeter

located at some distance from the fault also should be used, to verify that SSEs were NOT detected at it; this would provide a bit more confidence that detected SSEs are not false events.
Lines 139-149 discuss ML-detected and missed events judged relative to the manual catalog. The first line notes an amplitude change of 0.01 microstrain. This is not very informative information because no context is provided about what the instrumental noise and dynamic range of the strainmeters might be (particularly for the durations targeted), nor of likely environmental noise. This paragraph also mentions seemingly arbitrary decisions about 'smoother transients', false and missed detections (among the false detections, three were not interpreted and 16 dismissed as 'likely due to instrumental noise'), but does not provide any information about what criteria were applied to make these judgements.
Some of the criteria, or rather expectations, of what may be a strain signal indicative of an SSE or of another type of source, are noted in the Supplement. Since the results and inferences drawn from them depend critically on these judgements, addition of a concise summary of discriminating features between SSE and other signals, what metrics were used to assess confidence in consistency with these features (or whether judgements were purely subjective) should be included in the main body of the paper.
The authors state that 14 of the 21 SSEs identified also were detected on the nearby creepmeters, presenting this as evidence that the detections are real SSEs. However, they speculate that those SSEs not visible on the creepmeters may have been deeper than the others, which is plausible but is only speculation. To be convincing, the same analyses should have been done on creepmeters distant from the strainmeters, in order to demonstrate that spatial and temporal coincidence is likely physically linked to the fault beneath the instruments (and not chance or some longer-range coherent noise source). The discussion that follows in lines 169-175, about ML results from the strainmeters as they vary with distance from the fault provides some support, as this based on and conforms to simple physical reasoning that SSE signals should diminish with distance.
In Figure 3c, do the very different shapes of the Ene signals indicate propagation? Why are they so different?
The results shown in Figure 4c are nearly identical to those in Figure 1 of Gomberg et al. (2016), the latter of which implies not a fundamentally different process between 'slow' and 'fast' earthquakes but rather a change in geometry as slip events grow and differing rupture/slip speed and/or stress drops on different faults (the dependence on speed noted in line 212). Why wasn't this alternative mentioned?
The discussion about correlations between LFE activity and SSE occurrence needs more work. The map of Figure 1 shows only a handful of LFE families; were all these used in the analysis (do these families really contain a million LFEs as noted in line 230)? Are there other LFEs from outside the study area in Figure 1b? Figure 5b seems to show that an average of ~13 LFEs/day are observed regardless of the number of days (+-3 days or fewer) from the SSE onset time, and an LFE rate that systematically is greater or lesser by a few LFEs/day if only the period after or before an SSE is considered, respectively? To assess the significance of this, one would like to know what the variability in LFE/day rates is, and if the 'systematic' difference due to just one or two post-SSE intervals within +1 day that has lots more LFEs than the others? Similarly, in Figure 5c, is a normalized cross-correlation of +.03 indicative of a significant correlation (one that would not be achieved by chance, or assumes some ~uniform or other occurrence rate of LFEs)? The significance of the results conveyed in Figure 5 and discussed might be strengthened by showing the results for the other LFE depth ranges, and for the same analysis applied to LFEs from another section of the fault if they exist. If tremor is comprised of overlapping LFEs, a comment on why would there is no correlation between tremor and SSEs would be helpful.

(Remarks on code availability)

Reviewer #2

(Remarks to the Author)
This paper proposes a new catalog of short-duration SSEs in San Andreas Fault with their estimated location and slip directions. To do so, the authors combined a wavelet transform, a (deep learning) auto-encoder and several k-means clustering on strainmeter data from 3 stations. With this new catalog of 75 SSEs between 25min to 1 hour, most of them coherent with a creepmeter, scaling law seems to show a cubic scaling trend, and comparison with shallow LFE events seems to indicate a clear time correlation.

Positive:
The paper is clear and interesting, using a coherent method with a correct validation from creepmeter, and the scientific findings are also of interest even if mostly expected.
The catalog is been released, the code is supposed to be released (but no access for now). Related to the scientific findings, the source analysis is good and the link with LFE is convincing and clear, and it shows that most probably SSEs happen prior to LFE peaks.

Negative:
Related to the method, it seems as if the method could be used off-the-shelf for a new strainmeter, but I think this is misleading as there are many hyperparameters that were tuned here (such as the several k-means), and I don't know how easy they can be tuned when not accessing a creepmeter to compare. There are some other limits in the method, such as the fact that it is a daily detection but not a clear segmentation (picking time). While the code has been set to be released, the access was not granted and the reviewer-only access only gives access to the wavelet transform code. For the source analysis, it is not clear how the assumption of strike-slip only affects the slip directions found in figure 4.b (which are on both directions in both sides of the fault). For the scaling law, an analysis of other durations using the same method (using different wavelet scales?) could help in verifying the cubic assumption.

The paper is thus of interest, and I have listed here the comments that are important:

- the AutoencoderZ model is applied separately to each component --> why? I think it would make more sense to have a 2-channel input since both will be used at the end anyways.
- In your figure, it looks as if your auto-encoder has skip connections, which was not visible in your previous study and is not standard for autoencoders (compared to Unets, their goal is to reduce the dimension). Can you explain this point?
- Is the kmeans performed for all signals or for each station separately? How specific is it, i.e. how easy can your method be transferable to another location? Did you also make the code available in an inference setting (since kmeans partitions the space), at least for these stations?
- I would avoid the word 'classifies' --> it's clustering, not classification, so better use the word 'clusters' to avoid confusions.
- validation: how was the setting and hyperparameters chosen? was the creepmeter used during this stage? did you perform this light supervision on part of the data, and a separate test set was kept aside?
- If I understand correctly, the pipeline detects only whether there is a SSE in one day or not. Can you detect if there are more than one in one day?
- Why are many slips in the opposite direction (fig 4.b)? As I said previously, does your assumption of strike-slip only bias the results?
- Autoencoderz: did you compare with a simpler downscaling, or with more time downsampling bins, or with PCA?
- train/valid: on different days? did you separate the years?
- why did you perform several k-means and not one k-means with more clusters? K-means tends to find clusters of similar sizes, is that an issue here, or could another clustering method be more suited?
- fig5b: if I understand correctly, this figure is made over the 75 SSEs + 75 non-SSEs periods. Why are times before SSEs (-3 to -1 days) with lower SSEs than the non-SSEs?

typos:
remove asimple
areal
K-means or k-means? 'k-means algorithm. K-mean..'
l 264 of an SSE, and on the day and --> remove first 'and'

(Remarks on code availability)
The code here is only a small portion of the full code (the first part, related to the wavelet extraction). The rest is not present. The link in the paper is not available.

Reviewer #3

(Remarks to the Author)
This article discusses a well-identified general issue: transient slips at all time scales. The originality of the work lies in the use of strainmeters to detect brief, relatively superficial small slow slip events in the vicinity of the sensors.
The subject is therefore interesting. It seems to me that the article suffers from a number of inaccuracies and a lack of information to be fully convincing. I list these points below.

Reference is made to an unpublished manual catalogue (paper in prep) and there is no link to the GFZ data center.

lines 150–155. Why not show a comparison of your catalogue with available data including regular earthquakes, local and distant large events, and may be SSEs (likely deeper) confirmed by the GNSS network by Rousset et al. 2019 (see below).

You refer to Rousset (2019), which is not relevant to the discussion, but there is indeed a paper by Rousset et al. 2019 that discusses SSEs in the Parkfield region. To be corrected.

There is inconsistent use of the term 'frequency' in Figure S13. In the same figure, it seems to me from experience that the advantage of the wavelet transform is exaggerated. It should be shown that the time scales concerned are indeed the same in (b) and (c).

The validation of detections as SSEs is highly subjective. In particular, there are seven low amplitude-long duration events that are excluded a priori, even though they would have a strong influence on Figure 4d for the discussion of scaling. This should be clarified, and all detections should be presented.

line 177. Good idea, why not do it? It seems easy to achieve without changing your flow of processsing.

It seems to me that one weakness of the article lies in the issue of the resolution of the parameters for the location and size of events. It would be important to discuss the resolution/errors of the values discussed in the last part of the paper. A first step would be to show the misfits as a function of the parameters. Without these plots, it is not possible to be convinced of your assertions about depth, which is generally very poorly resolved with small numbers of static observations, as is the case here. From my perspective as a reader, this is a critical element that is missing.

Next, in line 443, you claim that you estimate the length of the slipping patches. This seems doubtful to me for reasons of resolution. Finally, why base the scaling analysis on a manual assessment of duration? Do you observe a relationship between length and duration?

(Remarks on code availability)

Version 1:

Reviewer comments:

Reviewer #1

(Remarks to the Author)
The manuscript is much improved from the original draft. I have included my questions and suggestions in comments in the annotated text of the revised manuscript that I am submitting as part of my review. Overall, the study and manuscript are largely focused on the observations and measurements derived from them, with secondary emphasis on the physical mechanisms that underlie them. They add to the growing constraints on our understanding of how faults slip and thus are useful contributions. The topic is timely, manuscript is well written and interesting.

(Remarks on code availability)
See my comments in the annotated manuscript returned.

Reviewer #2

(Remarks to the Author)
The authors have answered most of my comments, thank you.
- ok for the code
- ok for the source analysis and strike-slip assumption
- ok for the reason of the duration of events studied

I am in favor of publishing after few small further comments (minor review):
- validation and hyperparameters:
1) Ok I understand better now how was used the k-means. A new k-means needs to be fitted for every station and every strain component (why?), and a manual selection is done in order to select the SSE-like signals cluster in each. Does some of the kmeans clustering fall in local minima that is not desired?
2) I think that sometimes it is not clear what hyperparameter tuning mean. Selecting a workflow, an algorithm, over others, and how to organize them (in your case, having 2 k-means clusterings, with 2 k each, with a specific distance metric, etc.) is also part of the hyperparameter tuning. And I guess these choices were made based on the 20% validation set. This could be considered as a light overfitting - you select the workflow that works for that specific case, but we don't know how it performs to new samples. This is even more true since samples were randomized, so training and validation samples are likely very similar (contiguous in time). Nevertheless, since the model itself is unsupervised, I am fine with publishing this without a rigorous separated test set, but it could be added at the end that a further testing on later years/new regions would be needed to fully assess the workflow.
- AutoencoderZ: this is maybe not in the scope of this particular paper, but your answer on the new model, AutoencoderZ, having skip connections is not convincing. Indeed, I understand that with skip connections the reconstruction is better - this why we use them in Unets, to keep more details. But for feature encoding and auto-encoders, since only the features at the coarser scale are kept as encoding, if we add skip connections then it means that some information is 'by-passed' between the finer scales without being encoded in the coarse scale. So the encoding is likely to be less informative, at least on the highest frequencies...

(Remarks on code availability)

Reviewer #3

(Remarks to the Author)
In this new version, the misfits are represented as I had suggested. The reason for this request was the doubts that readers might have about the actual resolution of the values of the slip locations and amplitudes discussed in the paper.
The figures presented seem to confirm these doubts. While the determination of a parameter by inversion is effective if the misfit as a function of the parameter does indeed exhibit convexity around a minimum, it does not appear that this condition is satisfied in this study according to Figures S10 to S12. How can locations or depths be considered resolved under these conditions? Even for slip, there is no minimum in the range of parameters for most cases.
The misfits of the depths presented show characteristics that I find difficult to understand, with very rapid transitions from regimes where the rates of variation with depth are very large to others where they are zero at the precision of the representation. This casts doubt on the figures themselves.

The authors need to be able to justify their results more convincingly. As it stands, the basis for the discussion and conclusions is not established on actual observations.
There are still problems with the figures (see Frequency ranges in Supp. Fig 4 to 7).

(Remarks on code availability)

Version 2:

Reviewer comments:

Reviewer #3

(Remarks to the Author)
I note some more careful comments on the poor resolution of locations. The maps are still a bit misleading with the representation of the events by simple points. But at least a careful reader can be aware of the issue.

The authors have added a comment that I find a bit odd (lines 393–394):
As noted by Ide and Beroza (2023), the cubic scaling in ordinary earthquakes is governed by the modulation of elastodynamic interactions, which won't apply in the type of quasi-static deformation represented by SSEs.

What is "the modulation of elastodynamic interactions" ??
Of course there is no available duration for a static process, and the fundamental cubic scaling is with crack length (e.g. Eshelby, 1957). If the medium is elastic, This property should probably be preserved regardless of the process, as even dynamic stress-drop models confirm this.
The problem with slow-slip events is mainly due to the low resolution of depth data, which prevents an adequate assessment of the size of the sliding surface; this issue has been resolved for 'fast' earthquakes by assuming a rupture velocity of the same order of magnitude as that of shear waves for all earthquakes. There is no equivalent of such a constant-velocity assumption for slow-slip processes, but the physical scaling of moment versus length is likely still valid.
The new sentence in the text should be corrected.

(Remarks on code availability)

Open Access This Peer Review File is licensed under a Creative Commons Attribution 4.0 International License, which permits use, sharing, adaptation, distribution and reproduction in any medium or format, as long as you give appropriate credit to the original author(s) and the source, provide a link to the Creative Commons license, and indicate if changes were made.
In cases where reviewers are anonymous, credit should be given to 'Anonymous Referee' and the source.

The images or other third party material in this Peer Review File are included in the article's Creative Commons license, unless indicated otherwise in a credit line to the material. If material is not included in the article's Creative Commons license and your intended use is not permitted by statutory regulation or exceeds the permitted use, you will need to obtain permission directly from the copyright holder.
To view a copy of this license, visit https://creativecommons.org/licenses/by/4.0/

Editor:

We have now received reports from 3 reviewers and, after careful consideration, we have decided to invite a major revision of the manuscript.

As you will see from the reports copied below, the reviewers raise important concerns. We find that these concerns limit the strength of the study, and therefore we ask you to address them with additional work. Without substantial revisions, we will be unlikely to send the paper back to review. In particular, they are concerned about the validation of the approach and the criteria for the identification of SSEs as well as the comparison/validation of the findings with other data.

Dear Editor,

Thank you for your feedback and for forwarding the reviewers' reports. We appreciate the careful evaluation of our manuscript. We have undertaken substantial revisions to address all of the concerns raised by the three reviewers. In particular, we have:

- Expanded our methodology to explicitly investigate the possible existence of longer-duration slow slip events in the Parkfield strainmeter data using extended wavelet scales and complementary analyses. This required additional processing, validation, and the inclusion of new supplementary figures and notes.

- Clarified and strengthened the validation of the detection approach, including explicit criteria for SSE identification and their relation to the published manual catalog and independent observations.

- Expanded the methodological description to better explain the machine-learning workflow, feature extraction, clustering strategy, and their limitations.

- Performed additional analyses and robustness tests, including extended statistical evaluations and supplementary figures and tables, to support the comparison with independent datasets (LFEs, creepmeters, and tremor).

- Revised the Introduction and Discussion to better situate the study within the broader literature and to clarify the scope and limitations of our conclusions.

A detailed, point-by-point response to all reviewer comments is provided in the accompanying response letter, with our responses indicated in blue. All corresponding changes have been incorporated into the revised manuscript and Supplementary Material.

We believe that these revisions substantially strengthen the manuscript and directly address the concerns raised. We appreciate the reviewers' constructive feedback and hope that the revised version will now be suitable for consideration and publication.

Sincerely,
Dr. Zali and co-authors

REVIEWER COMMENTS

Reviewer #1 (Remarks to the Author)

This is an interesting paper about a timely subject, but in my opinion, requires either further clarifications and/or more work before it is ready for publication. The potentially novel aspects of the analyses and results are the use of strainmeter data to measure aseismic transients with temporal and spatial scales that have previously been challenging to make and possibly providing some observational constraints on linkages between aseismic and seismic fault slip. However, for reasons described below and in the annotated manuscript, **I am not convinced that the measurements or the inferences are meaningful.** I have attempted to provide some suggestions about how they may be made so.

We thank the reviewer for the critical and constructive review of our manuscript, which has greatly contributed to clarify concepts and expand our method.

C1: **The authors focus on 'short duration' slow slip events (SSEs), but the reason for doing so is not clear.** Nothing in the scaling laws, or otherwise, suggests that SSEs or seismic events should have durations only of discrete and particular lengths, so an explanation of why this study focuses on short durations. The machine learning (ML) analysis used relies on wavelets that effectively are tuned to caption signals of "1 second to 40 minutes" (line 371) – one wonders why was this duration chosen? (Also, 'short' needs to be defined prior to the 25-100 minutes noted on line 136.)

Reply: We appreciate the reviewer's comment and have substantially clarified the motivation for studying the short-duration (~25–100 min) SSEs in both the manuscript and the Supplementary Material. Our focus is based on three independent considerations:

1. Instrumental detectability limits of borehole strainmeters:

The short-duration transients in our catalog fall within the portion of the spectrum where borehole strainmeters have the highest stability and where the wavelet transform (WT) produces clear, localized signatures. Strainmeters excel in the time range between hours and weeks, and they can exhibit increasing noise and lower signal detectability toward longer periods. Even after applying standard corrections (tidal loading, barometric pressure, long-term trends), borehole strain records retain substantial low-frequency variability arising from environmental loading, borehole relaxation, hydrologic effects, and instrumental drift (Johnston et al., 1990; Gladwin et al., 1987; Hodgkinson et al., 2013). These long-period effects limit the reliable detection of small-amplitude, longer-duration transient strain signals.
This instrumental sensitivity trade-off is illustrated in Fig. 1 (from Reuveni et al 2012, figure 3b in that paper), which shows that strain-based estimates have markedly lower noise power at sub-daily periods, whereas GPS approaches comparable performance at day-scale and longer periods. Figure 2 also shows the rate sensitivity of borehole strainmeters, which excel at resolving deformation at periods from minutes to days, whereas GPS provides superior performance primarily at longer periods (e.g., Langbein, USGS; see schematic rate-sensitivity comparison).
Nevertheless, we acknowledge the reviewer's point and, motivated by this comment, expanded our analysis to explicitly search for longer-duration transients using extended wavelet scales. The results of this investigation; the results are presented in the following section and Supplementary Note 1.

REDACTED

Figure 1. Power spectral density (PSD) ratios of strain measurements normalized by the laser strainmeter (LSM). Shown are standard GPS processing (blue), GPS with improved tropospheric and sidereal corrections (red), and a borehole strainmeter (BSM; black). Lower PSD ratios indicate lower noise and higher strain sensitivity. Borehole strainmeters exhibit the lowest noise at sub-daily periods, corresponding to the frequency band targeted in this study for detecting short-duration SSEs. The figure is from Reuveni et al 2012, figure 3b in that paper.

REDACTED

Figure 2. Schematic comparison of rate sensitivity as a function of period for different geodetic instruments. Borehole strainmeters exhibit superior sensitivity at short to intermediate periods (minutes to a few days), whereas GPS measurements become increasingly effective at longer periods. The shaded region highlights the time scales where strainmeters are best suited for resolving transient deformation, consistent with the focus on short-duration slow slip events in this study. The figure is from Langbein, USGS.

2. Absence of longer-duration tectonic transients in the Parkfield strainmeter data

We now additionally examined our Parkfield strainmeter records for possible longer transients to address the concern of the reviewer.

In addition to the main analysis (WT scales 1–2048; duration sensitivity ~1 s to ~150 min), we performed an additional WT analyses using:

scales 4000–10000 → sensitivity to transients up to ~15 hours

Employing this setting, we did not find evidence of longer-duration tectonic transients embedded in the strainmeter data. These checks, together with examples from the signal and WT at the extended scale, as well as transient example in longer duration which is not SSE, are now documented in Supplementary Note 1.

For even longer-duration slow slips, such as events lasting days to several months, we did not apply our automatic detection algorithm. Instead, we visually inspected the continuous strainmeter records for each year (plots provided in Supplementary Note 1). These year-long plots show no abrupt or systematic strain changes indicative of day-long, multi-day, or multi-week tectonic transients in the Parkfield data. To clarify how such longer-duration SSEs would appear if present, we include here an example of a multi-day SSE from the San Juan Bautista region. We show it here to demonstrate that multi-day SSEs, when they contain sufficient amplitude, can be identified visually in strain records, whereas short-duration SSEs of the type examined in our study require a dedicated detection algorithm. The following figure is from our unpublished study, and we are using it only for the review process.

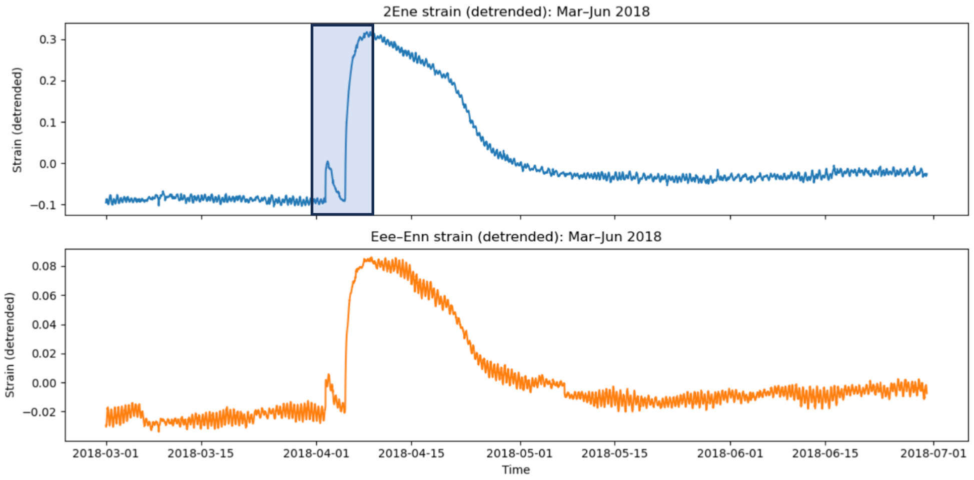

Figure 3. One SSE with a duration of some days that is easily detectable through a visual check of the strainmeter data plotted over a 4-month period.

3. The duration of the signals included in reference catalog and used for validation

Our detection framework is validated against a manually constructed catalog compiled by David Mencin (documented in his publicly available PhD thesis and in preparation for a publication. We now refer to the published thesis for clarity). All events in this reference catalog have duration ranging between 25 and 100 minutes.
The WT configuration was tuned to the duration range of catalogued events. Although the largest WT scale used in the main analysis corresponds to periods of ~40–45 minutes, step-like

transients with longer duration (up to ~120–150 minutes) still produce detectable WT energy because their onset contains a finite slope and curvature that project energy into the 10–40 minute band. We added a detailed explanation of this behavior in Supplementary Note 2, supported by synthetic examples (Supplementary Note Fig 3).
We also revised the "Wavelet Transform for Event Representation" subsection in Method in the manuscript to explicitly describe how the chosen WT scale range governs the detectable event durations (lines: 489 to 502 annotated manuscript).

C2: The introductory discussion on lines 77-88 seems somewhat incomplete, as it seems to neglect the common model in which much smaller seismic events (in terms of moment release) are largely triggered by slow slip, and are proxies for SSEs.
Additionally, it neglects the broader literature about SSEs on strike-slip faults. The statement "LFEs and SSEs may reflect a transition from stable, aseismic slip to more localized seismic activity" seems to imply that they occur in spatially distinct regions rather than the commonly invoked model in which LFEs originate from tiny patches embedded withing an otherwise slowly slipping larger fault area.
Also, why is this discussion restricted to LFEs, and does include other seismic events that appear to be associated with SSEs, such as tremors, VLFEs, and earthquake swarms?
The last sentence seems to suggest that SSEs near the surface appear to be associated with LFEs at depth (beneath the purely seismogenic zone, so 10+ km deeper?), which would be a somewhat novel observation. The observation of the LFEs following the SSEs thus implying the SSE modulates the LFE activity has been observed/inferred numerous times before, which should be noted. Finally, although not explicit, the tone of the introduction implies that observation or inference of SSEs on strike-slip faults is novel and that within California, there is something special about the San Andreas fault.
SSEs have been studied on numerous other strike-slip faults in California (see Vavra et al. (2024), Materna et al. (2024), Sirorattanakul et al. (2022), Ross et al. (2020), among others) and there is a broader literature about swarms and SSEs on strike-slip faults generally, particularly transform faults offshore. To put this study in the appropriate context, these others should be noted in the introduction.

Reply: We thank the reviewer for this thoughtful set of comments. We have revised the introduction substantially to address each of the points raised regarding (1) the physical interpretation of LFEs and their relationship to SSEs, (2) the broader context of SSEs and aseismic transients on strike-slip faults, (3) the inclusion of other seismic observables commonly associated with slow slip, and (4) clarification of our wording regarding depth and potential causal interpretations. Below we summarize the changes made.

1. In response to the reviewer's comment that the introduction did not mention the widely recognized model in which small seismic events are triggered by slow slip, we added the following text: "*In many regions, small seismic events, including LFEs, are viewed as indirect indicators of slow aseismic slip, because stress changes associated with gradual fault slip can promote brittle failure on small asperities embedded within the slipping region (Peng & Gomberg, 2010).*"

2. To avoid the unintended implication that LFEs and SSEs occur in spatially distinct zones, we rewrote the relevant sentences to show the commonly accepted model in which LFEs originate from small patches embedded within a broader, slowly slipping fault zone. The revised text now reads: "*The temporal correlation between LFEs and SSEs may arise from localized brittle failure within a broader region undergoing slow aseismic slip, as LFEs are*

generally interpreted to originate from small patches embedded within a larger, slowly slipping fault area."

3. To address the reviewer's concern that our discussion focused too narrowly on LFEs, we modified the opening sentence to include the broader suite of seismic manifestations commonly accompanying slow slip: "*Seismic manifestations of ongoing slow processes such as SSE commonly include tremor, very-low-frequency earthquakes, earthquake swarms, and low-frequency earthquakes (LFEs), all of which may occur in response to aseismic stress transients.*"

4. We clarified our intended meaning regarding the temporal relationship between SSEs and LFEs. The revised text states: "*In our study, we show that low-frequency earthquakes (LFEs) near Parkfield tend to follow the detected SSEs, suggesting a potential causal relationship between slow aseismic slip and subsequent LFE activity. This behavior aligns with earlier observations in both subduction and strike-slip tectonic regimes, where variations in LFE activity have been shown to track the evolution of slow slip transients*."

5. We added new sentences and citations to reflect the substantial existing work on SSEs and aseismic transients along strike-slip faults, as recommended by the reviewer:"*SSEs have been studied on several strike-slip faults in California (e.g., Materna et al., 2024; Ross et al., 2020).*"

and later:

"*…and similar relationships between aseismic slip transients and seismic events have also been documented on several strike-slip faults in California (Lohman & McGuire, 2007; Materna et al., 2024; Ross et al., 2020; Sirorattanakul et al., 2022; Vavra et al., 2024).*"

C3: The explanation of the machine learning (ML) approach needs to be expanded (lines 94-111). In particular, how does the algorithm distinguish transient signals (SSEs?) from background noise, and SSEs from short term earthquakes and instrumental noise? What characteristics or features of each signal are unique that enable distinguishing between them? What does this feature extraction have to do with clustering (does this refer to spatial or temporal clustering of measurement sites or to features)? The statistical or algorithmic aspects of this are explained a bit more in the Methods section, but not in any conceptual terms that seem essential to understand to judge the appropriateness of the method for the problem being addressed.

Reply: We have now expanded the description of our machine-learning workflow to clarify how transient deformation is distinguished from background noise, short-lived seismic signals, and instrumental noises. We now explain that the wavelet transform highlights short-duration, step-like strain changes by producing a characteristic broadband WT signature, which forms the basis for separating SSEs from other signals.

We also clarified the feature-extraction part. AutoencoderZ operates in a fully unsupervised manner and automatically learns a compact latent representation of each day's WT. These latent features capture the salient structure of the data, enabling each day to be represented by only 24 features instead of the full high-dimensional WT. Clustering is then performed in this low-dimensional space: the first stage identifies days containing any transient strain change, and the second isolates short-duration SSEs from other transient sources such as local

earthquakes and instrumental noise. After clustering and automatic detection, we visually inspect the detected events to remove any remaining non-tectonic or unwanted signals.

These clarifications have been incorporated into the revised manuscript (Lines 116 to 141 in annotated manuscript).

C4: Similarly, the discussion of the validation of the approach in the main text also needs improvement. No information is provided about how 'true' SSEs are identified by whomever did the visual inspection of the signals, nor is any documentation about the manual catalog available as it apparently is 'in preparation'. What subjective or objective metrics were used to identify 'true' SSEs and distinguish them confidently from signals due to other sources?

Reply: We thank the reviewer for raising this point. The manual catalog is not unpublished; it is fully documented in Mencin's publicly accessible PhD thesis (2018), which we now cite explicitly in the revised manuscript.

As described in Appendix A of that thesis, "true" SSE events were identified by visually inspecting the differential and engineering strain channels for characteristic short-duration, step-like strain transients, using 4-hour windows. When available, concurrent observations from the three nearby creepmeters were used as supporting evidence to confirm that a strain transient corresponded to shallow aseismic slip rather than noise, local earthquakes, or instrumental artifacts.

We have added a concise description of these criteria to the Methods section (Line 408 to 414 in annotated manuscript) and now explicitly reference the published thesis where the catalog is documented. Also, we are now publishing the waveforms and WT figures of all the SSE reported in our study as supplementary material. Supplementary Data 1 includes an Excel file with the complete SSE catalog and associated modeling information, and Supplementary Data 2 contains the waveform and WT figures for all SSEs.

C5: Data from three strainmeters near the fault were used, one of which is a 'reference strainmeter' (what does this mean?), presumably because these three strainmeters were thought most likely to record any SSEs. However, it seems that data from a strainmeter located at some distance from the fault also should be used, to verify that SSEs were NOT detected at it; this would provide a bit more confidence that detected SSEs are not false events.

Reply: We appreciate the reviewer's comment. Indeed, we had in mind a similar logic than what the reviewer describes. In this study, analyzed data from the three strainmeters (B073, B075, B076) located within a few kilometers of the creeping section of the San Andreas Fault because this is the only part of the network where strainmeter spacing is sufficiently dense to provide redundant observations of small, shallow slip transients, as also noted in Mencin (2018). B073, which we refer to in the manuscript as the "reference strainmeter," is simply the closest instrument to the fault and therefore records the clearest signals, and it was the strainmeter that was utilized to derive the manual catalog. Once we tuned our methodology to detect the signals in B073, we tested if they could also be detected at strainmeters at a slightly larger distance from the fault, that is B075 and B076. Indeed, the signals recorded in B075 and B076 show progressively smaller amplitudes which resemble the increasing distance from the fault. This amplitude decay is described in the Methods section, illustrated in Figure 3, and discussed in the main text. We have now added a brief clarification in the manuscript explaining why B073 is designated as the reference strainmeter.

After these three strainmeters, the next nearest strainmeter to the San Andreas Fault main trace is ~17 km away from B073 (see Methods section first paragraph). Given the very small amplitudes of many of the short-duration SSEs (often near the detection threshold even at B075 and B076), signals of this size would not be expected to be observable at such a distance. For this reason, the absence of a signal on a far-field strainmeter would not provide a meaningful test of event validity and would not strengthen the interpretation. Our validation therefore relies instead on redundancy within the local 3-stations, which also show increasing distance from the fault and where amplitude patterns are consistent with shallow slip, and on supporting observations from the nearby creepmeters when available.

C6: Lines 139-149 discuss ML-detected and missed events judged relative to the manual catalog. The first line notes an amplitude change of 0.01 microstrain. This is not very informative information because no context is provided about what the instrumental noise and dynamic range of the strainmeters might be (particularly for the durations targeted), nor of likely environmental noise. This paragraph also mentions seemingly arbitrary decisions about 'smoother transients', false and missed detections (among the false detections, three were not interpreted and 16 dismissed as 'likely due to instrumental noise'), but does not provide any information about what criteria were applied to make these judgements. Some of the criteria, or rather expectations, of what may be a strain signal indicative of an SSE or of another type of source, are noted in the Supplement. Since the results and inferences drawn from them depend critically on these judgements, addition of a concise summary of discriminating features between SSE and other signals, what metrics were used to assess confidence in consistency with these features (or whether judgements were purely subjective) should be included in the main body of the paper.

Reply: Thank you for this comment. We agree that additional clarification of the criteria used to distinguish SSEs from noise, local earthquakes, and instrumental artifacts was necessary and we have now refined our descriptions with that goal in mind..
The absolute dynamic range of Plate Boundary Observatory (PBO) borehole strainmeters is large (digitizer resolution ~10^{-10} strain and measurable signals up to at least 10^{-5} strain; Barbour & Agnew, 2011), so amplitudes of 0.01 μstrain (10^{-8} strain) are within instrumental capability. However, the detectability of transients is limited by increasing low-frequency noise. Barbour & Agnew (2011) show that strain noise rises steeply at frequencies below ~0.001 Hz (periods >1000 s), which overlaps the 25–100-minute durations of our SSEs. We have added this context to clarify why some small events are challenging to detect automatically (Lines 185-192 in annotated manuscript).
We have now also added a concise description of the criteria used to distinguish SSEs from other transient signals, consistent with the methodology documented in Mencin (2018). For each candidate event, we examined whether the signal exhibited a step-like strain change over tens of minutes, with the largest amplitudes in the differential and engineering components compared to the areal component. We also inspected the individual gauge records, as tectonic deformation produces coherent patterns across multiple gauges, whereas instrumental artifacts typically affect only one. When available, we additionally used supporting offsets on nearby creepmeters. We also compared the transient shape with the strain patterns expected from shallow dislocation models (e.g., Fig. 6 in Mencin 2018) to qualitatively assess consistency with aseismic fault slip. These criteria are now summarized explicitly in the main text (Lins 170 to 179). We also added Supplementary Fig. 4, which illustrates the characteristic short-duration step-like jumps and provides further explanation about those that produced 16 false detections and are very likely instrumental in origin. As shown in Supplementary Fig. 4, these

signals are non-tectonic based on their identical waveform shape and duration; all 16 reported cases exhibit the same characteristic pattern.

C7: The authors state that 14 of the 21 SSEs identified also were detected on the nearby creepmeters, presenting this as evidence that the detections are real SSEs. However, they speculate that those SSEs not visible on the creepmeters may have been deeper than the others, which is plausible but is only speculation. To be convincing, the same analyses should have been done on creepmeters distant from the strainmeters, in order to demonstrate that spatial and temporal coincidence is likely physically linked to the fault beneath the instruments (and not chance or some longer-range coherent noise source). The discussion that follows in lines 169-175, about ML results from the strainmeters as they vary with distance from the fault provides some support, as this based on and conforms to simple physical reasoning that SSE signals should diminish with distance.

Reply: We thank the reviewer for this comment. In our study we used the three closest creepmeters to the strainmeters (XVA1, XMD1, and XMM1) as shown in Fig. 1. These instruments span the same section of the San Andreas Fault as strainmeters B073, B075, and B076, and therefore provide the most direct and physically relevant comparison for slip beneath this part of the fault. As also illustrated in the supplementary figures 5-7, which shows the 3 creepmeters signals, slip associated with short-duration SSEs is typically detectable only on the creepmeter closest to the slipping patch, consistent with the expected rapid spatial decay of SSE signals. More distant creepmeters lie well outside the region where deformation from these small-amplitude events would be measurable. However, to address the reviewer's concern that the observed signals don't reflect broader-scale or non-tectonic noise sources, we extended our validation by examining creepmeters located farther from the strainmeter network. Specifically, we analyzed data from two additional instruments selected based on data availability and temporal resolution: CRR1, located ~15 km from the reference strainmeter stations, and XMR1, located ~96 km away. These distances allow us to test whether signals coincident with the detected SSEs appear regionally or persist far beyond the fault segment under study. For both creepmeters, we find no slip or transient signals coincident with the times of the strainmeter-detected SSEs. We illustrate this with example (Figure 4) time windows for two events: one from the manual catalog and one newly detected SSE, showing that neither CRR1 nor XMR1 exhibits slip during these intervals. This absence of coincident signals at more distant creepmeters argues against a regional or coherent non-tectonic noise source and supports the interpretation that the observed transients are localized fault slip beneath the strainmeter array.

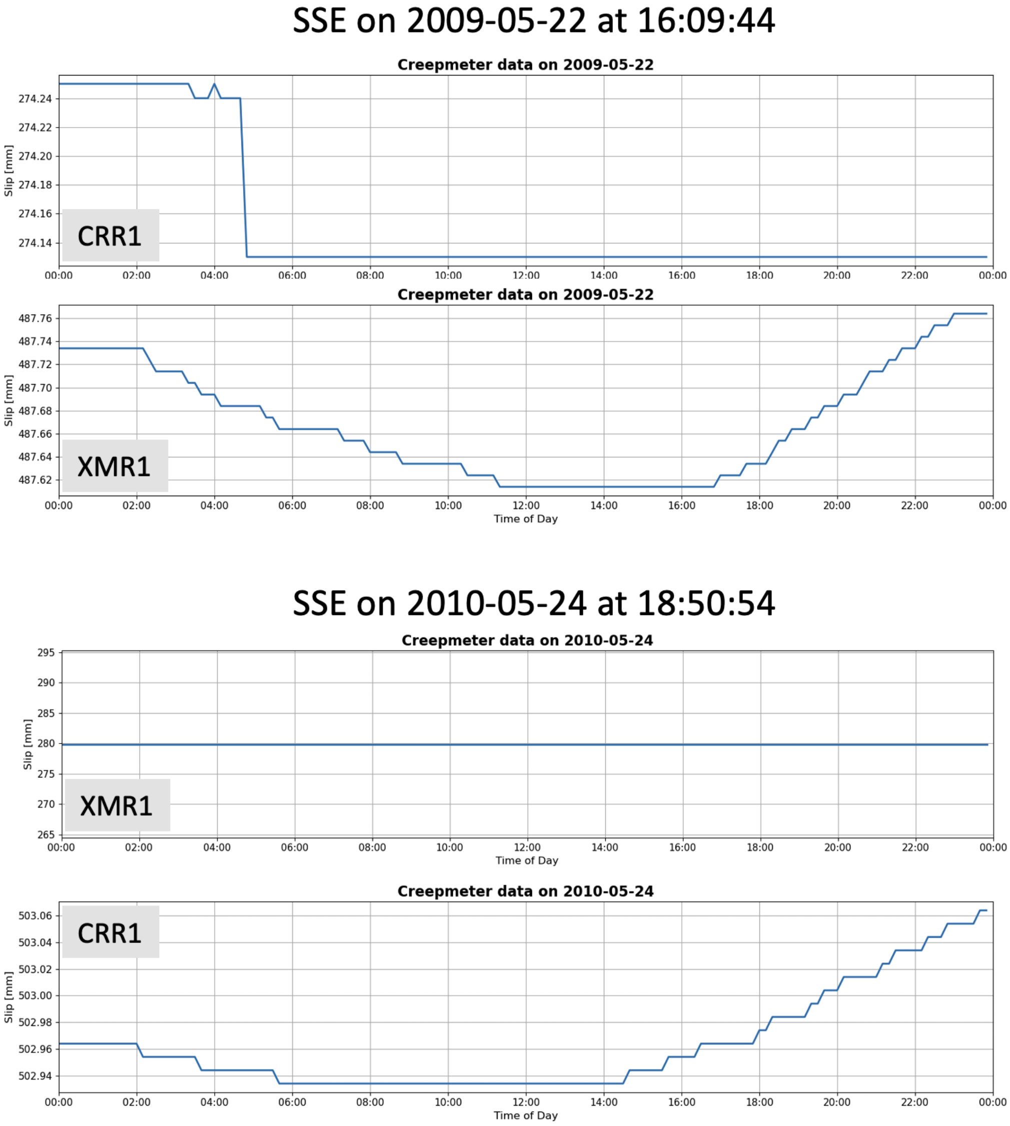

Figure 3. Examples of creepmeter observations at stations CRR1 (~15 km from the strainmeter B073) and XMR1 (~96 km away) during two SSEs detected by the strainmeters in our study. The top panels show creepmeter data on 22 May 2009 for an SSE occurring at 16:09:44 (from manual catalog), and the bottom panels show creepmeter data on 24 May 2010 for an SSE occurring at 18:50:54 (from newly detected SSE). No slip or transient signal is observed at either creepmeter coincident with the SSE times, indicating that the strainmeter-detected events are not associated with regional or coherent non-tectonic deformation affecting distant instruments. These examples support the interpretation that the observed SSEs are localized fault slip events beneath the strainmeter array.

C8: In Figure 3c, do the very different shapes of the Ene signals indicate propagation? Why are they so different?

Reply: In short, yes, it is likely that the different shape of the 2Ene signals indicate propagation. The occurrence of a slow slip event can be modeled for example as the slip of a crack of a given length described with a dislocation model (Okada, 1985). The resulting strain field is different depending on the location of the slow slip event (e.g. with depth) and it also can

strongly vary at different receiver positions. For a detailed view to understand the modelling of slow slip signals, we refer the reviewer to David Mencin's PhD Thesis (2018).

C9: The results shown in Figure 4c are nearly identical to those in Figure 1 of Gomberg et al. (2016), the latter of which implies not a fundamentally different process between 'slow' and 'fast' earthquakes but rather a change in geometry as slip events grow and differing rupture/slip speed and/or stress drops on different faults (the dependence on speed noted in line 212). Why wasn't this alternative mentioned?

Reply: We appreciate the reviewer's comment. Gomberg et al. (2016) showed that the global M_0–T scaling for slow and fast slip can be explained by a geometric transition from unbounded to bounded rupture growth on large subduction interfaces. However, this interpretation applies to slip events spanning many orders of magnitude in moment and duration, particularly in subduction zones. In contrast, our study focuses on short-duration, shallow slow slip events on the creeping section of the San Andreas Fault with moments far below the scale at which the bounded-unbounded transition becomes relevant. Our Figure 4 is not intended to interpret global scaling behavior but simply places our estimates within previously reported scaling relationships for SSEs. For this reason, and because Gomberg et al.'s geometric framework is not applicable to the Parkfield SSEs considered here, we do not include that discussion in the main text.

C10: The discussion about correlations between LFE activity and SSE occurrence needs more work. The map of Figure 1 shows only a handful of LFE families; were all these used in the analysis (do these families really contain a million LFEs as noted in line 230)? Are there other LFEs from outside the study area in Figure 1b?

Reply: Thank you for raising this point. The LFE catalog used in our analysis corresponds only to the 2009–2016 period of our study and contains more than 500,000 LFEs during this interval. The full Northern California catalog includes approximately one million LFEs over 2001–2016, but we use only the subset occurring within our study period. We have clarified this in the revised manuscript (Line 304 in the annotated manuscript). For a complete map of the spatial distribution of all LFE families in the full catalog, we refer the reviewer to Fig. 1 of Shelly (2016).

As described in the manuscript, our analysis proceeded in two steps. First, we tested the correlation between SSEs and the full set of LFEs occurring during 2009–2016. This broader comparison shows no meaningful temporal association (Supplementary Fig. S13a). Motivated by the small amplitudes and shallow depths of the short-duration SSEs here, we then restricted the analysis to LFEs occurring within 10 km of the strainmeters. Only within this spatial window does a temporal correlation emerge. We also evaluated the dependence on LFE depth: using LFEs across the full depth range (Fig. S13b), LFEs shallower than 20 km (Fig. 5a), and LFEs deeper than 20 km (Fig. S13c). These tests show that the observed correlation arises from LFEs within 10 km laterally and at depths <20 km, whereas deeper or more distant LFEs show no correlation. In the 10 km distance and depth shallower than <20 km, there are about 15000 LFE, and we added this information to the manuscript (Line 328 in the annotated manuscript).

C11: Figure 5b seems to show that an average of ~13 LFEs/day are observed regardless of the number of days (+-3 days or fewer) from the SSE onset time, and an LFE rate that systematically is greater or lesser by a few LFEs/day if only the period after or before an SSE is considered, respectively? To assess the significance of this, one would like to know what the

variability in LFE/day rates is, and if the 'systematic' difference due to just one or two post-SSE intervals within +1 day that has lots more LFEs than the others?

Reply: We thank the reviewer for this comment. We have expanded the analysis of LFE variability and updated Fig. 5b accordingly. In the original submission we computed LFE/day only for days that contained at least one LFE. We now recompute all statistics using all calendar days from 2009–2016, assigning zero LFEs to days without any LFE. The updated Fig. 5b reflects this correction. Although absolute values shift slightly, the pattern remains the same: LFE activity systematically increases following an SSE.

To quantify variability, we report the distribution of daily LFE counts over the entire 8-year period:
Total days: 2922
Mean LFE/day: 5.57
Standard deviation: 13.93
Interquartile range (IQR): 4.0 LFEs/day

We also computed LFE/day statistics for ±3 days around each SSE. The results are:

SSE −3 days: mean = 3.91, std = 8.98, IQR = 3.00

SSE −2 days: mean = 4.71, std = 9.58, IQR = 4.00

SSE −1 days: mean = 4.54, std = 8.78, IQR = 5.00

SSE day: mean = 8.09, std = 20.90, IQR = 4.00

SSE +1 days: mean = 8.77, std = 23.14, IQR = 6.00

SSE +2 days: mean = 7.15, std = 15.35, IQR = 5.00

We also computed per-SSE before/after differences:
Mean LFE/day before SSE: 4.39 (std = 7.17)
Mean LFE/day after SSE: 7.86 (std = 16.87)
Mean increase (after − before): 3.47 LFEs/day (std = 16.57)

Thus, although individual SSEs vary widely, the ensemble average shows a consistent increase in LFE activity following SSEs.

To evaluate whether this pattern could be dominated by one or two extreme post-SSE days, we repeated the calculation using median daily LFE counts, which are robust to outliers. The median-based results (now shown in Supplementary FigS. 16b) show the same qualitative behavior: median LFE rates are higher after SSEs than before them. We also include a complementary plot using only LFE-active days for comparison (Supplementary FigS. 16a), which again shows the same trend. We added this information to the manuscript and the table (Supplementary Table 1) with all the variability measures (mean, standard deviation, and interquartile range) of LFE/day rates.

These additions strengthen the conclusion that the observed increase in LFE activity following SSEs is not driven by a small number of outlier days but reflects a systematic pattern across the SSE catalog.

C12: Similarly, in Figure 5c, is a normalized cross-correlation of +.03 indicative of a significant correlation (one that would not be achieved by chance, or assumes some ~uniform or other occurrence rate of LFEs)?

Reply: The reviewer asks whether a normalized cross-correlation value of +0.03 is significant. In this case, the absolute magnitude of the coefficient is not the meaningful diagnostic, because the two time series being correlated differ strongly in their statistical properties: (1) the SSE time series is extremely sparse, with only 92 SSE days over 2922 days (≈3% non-zero entries), and (2) the LFE time series is highly variable, ranging from zero to 157 LFEs per day. When one time series contains very few non-zero entries, the normalized cross-correlation is mathematically constrained to remain small even when the timing between the two processes is non-random. In such cases, the shape of the cross-correlation curve and the lag of the maximum provide much more reliable information than the magnitude of the coefficient itself. The key observation in our analysis is therefore the peak near SSE + 1 day, which indicates that LFE activity tends to increase immediately after SSE onset. To clarify this point, we now explicitly state in the manuscript that the interpretation is based on the relative enhancement and timing of the peak, rather than on the absolute value of the normalized coefficient (Line 345 to 350 in the annotated manuscript).

C13: The significance of the results conveyed in Figure 5 and discussed might be strengthened by showing the results for the other LFE depth ranges, and for the same analysis applied to LFEs from another section of the fault if they exist.

Reply: We thank the reviewer for this suggestion. We have already performed the depth-dependent and region-dependent LFE analyses raised in this comment. Specifically, we examined LFE activity for (i) the full depth range of the catalog, (ii) LFEs deeper than 20 km, and (iii) LFEs shallower than 20 km. These results are shown in Supplementary Fig. 13b–c and discussed in the text. Only the shallow (<20 km) LFEs within 10 km of station B073 show a temporal increase following SSEs (Figure 5a); deeper events and LFEs at larger distances do not exhibit a systematic correlation. We also tested whether LFEs from a broader section of the fault show any correlation. Supplementary Fig. 13a shows the analysis using the full LFE catalog (covering a much larger portion of the fault system). In this case, no temporal association with SSEs is observed. Together, these depth- and region-dependent tests strengthen the conclusion that the observed correlation is specific to shallow LFEs located near the strainmeter and does not arise when considering LFEs from outside this zone.

C14: If tremor is comprised of overlapping LFEs, a comment on why would there is no correlation between tremor and SSEs would be helpful.

Reply: We appreciate the reviewer's question regarding tremor. Although tremor is known to consist of overlapping LFEs in some subduction environments, this relationship does not necessarily exist on the Parkfield segment. We already tested for a temporal association between tremor activity (using the publicly available catalog) and the SSEs identified in this work (Supplementary Figure 15), but we did not observe a relationship.

There are several plausible explanations, but we cannot determine the exact cause with the available data. One possibility is that the tremor-generating zone beneath Parkfield lies deeper than the shallow slip patches that produce the short-duration SSEs, reducing the sensitivity of tremor to small stress perturbations. The mean depth of tremors during our study period is approximately 22 km, with reported depths extending to about 93 km. Another possibility is that tremor in this region is more strongly modulated by long-term loading rather than by the

relatively small, localized stress changes associated with our shallow SSEs. Because of these uncertainties, we prefer to refrain from speculating about this in the paper.

Reviewer #2 (Remarks to the Author):

This paper proposes a new catalog of short-duration SSEs in San Andreas Fault with their estimated location and slip directions. To do so, the authors combined a wavelet transform, a (deep learning) auto-encoder and several k-means clustering on strainmeter data from 3 stations. With this new catalog of 75 SSEs between 25min to 1 hour, most of them coherent with a creepmeter, scaling law seems to show a cubic scaling trend, and comparison with shallow LFE events seems to indicate a clear time correlation.

Positive:
The paper is clear and interesting, using a coherent method with a correct validation from creepmeter, and the scientific findings are also of interest even if mostly expected. The catalog is been released, the code is supposed to be released (but no access for now). Related to the scientific findings, the source analysis is good and the link with LFE is convincing and clear, and it shows that most probably SSEs happen prior to LFE peaks.

Negative:
C15: Related to the method, it seems as if the method could be used off-the-shelf for a new strainmeter, but I think this is misleading as there are many hyperparameters that were tuned here (such as the several k-means), and I don't know how easy they can be tuned when not accessing a creepmeter to compare. There are some other limits in the method, such as the fact that it is a daily detection but not a clear segmentation (picking time). While the code has been set to be released, the access was not granted and the reviewer-only access only gives access to the wavelet transform code.

Reply: We thank the reviewer for these points. In the following, we clarify several aspects of the method here.

First, the code is now publicly accessible via the GitHub repository named DeepStrain as below:

https://github.com/ZahraZali/DeepStrain

Regarding tuning and general applicability, the method does not rely on creepmeter data for parameter tuning. Creepmeters were used only for independent validation of detected events, not to train or adjust the machine-learning model. The automated detection itself is entirely unsupervised. As explained earlier to the first reviewer, for each candidate event from the unsupervised detection method, we examined whether the signal exhibited a step-like strain change over tens of minutes, with the largest amplitudes in the differential and engineering components compared to the areal component. We also inspected the individual gauge records, as tectonic deformation produces coherent patterns across multiple gauges, whereas instrumental artifacts typically affect only one. When available, we additionally used supporting offsets on nearby creepmeters. We also compared the transient shape with the strain patterns expected from shallow dislocation models (e.g., Fig. 6 in Mencin 2018) to qualitatively assess consistency with aseismic fault slip. These criteria are now summarized explicitly in the main text. Considering all these validation criteria, our machine-learning method effectively detects true SSEs with a low rate of false detections, as described in the manuscript.

AutoencoderZ is a fixed architecture developed and validated in our previous studies (Zali et al., 2024; Zali et al., 2025) across different tectonic settings (volcanic and tectonic) and signal

durations. Its hyperparameters are not tuned for this specific dataset. The model is used solely for feature extraction and dimensionality reduction, and has been shown to generalize well across data types. The subsequent k-means clustering also does not involve tuning. We simply set the number of clusters as 2 and perform the k-means clustering 2 times to first separate noise from transients and then isolate SSE-like signals.

We agree that the method currently performs daily-scale detection rather than precise temporal segmentation. This choice was deliberate: over the 8-year period analyzed, only 92 SSEs were detected, and daily detection enables efficient screening of large datasets. Once candidate days are identified, start and end times can be determined by visual inspection based on waveform shape. Developing fully automated onset/offset picking using shorter wavelet windows and scale optimization could be a direction for future work.

Overall, our goal here is to introduce a first fully unsupervised framework for detecting small-amplitude, short-duration SSEs in continuous strainmeter data, optimized for rapid analysis of large datasets.

C16: For the source analysis, it is not clear how the assumption of strike-slip only affects the slip directions found in figure 4.b (which are on both directions in both sides of the fault).

Reply: We thank the reviewer for this comment. In our source analysis, we constrain the fault geometry to the San Andreas Fault and assume a pure strike-slip mechanism, which fixes the direction of motion to be parallel to the fault plane but does not prescribe whether slip is right- or left-lateral. The sense of slip is determined by the inversion and depends on the observed strain amplitudes and their signs.

The inferred slip directions shown in Fig. 4b indicate that the majority of events, and in particular those with larger slip magnitudes, are consistent with right-lateral motion along the fault. Some smaller events show inconsistency. This opposing slip directions on either side of the fault likely arise from the limited spatial coverage provided by only three strainmeters, which reduces azimuthal resolution and can introduce ambiguity in strain-based source inversions. This limitation associated with sparse station geometry has been noted previously (e.g., Gladwin et al., 1994).

To avoid ambiguity, we have revised the manuscript text to explicitly state that a strike-slip mechanism was assumed, while the sense of slip is inferred from the inversion. We also updated Fig. 4b to include a legend for the slip-vector magnitude. In the revised figure, arrow lengths scale linearly with slip magnitude to provide a more physically intuitive representation. In an earlier version, a square-root scaling was used purely for visualization, which exaggerated the relative length of small-magnitude vectors. The updated linear scaling preserves the true relative amplitudes of slip.

C17: For the scaling law, an analysis of other durations using the same method (using different wavelet scales?) could help in verifying the cubic assumption.

Reply: We thank the reviewer for this comment. We emphasize that all SSEs observable at the three strainmeter stations are included in the scaling-law analysis. This analysis does not rely on events detected by our automated algorithm; instead, it includes all SSEs that can be clearly identified by visual inspection at all three stations, since the Okada dislocation model requires strain amplitude changes at at least three sites. Events missed by the automated detection are included if they are observable in a detailed visual inspection. In practice, two of the seven

events missed by the automated method are included in the scaling analysis. While lower-amplitude and longer duration transients are generally more challenging to detect automatically, this is a relative limitation. The durations of the seven missed events in the automatic detection method (reported in the manuscript) remain below approximately 4 hours, consistent with the range reported by Mencin (2018), and fall within the 25–100 minute duration range considered in this study. We clarified this in the manuscript by adding the following sentence: *"For the location analysis, we include all SSEs from the manual catalog, regardless of whether they were detected by our machine learning method, together with newly detected SSEs, and retain only those that are coherently observable at all three stations; events observable at only one or two stations are excluded, although all manual and newly detected SSEs have comparable durations in the 25–100 min range."*

Regarding the existence of longer-duration SSEs beyond the available manual catalog, in this version of the manuscript we have already addressed this point in our response to reviewer # 1. Please see our reply to comment C1 from reviewer #1 in this report. That response consists of three parts; in the second part, titled *"2. Absence of longer-duration tectonic transients in the Parkfield strainmeter data"*, we present a new analysis specifically designed to search for longer-duration SSEs and show that no such signals are identified in the data.

The paper is thus of interest, and I have listed here the comments that are important:

C18: - the AutoencoderZ model is applied separately to each component --> why? I think it would make more sense to have a 2-channel input since both will be used at the end anyways.

Reply: We thank the reviewer for this suggestion. Using a multi-channel (joint) input is a reasonable idea and could be explored in future work. In the present study, however, the AutoencoderZ model is applied separately to the differential and engineering strain components because, although SSEs produce broadband energy in the wavelet domain in both components, the time-domain signal shapes differ between them (see Fig. 3). Treating the components independently allows the model to learn component-specific representations of transient behavior. However, we agree that testing a joint multi-channel model to evaluate whether it yields equivalent clustering results would be a valuable extension of this work and we are already testing some configurations in this respect, although they need substantial additional further work.

C19: - In your figure, it looks as if your auto-encoder has skip connections, which was not visible in your previous study and is not standard for autoencoders (compared to Unets, their goal is to reduce the dimension). Can you explain this point?

Reply: We thank the reviewer for this question. U-Nets can be viewed as a specific class of autoencoders that incorporate skip connections between corresponding encoder and decoder layers to preserve high-resolution spatial information, particularly for image segmentation tasks. More generally, skip connections are used in autoencoder architectures to retain information that may otherwise be lost at the bottleneck and to improve reconstruction quality and training stability. In our earlier work (Zali et al., 2024), we designed an autoencoder without skip connections. In a subsequent study (Zali et al., 2025), we improved our model by adding skip connections between encoder and decoder layers and named the updated architecture as AutoencoderZ. These skip connections allow the decoder to access intermediate representations that help preserve detailed signal features. As shown in Zali et al. (2025), this

modification improves reconstruction and robustness across different data types, tectonic settings, and signal durations.

C20: - Is the kmeans performed for all signals or for each station separately? How specific is it, i.e. how easy can your method be transferable to another location? Did you also make the code available in an inference setting (since kmeans partitions the space), at least for these stations?

Reply: We thank the reviewer for this comment. K-means clustering is applied separately for each station and each strain component, after feature extraction with AutoencoderZ. The method is fully unsupervised and does not rely on predefined labels or fixed classifiers. All code required for feature extraction and clustering is now publicly available on GitHub, and all input data used in this study are public, allowing the method to be reproduced and applied to other stations or regions by re-running the unsupervised workflow. For performing the model in another location, there is no tuning needed, and it can be trained as it is with the new data.

C21: - I would avoid the word 'classifies' --> it's clustering, not classification, so better use the word 'clusters' to avoid confusions.

Reply: Thanks for the comment. We modified the word.

C22: - validation: how was the setting and hyperparameters chosen? was the creepmeter used during this stage? did you perform this light supervision on part of the data, and a separate test set was kept aside?

Reply: We thank the reviewer for this question. Creepmeters were not used as part of the machine-learning model or during training. The AutoencoderZ model is trained in a fully unsupervised manner using only strainmeter data. After the automated detection step, the manual SSE catalog and nearby creepmeters were used only for post validation, to assess whether detected events correspond to known SSEs and whether surface slip was observed at the same time. This validation step does not influence the learned features or clustering.

During training of the autoencoder, the strainmeter dataset was split into 80% for training and 20% for validation, following standard practice to monitor reconstruction performance and avoid overfitting. No supervised labels or external observations were used in either stage. We have now clarified this procedure in the Methods section (Line 407 to 414 in the annotated manuscript).

C23: - If I understand correctly, the pipeline detects only whether there is a SSE in one day or not. Can you detect if there are more than one in one day?

Reply: The method currently performs daily-scale detection rather than precise temporal segmentation (see detailed response to the comment C15 in this review). This choice was deliberate: over the 8-year period analyzed, only 92 SSEs were detected, and daily detection enables efficient screening of large datasets. Once candidate days are identified, start and end times can be determined by visual inspection based on waveform shape. So if there is more than one SSE in one day, we will detect all when we visually check the detected days resulting from the automated detection process. In our analysis, there are a couple of days with 2 SSEs, which we detected in this way.

C24: - Why are many slips in the opposite direction (fig 4.b)? As I said previously, does your assumption of strike-slip only bias the results?

Reply: Thank you for the comment. We refer to the reply to the comment C16.

C25: - Autoencoderz: did you compare with a simpler downscaling, or with more time downsampling bins, or with PCA?

Reply: We did not perform a direct comparison with simpler dimensionality-reduction approaches (e.g., PCA) within the scope of this study. However, previous studies, including our previous work (Zali et al., 2024), show that autoencoders can capture the nonlinear structure of transient signals more effectively compared to traditional linear methods such as PCA. Based on those results, we adopted AutoencoderZ here as a robust and well-tested feature-extraction framework rather than re-evaluating alternative methods.

C26: - train/valid: on different days? did you separate the years?

Reply: The autoencoder was trained using an 80% / 20% train–validation split after randomly shuffling the daily WT samples. The split was performed across all available days from the full 8-year period (and not separately for each year) as explained in the manuscript.

C27: - why did you perform several k-means and not one k-means with more clusters? K-means tends to find clusters of similar sizes, is that an issue here, or could another clustering method be more suited?

Reply: We tested several clustering strategies, including applying a single k-means step with a larger number of clusters, as well as other clustering methods. We found that a two-stage k-means approach provided the most robust separation of signal types for this application. In the first stage, using two clusters allows the algorithm to efficiently separate days dominated by background variability from days containing any transient signal, which is the primary distinction of interest at this step. Applying clustering again only to the transient subset in the second stage, then separates transients with different waveform and wavelet characteristics, allowing SSEs to form a distinct cluster. When using a larger number of clusters in a single step, k-means tends to partition background-noise days into multiple groups, which does not aid SSE identification and can reduce the clarity of the separation. The staged approach therefore focuses the clustering on progressively more relevant structure in the data. Although k-means favors clusters of comparable size, we did not find this to be a limiting factor here.

C28: - fig5b: if I understand correctly, this figure is made over the 75 SSEs + 75 non-SSEs periods. Why are times before SSEs (-3 to -1 days) with lower SSEs than the non-SSEs?

Reply: Indeed, Fig. 5b shows that LFE rates during the days immediately preceding SSEs (−3 to −1 days) are slightly lower than the average rate during non-SSE periods. This is an empirical observation emerging from the data. At present, we do not have an explanation for this behavior and we are not sure how significant it is. Our main result focuses on the consistent increase in LFE activity during and after SSEs and we would prefer to refrain from interpreting that behavior at this stage.

typos:
C29: remove asimple Thanks, we corrected it.

C30: areal This is the areal component of the strainmeter.

C31: K-means or k-means? 'k-means algorithm. K-mean..' Thanks, we corrected it (K-means).

C32: l 264 of an SSE, and on the day and --> remove first 'and' Thanks, we corrected it.

Reviewer #2 (Remarks on code availability):
C33: The code here is only a small portion of the full code (the first part, related to the wavelet extraction). The rest is not present. The link in the paper is not available.

Now the full code is publicly available on GitHub.

Reviewer #3 (Remarks to the Author):

This article discusses a well-identified general issue: transient slips at all time scales. The originality of the work lies in the use of strainmeters to detect brief, relatively superficial small slow slip events in the vicinity of the sensors.

The subject is therefore interesting. It seems to me that the article suffers from a number of inaccuracies and a lack of information to be fully convincing. I list these points below.

C34: Reference is made to an unpublished manual catalogue (paper in prep) and there is no link to the GFZ data center.

Reply: Our detection framework is validated against a manually constructed catalog compiled by David Mencin (documented in his PhD thesis and in preparation for a publication). Now we refer to the publicly published PhD thesis as Mencin (2018). Our catalog, which underpins the present study and includes both the events reported in the Mencin thesis and our newly detected SSEs, is provided as supplementary material and will be published with the manuscript. The catalog is supplied as an Excel file (Supplementary Data 1), together with a separate supplementary file containing the waveform and wavelet transform figures for all SSEs (Supplementary Data 2).

C35: lines 150–155. Why not show a comparison of your catalogue with available data including regular earthquakes, local and distant large events, and may be SSEs (likely deeper) confirmed by the GNSS network by Rousset et al. 2019 (see below).
You refer to Rousset (2019), which is not relevant to the discussion, but there is indeed a paper by Rousset et al. 2019 that discusses SSEs in the Parkfield region. To be corrected.

Reply: Thank you for this comment. We indeed intended to refer to Rousset et al. (2019) and have corrected the citation accordingly.

We would like to clarify, however, that a direct event-by-event comparison between our SSE catalog and the results of Rousset et al. (2019) is not feasible, due to fundamental methodological differences and data availability. Rousset et al. (2019) do not detect individual slow slip events directly from geodetic or strainmeter observations. Instead, their analysis identifies SSEs signal by stacking GPS time series using the timing of low-frequency earthquake (LFE) bursts from the Shelly (2017) catalog as temporal guides. The "SSE times" in that study are therefore inferred from LFE activity, rather than independently observed slow slip events. Individual SSE origin times, durations, or a reusable event catalog are not published; the timing information is only shown graphically (e.g., Fig. 2c in Rousset et al., 2019) and represents approximate central times of LFE bursts.

Importantly, Rousset et al. (2019) explicitly report that they could not detect these SSEs in borehole strainmeter data, stating:
"*We also searched for the transient signals detected by GPS in NW Parkfield in strainmeter time series, but given the noise level at periods of ~10 days, we did not succeed in significantly extracting the signal due to the slow slips (Fig. S5)" (text from Rousset et al., 2019).*

In contrast, the SSEs identified in our study are directly detected from borehole strainmeter data, with well-defined onset times and short durations (25–100 minutes), and are independently validated across multiple strainmeters and nearby creepmeters. Because Rousset

et al. (2019) do not provide an explicit SSE catalog or precise event times, a direct comparison with our strainmeter-based SSE catalog is not possible.

Nevertheless, we do compare our SSE catalog with the same underlying LFE catalog (Shelly, 2017) used by Rousset et al. (2019). This allows us to discuss similarities and differences in the temporal relationship between slow slip and LFEs. We have clarified this distinction in the manuscript (Lune 317 to 324).

We also did the comparison with the earthquake and tremor catalog. As already stated in the manuscript (line 329 to 333 in the annotated manuscript) : "*We also compared the cumulative number of SSEs with that of earthquakes (Fig. S14) and tremors (Fig. S15) at different distances from the reference strainmeter in the central San Andreas Fault region (Fig. S10). No correlation is observed between SSEs and tremors, while a possible weak correlation appears for earthquakes, though it is much less clear than the correlation with LFEs.*"

C36: There is inconsistent use of the term 'frequency' in Figure S13. In the same figure, it seems to me from experience that the advantage of the wavelet transform is exaggerated. It should be shown that the time scales concerned are indeed the same in (b) and (c).

Reply: We thank the reviewer for pointing this out. We agree that the term "frequency" can be ambiguous when referring to wavelet representations. In the revised figure and caption, we now explicitly distinguish between Fourier frequency (STFT) and wavelet pseudo-frequency (scale). The wavelet pseudo-frequencies shown in Fig. S19c (Fig. S13 in the submitted version) are converted from wavelet scales using the Morlet wavelet relation and correspond to the same range of periods as the STFT frequencies in Fig. S19b.

The time axis and temporal window are identical in panels (b) and (c), and both representations are computed from the same 1-Hz sampled strain data. Because of the sampling rate, the STFT is limited to frequencies below 0.5 Hz, whereas the wavelet transform emphasizes time-localized energy at comparable low-frequency periods. The advantage of the wavelet transform highlighted here is therefore not due to different time or frequency ranges, but to its improved time localization of transient, non-stationary signals such as short-duration SSEs.

We have clarified this point in the caption to avoid confusion and to ensure that the comparison between STFT and wavelet representations is made on equivalent time scales.

C37: The validation of detections as SSEs is highly subjective. In particular, there are seven low amplitude-long duration events that are excluded a priori, even though they would have a strong influence on Figure 4d for the discussion of scaling. This should be clarified, and all detections should be presented.

Reply: We thank the reviewer for this comment and agree that clarification was needed. The seven "missed events" discussed in the manuscript are mentioned exclusively in the context of the limitations of the automated detection workflow, not as events that were excluded from subsequent analyses. These seven events were not detected by the automated method because they exhibit relatively small amplitudes (~0.01 μstrain) and smoother strain evolutions, which make them more difficult to distinguish from background variations in the wavelet domain. This discussion is to explain detection performance.

Importantly, no SSEs were excluded from the scaling analysis on the basis of amplitude or duration. For the source location and moment magnitude analysis, only events observable on

all three strainmeters are used, as this is required for performing the Okada dislocation analysis. For transparency, we now provide the corrected strainmeter signals, their corresponding wavelet transform (WT) representations, and the traces of the three nearby creepmeters for all events in the catalog in Supplementary Data 2. The complete SSE catalog is provided as an Excel file in Supplementary Data 1. The characteristics and waveform shapes of the seven missed events can be directly inspected in the figures in Supplementary Data 2; these missed events are related to dates: 2009-05-22, 2012-01-03, 2012-06-10, 2012-10-30, 2014-01-09, 2014-10-18, and 2015-12-13.

Although we note that lower-amplitude, smoother transients are more challenging to detect automatically, this is a relative statement. The durations of the seven missed events still remain below ~4 hours, consistent with the range reported by Mencin (2018), and they lie within the 25–100 minute range considered in this study. As stated in the scaling analysis section, "*to investigate the physical characteristics of the short-term SSEs, we analyze 22 events coherently observed across all three strainmeters (B073, B075, and B076).*" This selection is not related to detection limitations but is based solely on the requirement of coherent observations at three stations. The manual catalog is created based on a single station, and not all events are observable at all three strainmeters (also incomplete data availability across the 8-year period on the second and third stations). Source location and moment estimation are not feasible for events observed at only one or two stations. However, this does not affect the scaling results shown in Fig. 4d, because the durations of all 92 events fall within the same range as the events analyzed here (25–100 minutes) and would therefore not alter the inferred scaling relationship. In fact, among the 22 events used for the source and scaling analysis, two events (SSE on 2012-06-10 and 2015-12-13) belong to the group of seven events missed by the automated detection, and their inclusion does not change the scaling relation. We clarified this in the manuscript by adding the following sentence: *"For the location analysis, we include all SSEs from the manual catalog, regardless of whether they were detected by our machine learning method, together with newly detected SSEs, and retain only those that are coherently observable at all three stations; events observable at only one or two stations are excluded, although all manual and newly detected SSEs have comparable durations in the 25–100 min range."*

C38: line 177. Good idea, why not do it? It seems easy to achieve without changing your flow of processsing.

Reply: While masking known earthquakes using external catalogs is technically feasible, it is not straightforward to implement without introducing additional assumptions. In particular, SSEs and earthquakes may be temporally correlated, and removing seismic events a priori could inadvertently cut strain transients associated with or triggered by SSEs. Moreover, such masking would require subjective choices regarding magnitude thresholds, distance cutoffs, and time windows, which may vary between regions and datasets. We now explicitly add this to the manuscript: "*However, this would require careful assessment to avoid excluding strain transients that may be temporally correlated with SSEs and to define appropriate magnitude and distance thresholds.*". In this study, our primary goal is to develop and demonstrate an unsupervised, data-driven detection framework for SSEs. Given the relatively small number of false detections attributed to earthquakes, we opted to retain a simple processing workflow and address these cases through manual inspection. We agree that systematic integration of earthquake catalogs and transient suppression strategies is an important avenue for future improvement.

C39: It seems to me that one weakness of the article lies in the issue of the resolution of the

parameters for the location and size of events. It would be important to discuss the resolution/errors of the values discussed in the last part of the paper. A first step would be to show the misfits as a function of the parameters. Without these plots, it is not possible to be convinced of your assertions about depth, which is generally very poorly resolved with small numbers of static observations, as is the case here. From my perspective as a reader, this is a critical element that is missing.

Reply: We thank the reviewer for this important comment. We agree that resolution and parameter uncertainty are critical aspects of the source analysis, particularly given the limited number of stations. To address this concern, we have now included misfit surfaces for inverted parameters (horizontal location x–y, depth, and slip) for each event, which are presented in the Supplementary Material (Figs. S10–S12). These plots show the misfit variation as a function of each parameter and allow direct assessment of parameter resolution.

The misfit curves indicate that slip amplitude is generally well constrained, whereas depth and horizontal location are more weakly resolved. In particular, depth misfit curves typically exhibit a broad minimum at shallow depths (<4 km), beyond which the misfit becomes relatively flat. This behavior indicates that while the data favor shallow sources, depth is not sharply resolved, consistent with the use of only three strainmeters. Similarly, x–y misfit maps show minimum near the barycenter of the strainmeters, with no improvement in misfit at larger distances. This supports the stability of the inferred source locations while highlighting limited spatial resolution due to sparse station geometry. We have clarified this limitation explicitly in the manuscript by adding the following notes (Line 269 to 274 in the annotated manuscript): *"Misfit analyses (Figs. S10–S12) indicate that slip amplitudes are generally better constrained than source depth and horizontal location. Depth misfit curves typically exhibit broad minima at shallow depths, beyond which misfit varies weakly, reflecting limited depth resolution with only three strainmeters. Accordingly, the inferred shallow depths should be interpreted as preferred solutions rather than tightly constrained estimates."* and now include all misfit plots to provide transparency regarding parameter resolution.

C40: Next, in line 443, you claim that you estimate the length of the slipping patches. This seems doubtful to me for reasons of resolution. Finally, why base the scaling analysis on a manual assessment of duration? Do you observe a relationship between length and duration?

Reply: We agree that resolving the detailed geometry of the slipping patches is challenging with the limited spatial coverage of three strainmeters. In our analysis, fault length is not independently or robustly resolved in the same sense as slip magnitude or moment. Length is explored within a prescribed range during the grid search, but the misfit surfaces show that length is generally weakly constrained. For this reason, our interpretations focus primarily on slip magnitude, moment, and duration, which are better resolved by the data. We have revised the manuscript by adding this (Line 605 to 608 in the annotated manuscript) *"While fault length is explored during the grid search, the limited three-station geometry provides only weak constraints on source dimensions. Slip and seismic moment are better resolved than fault length or depth, and we therefore focus the scaling analysis on moment–duration relationships."* to clarify that fault length estimates should be regarded as approximate and model-dependent rather than tightly constrained parameters.
Given the limited resolution of length we do not interpret length–duration scaling in this study. Instead, the scaling analysis focuses on moment–duration relations, which are more robustly supported by the observations.

To address the reviewer’s concern explicitly, we now include supplementary figures (Fig. S10-12) showing misfit variations with respect to slip, depth, and horizontal location. These figures demonstrate that slip (and therefore moment) is generally better constrained than geometric parameters such as depth and length.

Regarding the duration, we base the scaling analysis on manually assessed durations because SSEs are characterized by gradual strain transients without sharp onsets or offsets, making automated picking difficult to apply in a robust and consistent manner. After the automated detection algorithm identifies days containing SSEs, the start and end times of each event are determined by manual visual inspection of the strain records.

REVIEWER COMMENTS

Reviewer #1:

The manuscript is much improved from the original draft. I have included my questions and suggestions in comments in the annotated text of the revised manuscript that I am submitting as part of my review. Overall, the study and manuscript are largely focused on the observations and measurements derived from them, with secondary emphasis on the physical mechanisms that underlie them. They add to the growing constraints on our understanding of how faults slip and thus are useful contributions. The topic is timely, manuscript is well written and interesting.

Reply: Thank you for this positive and encouraging assessment of our revised manuscript. We appreciate the reviewer's careful reading and thoughtful comments, as well as the acknowledgement that the revisions have improved the clarity and strength of the study. We provide detailed responses to the specific comments below.

C1: Why are these off the SAF?

Reply: The low-frequency earthquake (LFE) locations shown in Fig. 1b are not determined in this study but are taken from the published catalog of Shelly (2017). While not entirely sure about the most plausible hypothesis, one explanation is that the fault has a slight dip. These LFEs occur on the deeper portion of the San Andreas Fault (~16–30 km depth) and therefore may appear laterally offset from the mapped surface fault trace in map view. As described in Shelly (2017), individual LFEs are assigned to family centroid locations with typical uncertainties of ~1–2 km, and their apparent scatter also reflects this assignment and visualization choices.

C2: This is not in the reference list! Where and how to get this pub does not seem to be mentioned anywhere! Since this Mencin work is a major anchor of this study, it seems critical to make it accessible! The response to the reviewers says it is now 'published' – but where?!

Reply: As described in our previous response, the SSE catalog compiled by D. Mencin is documented in his publicly available PhD thesis and is also in preparation for a journal

publication. We apologize for not including this reference previously in the original reference list. We have now added the thesis to the reference list. The thesis is openly available and can be downloaded at:
Mencin, D. (2018). Periodic and Static Strain Investigations with Borehole Strainmeters and GPS. PhD thesis, University of Colorado Boulder.
https://scholar.colorado.edu/concern/graduate_thesis_or_dissertations/xd07gs87r

C3: At a glance, the signals in Fig. S1 don't look hugely different than those in the positive detections. Also, in all the figures, since it is the strain change that is important and to make it easier to compare signals, please plot the y-axes showing the strain CHANGEs, relative to some zero or mean strain value – so that the reader doesn't have to do this in his/her head!

Reply: As already described in the caption of Fig. S1 and in the manuscript (line 163), this example is classified as a false detection because its strain component behavior is inconsistent with typical SSE signatures. Although a transient is visible in the wavelet transform, the areal strain component (Eee + Enn) shows a substantially larger amplitude than the differential and engineering components, whereas true SSEs are characterized by the largest amplitudes appearing in the differential and engineering strain components relative to the areal component. This pattern suggests a non-tectonic origin for the signals shown in Fig. S1.
Thank you for your suggestion regarding the figures. The plotted time series show calibrated strain components after standard corrections and pre-processing. We prefer to retain the real strain values and keep the current plotting style for consistency across all figures through the text.

C4: If this likely instrumental artifact was removed the remaining strains look much like those inferred to be positive, so it's unclear why this one (and maybe others) is deemed 'false'?

Reply: As described in the manuscript and in the caption of Supplementary Fig. S4, these signals are identified as false detections because they exhibit an abrupt, step-like jump followed by a rapid return to baseline, which is inconsistent with the gradual, ramp-like strain evolution expected for SSEs. We did not remove these artifacts from the records; they are shown in their original form. Importantly, these artifacts occur only within a limited portion of the 2016 record, with a fixed duration, further supporting their instrumental origin rather than a tectonic source.

C5: The duration of the strain changes in those in Figs. S5 and S6 are nearly as short as that dismissed as an instrumental artifact and false detection in Fig. S4 – is it the creepmeter data that suggest this is real?

Reply: As described in the caption of Supplementary Fig. S4 and in our previous response, the signals shown there are clearly instrumental artifacts: they have a fixed short duration, occur only during a limited period of the record, and exhibit an abrupt step-like jump followed by a rapid return to baseline. This behaviour is fundamentally different from the signals shown in Supplementary Figs. S5 and S6, which display the gradual, ramp-like strain evolution characteristic of SSEs. In addition, the events in Figs. S5 and S6 are independently supported by contemporaneous creepmeter observations, which show consistent deformation signals. The durations of these events are within the range of other short-duration SSEs identified in our study. The creep-meter data are publicly available through the USGS deformation database: https://earthquake.usgs.gov/monitoring/deformation/data/download.php

We have also added the corresponding link for the creepmeter to the Data Availability section of the revised manuscript.

C6: How confident are you that the inferred SSE strain changes are not atmospheric pressure changes added to the signals when doing the preprocessing?

Reply: As described in the Methods section of the manuscript (line 426) and illustrated in Supplementary Fig. S17, all preprocessing steps are explicitly documented. In particular, atmospheric pressure effects were corrected using coefficients calculated within the BAYTAP08 framework, following standard strainmeter processing procedures. These corrections were performed using the EarthScopeStrainTools workflow, as described and referenced in the manuscript.

C7: Be more quantitative than 'predominantly'?

Reply: Thank you for this suggestion. We have made this statement quantitative. Based on our inversion results, events consistent with right-lateral motion account for ~89.8% of the total modelled slip magnitude, calculated by considering both the number of events and the amplitudes of their modelled slip relative to those of the non-consistent events. We have revised the manuscript text accordingly to state: "with slip directions consistent with right-lateral motion along the fault. Quantitatively, events consistent with right-lateral motion account for ~90% of the total modelled slip magnitude."

C8: If you require the slip to be right lateral, how well can the data be fit? That is, can you rule out right-lateral slip? If not, this might strengthen the case for these signals being due to SSEs?

Reply: Thank you for your comment. In our inversion, the sense of slip is not imposed but inferred from the strain data. The larger and better-resolved events consistently show right-lateral motion, whereas some smaller-magnitude events display opposite directions that likely reflect weaker constraints due to limited station coverage and low signal amplitudes. We have clarified this point in the revised manuscript by adding the sentence: "For smaller events, the sense of slip is weakly constrained due to limited station coverage and low signal amplitudes.

C9: Please comment on why the strain meter measurements scale like earthquakes (or SSEs for that matter) but are offset (in duration) by several orders of magnitude from the scaling measured for earthquakes!! The simplest model is log(T) = log(M0)/3 – log(K)/3 with K proportional to the stress drop and V^3. Does this offset suggest this offset is either because the stress drops are orders of magnitude lower or the slip velocities are much higher than the values for earthquakes? If they're slow, then it's likely low stress drops – is this because they're so shallow?

Reply: Thank you for this insightful comment. The offset toward longer durations at a given moment could possibly reflect the lower stress drops and slower slip velocities characteristic of SSEs compared to regular earthquakes. This behaviour is consistent with their shallow depth and stable sliding conditions. However, as we do not have clear evidence and to not enter into speculation, we refrain from commenting on this aspect.

C10 & C11: It would be much easier to see the if there is any correlation between LFE and SSE rates if the trends or average rates were removed! Please do this! It is almost impossible

to assess any relation between the curves shown in Figure 13, because the trend overwhelms the changes!
As above, in Figures 5, S14, S15 remove the trends or average rates! The rate changes are what are of interest physically, but these and thus any correlation between them in the different phenomena are buried in the change due to the average rate!

Reply: Thank you for this suggestion. Because LFEs occur frequently, whereas SSEs are sparse episodic events, removing long-term trends from cumulative curves does not provide a meaningful basis for comparison. Instead, we evaluate their temporal relationship using analyses that directly quantify short-term coupling independent of long-term trends. For that reason, we prefer to maintain the cumulative curves in Fig. 5a to illustrate the long-term evolution of both types of events. The other two panels (Fig 5b and Fig 5c) also contribute to evaluating the relative changes between these quantities. Fig. 5b compares mean daily LFE counts relative to SSE occurrence times with those during randomly selected non-SSE periods, demonstrating a systematic increase in LFE activity near SSEs. In addition, Fig. 5c shows the normalized cross-correlation between daily LFE activity and SSE timing, which further quantifies their temporal association. These approaches directly assess rate changes and correlations, which are the physically relevant quantities,

C12: Please add uncertainties, or a measure of the variability in each average LFE daily rate estimate (for the + or – 0,1,2, 3 day lags), on the blue curve and bars in Fig. 5b. In other words, is the difference in daily rate of 2-3 show really significant or meaningful, or is it just within the variability in the rates?

Reply: We thank the reviewer for this suggestion. As requested in the first revision (based on your suggestions), we already quantified the variability of daily LFE rates and reported the corresponding statistics (mean, standard deviation, and interquartile range) in Supplementary Table 1. Following this reviewer's request, we now explicitly show this variability in Fig. 5b by adding error bars representing the standard error of the mean daily LFE rate at each lag.

These results indicate that although daily LFE counts exhibit large intrinsic variability, the ensemble-averaged pattern remains robust: mean LFE activity is systematically higher during and after SSEs than in the preceding days. This conclusion is further supported by the median-based analysis (Supplementary Fig. 16b), which is insensitive to outliers, and by the normalized cross-correlation analysis shown in Fig. 5c. Together, these measures demonstrate that the observed increase in LFE activity following SSEs is not driven by a small number of extreme days but reflects a consistent temporal relationship across the SSE catalog.

C13: Both during non-SSE and SSE intervals?

Reply: Yes. In this robustness test, the restriction to LFE-active days was applied consistently to both SSE and non-SSE intervals. For each lag relative to SSE occurrence, we recomputed the statistics using only calendar days that contained at least one detected LFE, while applying the same criterion to the non-SSE periods. As shown in Supplementary Fig. S16a, this consistent treatment does not change the qualitative result: LFE activity remains systematically higher during and after SSEs compared to both pre-SSE days and non-SSE reference intervals.

C14: Perhaps I am missing the obvious...It seems to me that intervals with no SSEs include intervals 1,2, or 3 days before SSEs? Thus the rates for SSE-1, SSE-2, and SSE-3 should be identical to the rate for the non-SSE intervals (within the uncertainty of the rate measurement),

yet these 'pre- SSE' interval measurements are systematically lower than that for the no-SSE intervals. Why is this? The magnitude of the differences between the rates for the SSE+1, SSE+2, and SSE+3 intervals relative to the no- SSE intervals are comparable or less than those for the differences in the SSE-1, SSE-2, and SSE-3 intervals, so this can't be due to measurement variability if the former elevated (positive differences) are significant? Please explain.

Reply: Thank you for this clarification. In our analysis, the non-SSE reference windows are not allowed to overlap with SSE neighborhoods. Non-SSE windows are selected from periods without SSEs and are required to lie outside the ±3-day interval around any SSE. Therefore, the pre-SSE bins (SSE−3 to SSE−1) are not part of the non-SSE reference set and represent a distinct, conditionally defined population immediately preceding SSEs. We have clarified this definition in the revised manuscript. By adding this: "*Non-SSE reference windows were randomly selected from periods without SSEs and were required to lie outside the −3 to +2 day neighbourhood of any SSE to avoid overlap with pre- and post-SSE intervals.*"

C15: Would this be difficult to reconcile with your inference that the shallower SSEs seem to precede the deeper LFEs?

Reply: These observations are not contradictory because they refer to different processes. The cited studies describe the internal evolution of creep events themselves, where slip initiates at depth and propagates upward, leading to surface slip lagging deeper slip. In contrast, our inference concerns the temporal relationship between two phenomena: shallow SSEs and deeper LFEs. Our results indicate that shallow SSEs are followed by increased activity of deeper LFEs, suggesting a coupling between shallow aseismic slip and deeper fault processes rather than describing the internal propagation of a single slip event.

C16: As above, it would provide additional confidence in the results to know that the pressure measurements do not introduce signals that could be construed as tectonic signals!

Reply: Atmospheric pressure effects are explicitly estimated and removed using collocated barometric data and gauge-specific correction coefficients derived from BAYTAP08. Pressure-induced strain signals are coherent and correlated with barometric fluctuations, whereas the identified SSE signals exhibit distinct transient multi-component strain patterns and show no systematic correlation with atmospheric pressure variations. We have clarified this in the revised manuscript.

Reviewer #2:

The authors have answered most of my comments, thank you.
- ok for the code
- ok for the source analysis and strike-slip assumption
- ok for the reason of the duration of events studied

I am in favor of publishing after few small further comments (minor review):
- validation and hyperparameters:
1) Ok I understand better now how was used the k-means. A new k-means needs to be fitted for every station and every strain component (why?), and a manual selection is done in order to select the SSE-like signals cluster in each. Does some of the kmeans clustering fall in local minima that is not desired?

Reply: K-means clustering is applied separately to each station and strain component because the statistical properties of the strain signals (noise levels, amplitudes, and instrumental responses) differ between stations and between components. Performing a single clustering across all data would mix signals with different distributions and reduce the ability to distinguish SSE-like patterns. However, we agree that joint clustering across stations and components could be explored in future studies.
The clustering procedure itself is fully automatic. No manual selection is applied to individual events; rather, cluster interpretation is based on inspecting representative examples from each cluster to identify the one whose signal characteristics match the expected SSE strain patterns. Regarding the possibility of convergence to undesired local minima, we note that the k-means implementation used in our analysis employs multiple random initializations (n_init = 20) and selects the solution with the lowest within-cluster variance. This ensures stable clustering results across runs and minimizes sensitivity to initialization. We have clarified this point in the revised manuscript by adding: "*K-means clustering was performed separately for each station and strain component to account for differences in signal characteristics, and multiple random initializations (n_init = 20) were used to ensure stable clustering results.*"

2) I think that sometimes it is not clear what hyperparameter tuning mean. Selecting a workflow, an algorithm, over others, and how to organize them (in your case, having 2 k-means clusterings, with 2 k each, with a specific distance metric, etc.) is also part of the hyperparameter tuning. And I guess these choices were made based on the 20% validation set. This could be considered as a light overfitting - you select the workflow that works for that specific case, but we don't know how it performs to new samples. This is even more true since samples were randomized, so training and validation samples are likely very similar (contiguous in time). Nevertheless, since the model itself is unsupervised, I am fine with publishing this without a rigorous separated test set, but it could be added at the end that a further testing on later years/new regions would be needed to fully assess the workflow.

Reply: We thank the reviewer for this thoughtful comment. The autoencoder was trained using standard training and validation splits to ensure stable feature extraction and to prevent overfitting during representation learning. However, the subsequent clustering step is fully unsupervised and does not involve optimization against labeled targets or selection based on validation performance. Therefore, the workflow does not involve hyperparameter tuning in the supervised learning sense.
The choice of two clusters in the k-means step is not tuned using a validation set but is motivated by the physical characteristics of the signals. SSEs produce a distinctive step-like transient in the strain time series, which appears as a spike-like feature in the encoded representation and is clearly separable from background signals. Thus, a two-cluster separation naturally corresponds to distinguishing SSE-like patterns from non-SSE signals.
Because the workflow is unsupervised, it is not optimized for a specific dataset. Nevertheless, we agree that broader testing is valuable, and ongoing work applying this method to additional stations and regions shows consistent performance. To better reflect this in the text, we added the following sentence in lines 585-590 of the manuscript: "*The autoencoder was trained using standard training and validation splits to ensure stable feature extraction, while the subsequent clustering step is fully unsupervised and does not rely on labeled targets. The choice of two clusters and separate clustering for each station and strain component reflects the distinct signal characteristics of SSEs, which produce step-like transient strain changes that are clearly separable from background noise. Further validation on additional years and regions will be valuable to assess its broader applicability.*"

- AutoencoderZ: this is maybe not in the scope of this particular paper, but your answer on the new model, AutoencoderZ, having skip connections is not convincing. Indeed, I understand that with skip connections the reconstruction is better - this why we use them in Unets, to keep more details. But for feature encoding and auto-encoders, since only the features at the coarser scale are kept as encoding, if we add skip connections then it means that some information is 'by-passed' between the finer scales without being encoded in the coarse scale. So the encoding is likely to be less informative, at least on the highest frequencies...

Reply: We thank the reviewer for this insightful comment. We agree that skip connections allow fine-scale information to be transferred directly from encoder to decoder during reconstruction. However, in our workflow, clustering is performed exclusively on the latent representation at the bottleneck layer, not on the reconstructed signals. Therefore, the features used for clustering remain fully constrained by the encoded coarse-scale representation.

The role of skip connections in our architecture is primarily to improve training stability and reconstruction quality by facilitating gradient flow and preserving signal structure during decoding, rather than to bypass the latent encoding. In our previous study, we observed that including skip connections leads to better reconstruction and more informative latent representations (Zali et al ., 2025).

Reviewer #3:

In this new version, the misfits are represented as I had suggested. The reason for this request was the doubts that readers might have about the actual resolution of the values of the slip locations and amplitudes discussed in the paper.
The figures presented seem to confirm these doubts. While the determination of a parameter by inversion is effective if the misfit as a function of the parameter does indeed exhibit convexity around a minimum, it does not appear that this condition is satisfied in this study according to Figures S10 to S12. How can locations or depths be considered resolved under these conditions? Even for slip, there is no minimum in the range of parameters for most cases.
The misfits of the depths presented show characteristics that I find difficult to understand, with very rapid transitions from regimes where the rates of variation with depth are very large to others where they are zero at the precision of the representation. This casts doubt on the figures themselves.
The authors need to be able to justify their results more convincingly. As it stands, the basis for the discussion and conclusions is not established on actual observations.

Reply: We thank the reviewer for this careful assessment of the inversion results. We agree that parameter resolution must be evaluated explicitly, particularly given the limited three-station strainmeter geometry.
Our source analysis is based on a discrete grid search over geometry parameters (x, y, depth, strike, dip, and length). For each trial geometry, slip amplitude is determined analytically by least squares $\varepsilon^{obs} = G.s$, and misfit is computed as the sum of squared residuals between observed and predicted strain components. The misfit curves shown in Figs. S10–S12, therefore represent discrete evaluations over the tested parameter grid rather than continuous analytical functions.
With only three strainmeters, depth and horizontal location are inherently weakly constrained. The broad or flat regions observed in the depth misfit curves reflect limited sensitivity of static strain observations to depth variations for shallow strike-slip sources, leading to extended low-

misfit regions rather than sharply defined minima. Similarly, horizontal misfit maps show diffuse minima consistent with trade-offs imposed by the sparse network geometry.
We have revised the manuscript to clarify that horizontal location and, particularly, depth are weakly resolved and that shallow depths represent preferred solutions within the explored domain rather than uniquely constrained values. The scientific conclusions that we extract in the paper rely on different evidence rather than on precise source location for slip events. The shallow depth of the inferred slip is supported both by the preference for shallow solutions in the misfit analysis and by independent surface creep observations.
The moment–duration scaling primarily depends on slip amplitude estimates, which are less sensitive to geometric uncertainty. We have modified the manuscript to more clearly describe these resolution limitations and to avoid overstating parameter constraints (Line 260- 266, and Line 609-611).

There are still problems with the figures (see Frequency ranges in Supp. Fig 4 to 7).

Reply: We thank the reviewer for pointing this out. We have corrected the labeling in all the related Supplementary figures. The y-axis in the wavelet transform panels is now labeled as wavelet pseudo-frequency [Hz] rather than frequency. We also clarified in the captions that these values are derived from the scale–frequency mapping of the continuous wavelet transform, and therefore correspond to pseudo-frequencies rather than Fourier frequencies.

Editor:

We have now received reports from 1 reviewers and, based on their comments, we have decided to invite a revision of your work. Please address the remaining concerns from the reviewer. In general, we ask you to please further tone down your claims regarding the scaling and the resolution, clearly acknowledge the caveats and potentially offer a suggestion on how this could be improved in future studies. We really need to see these things clarified in order to move forward with the work.

Reply: We thank the Editor for the careful evaluation. We have addressed the remaining concerns by revising the manuscript to more clearly describe the limitations of the three-station inversion, explicitly acknowledging the associated uncertainties, and toning down claims related to source location and scaling. We have modified the relevant text in accordance with the reviewer's and your comments, added a sentence to the abstract, and included a new paragraph in the Discussion to clearly outline the limitations, key caveats, and directions for future work. We have also revised the discussion of the scaling behavior to avoid over-interpretation.

Reviewer #3 (Remarks to the Author):

I note some more careful comments on the poor resolution of locations. The maps are still a bit misleading with the representation of the events by simple points. But at least a careful reader can be aware of the issue.

Reply: We thank the reviewer for this comment. To make this limitation more explicit, we have added the following statement to the figure caption: *"Event locations represent best-fitting models; uncertainties are expected to be large given the limited station geometry."* In addition, we have modified Fig. 4 by removing the depth-related color scale, so that depth is no longer represented in the figure.

The authors have added a comment that I find a bit odd (lines 393–394): As noted by Ide and Beroza (2023), the cubic scaling in ordinary earthquakes is governed by the modulation of elastodynamic interactions, which won't apply in the type of quasi-static deformation represented by SSEs.
What is "the modulation of elastodynamic interactions" ??
Of course there is no available duration for a static process, and the fundamental cubic scaling is with crack length (e.g. Eshelby, 1957). If the medium is elastic, This property should probably be preserved regardless of the process, as even dynamic stress-drop models confirm this.
The problem with slow-slip events is mainly due to the low resolution of depth data, which prevents an adequate assessment of the size of the sliding surface; this issue has been resolved for 'fast' earthquakes by assuming a rupture velocity of the same order of magnitude as that of shear waves for all earthquakes. There is no equivalent of such a constant-velocity assumption for slow-slip processes, but the physical scaling of moment versus length is likely still valid. The new sentence in the text should be corrected.

We thank the reviewer for this comment. The "modulation of elastodynamic interactions" refers to the fact that in all models of dynamic rupture propagation, it is the elastodynamics

(the stress concentration ahead of the rupture front) that leads to continuing rupture. In those models, the rupture velocity is comparable to the seismic wave speed. We introduced this sentence because constant stress drop scaling by itself does not imply a temporal dependence (as the reviewer correctly notes), and the scaling of the duration must come from something else. What Ide and Beroza suggest is that if stress drop is constant, and if earthquake rupture is driven elastodynamically that leads to scaling where both the duration and the length of rupture grow linearly with time. This causes the ruptured area to grow quadratically with time. This combination leads to moment scaling with the cube of time. Because this requires a bit of explanation, we had cited Ide and Beroza (2023), where it is explained in detail. This is an important concept; We would like to clarify this point, and we have added a short explanation.

The revised text now reads: "*Their moment–duration scaling follows a cubic law, similar to regular earthquakes. We note that in dynamic earthquake models, it is the elastodynamic stress concentration ahead of the rupture front that sustains rupture. In those models, the rupture velocity is comparable to the seismic wave speed. Ide and Beroza (2023) suggest that elastodynamic rupture leads to scaling where both the duration and the length of rupture grow linearly with time, which leads to moment scaling with the cube of time if the stress drop is constant. For slow slip events, the cubic scaling with duration must have another origin; however, given the limited resolution of source dimensions, particularly depth and fault area, this scaling should be interpreted with caution. In particular, uncertainties in the size of the slipping area may affect the inferred relationship.*

In addition, we have added the following sentence to the abstract: "*However, given the limited three-station geometry, source parameters remain weakly constrained, and denser geodetic observations could help to better resolve event locations and scaling.*"

We have also added the following paragraph to the Discussion to explicitly outline the limitations of the inversion and their implications for the scaling analysis, as well as directions for future work: "*The limited three-station strainmeter geometry strongly constrains the resolving power of the source inversion. Depth and horizontal location are weakly constrained and should be interpreted as approximate, preferred solutions rather than uniquely resolved parameters. This limitation also affects the estimation of source dimensions and can introduce uncertainty in the interpretation of scaling relationships. Future work combining denser geodetic networks, additional independent observations (e.g., GPS or creepmeters), and improved constraints on fault geometry could help to better resolve source properties and to more robustly assess the scaling behavior of short-term slow slip events.*"

We believe these revisions address the reviewer's concerns and improve the clarity and robustness of the manuscript.
